# Photochemical Manganese-Catalyzed [2 + 2 + 2] Cycloaddition Reactions

**DOI:** 10.1021/acscatal.5c00349

**Published:** 2025-03-24

**Authors:** Benedikt N. Baumann, Phong Dam, Jabor Rabeah, Christoph Kubis, Angelika Brückner, Haijun Jiao, Marko Hapke

**Affiliations:** § Institute for Catalysis (INCA), Johannes Kepler University Linz (JKU), Altenberger Strasse 69, Linz 4040, Austria; # Leibniz Institute for Catalysis e.V. (LIKAT), Albert-Einstein-Strasse 29a, Rostock 18069, Germany

**Keywords:** manganese, [2 + 2 + 2] cycloaddition, oligoalkynes, reaction mechanism, arenes

## Abstract

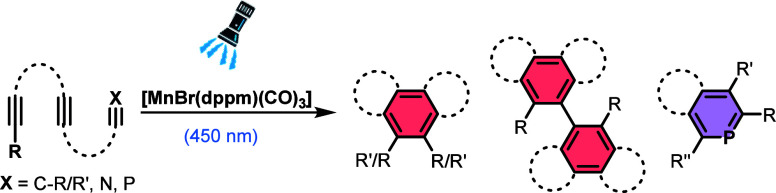

We report the cyclotrimerization reactions of triynes using Mn(I) complexes derived from MnBr(CO)_5_ and phosphine ligands, such as 1,1-bis(diphenylphosphino)methane (dppm). These reactions are driven by irradiation under mild conditions (30–80 °C) without the need of additional photoinitiators. Our catalytic screening revealed that counteranions and ligands significantly influence the process. This method accommodates a broad range of functionalities in the substrates, including alkyl, aryl, Bpin, SiMe_3_, GeEt_3_, PPh_2_, pyridyl, and thienyl moieties, without notable interference in the transformation. Additionally, this method enables reactions with oligoalkynes-like (un)substituted hexaynes, producing 2-fold cyclization products in very good yields. Under stoichiometric conditions, the cyclization of diynes with phosphaalkynes results in the unique photochemical synthesis of phosphinines. Experimental and theoretical mechanistic studies indicate that the dissociation of the diphosphine ligand precedes the involvement of the Mn carbonyl species in the catalytic cycle. The ligand plays a crucial role in stabilizing the catalyst during the catalytic transformation and preventing the formation of unreactive cluster species.

## Introduction

The *de novo* synthesis of (hetero)aromatic compounds from smaller building blocks has become an attractive target over the last decades, benefiting from the rapid development of synthetic methods in organic syntheses and (bio)catalytic science. In many cases, such transformations require a multistep approach to assemble the aromatic core, and the extension of the aromatic system to a larger carbo- or heterocyclic congener is a common methodology. The toolbox for the one-step construction of (hetero)arenes from simple alkyne building blocks is limited, with the cyclotrimerization reaction of alkynes and heterocumulenes being the prime example.^1^ Such a reaction requires catalytic facilitation to overcome the intrinsically high activation barrier, which was theoretically described for the formation of benzene from acetylene.^2^ Transition metal catalysts for the (catalytic) mediation of [2 + 2 + 2] cycloaddition reactions of an increasingly large substrate array comprising alkynes, oligoalkynes, nitriles, and other heterocumulenes have evolved toward maturity for metals from groups 8–10, namely, ruthenium, cobalt, rhodium, iridium, and nickel.^3−5^ Besides these commonly used transition metals, other metals including, e.g., niobium,^6^ molybdenum,^7^ iron,^8^ or palladium^9^ have seen less significant evolution or have just been investigated for a limited scope of substrates but reveal interesting novel possibilities for cyclotrimerizations. Especially 3d transition metal catalysis can open new alleys of reactivity, as was showcased recently by the catalytic utilization of iron^10^ and cobalt^11^ for the cyclotrimerization of unusual substrates like phosphaalkynes with alkynes, leading to substituted phosphinines. Although metal-free formal cyclotrimerizations have been reported,^12^ they include alternative pathways or reactions under more drastic conditions to yield the cycloaddition products. Only quite recently, electro- and photochemical methodologies have been disclosed for transition metal-free cyclotrimerization reactions of alkynes and nitriles, regioselectively furnishing substituted pyridines.^13^

In general, for the middle range of 3d transition metals like iron, manganese, or chromium significantly fewer catalytic procedures for the assembly of alkynes to furnish benzene derivatives are known so far. While iron is the pacemaker among those, chromium has only seen exemplary stoichiometric application, in combination with other metals.^14^ Iron-catalyzed cyclotrimerization reactions include particular precatalysts such as isolated metalates, *in situ* generated systems, bimetallic catalysts, and electro- and photochemically driven transformations. Also, CpFe-based catalysts have been reported for successful cyclotrimerization reactions to furnish benzenes and pyridines.^15^ Concerning the group 7 metals, only manganese and rhenium compounds are catalytically applied in organic synthesis, particularly in C–C and C–H activation reactions.^16^

**Scheme 1:**
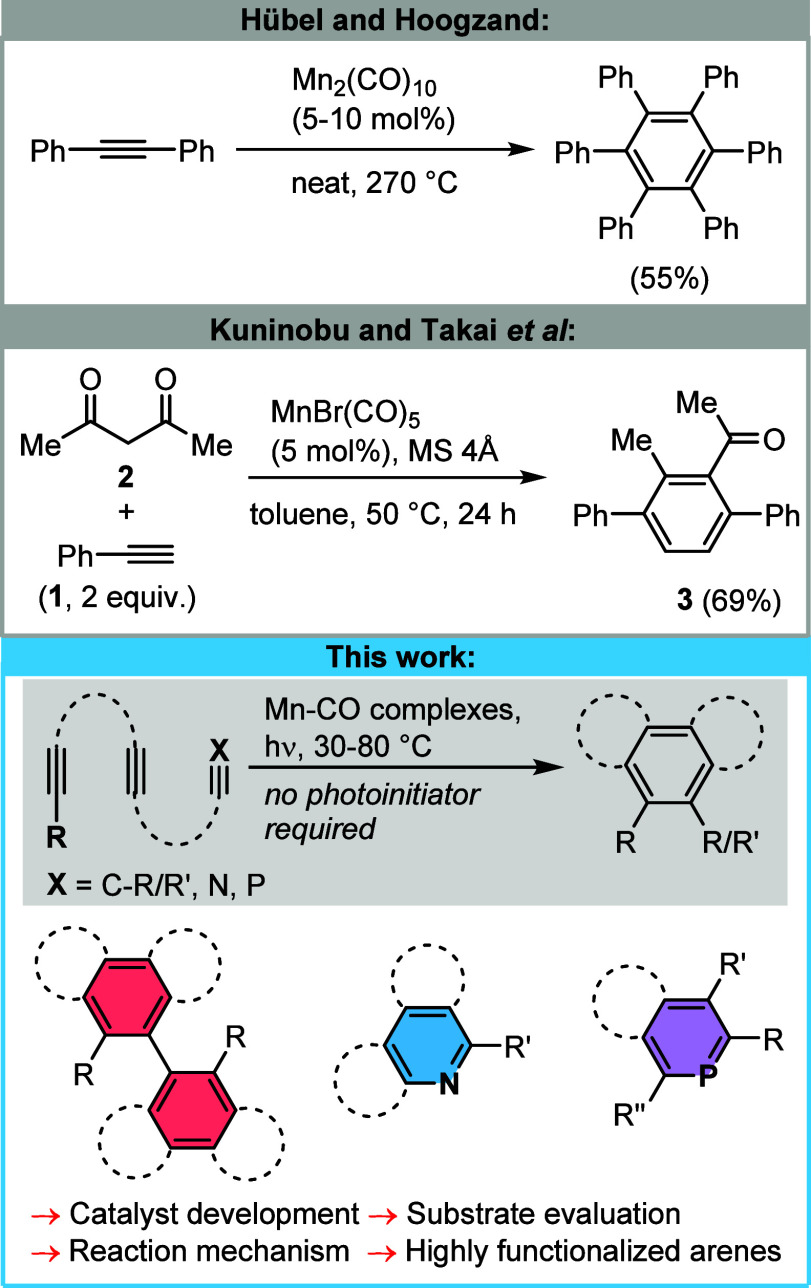
Known and Novel Manganese–Carbonyl-Catalyzed Formal Cyclotrimerizations of Alkynes toward Arene and Heteroarene Products

Recently reported manganese-catalyzed transformations include hydrogenation, transfer hydrogenation, hydrohetero-functionalization, and cross-coupling reactions as well as radical-mediated reactions, among others.^17,18^ Very few singular examples appear as formal cyclotrimerization reactions and reveal peculiarities concerning substrates and mechanisms compared to other transition metal complexes.^19^ One of the earliest examples was reported in a general investigation of transition metal carbonyls for the cyclotrimerization of diphenylacetylene to hexaphenylbenzene under rather drastic conditions using Mn_2_(CO)_10_ (Scheme 1, top).^20^ The Mn(I)-catalyzed cyclization of phenylacetylene (**1**) and 2,4-pentanedione (**2**) led to the formation of substituted *p*-terphenyl product **3** (Scheme 1, middle).^21^

Such cyclization reactions are also known in very few cases for the rhenium complexes [Re_2_(CO)_10_] or [ReBr(CO)_3_(THF)_2_], also furnishing substituted arenes from 1,3-dicarbonyl compounds and terminal alkynes. However, the mechanistic rationale for these transformations rather includes formal [2 + 1 + 2 + 1] or [2 + 2 + 1 + 1] cycloaddition steps than the known cyclotrimerization pathways.^22^ Recently, an approach toward Mn-catalyzed cyclotrimerization using photoredox catalysis was reported, covering the reaction between diynes and acetylenes as a classical substrate combination.^23^ This methodology required large catalyst loading of a Mn(II) salt such as Mn(acac)_2_, an additional photoredox catalyst, chlorobenzene as a (partial) solvent, and reaction times up to 12 h. Another formal cyclization of diynes with indoles leading to annulated benzenes was reported via C–H annulation requiring a 2-pyridyl directing group for the sequential alkyne insertion.^24^

The reactivity of Mn_2_(CO)_10_ is in some aspects reminiscent of the reactivity of Co_2_(CO)_8_, which has seen increasing application as a cyclotrimerization catalyst in recent years.^25^ These binary metal carbonyl complexes share significant structural similarities, as they are dinuclear complexes, with two additional CO ligands on the manganese complex to fulfill the formal 18-electron rule. The use of Co_2_(CO)_8_ in alkyne chemistry is well documented as it can act beside its catalytic activity as a unique protecting group of the alkyne moiety.^26^ On the contrary to manganese, the CoX(CO)_4_ (X = I, Br) compounds are rather unstable and delicate to handle, while congeners such as MnBr(CO)_5_ are well-known and stable as well as commercially available. The catalytic potential of manganese in comparison to its higher 3d metal congeners iron and cobalt was revealed in recent years, particularly for (de)hydrogenation reactions.^27^ An interesting connection between transition metal compounds and catalytic activity was proposed in terms of diagonal relationships in the periodic table.^28^ Here, a connection in the diagonal triad Mn–Ru–Ir was discussed for hydrogenation and transfer hydrogenation reactions, aligning comparable reactivities for Mn(I) as well as Ru(II) complexes. This is particularly interesting since Co(I) and Ru(II) complexes are frequently utilized catalysts in catalytic [2 + 2 + 2] cycloaddition reactions.

In a number of investigations, manganese carbonyls were used directly as catalysts under photochemical conditions, also due to the ease of modification by substitution of the CO groups with other heteroatom ligands. We envisioned the possibility of exploiting this reactivity for cyclotrimerization reactions of triynes and heterocumulenes under photochemical conditions, thus possibly allowing particularly mild conditions and the opportunity to fine-tune the reactivity via additional ligands. We also concentrate on unusual substitution patterns and structures in the alkyne substrates, including functionalization with heteroatom-containing groups. Subsequent cyclization should result in the formation of arenes which are difficult to synthesize and open new venues toward the preparation of functional molecules (Scheme 1, below).

## Results and Discussion

In the initial experiments, we used compound **4** as a conventional triyne as the testing substrate to evaluate the general reactivity in cases when the catalyst is not expected to undergo a reaction with the terminal C–H bonds. A thermal blind reaction (microwave, 150 °C, 2 h) under the exclusion of light did not yield any cyclization product. The initial screening reactions were performed at 30 °C under irradiation at 450 nm wavelength for 30 min, to identify any reactivity at this mild temperature, utilizing 10 mol % of manganese, rhenium, or molybdenum catalysts. The results of this screening are displayed in Table 1 for the precatalysts **Mn1–5** (entries 1–5), **Re** (entry 6), and **Mo** (entry 7). The catalytic activities of these complexes are quite different, with **Mn1** and **Mn2** being the most active neutral complexes. Except for Mn_2_(CO)_10_ (**Mn5**) all other metal carbonyls provided very small amounts of **5**, including the heavy group congener rhenium (**Re**). Further screening results including reactions in different photochemical reaction setups with **Mn1**–**Mn5** and solvents are compiled in the Supporting Information.

**Table 1:**
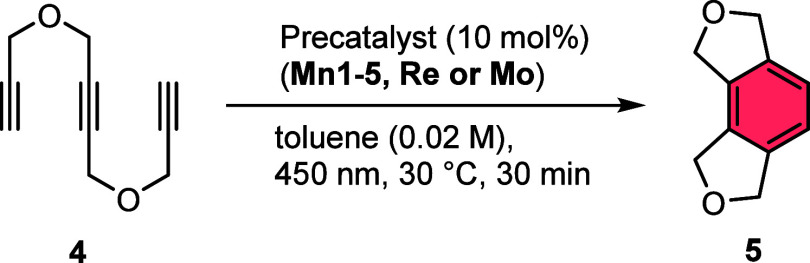
Screening of Manganese(I) Precatalysts

#	precatalyst	yield **5** [%]^a^
1	MnBr(CO)_5_ (**Mn1**)	42
2	MnCl(CO)_5_ (**Mn2**)	27
3	Mn(OTf)(CO)_5_ (**Mn3**)	11
4	Cp’Mn(CO)_3_ (**Mn4**)	<5
5	Mn_2_(CO)_10_ (**Mn5**) (5 mol %)	20
6	ReBr(CO)_5_ (**Re**)	<5
7	Mo(CO)_6_ (**Mo**)	11

a Determination of yield by GC-MS with external calibration.

After investigating the general reactivity of the simple manganese–carbonyl complexes, we started our further investigation with the screening of different ligands toward their influence and modification of the catalytic performance of the most suitable manganese(I) precursor **Mn1** (Table 2). As constraints, we utilized the same substrate and overall reaction conditions but kept the reaction times short (15 min) to evaluate the productivity of the *in situ* formed catalyst system. The addition of PPh_3_ (**L1**) as a simple monodentate ligand already led to a nearly complete conversion of triyne **4** and a very high yield of **5** (entry 1, Table 2).

**Table 2:**
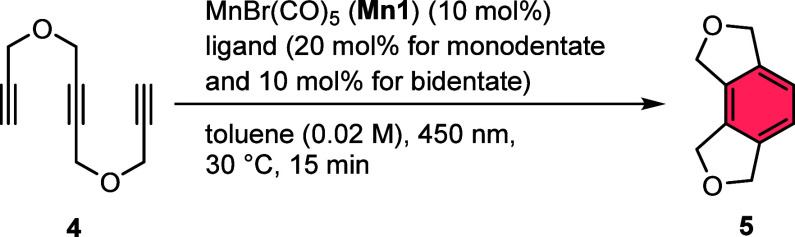
Screening Results from Reactions with **Mn1** and Mono- and Bidentate Ligands

#	ligand	yield **5** [%]^a^
1	triphenylphosphine (**L1**)	98
2	triphenylphosphite (**L2**)	46
3	tris(1-naphthyl) phosphine (**L3**)	28
4	tris(pentafluorophenyl) phosphine (**L4**)	18
5	triisopropylphosphine (**L5**)	51
6	1,1-bis(diphenylphosphino) methane (dppm, **L6**)	>99
7	1,2-bis(diphenylphosphino) ethane (dppe, **L7**)	<5
8	1,3-bis(diphenylphosphino) propane (dppp, **L8**)	<5
9	1,2-bis(diphenylphosphino) benzene (dppbenz, **L9**)	<5
10	no ligand	24

a Determination of yield by GC-MS with external calibration.

Application of other phosphines with different steric or electronic properties or triphenylphosphite (**L2-L5**) led to rather low yields (entries 2–5, Table 2). The application of the chelating ligand 1,1-bis(diphenylphosphino) methane (dppm, **L6**), however, led to a significant improvement of the yield to >99% (entry 6, Table 2), only comparable to the use of PPh_3_ (**L1**, 98%) as a ligand (entry 1, Table 2). Investigation of other chelating ligands (**L7**–**L9**) did not lead to improved product formation (entries 7–9, Table 2). The test reaction using only MnBr(CO)_5_ (**Mn1**) resulted in 24% product formation after irradiation for 15 min without any additional ligand, clearly demonstrating the beneficial effect of **L1** and **L6** (entry 10, Table 2). From these experiments, it was concluded that the phosphine ligands **L1** and **L6** presumably modify the catalyst system toward a higher cyclization rate and longer activity, preventing early deactivation as can be presumed for the ligand-free system only using **Mn1** as a precatalyst.

**Chart 1:**
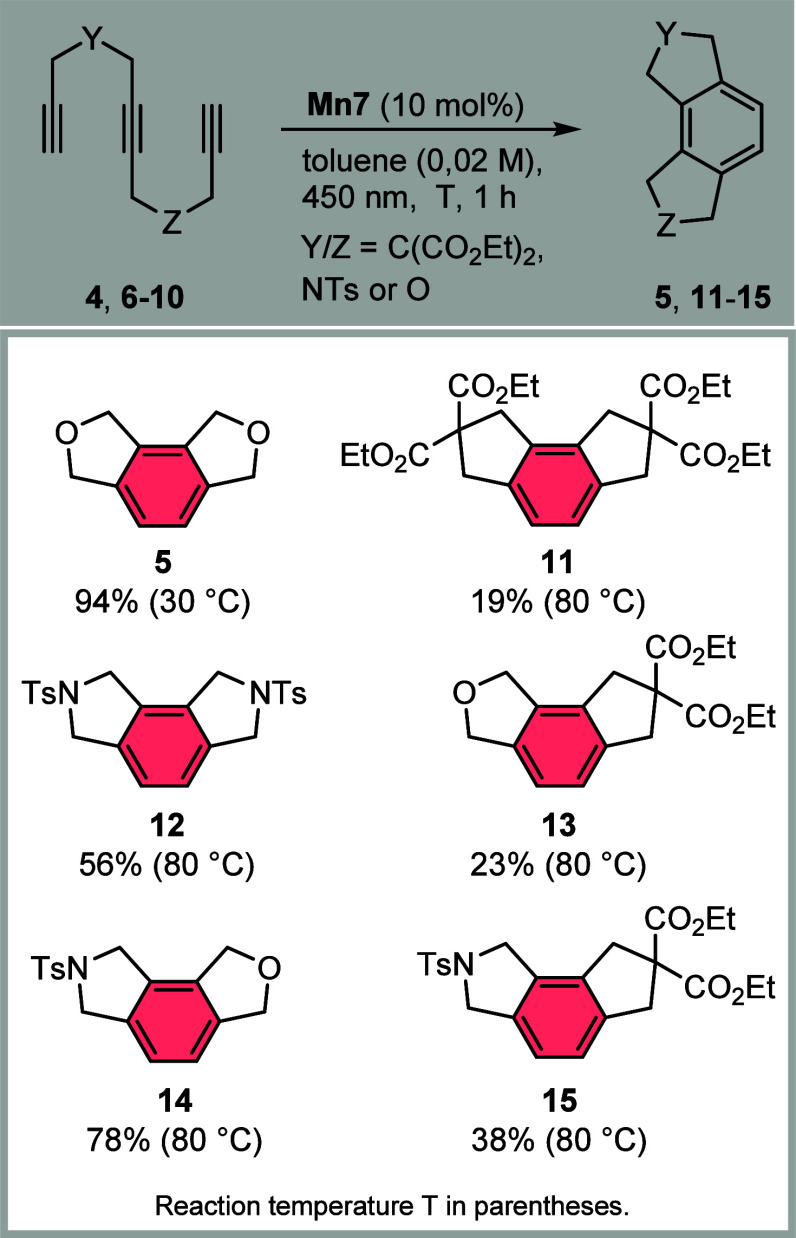
Investigation of Triynes Comprising Different Alkyne-Bridging Units

Following evaluation of the optimal catalyst system, we turned our attention to screen the substrate scope for this photocatalytic Mn(I)-diphosphine system (Chart 1). The molecular precatalyst *fac*-MnBr(CO)_3_(dppm) (**Mn7**) was conveniently synthesized from MnBr(CO)_5_ (**Mn1**) and dppm (**6**) and isolated with 91% yield as pure, easy to handle, and for short periods air-stable solid. Cyclization studies using terminally unsubstituted and differently bridged triynes (**4**, **6**–**10**) yielded the corresponding arene products **5** and **11**–**15** with different yields at 30 and 80 °C reaction temperatures (Chart 1). The results indicated that ether-bridging is superior to exclusive bridging by malonate (**11**) or amide (**12**) tethers. Triynes containing different bridging units while keeping the tether length identical, however, were obtained in moderate yields, corroborating the importance of the nature of the bridging groups. The best result was obtained by an ether-amide bridging to yield the arene **14** with 78% yield after irradiation at 80 °C for 1 h.

**Chart 2:**
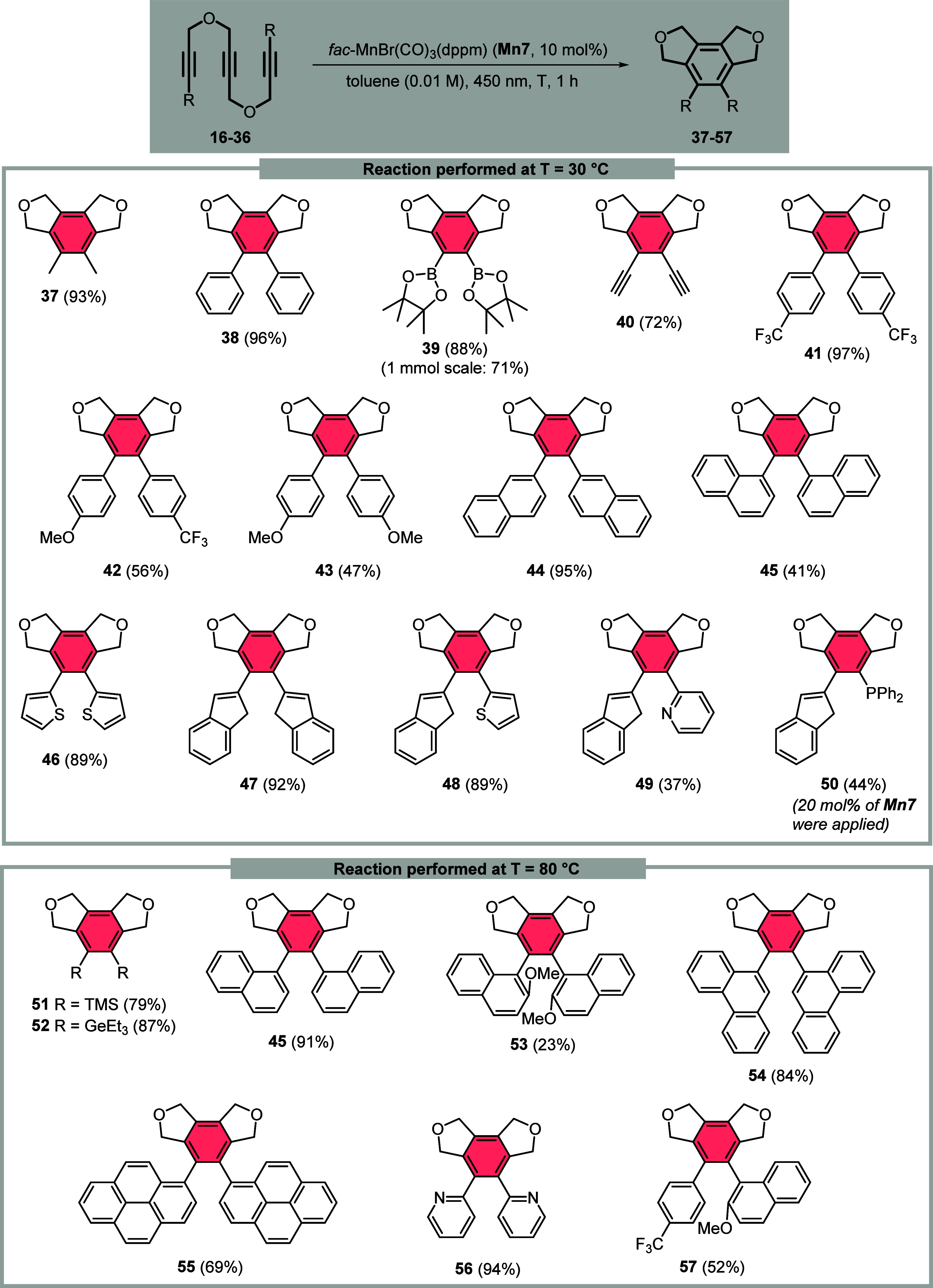
Substrate Screening for the Photocatalytic Mn(I)-Catalyzed [2 + 2 + 2] Cycloaddition Reaction Using a Variety of Functionalized Triynes (Isolated Yields)

The substrate scope investigation was therefore extended to functionalized triynes **16**–**36**, based on the overall structure of ether-bridged triyne **4** (Chart 2). We established efficient synthetic pathways for these substrates to provide a large structural variety of different functional groups and useful amounts of cyclization substrates for further elaborations. These results demonstrated the efficiency of the catalytic system for converting even standard triynes to the expected products **37** and **38** with excellent yields at 30 °C. The synthesis of the *ortho*-diborylated arene **39** with 89% yield is particularly remarkable, as the identical transformation of the terminally diborylated precursor using a CpCo(I) precatalyst under microwave conditions at 200 °C gave only 24% of **39** beside partially or completely deborylated side products.^29^ The cyclization of a previously unknown and newly synthesized pentayne **19** containing two terminal 1,3-butadiyne units was also successful under these very mild reaction conditions, yielding the 1,2-bisethinylated fully substituted benzene **40** with a 72% yield. The influence of electronically different groups in the *para*-position of the terminal arene introducing CF_3_ or OMe substituents was investigated for triynes **20**–**22**. Excellent results were only obtained for CF_3_ groups as shown for product **41**, comparable to compound **38**. On the other hand, the unsymmetrically or the symmetrically *para*-methoxy-functionalized triynes **21** and **22** led under identical reaction conditions to a lower yield of **42** (56%) and **43** (47%). An interesting position of substitution effect has been found in triaryls **44** and **45**, where at 30 °C the terminal 2-naphthyl-substituted triyne **24** was converted with an excellent yield to triaryl **44**, compared to the significantly lower yield for the conversion of terminal 1-naphthyl-substituted triyne **25**. Increasing the reaction temperature to 80 °C, however, led to the cyclization of triyne **25** with up to 91% yield, compared to 41% yield at 30 °C to the triaryl **45**, indicating the necessity of higher reaction temperatures with increasing steric demand of the arene. The methodology also allowed the cyclization of 2-thienyl-substituted triyne **25** with an impressive 89% yield at 30 °C, leading to the unknown bisthienyl compound **46**. An intriguing synthesis of an *ortho*-di-2-indenyl substituted arene **47** could also be realized in 92% yield, delivering a potential new ligand blueprint for metallocene synthesis. We further exploited this possibility by synthesizing unsymmetrical, 2-indenyl-substituted triynes **27–29** with additional heteroatom-containing substituents. In this case, the aryl-substituted 2-indenes **48**, **49**, and **50** could be successfully obtained with yields up to 89% at only 30 °C. Turning our attention also toward further heteroatom-functionalized triynes, the cyclization of triyne substrates **30** and **31** gave the 1,2-bissilylated and 1,2-bisgermylated compounds **51** and **52** with very good yields. Substitution of the triyne backbone with 2-methoxy-1-naphthyl groups led to a 23% yield for triaryl **53** under standard cyclization conditions at 80 °C, while the unreacted **32** was reisolated in pure form. This is another significant advantage of the presented methodology, in which unreacted triyne substrates can often be recovered after the reaction, and no significant side reactions are encountered. However, attachment of phenanthrene or pyrene groups allows the isolation of triaryls **54** and **55** with up to 84% yield. Interestingly, when triyne **35** bearing 2-pyridyl substituents was subjected to cyclization, heterotriaryl **56** was obtained with a nearly quantitative yield. Finally, applying an unsymmetrically substituted triyne containing the sterically rather bulky 2-methoxy-1-naphthyl group, triaryl **57** was still isolated in 52% yield. As a general trend, sterically large terminal aryl substituents require elevated reaction temperature to reach maximum conversion. From the initial studies using an air-cooled photoreactor, we switched to a water-cooled reactor for the entire study, allowing precise temperature control over the entire reaction time. The role of temperature in photocatalytic reactions has been investigated for several photoreactor models for a number of selected reactions and it was found that the temperature can exert a significant influence, either in a negative or positive fashion.^30^ The overall influence of temperature as a parameter in photocatalytic reactions has only rarely been studied systematically.^31^ For cobalt-catalyzed cyclotrimerization reactions using gaseous acetylene and nitrile substrates, the temperature dependence of the reaction outcome was causing side product formation (benzene) by influencing the solubility of the acetylene in the reaction solvent.^32^ In our case, solubility issues with dependence on the reaction temperature have never been observed.

**Chart 3:**
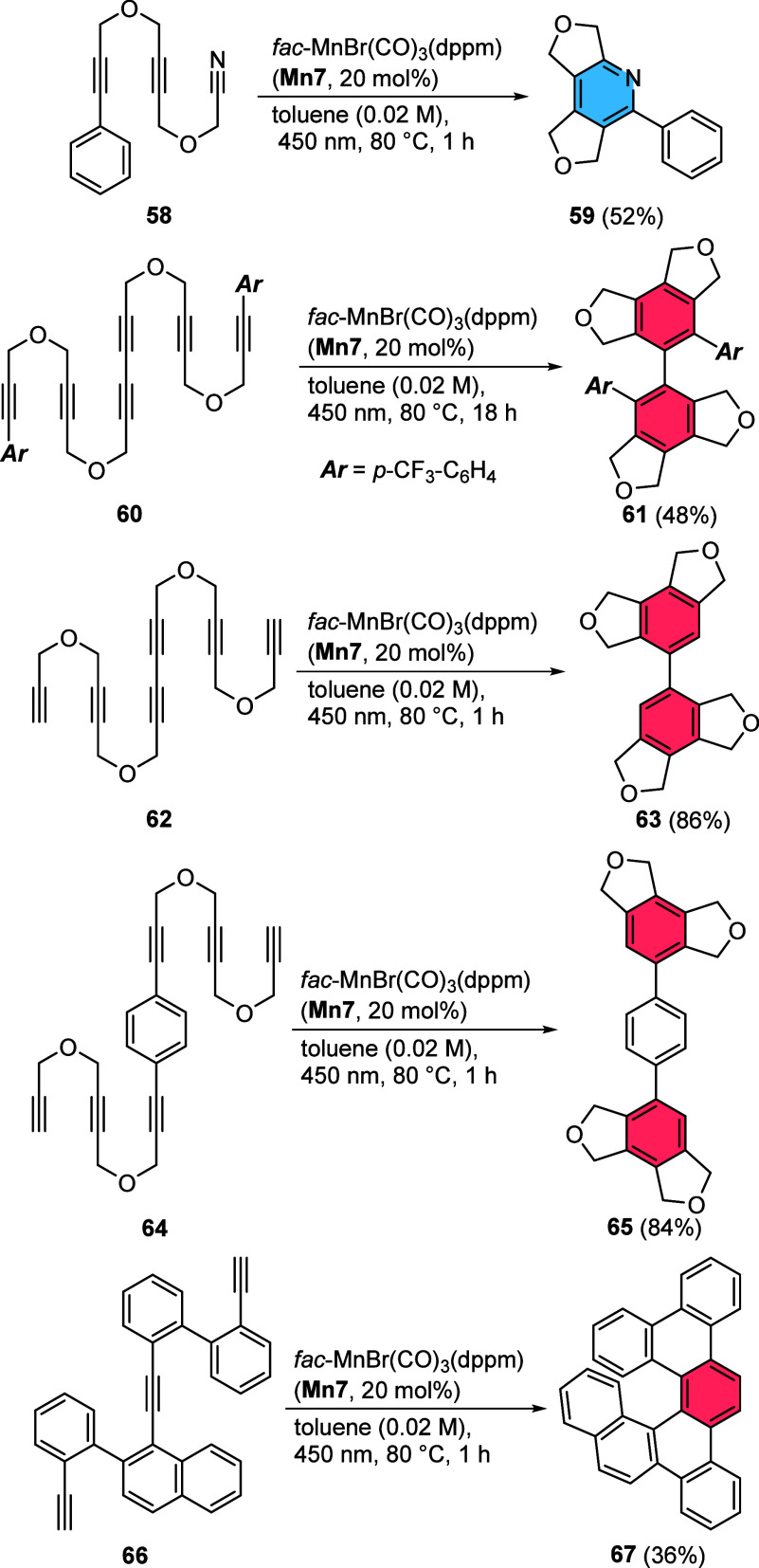
Showcase of Complex Substrates for Successful Cyclotrimerization Reactions Using Precatalyst **Mn7** (Isolated Yields)

Further reactions with selected structurally challenging reaction partners revealed the potential of the Mn-catalyzed transformation (Chart 3). Besides intramolecular cyclizations, we investigated the intermolecular reaction of phenylacetylene as well as benzonitrile with dipropargyl ether as substrates for the synthesis of the corresponding carbo- or heterocyclic products with our catalyst system; however, under the investigated reaction conditions only in the case of the phenylacetylene, the formation of the corresponding 1,3,5- and 1,2,4-triphenylbenzenes in a regiomer ratio of ca. 1:1 with 12% overall product yield was observed.

We switched to a completely intramolecular reaction using cyanodiyne **58** and found the formation of pyridine **59** with 52% yield at 80 °C. Obviously, the formation of pyridines in principle is possible but ideally requires a completely intramolecular approach under the investigated conditions. For the next example, we developed efficient synthetic approaches toward the hexaynes **60**, **62**, and **64**.^33^ The subsequent cyclization under optimized reaction conditions led to the formation of the fully substituted biphenyl derivative **61** in 48% yield, while unreacted starting material could simply be reisolated in pure form. The unsubstituted hexayne **62** as well as the phenyl-bridged hexayne **64** were converted to the biphenyl or *para-*terphenyl derivatives **63** and **65** with excellent 86 and 84% yield. Again, the transformations leave only separable unreacted starting materials behind. The cyclization of the arene-bridged triyne **66** resulted in the formation of the helical structure **67** with 36% yield. These examples illustrate the capabilities of photocatalyzed cyclotrimerizations based on Mn(I)-carbonyl catalysts for rapid transformation of complex substrates at mild temperatures.

**Chart 4:**
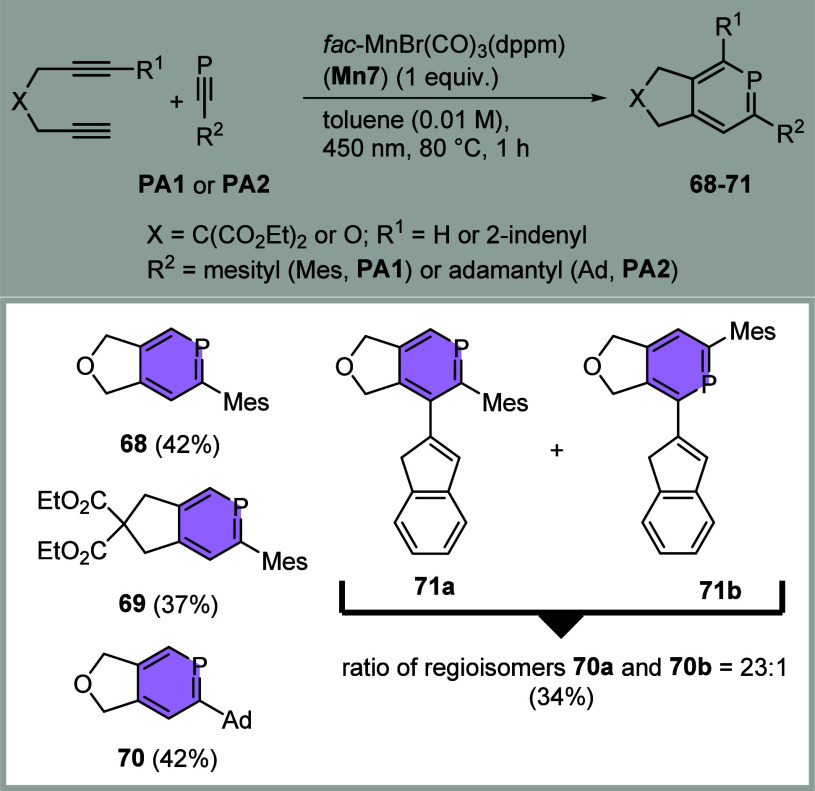
[2 + 2 + 2] Cycloaddition of Diynes and Phosphaalkynes Using **Mn7** in Stochiometric Amounts (Isolated Yields)

We further investigated the reaction of diynes with phosphaalkynes, which was recently reported using 3d transition metal catalysts based on Fe^II^ or Co^II^ compounds for the formation of phosphinines.^10,11,34^ Reactions of phosphaalkynes with low-valent metal complexes often lead to the (undesired) formation of P-ring systems, which strongly coordinate the catalyst and inhibit further reactions.^35^ Manganese complexes with phosphinines are also reported.^36^ The reactivity of the C≡N and the C≡P moieties in cycloadditions is quite dependent on the catalyst due to their different electronic properties, with the phosphalkynes having more similarity with a C≡C triple bond than the nitriles.^37^ We set out to evaluate possible reactivity in the reaction of phosphaalkynes with diynes using higher amounts of the most efficient precatalyst **Mn7** under irradiation. To our delight, transformations between diynes and mesitylphosphaalkyne (**PA1**) or adamantylphosphaalkyne (**PA2**) applying stoichiometric amounts of **Mn7** were found to be successful and delivered the phosphinines **68**–**71** with yields of up to 42% (Chart 4).

Dipropargylether as well as dipropargylmalonate worked with **PA1** to give products **68** and **69**, and also **PA2** reacted in a similar yield to product **70**. Dipropargylic ether proved to be particularly suitable as already observed for the cobalt catalysis, since homocyclization of the diyne is a neglectable process.^38^ Interestingly, in the formation of **71a** and **71b** the more crowded substituted phosphinine **71a** is formed in a large excess. This is, to the best of our knowledge, the first photo- and transition metal-mediated phosphinine synthesis under rather mild conditions reported yet.

**Chart 5:**
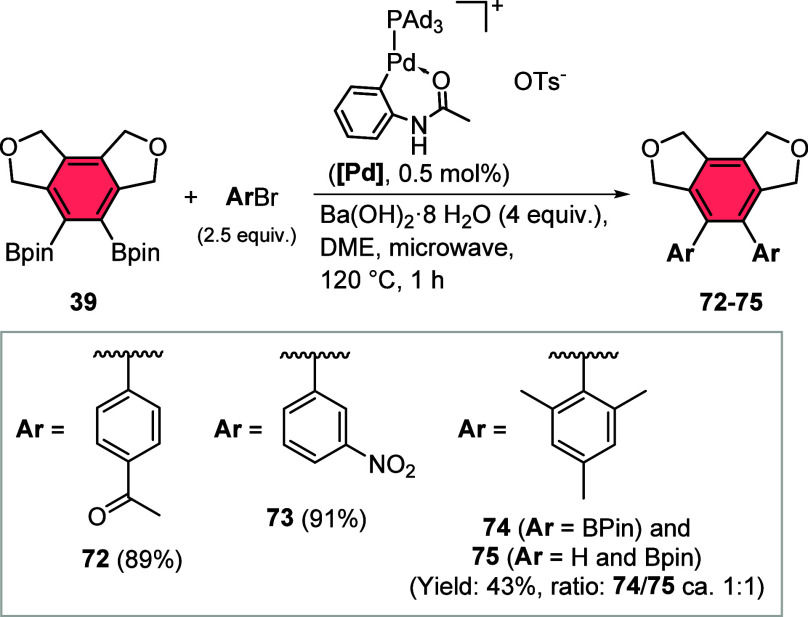
Follow-up Chemistry with Compound **39**: Synthesis of Highly Substituted Benzenes by Suzuki–Miyaura Reactions with Aryl Bromides (Isolated Yields)

Since our catalyst system allowed the facile preparation of larger amounts of the bisborylated compound **39** it can be regarded as a versatile platform for the introduction of substituents in the 1,2-position for the formation of hexasubstituted benzenes. Naturally, as the most common feature, we evaluated the possibility of further elaboration by Suzuki–Miyaura cross-coupling reaction of **39** under standard conditions using a highly active palladium catalyst and selected functionalized aryl halides as coupling partners (Chart 5). Initial experiments proved the complex **[Pd]** as a very suitable precursor for rapid reactions under convenient conditions, possessing tri(1-adamantly)phosphine (PAd_3_) as an electron-rich monodentate ligand.^39^ Coupling reactions with aryl bromides bearing substituents in the *meta*- and *para*-position gave products **72** and **73** with excellent yields of up to 91%. Attempted coupling with sterically demanding 1-bromomesityl also proceeded to the highly substituted desired product **74**, however, with this challenging substrate also protodeboronation occurred to yield the monocoupling product **75**. Both products were obtained in a roughly 1:1 ratio with a 43% overall yield.

## Discussion of Mechanistic Aspects

Furthermore, we are interested in the reaction mechanism of this unique and unusual photocatalytic cyclotrimerization process, applying manganese(I) complexes. Reaction mechanisms in the field of transition metal-catalyzed [2 + 2 + 2] cycloadditions have been known to follow a general overall pathway with a large variety of mechanistic intricacies, depending on the catalyst metal applied.^40^ As a common intermediate in most cases regardless of the metal, however, the formation of metallacyclopentadienes from two alkyne moieties was proposed and is now widely accepted based on experimental and theoretical data.^41^ In the case of manganese, conceivable and isolable manganacyclopentadiene intermediates have not yet been postulated. The (thermal) stoichiometric reaction of 1,6-diynes with MeMn(CO)_5_ also follows a unique route, as demonstrated by an investigation by Hong and Chung.^42^ It proceeded via the manganaacetylation of a triple bond and the formation of an isolable compound. This reaction involves the insertion of a carbonyl ligand instead of a metallacyclopentadiene intermediate during the formation of [2,3]-fused cyclopentadiene compounds. Subsequent oxidation with trimethylamine oxide and hydrolysis furnished the [2,3]-annulated cyclopentadienes possessing a tertiary alcohol group. The insertion step is well-known for cobalt complexes possessing CO ligands, exemplified in [2 + 2 + 1]-type cycloaddition reactions of alkynes,^43^ including photochemical assistance in the exchange of CO groups for alkynes in the initial part of the catalytic cycle.^44^ A potential intermediate of the cobalt-catalyzed process has been isolated by the group of Vollhardt.^45^ Detailed studies on organometallic intermediates for the reaction of manganese–carbonyl complexes toward cyclization-type transformations with alkynes are scarce. On the contrary, significant effort is devoted to the photochemistry of manganese–carbonyl complexes containing N-heterocyclic ligands, e.g., for CO_2_ reduction or CO release.^46,47^

**Scheme 2:**
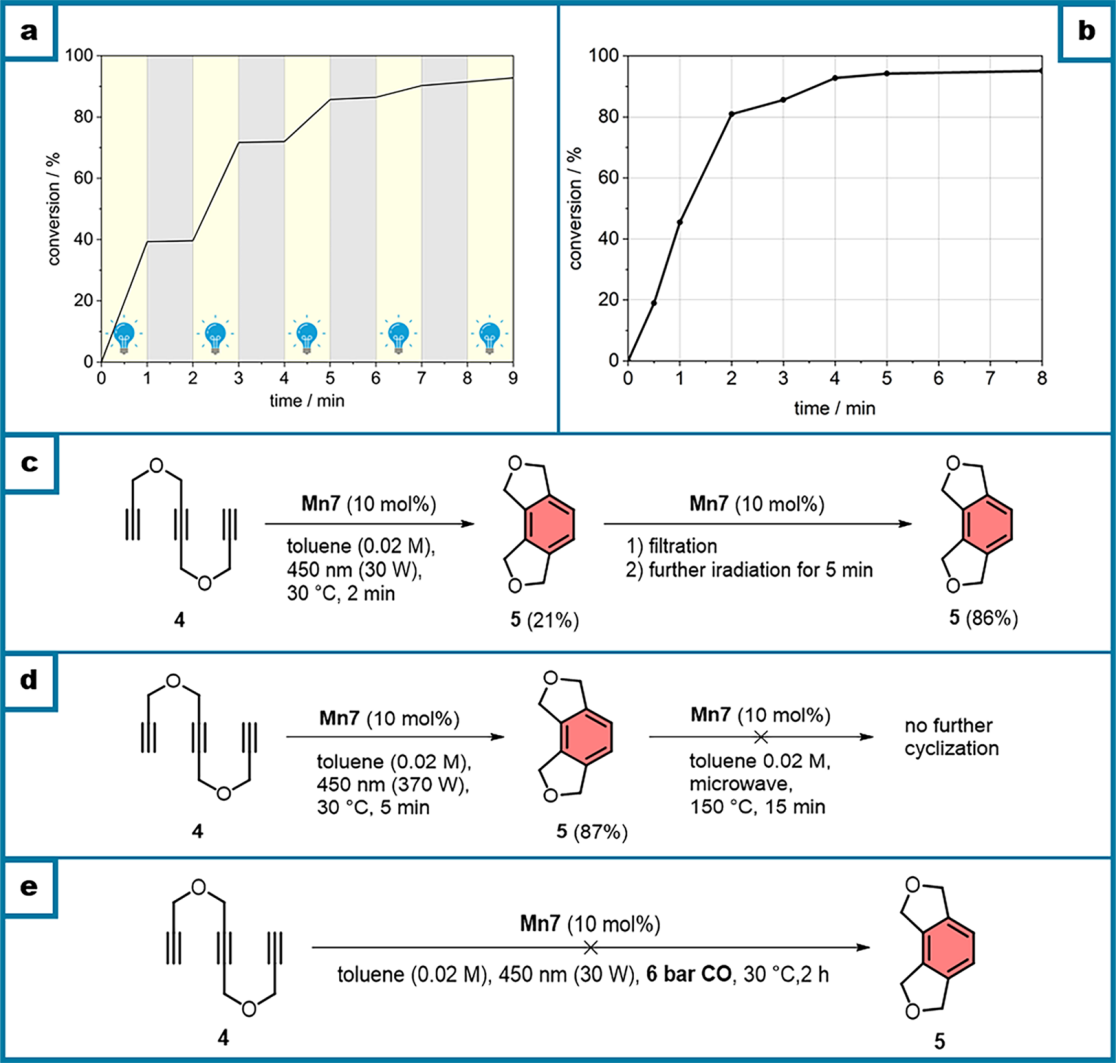
(a) Light On–Off Experiment Using **Mn7** and Triyne **4**, (b) Time–Yield Plot for the Cyclization Reaction Triyne **4** Using **Mn7**, (c) Filtration Test, (d) Control Experiments to Exclude Thermal Background Reactions, and (e) Attempted Cyclization under CO Atmosphere

Since mainly cobalt complexes are known for versatile and general photocatalyzed cyclotrimerization reactions without any photoinitiator, we investigated the role of irradiation for the manganese catalysis and performed a light on–off experiment using triyne **4** (Scheme 2a). It clearly showed that the reaction only proceeds under irradiation. We also investigated the conversion–time relationship with triyne **4** and found that the reaction is very rapid, with approximately 80% conversion after 2 min (Scheme 2b). Since we observed the formation of a precipitate after starting the reaction, we made a filtration test by filtering off the formed precipitate and further irradiation of the clear solution, leading to excellent conversion of the triyne **4**, allowing us to conclude that the solid formed is not participating in the catalysis (Scheme 2c). For comparison, we also performed the reaction under thermal conditions at 150 °C (microwave), which did not lead to any conversion of triyne **4**.

This was also observed when performing first a photochemical reaction and then later continuing with thermal heating, where no further reaction was observed (Scheme 2d). Finally, to further elucidate the presence of excess CO for the reaction, we performed the reaction in a CO-pressurized flask and could not find any conversion of the triyne upon irradiation, demonstrating that a large excess of CO successfully inhibited the reaction (Scheme 2e).

For a direct comparison of the performance of the molecularly defined precatalyst **Mn7** with the catalyst system obtained from **Mn1** in combination with either dppm or PPh_3_, we applied triyne **21** as the testing substrate. This compound not only demonstrated excellent reactivity in substrate scope evaluations but also offered a straightforward, high-yield synthesis from triyne **4** while providing an additional nucleus for the NMR spectroscopic analysis of potential side products during the cyclization reactions. In these comparative experiments, cyclization product **41** was obtained with 91% yield when applying **Mn7**, and with 86 or 84% yield when using **Mn1** in combination with either the bidentate (dppm, **L6**) or monodentate (PPh_3_,**L1**) ligand *in situ*. Additional experimentation of the *in situ* catalyst system was conducted as we hypothesized further CO dissociation from **Mn1** under photochemical reaction conditions, and subsequent removal of the gaseous CO from the reaction mixture could potentially favor the deactivation of the catalyst by cluster formation. We tested the addition of an isonitrile as the ligand instead of phosphines with **Mn1** for eventual stabilization of the active catalyst. However, the application of 2,6-diisopropylphenyl isonitrile did not show the intended effect, as product **41** was obtained with only 17% yield, indicating deactivation of the catalyst by coordination of the isonitrile (compare compiled results in the Supporting Information).

The ^31^P NMR investigation of a solution of MnBr(CO)_3_(dppm) (**Mn7**) and a 1:1 mixture of **Mn1** and bidentate ligand dppp under photochemical conditions already provided clear proof of the different behavior of the species under irradiation. While in the case of **Mn7** after 10 min only the free dppm ligand signal was visible at −22 ppm, in the case of MnBr(CO)_3_(dppp) the complex was formed under photochemical conditions and remained stable (see SI, Figure SI-6 and SI-8). Also, the ^1^H NMR resonances for precatalyst **Mn7** became broader, suggesting that paramagnetic species may have been formed, while the spectrum of the formed complex MnBr(CO)_3_(dppp) remained unchanged over time (see SI, Figures SI-7 and SI-9). In the case of precatalyst **Mn7**, the resulting species were catalytically inactive when the triyne substrate was not present at the start of the irradiation but was added later, while the irradiation was continued. This suggests that the precatalyst in the absence of the cyclization substrate is decomposing to one or more new species with higher nuclearity.

**Scheme 3:**
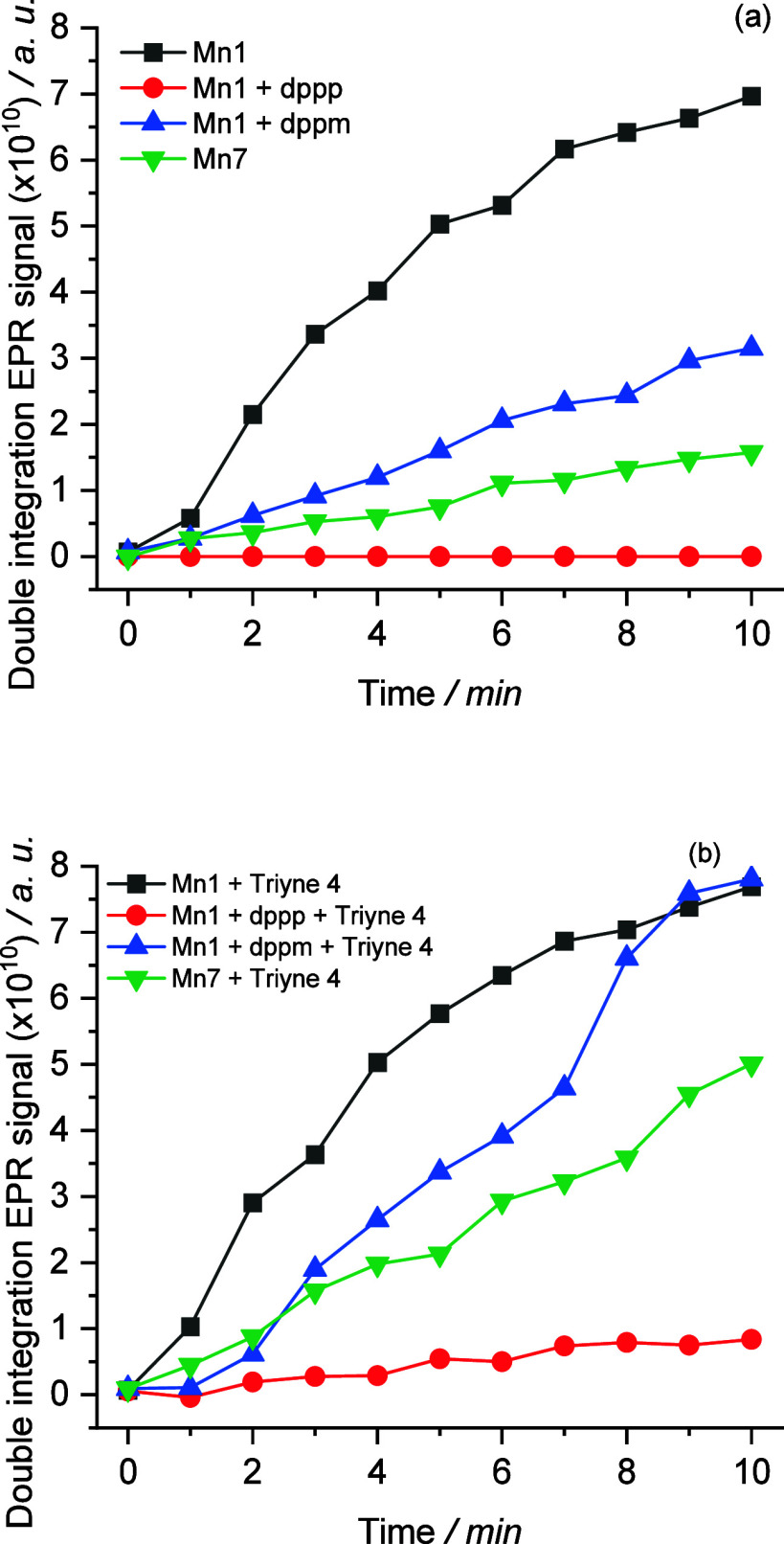
Double Integration of EPR Spectra of a Mixture of (a) **Mn1** (1 mM) and Different Ligands (1 mM); and (b) in Addition of Triyne **4** (10 mM)

Since the observed line broadening in the ^1^H NMR spectra suggested the formation of paramagnetic species upon irradiation, we conducted *in situ* EPR measurements of **Mn1** and **Mn7** and observed the evolution of a broad singlet signal over time (see SI, Figure SI-10). This signal is characteristic of Mn^II^ clusters. Most probably, these clusters are part of the catalytically inactive solid residue, the formation of which was observed during the catalytic tests (*vide supra*). Note that the intact **Mn1** and **Mn7** species in solution contain a Mn^I^ central atom, which is not EPR active. Thus, the formation of Mn^II^ clusters by *in situ* EPR points to the partial decomposition of the initial Mn complexes under reaction conditions. By monitoring the area (double integral) of the EPR signal as a function of time, it was found that the extent of Mn^II^ cluster formation depends on the presence of ligands (Scheme 3a). While the fastest cluster formation was observed for MnBr(CO)_5_ (**Mn1**), MnBr(CO)_3_(dppm) (**Mn7**) was much more stable under the same reaction conditions. Cluster formation could be significantly reduced and even totally suppressed by adding dppm or dppp, respectively, to the solution of **Mn1**. This points to the stabilizing effect of these ligands. Subsequent addition of the triyne substrate **4** accelerated the decomposition of **Mn7** and **Mn1** even in the presence of the dppm and dppp ligands (Scheme 3b). These results suggest that cluster formation from **Mn1** and **Mn7** might be initiated by the formation of vacant coordination sites upon release of CO (also supported by DFT calculations, *vide infra*) and depends on how fast these sites are filled by ligand or substrate molecules.

Interestingly, the partial decomposition of **Mn1** in combination with dppm as well as isolated precatalyst **Mn7** has only a negligible impact on the catalytic performance as almost the same high yields for the cyclization process were obtained with triyne **4** (Table 2, entry 6 and Chart 1). Obviously, this decomposition comprises only a minor part of the total Mn content in the solution, rendering sufficient intact Mn complex molecules available to drive the catalytic reaction.

The ATR-IR experiments revealed the degradation of **Mn7** and **Mn1** under irradiation conditions. Both the manganese complexes were decomposed to species with vibrational bands shifted toward lower wavenumbers (see SI, Figures SI-12 and SI-13). However, the decomposition of Mn in the presence of the dppm ligand was confirmed to be slower than without the ligand. In the presence of triyne **4**, the concentration profile of the cyclization product showed a steady increase over the entire investigated period, which is interestingly in correlation with the development of the free dppm ligand. Regarding the Mn complex, two species were determined during the irradiation. The first one is the precatalyst **Mn7**, which was gradually converted to a new manganese species. Compared to the degraded species from **Mn7**, this new species possesses an additional broad vibrational band in the region between 1970 and 2040 cm^–1^, which might be due to the coordination of the Mn center and triyne **4**.

Based on the literature precedent for other [2 + 2 + 2] cycloadditions^40^ and our own experience, we investigated the potential reaction mechanism by theoretical methods, and the details of computational methods, structures, energies, and stabilities of substrates and precatalysts as well as possible intermediates are given in the Supporting Information. While MnBr(CO)_5_ (**Mn1**) is catalyzing the cycloaddition reaction under irradiation, the addition of PPh_3_ or dppm as the ligand significantly improves the cyclization process, as our experiments have demonstrated. Other ligands specifically acted detrimental or even prevented any cyclization reaction. These studies are therefore directed toward a general understanding of the overall catalytic cycle and similarities and differences to reaction mechanisms of other transition metals as well as the specific understanding of the role of donor ligands in the precatalyst **Mn7** for the improvement of performance in the [2 + 2 + 2] cycloaddition.

**Scheme 4:**
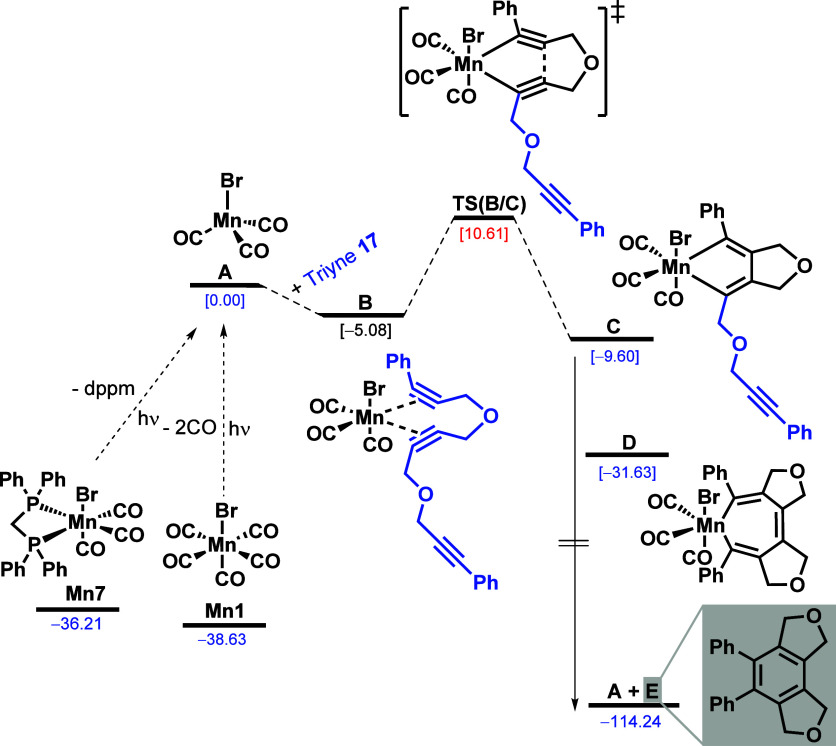
Presumed Reaction Mechanism for the Manganese-Catalyzed Cyclization of Triyne **17** from Theoretical Calculations

For the theoretical study, we first evaluated the possible first steps starting from MnBr(CO)_5_ (**Mn1**) and found CO ligand dissociation, resulting in the formation of MnBr(CO)_3_ (**A**) energetically more favorable over heterolytic and homolytic Br–Mn bond dissociation to form a Mn(CO)_5_ cation and a radical (Figure SI-14), respectively. Starting from MnBr(CO)_3_ (**A**), which has a tetragonal structure with 14 valence electrons in the singlet state, the cyclization sequences of triyne **17** are computed. Starting from both facial and meridional configuration of complex (**Mn7**), the most preferred route is dppm decoordination and the most favored intermediate is MnBr(CO)_3_ (**A**) (Figure SI-16). Therefore, we computed the reaction starting from MnBr(CO)_3_ (**A**) and triyne **17**. The proposed mechanism is shown in Scheme 4. The coordination of complex **A** and triyne **17** leading to intermediate **B** is exergonic by ∼5 kcal/mol, and subsequently overcoming an apparent activation barrier of nearly 15.69 kcal/mol led to the slightly exothermic formation of manganacyclopentadiene **C**, from which upon presumed insertion of the third triple bond the product **E** is formed in a highly exothermic reaction via **D**.

These results show that complex **A**, once formed, can react easily with triyne **17** to form the cyclization product via a rather small apparent barrier (10.61 kcal/mol). This agrees with the experimental condition of low temperature and short time. The most energy-demanding step is the decoordination of CO and dppm (38.63 and 36.21 kcal/mol, respectively), and such energy cost reveals why the reaction needs light irradiation and why it is not active only under thermal conditions at 150 °C. To check the influence of bulky substituents on the cyclization, the reaction of the triyne with 1-naphthyl substituents was computed (Chart 2, product **45**). However, it was found that the computed Gibbs free energy barrier and the reaction Gibbs free energy from complex **B** to complex **C** are close to those of the related compound **38** with phenyl substituent (16.04 vs 15.69 and −7.24 vs −4.52 kcal/mol). In addition, the cyclization energy from the substrate to product **45** is nearly the same as found for product **38** possessing phenyl groups (−110.66 vs −114.24 kcal/mol). All these data corroborate that the substitutes do not significantly affect the reaction kinetics and thermodynamics.

Since the CO ligand decoordinated from MnBr(CO)_5_ will escape from the system, the coordinatively unsaturated MnBr(CO)_3_ (**A**) will either form clusters or react with a different ligand, such as dppm. As found in our control experiments, the cyclization reaction with MnBr(CO)_5_ (**Mn1**) results in increased cluster formation and shows no cyclization when the reaction is conducted under a CO pressure with **Mn7**.

**Scheme 5:**
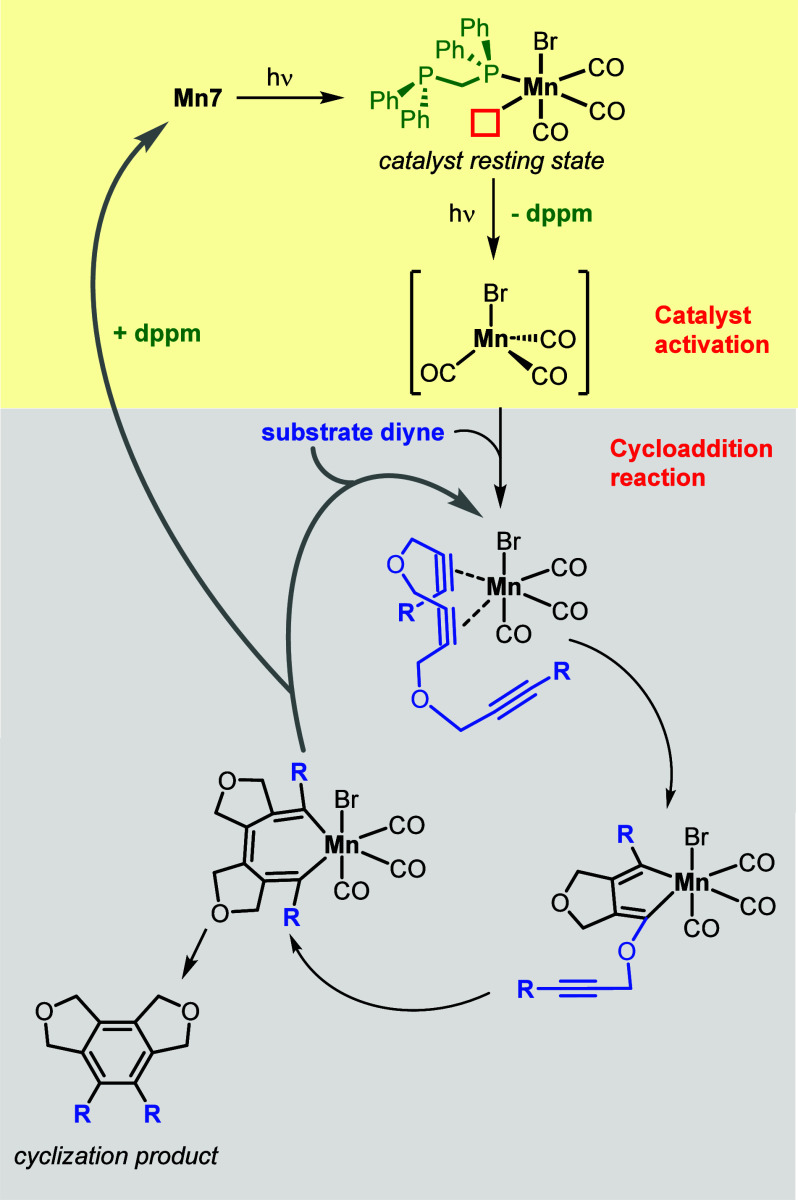
Tentative Catalytic Mechanism of the Manganese-Catalyzed [2 + 2 + 2] Cycloaddition Reaction

The catalytic cycle in Scheme 5 compiles the experimental and theoretical work toward the mechanism elucidation, exemplified for precatalyst **Mn7**. The experiments corroborated that the monodentate ligand PPh_3_ can provide equal coordinative support to the “MnBr(CO)_3_” fragment as a catalytically active species compared to dppm. The phosphine does not adopt a role as a steering ligand during the catalytic cycle but rather acts as a pure spectator ligand in the back row to provide support in preventing catalyst decomposition and aggregation to catalytically inactive species.^48^ The cycle represents a photochemically driven activation part, providing the active species and presumably also a thermally driven part for the cyclization to convert the triyne starting material, indicated by improved cyclization results for sterically larger substrates (compare Chart 2 with the results for the formation of triaryl **45** at different temperatures).

## Conclusions

In this investigation, we report on the first use of the manganese(I)–carbonyl complex MnBr(CO)_5_ as a catalyst for the [2 + 2 + 2] cycloaddition of functionalized triynes under mild photochemical conditions using irradiation at 450 nm wavelength to rapidly furnish the aromatic reaction products without requiring any other additional photo(redox)catalyst. The application of the small bite angle ligand 1,1-bis(diphenylphosphino)methane (dppm) to assemble the precatalyst MnBr(CO)_3_(dppm) significantly improves the performance and allows the isolation of high product yields within rather short reaction times. Reactions were usually performed at 30 °C, but depending on the substituents in the substrates, an increase of the reaction temperature to 80 °C leads to optimized yields even for larger substituents. The implementation of efficient synthetic pathways for the assembly of oligoalkynes from simple building blocks including the installation of a large variety of functional groups (e.g., substituted aryl, indenyl, Bpin, SiMe_3_, PPh_2_, pyridyl, thienyl) allows the assembly of highly substituted benzene derivatives including examples for further derivatization by cross-coupling reactions. The cyclizations can also be performed with hexaynes, leading to the corresponding biphenyl products in a 2-fold cyclization process. The successful performance was exemplarily demonstrated for cyanodiynes, leading to the substituted pyridine core. Application of the catalyst under stoichiometric reactions in the transformation between diynes and phosphaalkyne led to the first photomediated synthesis of phosphinines under rather mild reaction conditions. The reaction mechanism was investigated by spectroscopic and theoretical methods to elucidate the initiation of the catalytically active species and its entry into the catalytic cycle. The applied dppm and PPh_3_ ligands are only required for the fast generation and the stabilization of the catalytically active “MnBr(CO)_3_” fragment but are not involved in the catalytic cycle. Further computation confirmed the progression of the catalytic cycle via oxidative cyclization and insertion of the third alkyne moiety toward a manganacycloheptatriene intermediate and final ring closure via reductive elimination, yielding the aromatic product.

## Supporting Information Available

Detailed experimental information on the synthesis of starting materials and products, computational details, and spectral data for all synthesized compounds (PDF)
