# Supplementary material for: Photochemical Manganese-Catalyzed [2 + 2 + 2] Cycloaddition Reactions
Source: ACS Catal. 2025 Mar 24;15(7):5718–30. doi: 10.1021/acscatal.5c00349 (PMC11976702; doi:10.1021/acscatal.5c00349)
Supplement: Supplementary file 1 — cs5c00349_si_001.pdf [file cs5c00349_si_001.pdf]

Supporting Information

Photochemical Manganese-catalyzed [2+2+2] Cycloaddition Reactions

Benedikt N. Baumann[a], Phong Dam[b], Jabor Rabeah[b], Christoph Kubis[b], Angelika Brückner*[b], Haijun Jiao*[b] and Marko Hapke*[a,b]

[a] DI Benedikt N. Baumann, Prof. Dr. Marko Hapke
Institute of Catalysis (INCA), Johannes Kepler University Linz, Altenberger Strasse 69, 4040 Linz, Austria
E-mail: marko.hapke@jku.at
[b] MSc Phong Dam, Prof. Dr. Jabor Rabeah, Dr. Christoph Kubis, Prof. Dr. Angelika Brückner, Prof. Dr. Haijun Jiao, Prof. Dr. Marko Hapke
Leibniz Institute for Catalysis e.V. (LIKAT), Albert-Einstein-Strasse 29a, 18059 Rostock, Germany
E-mails: angelika.brueckner@catalysis.de; haijun.jiao@catalysis.de

Content

1 General Information 2
2 Experimental Procedures 4
2.1 Preparation of the precatalyst *fac*-MnBr(CO)$_3$(dppm) (**Mn7**) 4
2.2 Manganese-catalyzed [2+2+2] cycloaddition reactions 4
2.2.1 General procedure for the cyclization of triynes and hexaynes (GP1) 4
2.2.2 General procedure for the cyclization of diynes with phosphaalkynes (GP2) 17
2.3 General procedure for Suzuki-Miyaura cross-coupling reactions 19
2.4 Intermolecular and partially intermolecular cyclizations 21
2.5 Overview of screening reactions 22
2.5.1 Screening reactions with PRS1 (370 W) 22
2.5.2 Screening reactions with PRS2 (30 W) 24
2.5.3 Attempted cyclization under CO atmosphere 25
2.6 Attempted thermal cyclization with **Mn7** 25
2.7 Synthesis of new substrates and reported compounds 26
3 NMR Spectroscopic Data 41
3.1 NMR spectra of **Mn7** 41
3.2 NMR spectra of cyclotrimerization products 42
3.3 NMR spectra of new substrates and reported compounds 79
4 Mechanistic Investigations 111
4.1 NMR spectroscopic investigations 111
4.2 Other spectroscopic data 115
5 Computational Calculation Data 117
6 References 145

1 General Information

Reactions with Schlenk equipment were performed under inert atmosphere in flame dried glassware using standard Schlenk technique with argon (purity 5.0 purchased from Linde Gas GmbH) as inert gas or in an argon filled LABmaster PRO glove-box from M. Braun (purity 6.0 purchased from Linde Gas GmbH). Dry solvents were either obtained from a SPS 7 solvent purification system from M. Braun (acetonitrile, dichloromethane (DCM), Et_2O, *n*-hexane, tetrahydrofurane (THF), toluene) or from commercial sources delivered in bottles sealed with a septum and used without further purification. The use of celite refers to diatomaceous earth traded as Celite 545®. If necessary, solvents were degassed by freeze-pump-thaw cycles. $MnBr(CO)_5$ was purified by sublimation prior to use and analyzed by ^{55}Mn NMR as well as IR spectroscopy. Adamantylphosphaalkyne was purchased from VeZerv Laborsynthesen. An overview of compounds synthesized according to published literature procedures is provided in Table S1. All other compounds were commercially available.

NMR spectroscopy was performed on a Bruker Avance 300 MHz spectrometer (^{1}H: 300 MHz, ^{13}C: 75 MHz, ^{11}B NMR: 96 MHz ^{19}F: 282 MHz. ^{31}P: 122 MHz), or a Bruker Avance III 500 MHz spectrometer (^{1}H: 500 MHz, ^{13}C: 126 MHz, ^{19}F: 471 MHz, ^{31}P: 203 MHz, ^{55}Mn: 124 MHz) at 298 K. Chemical shifts are stated in parts per million (ppm) on the delta scale (δ). Axis calibration was performed using residual protic solvent signals ($CDCl_3$: 7.26 ppm, CD_2Cl_2: 5.32 ppm d_3-MeCN: 1.94 ppm, DMSO-d_6: 2.50 for ^{1}H NMR; $CDCl_3$: 77.16 ppm, CD_2Cl_2: 53.84 ppm, d_3-MeCN: 1.32 ppm for ^{13}C NMR). Multiplicities are stated as s (singlet), d (doublet), dd (doublet of doublets), t (triplet) or m (multiplet).

EPR spectra were recorded on a Bruker EMX CW-micro X-band spectrometer with a microwave power of 6.9 mW, a modulation frequency of 100 kHz and a modulation amplitude of up to 5 G. The EPR spectrometer is equipped with a variable temperature control unit including a liquid N_2 cryostat and a temperature controller for recording the EPR spectra at low temperature down to 100 K. g values were calculated using the equation $h\nu = g\beta B_0$ with β, B_0 and ν being the Bohr magneton, resonance field and frequency, respectively. Calibration of the g values was performed using a DPPH standard (g = 2.0036 ± 0.0004). A side of cavity towards the light source was split into grids for the irradiation coming through. The irradiation source for EPR measurements was by a Xenon lamp with a 420 nm filter. Before the measurement, a reaction mixture filled in a normal EPR quartz-tube and covered by a rubber septum was prepared in a glovebox. The spectra were recorded in function of time with the domain of 1 minute.

ATR-IR spectroscopy was performed using a ReactIR 15 spectrometer equipped with a fibre-optical silicon probe (Mettler-Toledo) with 8 reflexions. The ATR probe was inserted from the top of the Schlenk tube, from which was connected to a Schlenk line to evacuate/purge with argon five times before each experiment. All the spectra were automatically recorded between 3000 - 650 cm^{-1} at 30-second intervals. Data analysis of the IR-spectroscopic data was conducted with the help of the software tool peak group analysis (PGA)[1] being part of the FACPACK[2] package and using the program Matlab 2019b.

High-resolution mass spectrometry was carried out on an Agilent QTOF 6520 with electrospray ionization (ESI) (mass error: ±5 ppm).

Flash column chromatography was performed on silica gel obtained from Macherey-Nagel (particle size: 0.04-0.063 mm) with an Interchim puriFlash XS420 chromatograph equipped with an UV-detector.

Photochemical reaction setup 1 (PRS1): Reactions were conducted in a HepatoChem Lucent 360 photoreactor equipped with LEDs emitting at 450 nm (λ_{max}). The irradiation setup consists of four side lights and one bottom light with a total power consumption of 370 W. The reaction chamber is tempered by water which is continuously pumped over a heat exchanger connected to an external cryostat for temperature control. Reactions were performed in 4 mL screw cap vials or 20 mL scintillation vials and additionally sealed by duct tape.

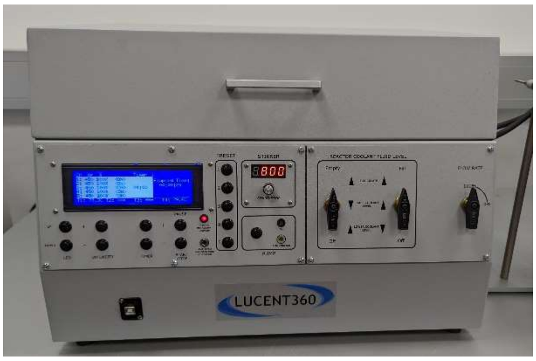

Figure SI-1: Hepatochem Lucent360 photoreactor.

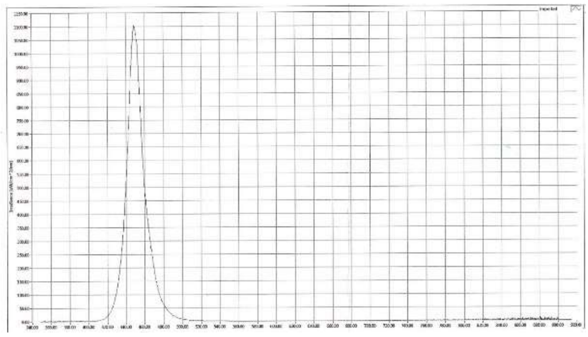

Figure SI-2: Emission spectra of the applied light source.

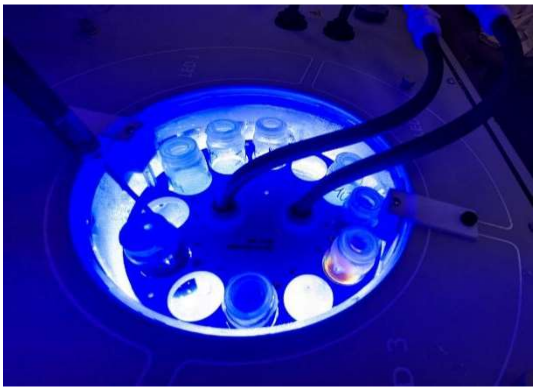

Figure SI-3: Inlet for reactions in 20 mL scintillation vials.

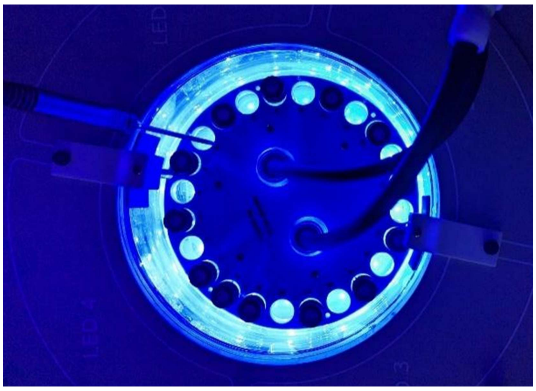

Figure SI-4: Inlet for reactions in 4 mL screw cap vials.

<u>Photochemical reaction setup 2 (PRS2):</u> Reactions were conducted in an air-cooled EvoluChem™ PhotoRedOx Box equipped with a 30 W LED emitting at 450 nm (λ_{max}) in 4 mL screw cap vials or 20 mL scintillation vials and additionally sealed by duct tape.

2 Experimental Procedures

2.1 Preparation of the precatalyst *fac*-MnBr(CO)$_3$(dppm) (Mn7)[3]

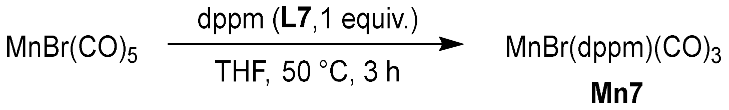

CAUTION: CO is a highly toxic gas! It is crucial to ensure proper ventilation and utilize appropriate safety measures!

The synthesis of precatalyst **Mn7** was adopted from the literature. *To minimize light exposure, the Schlenk tube was covered in aluminum foil during the reaction.* In a 50 mL Schlenk tube connected to a bubble counter, MnBr(CO)$_5$ (**Mn1**, 824.7 mg, 3.0 mmol) and dppm (**L6**, 384.4 mg, 3.0 mmol) were stirred in 20 mL dry and degassed THF at 50 °C for 3 h. The progress of the reaction was evaluated by IR spectroscopy. The volume of THF was reduced to approximately 5 mL *in vacuo* at 50 °C. At room temperature, 20 mL of *n*-pentane were added, resulting in the formation of a yellow precipitate. The supernatant was removed with a syringe. This purification process was repeated three times. The yellow precipitate was further purified *in vacuo* at 50 °C to obtain **Mn7** as a yellow powder (1.683 g, 2.79 mmol, 93%).

^{1}H NMR (300MHz, CDCl$_3$): δ = 7.60-7.70 (m, 4H), 7.44-7.54 (m, 4H), 7.29-7.42 (m, 12H), 4.74-4.90 (m, 1H), 4.59-4.73 (m, 1H).
^{13}C{^{1}H} NMR (125 MHz, CDCl$_3$): δ = 222.1, 218.8, 133.8 (t, *J* = 18.0 Hz,), 132.4 (t, *J* = 23.3 Hz, overlapping), 132.2 (t, *J* = 4.5 Hz, overlapping), 131.7 (t, *J* = 4.6 Hz) 130.7, 130.6, 129.0 (t, *J* = 4.8 Hz), 128.8 (t, *J* = 4.8 Hz), 40.1 (t, *J* = 19.9 Hz).
^{31}P{^{1}H} NMR (202 MHz, CDCl$_3$): δ = 12.5.
^{55}Mn NMR (124 MHz, CDCl$_3$): δ = -967.35.

2.2 Manganese-catalyzed [2+2+2] cycloaddition reactions

2.2.1 General procedure for the cyclization of triynes and hexaynes (GP1)

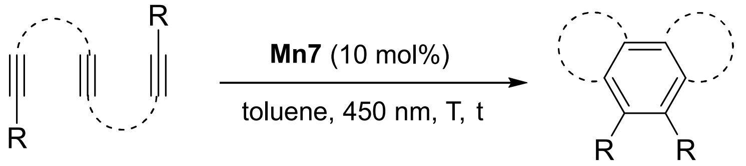

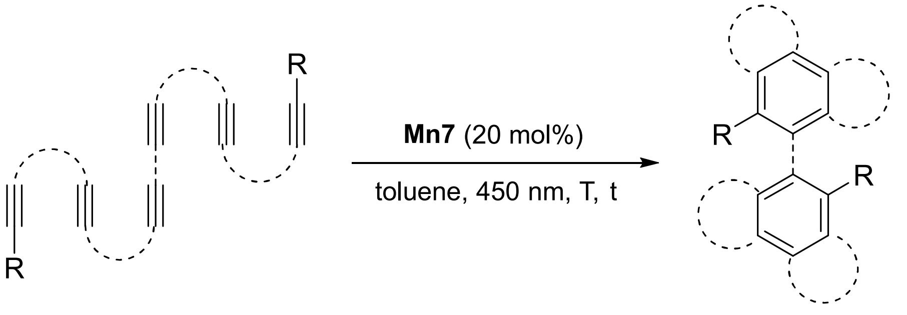

In the glove-box, the respective triyne or hexayne (0.400 mmol), **Mn7** (24 mg, 0.040 mmol, 10 mol% or 48 mg, 0.080 mmol, 20 mol%) and 20 mL dry and degassed toluene were combined in a 20 mL scintillation vial. The vial was additionally sealed with duct tape and stirred in PRS1 without irradiation until all components were dissolved. The homogeneous reaction mixture was then irradiated in PRS1 while stirring at 30 °C or 80 °C for 1 hour. The reaction mixture was charged on silica and the product isolated by flash column chromatography followed by removal of volatiles *in vacuo*.

Compound 5:[4]

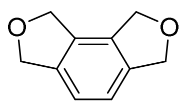

The cyclization reaction was conducted according to GP1 with compound **4** (65 mg, 0.400 mmol) and 10 mol% of **Mn7** (reaction conditions: 30 °C for 1 h reaction time).
Yield: 94% (61 mg, 0.376 mmol)
Eluent composition for chromatography: n-heptane/DCM (v/v 3:1).

^{1}H NMR (300 MHz, $CDCl_3$): δ = 7.15 (s, 2H), 5.13 (s, 4H), 5.03 (s, 4H).
^{13}C{^{1}H} NMR (75 MHz, $CDCl_3$): δ = 138.60, 132.21, 119.25, 73.42, 72.23.
HRMS(ESI) calculated for $[M+H]^+$: 163.0754; found: 163.0751.

Compound 11:[5]

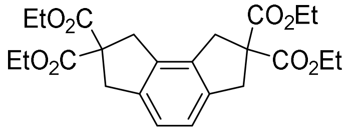

The cyclization reaction was conducted according to GP1 with compound **6** (177 mg, 0.400 mmol) and 10 mol% of **Mn7** (reaction conditions: 80 °C for 1 h reaction time).
Yield: 19% (34 mg, 0.076 mmol)
Eluent composition: gradient elution from *n*-heptane/DCM (v/v 1:1) to pure DCM.

^{1}H NMR (300 MHz, $CDCl_3$): δ = 7.01 (s, 2H), 4.24 (q, *J* = 7.1 Hz, 8H), 3.56 (s, 4H), 3.51 (s, 4H), 1.26 (q, *J* = 7.1 Hz, 12H).
^{13}C{^{1}H} NMR (75 MHz, $CDCl_3$): δ = 171.70, 139.03, 135.80, 122.8, 61.83, 60.61, 40.40, 39.02, 14.06.
HRMS(ESI) calculated for $[M+H]^+$: 447.2013; found: 447.2015.

Compound 12:[4]

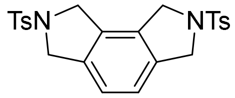

The cyclization reaction was conducted according to GP1 with compound **7** (187 mg, 0.400 mmol) and 10 mol% of **Mn7** (reaction conditions: 80 °C for 1 h reaction time).
Yield: 56% (105 mg, 0.224 mmol)
Eluent composition: gradient elution from *n*-heptane/DCM (v/v 1:1) to pure DCM.

^{1}H NMR (300 MHz, $CDCl_3$): δ = 7.77-7.72 (d, *J* = 7.8 Hz, 4H), 7.33-7.29 (d, *J* = 7.7 Hz, 4H), 7.04 (s, 2H), 4.57 (s, 4H), 4.45 (s, 4H), 2.40 (s, 6H).
^{13}C{^{1}H} NMR (75 MHz, $CDCl_3$): δ = 144.02, 136.24, 133.68, 130.99, 130.04, 127.70, 122.21, 53.59, 52.31, 21.65.
HRMS(ESI) calculated for $[M+NH_4]^+$: 486.1516; found: 489.1516.

Compound 13:[6]

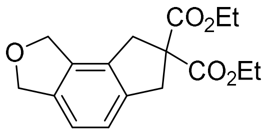

The cyclization reaction was conducted according to GP1 with compound **8** (122 mg, 0.400 mmol) and 10 mol% of **Mn7** (reaction conditions: 80 °C for 1 h reaction time).
Yield: 23% (28 mg, 0.092 mmol)
Eluent composition: gradient elution from *n*-heptane/DCM (v/v 1:1) to pure DCM.

^{1}H NMR (300 MHz, $CDCl_3$): δ = 7.12 (d, *J* = 6.5 Hz, 1H), 7.04 (d, *J* = 6.5 Hz, 1H), 5.09 (s, 2H), 5.05 (s, 2H), 4.22 (q, *J* = 7.1 Hz, 4H), 3.61 (s, 2H), 3.48 (s, 2H), 1.27 (t, *J* = 7.1 Hz, 6H).
^{13}C{^{1}H} NMR (75 MHz, $CDCl_3$): δ = 171.61, 138.59, 138.22, 135.22, 135.01, 133.20, 123.24, 119.59, 73.76, 72.52, 61.91, 60.73, 40.23, 38.89, 14.12.
HRMS(ESI) calculated for $[M+H]^+$: 305.1384; found: 305.1388.

Compound 14:[7]

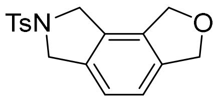

The cyclization reaction was conducted according to GP1 with compound **9** (126 mg, 0.400 mmol) and 10 mol% of **Mn7** (reaction conditions: 80 °C for 1 h reaction time).
Yield: 78% (98 mg, 0.311 mmol)
Eluent composition: *n*-heptane/DCM (v/v 2:1).

^{1}H NMR (300 MHz, $CDCl_3$): δ = 7.77 (d, *J* = 7.7 Hz, 2H), 7.32 (d, *J* = 7.7 Hz, 2H), 7.09 (m, 2H), 5.02 (s, 4H), 4.57 (s, 4H), 2.41 (s, 6H).
^{13}C{^{1}H} NMR (75 MHz, $CDCl_3$): δ = 144.10, 139.51, 135.90, 134.06, 133.95, 130.20, 129.77, 127.90, 121,91, 120.73, 73.76, 72.36, 53.76, 52.67, 21.83.
HRMS(ESI) calculated for $[M+H]^+$: 399.1720; found: 399.1719.

Compound 15:

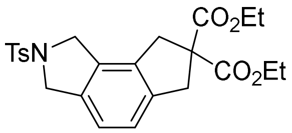

The cyclization reaction was conducted according to GP1 with compound **10** (183 mg, 0.400 mmol) and 10 mol% of **Mn7** (reaction conditions: 80 °C for 1 h reaction time).
Yield: 38% (70 mg, 0.153 mmol)
Eluent composition: gradient elution from *n*-heptane/DCM (v/v 1:1) to pure DCM.

^{1}H NMR (300 MHz, $CDCl_3$): δ = 7.78-7.74 (d, (d, *J* = 8.1 Hz, 2H), 7.33-7.30 (d (d, *J* = 7.9 Hz, 2H), 7.08 (d, *J* = 7.83 Hz, 1H), 6.96 (d, *J* = 7.83 Hz, 1H) 4.58 (s, 2H), 4.52 (s, 2H), 4.19 (q, *J* = 7.10 Hz, 4H), 3.54 (s, 2H), 3.42 (s, 2H), 2.40 (s, 3H), 1.24 (t, *J* = 7.10 Hz, 6H).
^{13}C{^{1}H} NMR (75 MHz, $CDCl_3$): δ = 171.45, 143.80, 140.10, 135.10, 134.63, 133.83, 132.10, 129.96, 127.69, 123.76, 121.29, 61.98, 60.55, 53.80, 52.64, 40.29, 38.73, 21.61, 14.11.
HRMS(ESI) calculated for $[M+H]^+$: 458.1632; found: 458.1631.

Compound 37:[6]

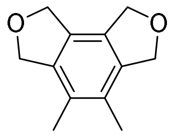

The cyclization reaction was conducted according to GP1 with compound **16** (76 mg, 0.400 mmol) and 10 mol% of **Mn7** (reaction conditions: 30 °C for 1 h reaction time).
Yield: 93% (71 mg, 0.373 mmol)
Eluent composition: *n*-heptane/DCM (v/v 1:1).

^{1}H NMR (300 MHz, $CDCl_3$): δ = 5.09 (s, 4H), 5.04 (s, 4H), 2.15 (s, 6H).
^{13}C{^{1}H} NMR (75 MHz, $CDCl_3$): δ = 138.22, 128.94, 128.71, 73.40, 73.02, 15.60.
HRMS(ESI) calculated for $[M+H]^+$: 191.1067; found: 191.1063.

Compound 38:[4]

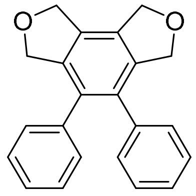

The cyclization reaction was conducted according to GP1 with compound **17** (126 mg, 0.400 mmol) and 10 mol% of **Mn7** (reaction conditions: 30 °C for 1 h reaction time).
Yield: 96% (121 mg, 0.385 mmol)
Eluent composition: *n*-heptane/DCM (v/v 1:1).

^{1}H NMR (300 MHz, $CDCl_3$): δ = 7.22-7.13 (m, 6H), 7.06-7.00 (m, 4H), 5.18 (s, 4H), 5.00 (s, 4H).
^{13}C{^{1}H} NMR (75 MHz, $CDCl_3$): δ = 138.92, 138.64, 134.32, 131.44, 129.60, 128.12, 127.00, 73.82, 72.93.
HRMS(ESI) calculated for $[M+NH_4]^+$: 315.1645 found: 315.1650.

Compound 39:[8]

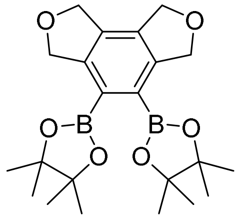

The cyclization reaction was conducted according to GP1 with compound **18** (165 mg, 0.400 mmol) and 10 mol% of **Mn7** (reaction conditions: 30 °C for 1 h reaction time).
Yield: 88% (145 mg, 0.350 mmol)
Eluent composition: gradient elution from *n*-heptane/DCM (v/v 1:1) to pure DCM.

^{1}H NMR (300 MHz, $CDCl_3$): δ = 5.19 (s, 4H), 5.00 (s, 4H), 1.35 (s, 24H).
^{11}B NMR (96 MHz, $CDCl_3$): δ = 30.97.
^{13}C{^{1}H} NMR (75 MHz, $CDCl_3$): δ = 144.44, 133.26, 84.18, 74.58, 72.18, 25.11. *The borylated carbon was not detectable.*
HRMS(ESI) calculated for $[M+H]^+$: 415.2466; found: 415.2466.

Compound 40:

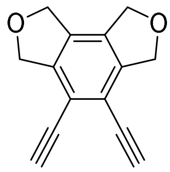

The cyclization reaction was conducted according to GP1, with compound **19** (84 mg, 0.400 mmol) and 10 mol% of **Mn7** (reaction conditions: 30 °C for 1 h reaction time).
Yield: 72% (61 mg, 0.290 mmol)
Eluent composition: DCM.

^{1}H NMR (500 MHz, $CDCl_3$): δ = 5.15 (s, 4H), 5.05 (s, 4H), 3.48 (s, 2H).
^{13}C{^{1}H} NMR (126 MHz, $CDCl_3$): δ = 143.13, 133.02, 117.44, 84.84, 79.00, 73.80, 72.98.
HRMS(ESI) calculated for $[M+NH_4]^+$: 228.1019; found: 228.1011.

Compound 41:[9]

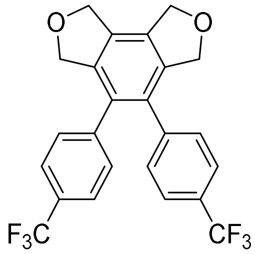

The cyclization reaction was conducted according to GP1 with compound **20** (180 mg, 0.400 mmol) and 10 mol% of **Mn7** (reaction conditions: 30 °C for 1 h reaction time).
Yield: 97% (175 mg, 0.389 mmol)
Eluent composition: pure *n*-heptane to pure DCM (gradient elution).

^{1}H NMR (500 MHz, $CDCl_3$): δ = 7.53-7.43 (m, 4H), 7.19-7.09 (m, 4H), 5.16 (s, 4H), 4.97 (s, 4H).
^{13}C{^{1}H} NMR (125 MHz, $CDCl_3$): δ = 141.9, 139.2, 132.8, 132.6, 129.9, 129.6 (q, *J* = 32.8 Hz), 125.4 (q, *J* = 3.6 Hz), 124.1 (q, *J* = 272.2 Hz), 73.6, 72.9.
^{19}F{^{1}H} (471 MHz, $CDCl_3$): δ = -62.67.
HRMS(ESI) calculated for $[M+H]^-$: 449.0982; found: 449.0986.

Compound 42:

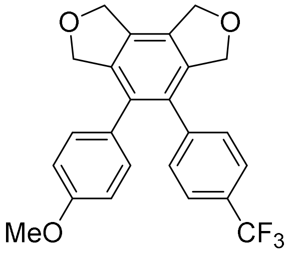

The cyclization reaction was conducted according to GP1 with compound **21** (165 mg, 0.400 mmol) and 10 mol% of **Mn7** (reaction conditions: 30 °C for 1 h reaction time).
Yield: 56% (93 mg, 0.226 mmol)
Eluent composition: pure *n*-heptane to pure DCM (gradient elution).

^{1}H NMR (500 MHz, $CDCl_3$): δ = 7.47 (d, *J* = 8.1 Hz, 2H), 7.15 (d, *J* = 8.1 Hz, 2H) 6.94-6.89 (m, 2H), 6.75-6.72 (m, 2H), 5.16-5.12 (m, 4H), 5.00 (s, 4H), 4.96 (s, 4H), 3.76 (s, 3H).

$^{13}C\{^1H\}$ NMR (126 MHz, $CDCl_3$): δ = 158.69, 142.72, 139.40, 138.82, 134.00, 132.93, 132.19, 131.52, 130.62, 130.19, 129.98, 129.08 (q, *J* = 32.3 Hz), 125.20 (q, *J* = 3.7 Hz), 124.2 (q, *J* = 272.5 Hz), 113.82, 73.84, 73.64, 72.93, 72.88, 55.26.
$^{19}F\{^1H\}$ (471 MHz, $CDCl_3$): δ = -62.51.
HRMS(ESI) calculated for $[M+H]^+$: 413.1359; found: 413.1353.

Compound 43:[8]

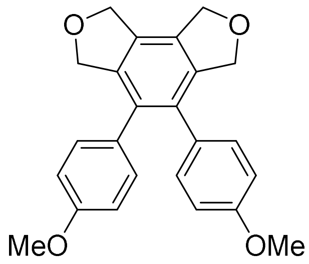

The cyclization reaction was conducted according to GP1 with compound **22** (150 mg, 0.400 mmol) and 10 mol% of **Mn7** (reaction conditions: 30 °C for 1 h reaction time).
Yield: 47% (70 mg, 0.188 mmol)
Eluent composition: DCM.

^{1}H NMR (500 MHz, $CDCl_3$): δ = 6.98-6.90 (m, 2H), 6.68-6.70 (m, 2H) 5.13 (s, 4H), 4.99 (s, 4H), 3.75 (s, 4H).
^{13}C NMR (126 MHz, $CDCl_3$): δ = 158.31, 139.00, 130.02, 131.09, 130.95, 130.62, 113.5, 73.84, 72.80, 55.12.
HRMS(ESI) calculated for $[M+H]^+$: 375.1592; found: 375.1591.

Compound 44:

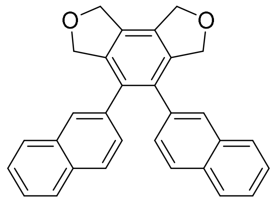

The cyclization reaction was conducted according to GP1 with compound **23** (166 mg, 0.400 mmol) and 10 mol% of **Mn7** (reaction conditions: 30 °C for 1 h reaction time).
Yield: 95% (156 mg, 0.376 mmol)
Eluent composition: pure *n*-heptane to pure DCM (gradient elution).

^{1}H NMR (300 MHz, $CDCl_3$): δ = 7.74-7.55 (m, 8H), 7.44-7.40 (m, 4H), 7.16-7.15 (m, 2H), 5.21 (s, 4H), 5.08 (s, 4H).
^{13}C NMR (126 MHz, $CDCl_3$): δ = 139.39, 136.23, 134.29, 133.14, 132.21, 131.70, 128.36, 127.97, 127.80, 127.76, 127.71, 126.17, 126.10, 73.91, 72.95.
HRMS(ESI) calculated for $[M+NH_4]^+$: 432.1958; found: 432.1956.

Compound 45:[20]

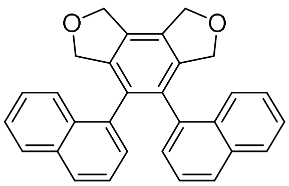

The cyclization reaction was conducted according to GP1 with compound **24** (166 mg, 0.400 mmol) and 10 mol% of **Mn7** (reaction conditions: 30 °C or 80 °C for 1 h reaction time).
Yield (30 °C): 41% (68 mg, 0.164 mmol)
Yield (80 °C): 91% (151 mg, 363 mmol)
Eluent composition: pure *n*-heptane to pure DCM (gradient elution).

^{1}H NMR (500 MHz, CD_3CN): δ = 7.80-7.00 (m, 14H), 5.21-5.18 (m, 4H), 4.66-4.49 (m, 2H), 4.59-4.40 (m, 2H).
$^{13}C\{^1H\}$ NMR (125 MHz, $CDCl_3$): δ = 140.1, 140.0, 1, 136.0, 135.6, 133.6, 133.5, 133.40, 133.36, 131.7, 131.6, 131.4, 131.0, 128.5, 128.16, 128.14, 127.74, 128.71, 126.4, 126.1, 125.9, 125.8, 125.7, 125.66, 125.63, 125.1, 124.7, 73.9, 73.8, 73.1, 73.0.
HRMS(ESI) calculated for $[M+H]^+$: 415.1693; found: 415.1699.

Compound 46:

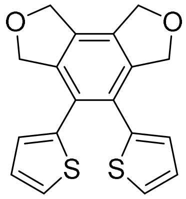

The cyclization reaction was conducted according to GP1 with compound **25** (131 mg, 0.400 mmol) and 10 mol% of **Mn7** (reaction conditions: 30 °C for 1 h reaction time).
Yield: 89% (117 mg, 0.356 mmol)
Eluent composition: pure *n*-heptane to pure DCM (gradient elution).

^{1}H NMR (300 MHz, $CDCl_3$): δ = 7.30-7.23 (m, 2H), 6.99-6.91 (m, 2H), 6.86-6.78 (m, 2H), 5.16-5.06 (m, 8H).
$^{13}C\{^1H\}$ NMR (75 MHz, $CDCl_3$): δ = 140.17, 139.11, 132.39, 127.98, 127.76, 126.90, 126.57, 74.15, 72.98.
HRMS(ESI) calculated for $[M+H]^+$: 327.0508; found: 327.0511.

Compound 47:

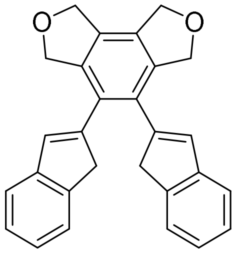

The cyclization reaction was conducted according to GP1 with compound **26** (156 mg, 0.400 mmol) and 10 mol% of **Mn7** (reaction conditions: 30 °C for 1 h reaction time).
Yield: 92% (144 mg, 0.379 mmol)
Eluent composition: *n*-heptane/DCM (v/v 1:1).

^{1}H NMR (300 MHz, CD_2Cl_2): δ = 7.41-7.23 (m, 8H), 6.88-6.87 (m, 2H), 5.15 (s, 4H), 5.11 (s, 4H), 3.46-3.45 (m, 4H).
$^{13}C\{^{1}H\}$ NMR (75 MHz, CD_2Cl_2): δ = 146.08, 144.98, 143.91, 139.29, 132.14, 131.97, 130.33, 126.84, 125.16, 123.96, 121.42, 74.24, 73.05, 42.22.
HRMS(ESI) calculated for $[M+H]^+$: 391.1693; found: 391.1694.

Compound 48:

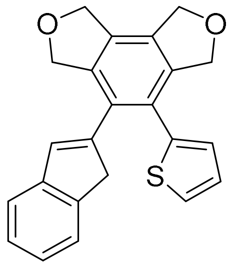

The cyclization reaction was conducted according to GP1 with compound **27** (143 mg, 0.400 mmol) and 10 mol% of **Mn7** (reaction conditions: 30 °C for 1 h reaction time).
Yield: 92% (128 mg, 0.356 mmol)
Eluent composition: pure *n*-heptane to pure DCM (gradient elution).

^{1}H NMR (500 MHz, $CDCl_3$): δ = 7.42-7.39 (m, 1H), 7.36-7.27 (m, 4H), 7.19-7.15 (m, 1H), 6.85 (s, 1H), 5.17-5.11 (m, 8H), 3.18 (s, 2H).
^{13}C NMR (126 MHz, $CDCl_3$): δ = 145.29, 144.46, 143.80, 139.85, 139.10, 132.25, 131. 71, 130.10, 127.71, 127.49, 127.19, 126.84, 126.70, 126.54, 126.50, 124.90, 123.58, 121.14, 74.15, 74.03, 72.91, 72.89, 41.34.
HRMS(ESI) calculated for $[M+Na]^+$: 381.0920; found: 381.0920.

Compound 49:

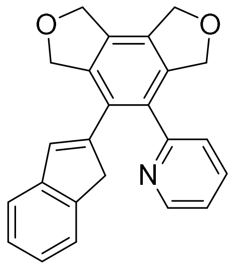

The cyclization reaction was conducted according to GP1 with compound **28** (141 mg, 0.400 mmol) and 10 mol% of **Mn7** (reaction conditions: 30 °C for 1 h reaction time).
Yield: 92% (52.3 mg, 0.148 mmol)
Eluent composition: pure *n*-heptane to pure acetone (gradient elution).

^{1}H NMR (300 MHz, $CDCl_3$): δ = 8.68-8.62 (m, 1H), 7.56-7.51 (m, 1H), 7.38-7.32 (m, 1H), 7.30-7.10 (m, 8H), 6.76 (s, 1H), 5.26-5.02 (m, 8H), 3.07 (s, 2H).
$^{13}C\{^{1}H\}$ NMR (75 MHz, $CDCl_3$): δ = 157.99, 19.63, 145.02, 144.49, 143.56, 139.81, 138.88, 136.41, 132.90, 132.53, 132.14, 129.90, 126.56, 124.91, 124.67, 123.56, 122.23, 121.17, 74.19, 73.69, 72.91, 72.47, 41.90.
HRMS(ESI) calculated for $[M+H]^+$: 354.1489, found: 354.1489.

Compound 50:

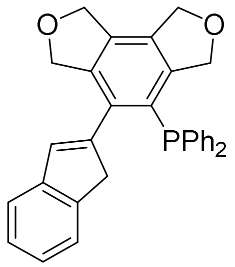

The cyclization reaction was conducted according to GP1 with compound **29** (184 mg,f 0.400 mmol) and 10 mol% of **Mn7** (reaction conditions: 30 °C for 1 h reaction time).
Yield: 92% (81 mg, 0.176 mmol)
Eluent composition: pure *n*-heptane to pure DCM (gradient elution).

^{1}H NMR (300 MHz, $CDCl_3$): δ = 7.45-7.12 (m, 14H), 6.49 (s, 2H), 5.10 (s, 2H), 5.02 (s, 2H), 4.91 (s, 2H), 4.08 (s, 2H), 3.67(s, 2H).
^{13}C NMR (126 MHz, $CDCl_3$): δ = 145.87, (d, *J* = 9.4 Hz), 145.23 (d, *J* = 2.3 Hz), 144.64, 143.63, 138.97 (d, *J* = 16.5 Hz), 138.76 (d, *J* = 13.8 Hz), 135.85 (d, *J* = 12.9 Hz), 134.1, 132.56 (d, *J* = 19.4 Hz), 128.65 (d, *J* = 30.3 Hz), 126.56, 124.95, 123.70, 121.29, 73.85 (d, *J* = 3.3 Hz), 73.49 (d, *J* = 1.6 Hz), 73.01, 71.40, 43.49, 43.42.
^{31}P{^{1}H} NMR (121 MHz, $CDCl_3$): δ = 13.65.
HRMS(ESI) calculated for $[M+H]^+$: 461.1665; found: 461.1668.

Compound 51:[10]

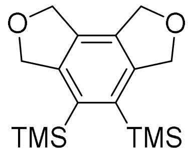

The cyclization reaction was conducted according to GP1 with compound **30** (122 mg, 0.400 mmol) and 10 mol% of **Mn7** (reaction conditions: 30 °C for 1 h reaction time).
Yield: 79% (97 mg, 0.316 mmol)
Eluent composition: pure *n*-heptane to pure DCM (gradient elution).

^{1}H NMR (300 MHz, $CDCl_3$): δ = 5.16 (s, 4H), 4.95 (s, 4H), 0.40 (s, 18H).
^{13}C{^{1}H} NMR (75 MHz, $CDCl_3$): δ = 15,75, 139.07, 132.53, 75.03, 71.38, 3.77.
^{29}Si{^{1}H} NMR (60 MHz, $CDCl_3$): -3.90
HRMS(ESI) calculated for $[M+H]^+$: 307.1544; found: 307.1548.

Compound 52:

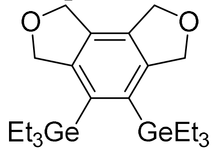

The cyclization reaction was conducted according to GP1 with compound **31** (122 mg, 0.400 mmol) and 10 mol% of **Mn7** (reaction conditions: 30 °C for 1 h reaction time).
Yield: 87% (173 mg, 0.348 mmol)
Eluent composition: pure *n*-heptane to pure DCM (gradient elution).

^{1}H NMR (300 MHz, $CDCl_3$): δ = 5.13 (s, 4H), 4.97 (s, 4H), 1.17-0.94(m, 30H).
^{13}C NMR (75 MHz, $CDCl_3$): δ = 145.52, 139.0, 131.48, 75.27, 71.76, 9.46, 7.86.
HRMS(ESI) calculated for $[M+NH_4]^+$: 481.1386; found: 481.1388.

Compound 53:

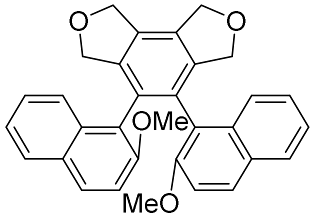

The cyclization reaction was conducted according to GP1 with compound **32** (190, 0.400 mmol) and 10 mol% of **Mn7** (reaction conditions: 80 °C for 1 h reaction time).
Yield: 23% (36 mg, 0.076 mmol)
Eluent composition: pure *n*-heptane to *n*-heptane/acetone (v/v 1:1) (gradient elution).

^{1}H NMR (300 MHz, $CDCl_3$): δ = 7.55-7.41 (m, 6H), 7.10-6.91 (m, 6H), 5.30-5.19 (m, 4H), 4.89-4.72 (m, 4H), 3.75 (s, 6H).
^{13}C NMR (75 MHz, $CDCl_3$): δ = 153.83, 153.33, 140.87, 140.54, 132. 63, 132.01, 131.64, 131.32, 130.65, 3, 129.37, 129.15, 128.53, 128.37, 127.78, 127.62, 125.90, 125.83, 125.74, 125.33, 123.14, 123.11, 120.55, 120.48, 111.96, 111.84, 74.26, 74.10, 73.25, 73.19, 56.00, 54.93.
HRMS(ESI) calculated for $[M+NH_4]^+$: 492.2169; found: 492.2168.

Compound 54:[11]

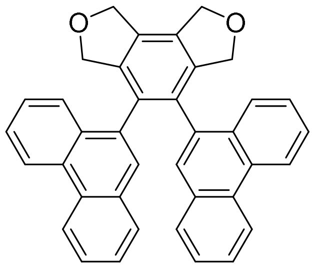

The cyclization reaction was conducted according to GP1 with compound **33** (206 mg, 0.400 mmol) and 10 mol% of **Mn7** (reaction conditions: 80 °C for 1 h reaction time).
Yield: 84% (173 mg, 0.336 mmol)
Eluent composition: pure *n*-heptane to pure DCM (gradient elution).

^{1}H NMR (300 MHz, $CDCl_3$): δ = 8.59-8.39 (m, 4H), 7.88-7.59 (m, 6H), 7.49-7.28, (m, 8H), 5.32-5.23 (m, 4H), 4.87-4.70 (m, 4H).
HRMS(ESI) calculated for $[M+H]^+$: 512.2006; found:515.2002.

Compound 55:

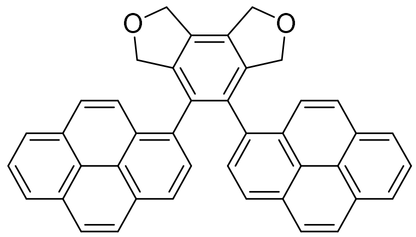

The cyclization reaction was conducted according to GP1 with compound **34** (226, 0.400 mmol) and 10 mol% of **Mn7** (reaction conditions: 80 °C for 1 h reaction time).
Yield: 69% (155 mg, 0.276 mmol)
Eluent composition: pure *n*-heptane to pure DCM (gradient elution).

^{1}H NMR (500 MHz, $CDCl_3$): δ = 8.27-7.41 (m, 18H), 5.40-5.29 (m, 4H), 4.97-4.70 (m, 4H).
^{13}C NMR (126 MHz, $CDCl_3$): δ = 140.61, 140.51, 134.29, 134.15, 133.65, 133.19, 131.77, 131.65, 131.31, 131.10, 130.98, 130.73, 130.50, 130.4005, 128.72, 128.44, 128.18, 127.49, 127.41, 127.34, 127.32, 127.15, 126.76, 126.14, 125.86, 125.41, 125.19, 125.13, 125.00, 124.98, 124.82, 124.65, 124.60, 124.56, 124.06, 73.99 (overlapping peaks), 73.17, 73.14.
HRMS(ESI) calculated for $[M+H]^+$: 563.2006; found: 563.2005.

Compound 56:

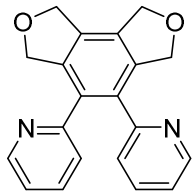

The cyclization reaction was conducted according to GP1 with compound **35** (127 mg, 0.400 mmol) and 10 mol% of **Mn7** (reaction conditions: 80 °C for 1 h reaction time).
Yield: 94% (119 mg, 0.376 mmol)
Eluent composition: pure *n*-heptane to pure acetone (gradient elution).

^{1}H NMR (500 MHz, $CDCl_3$): δ = 8.60 (dt, J = 5.0, 1.3 Hz, 2H), 7.39 (td, J = 7.7, 1.8 Hz, 2H), 7.10 (ddd, J = 7.6, 4.9, 1.2 Hz, 2H), 6.81 (d, J = 7.8 Hz, 2H), 5.13 (s, 4H), 5.12 (s, 4H).
^{13}C{^{1}H} NMR (126 MHz, $CDCl_3$): δ = 157.60, 149.50, 139.80, 135.78, 132.98, 132.85, 125.46, 121.92, 73.81, 72.47.
HRMS(ESI) calculated for $[M+Na]^+$: 339.1104; found: 339.1105.

Compound 57:

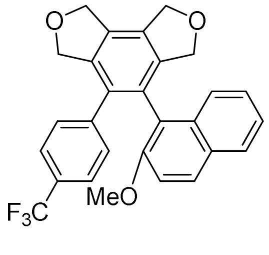

The cyclization reaction was conducted according to GP1 with compound **36** (185 mg, 0.400 mmol) and 10 mol% of **Mn7** (reaction conditions: 80 °C for 1 h reaction time).
Yield: 52% (96 mg, 0.208 mmol)

Eluent composition: pure *n*-heptane to pure DCM (gradient elution).

^{1}H NMR (500 MHz, CD_2Cl_2): δ = 7.83-7.75 (m, 2zH), 7.38-7.17 (m, 8H), 7.39-7.34 (m, 2H), 5.22-5.15 (m, 4H), 5.02-5.97 (m, 2H), 4.69-4.56 (m, 2H), 3.74 (s, 3H).
^{13}C{^{1}H} (126 MHz, CD_2Cl_2): δ = 153.73, 142.84, 140.44, 138.56, 134.41, 132.54, 132.24, 132.20, 132.06, 129.85, 128.81, 128.63, 128.63 (q, *J* = 3.7 Hz), 128.12, 126.92, 124.41 (q, *J* = 32.3 Hz), 124.19 (q, *J* = 272.5 Hz), 124.04, 123.51, 119.88, 112.46, 73.31, 73.30, 72.79, 72.72, 55.83.
^{19}F{^{1}H} NMR (471 MHz, CD_2Cl_2): δ = -62.83.
HRMS(ESI) calculated for $[M+H]^+$: 463.1516; found: 463.1512.

Compound 59:

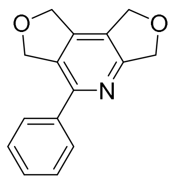

The cyclization reaction was conducted according to GP1 with compound **58** (96 mg, 0.400 mmol) and 20 mol% of **Mn7** (reaction conditions: 80 °C for 1 h reaction time).
Yield: 52% (57 mg, 0.217 mmol)
Eluent composition: gradient elution from DCM to DCM/acetone (v/v 10:1).

^{1}H NMR (300 MHz, $CDCl_3$): δ = 7.71-7.66 (m, 2H), 7.49-7.36 (m, 3H), 5.30-5.28 (t, *J* = 2.0 Hz, 2H), 5.13 (s, 4H), 5.05-5.03 (t, *J* = 2.1 Hz, 2H).
^{13}C{^{1}H} NMR (75 MHz, $CDCl_3$): δ = 160.48, 151.81, 143.70, 138.86, 131.51, 129.02, 128.81, 127.71, 124.13, 73.10, 72.89, 71.75, 71.17.
HRMS(ESI) calculated for $[M+H]^+$: 240.1019; found: 240.1019.

Compound 61:

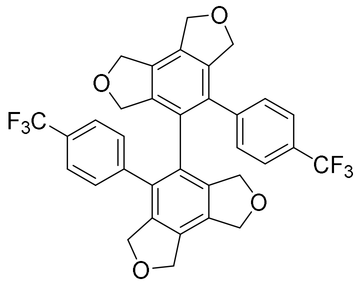

The cyclization reaction was conducted according to GP1 with compound **60** (244 mg, 0.400 mmol) and 20 mol% of **Mn7** (reaction conditions: 80 °C for 1 h reaction time).
Yield: 48% (117 mg, 0.192 mmol)
Eluent composition: gradient elution with DCM to DCM/acetone (v/v, 10:1).

^{1}H NMR (300 MHz, $CDCl_3$): δ = 7.36 (d, *J* = 8.0 Hz, 4H), 6.54 (d, *J* = 8.0 Hz, 4H), 5.22-4.49 (m, 16H).
^{13}C{^{1}H} NMR (126 MHz, $CDCl_3$): δ = 158.10, 141.33, 133.11, 132.58, 132.42, 132.16, 129.64 (q, *J* = 32.8 Hz), 129.55, 129.22, 125.07, (q, *J* = 3.7 Hz), 124.0 (q, *J* = 271.9 Hz), 74.03, 73.42, 72.96, 72.76.
^{13}C{^{1}H} NMR (126 MHz, $CDCl_3$): δ = 141.32, 139.67, 139.18, 133.09, 132.58, 132.41, 129.63 (q, *J* = 32.7 Hz), 129.54, 129.21, 125.05 (q, *J* = 3.8 Hz), 124.05 (q, *J* = 272.4 Hz), 74.01, 73.40, 72.95, 72.75
^{19}F{^{1}H} (471 MHz, $CDCl_3$): δ = -62.54.
HRMS(ESI) calculated for $[M+H]^+$: 611,1652; found: 611.1653.

Compound 63:

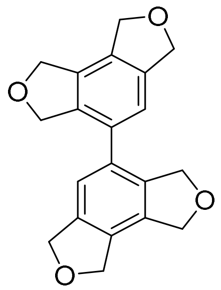

The cyclization reaction was conducted according to GP1 with compound **62** (129 mg, 0.400 mmol) and 20 mol% of **Mn7** (reaction conditions: 80 °C for 1 h reaction time).
Yield: 86% (111 mg, 0.344 mmol)
Eluent composition: gradient elution with DCM to DCM/acetone (v/v, 10:1).

^{1}H NMR (300 MHz, $CDCl_3$): δ = 7.00 (s, 2H), 5.20-4.91 (m, 16H). ^{1}H NMR (300 MHz, $CDCl_3$): δ = 7.00, 5.15, 5.09, 5.08, 5.07, 4.98.
^{13}C{^{1}H} NMR (75 MHz, $CDCl_3$): δ = 139.80, 137.35, 133.50, 133.17, 132.08, 119.93, 73.44, 73.35, 72.71, 72.29.
HRMS(ESI) calculated for $[M+NH_4]^+$: 340.1543; found: 340.1545.

Compound 65:

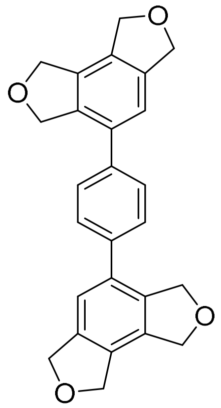

The cyclization reaction was conducted according to GP1 with compound **64** (159 mg, 0.400 mmol) and 20 mol% of **Mn7** (reaction conditions: 80 °C for 1 h reaction time).
Yield: 84% (134 mg, 0.336 mmol)
Eluent composition: gradient elution with DCM to DCM/acetone (v/v, 10:1).

^{1}H NMR (500 MHz, $CDCl_3$): δ = 7.46 (s, 4H), 7.25 (s, 2H), 5.24 (s, 4H), 5.19 (s, 4H), 5.11-5.09 (m, 8H).
^{13}C NMR (126 MHz, $CDCl_3$): δ = 140.11, 139.43, 137.03, 135.03, 133.26, 131.74, 128.33, 120.17, 73.64, 73.52, 72.57, 72.34.
HRMS(ESI) calculated for $[M+NH_4]^+$: 416.1856; found: 416.1855.

Compound 67:[12]

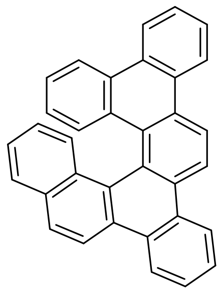

The cyclization reaction was conducted according to GP1 with compound **66** (171 mg, 0.400 mmol) and 20 mol% of **Mn7** (reaction conditions: 80 °C for 1 h reaction time).
Yield: 36% (62 mg, 0.145 mmol)
Eluent composition: pure *n*-heptane to pure DCM (gradient elution).

^{1}H NMR (300 MHz, CD_2Cl_2): δ = δ = 8.92-8.77 (m, 1H), 8.75-8.68 (m, 2H), 8.44 (d, *J* = 8.3 Hz, 1H), 8.04 (d, *J* = 8.9 Hz, 1H) 7.81-7.68 (m, 5H, 7.54 (d, *J* = 8.4 Hz, 1H), 7.31 (d, *J* = 8.6 Hz, 1H), 7.24-7.14 (m, 2H), 6.76-6.71 (m, 1H), 6.66-6.56 (m, 1H).
HRMS(ESI) calculated for $[M+H]^{-}$: 427.1492; found: 427.1496.

2.2.2 General procedure for the cyclization of diynes with phosphaalkynes (GP2)

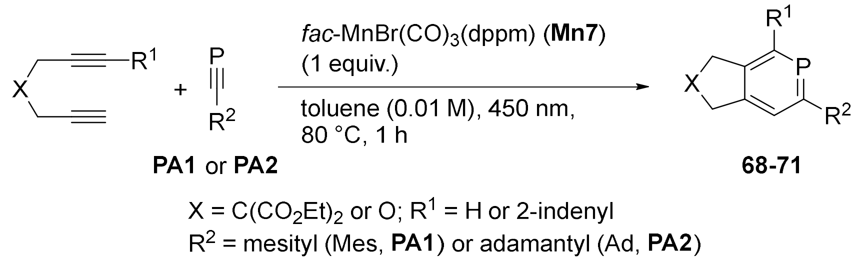

In the glove-box, **Mn7** (121.0 mg, 0.200 mmol), the respective diyne (0.200 mmol), the corresponding phosphaalkyne (0.200 mmol) and 20 mL dry and degassed toluene were combined in a 20 mL scintillation vial that was sealed with duct tape and irradiated in PRS1 at 80 °C for 1 h. The reaction mixture was charged on silica and the product isolated by flash column chromatography, followed by the removal of volatiles *in vacuo*.

Compound 68:

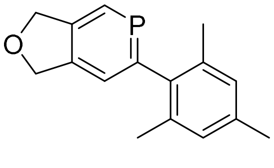

The cyclization reaction was conducted according to GP2 using dipropargylether (21 µL, 0.204 mmol) and mesitylphosphaalkyne (33 mg, 0.203 mmol).
Yield: 42% (22 mg, 0.084 mmol)
Eluent composition: *n*-heptane:DCM (20:1).

^{1}H NMR (500 MHz, $CDCl_3$): δ = δ = 8.71 (d, *J* = 37.1 Hz, 1H), 7.45 (d, *J* = 5.6 Hz, 1H), 6.98 (s, 2H), 5.24-5.19 (m, 4H), 2.34 (s, 3H), 2.02 (s, 6H).
$^{13}C\{^{1}H\}$ NMR (126 MHz, $CDCl_3$): δ = 170.79 (d, *J* = 54.7 Hz), 147.41 (d, *J* = 59.5 Hz), 142.74 (d, *J* = 14.5 Hz), 142.36 (d, *J* = 14.6 Hz), 139.49 (d, *J* = 20.9 Hz), 137.08 (d, *J* = 1.7 Hz), 135.67 (d, *J* = 5.2 Hz), 135.66, 128.63, 128.54, 128.48, 75.07, 75.04, 21.71 (d, *J* = 1.7 Hz), 21.17.
$^{31}P\{^{1}H\}$ NMR (121 MHz, $CDCl_3$): δ = 203.5.

HRMS(ESI) calculated for $[M+H]^+$: 257.1090; found: 257.1030.

Compound 69:

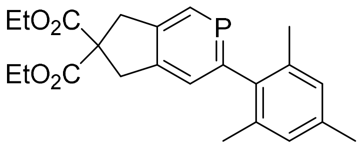

The cyclization reaction was conducted according to GP2 using dipropargylmalonate (48 mg, 0.203 mmol) and mesitylphosphaalkyne (33 mg, 0.203 mmol).
Yield: 37% (30 mg, 0.074 mmol)
Eluent composition: *n*-heptane:DCM (5:1).

^{1}H NMR (300 MHz, $CDCl_3$): δ = 8.66 (d, *J* = 37.6 Hz, 1H), 7.41 (d, *J* = 5.7 Hz, 1H), 6.94 (s, 2H), 4.22 (q, *J* = 7.10 Hz, 4H), 3.78-3.64 (m, 4H), 2.32 (s, 3H), 1.99 s, 6H), 1.27 (t, *J* = 6.9 Hz, 6H).
^{13}C{^{1}H} NMR (75 MHz, $CDCl_3$): δ = 171.60 170.14 (d, *J* = 53.2 Hz), 150.73 (d, *J* = 57.5 Hz), 143.75 (d, *J* = 14.9 Hz), 143.04 (d, *J* = 16.3 Hz), 139.80 (d, *J* = 20.83 Hz), 136.85 (d, *J* = 1.9 Hz), 135.70 (d, *J* = 5.19 Hz), 131.98 (d, *J* = 11.8 Hz), 128.4, 59.19, 42.74, 42.29 (d, *J* = 2.3 Hz) 22.64, 21.68 (d, *J* = 1.8 Hz), 21.15, 14.36.
^{31}P{^{1}H} NMR (121 MHz, $CDCl_3$): δ = 200.3.
HRMS(ESI) calculated for $[M+H]^+$: 399.1720; found: 399.1719.

Compound 70:[13]

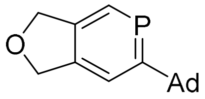

The cyclization reaction was conducted according to GP2 with dipropargylether (21µL, 0.204 mmol) and adamantylphospaalkyne (36 mg, 0.202 mmol).
Yield: 42% (23 mg, 0.084 mmol)
Eluent composition: *n*-heptane:DCM (20:1).

^{1}H NMR (300 MHz, $CDCl_3$): δ = 8.65 (d, *J* = 37.5 Hz, 1H), 7.80 (d, *J* = 5.6 Hz, 1H), 5.11-5.20 (m, 4H), 2.04-2.16 (m, 9H), 1.75-1.82 (m, 6H).
^{13}C{^{1}H} NMR (75 MHz, $CDCl_3$): 184.45 (d, *J* = 61.7 Hz), 145.26 (d, *J* = 53.2 Hz), 142.08 (d, *J* = 14.9 Hz), 141.65 (d, *J* = 15.2 Hz), 124.12, (d, *J* = 14.3 Hz), 75.16 (d, *J* = 2.7 Hz), 74.90, 45.38 (d, *J* = 12.6 Hz), 36.75, 29.32 (d, *J* = 2.2 Hz).
^{31}P{^{1}H} NMR (121 MHz, $CDCl_3$): δ = 198.52.
HRMS(ESI) calculated for $[M+H]^+$: 273.1403; found: 273.1405.

Compound 71a and 71b:

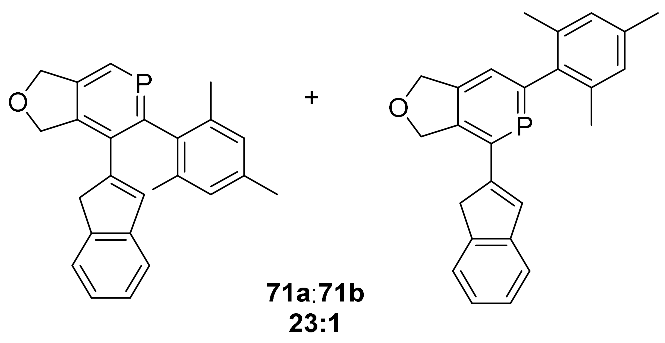

The cyclization reaction was conducted according to GP2 using **SI-16** (42 mg, 0.200 mmol) and mesitylphosphaalkyne (33 mg, 0.203 mmol).
Yield: 34% (25 mg, 0.068 mmol)
Eluent composition: *n*-heptane/DCM (20:1).

^{1}H NMR (500 MHz, $CDCl_3$): δ = 8.65 (d, J = 36.9 Hz, 1H), 7.39-7.11 (m, 4H), 6.86 (s, 2H), 6.75 (s, 1H), 5.33-5.14 (m, 4H), 3.16 (s, 2H), 2.26 (s, 3H), 2.05 (s, 6H) (**71a**).
^{13}C{^{1}H} NMR (126 MHz, $CDCl_3$): δ = 168.23 (d, J = 55.6 Hz), 146.58 (d, J = 59.5 Hz), 146.13 (d, J = 1.98 Hz), 144.17, 144.05, 143.85, 142.2 (d, J = 14.04 Hz), 138.34 (d, J = 12.20 Hz), 137.90 (d, J = 31.91 Hz), 137.17 (d, J = 2.50 Hz), 135.91 (d, J = 5.82 Hz), 131.16, 128.60, 126.40, 124.94, 123.57, 121.22, 76.00, 75.87 (d, J = 2.17 Hz), 40.93, 21.74 (d, J = 1.748Hz), 21.16 (**71a**).
^{31}P{^{1}H} NMR (202 MHz, $CDCl_3$): δ = 207.4 (**71a**), 199.7 (**71b).**
*^{1}H NMR and ^{13}C{^{1}H} NMR data for **71b** were not fully assignable due to the low concentration of the regioisomer.*
HRMS(ESI) calculated for $[M+H]^+$: 371.1559; found: 371.1559.

2.3 General procedure for Suzuki-Miyaura cross-coupling reactions

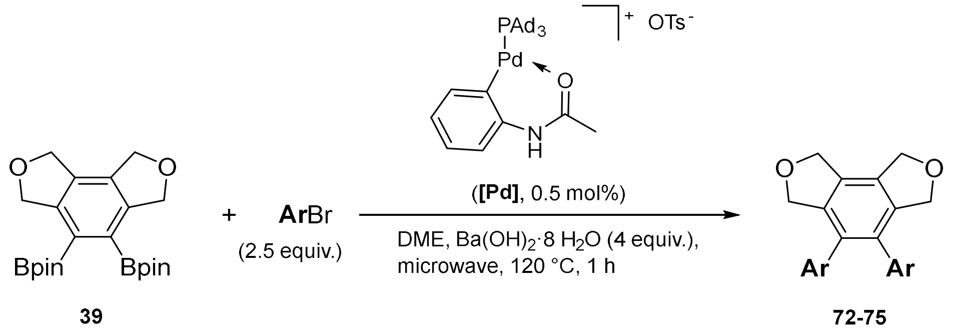

In the glove-box, a 20 mL microwave vial was charged with **[Pd]**[14] (1 mL of a 1 µm stock solution in THF) cyclization product **39** (83 mg, 0.200 mmol), 2.5 equiv. of the aryl bromide (99 mg, 0.500 mmol 1-(4-bromophenyl)ethan-1-one for compound **72**; 101 mg, 0.500 mmol 1-bromo-3-nitrobenzene for compound **73**; 75 µL, 0.500 mmol 2-bromo-1,3,5-trimethylbenzene for compounds **74** and **75**), $Ba(OH)_2 \cdot 8\ H_2O$ (252 mg, 0.800 mmol, 4 equiv.), and 1,2-dimethoxyethane (10 mL) under argon. The reaction was stirred at 120 °C for 1 h followed by filtration over cotton at room temperature. The solvent was distilled off and the products were isolated by flash column chromatography.

Compound 72:

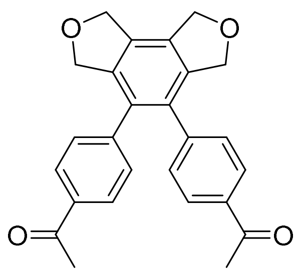

Yield: 89% (71 mg, 0.178 mmol)
Eluent composition: pure DCM to DCM:acetone (10:1 v/v) (gradient elution).
^{1}H NMR (300 MHz, $CDCl_3$): δ = 7.80-7.04 (m, 8H), 5.14 (s, 4H), 4.96 (s, 4H), 2.55 (s, 6H).
^{13}C{^{1}H} NMR (75 MHz, $CDCl_3$): δ = 197.65, 143.30, 138.97, 135.85, 133.04, 129.77, 128.37, 79.51, 72.80, 26.63.
HRMS(ESI) calculated for $[M+H]^+$: 416.1884; found: 416.1886.

Compound 73:

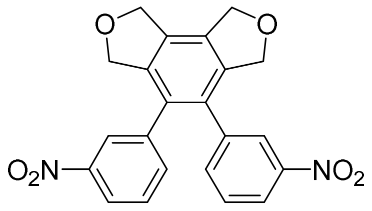

Yield: 91% (74 mg, 0.182 mmol)
Eluent composition: pure DCM to DCM:acetone (10:1 v/v) (gradient elution).
^{1}H NMR (300 MHz, $CDCl_3$): δ = 8.09-8.04 (m, 2H), 7.94-7.89 (m, 2H), 7.46-7.37 (m, 4H), 5.17 (s, 4H), 4.98 (s, 4H).
^{13}C{^{1}H} NMR (75 MHz, $CDCl_3$): δ = 148.13, 139.51, 139.27, 135.56, 133.17, 131.72, 129.67, 124.29, 122.57, 73.25, 72.79.
HRMS(ESI) calculated for $[M+NH_4]^+$: 422.1345; found: 422.1347.

Compound 74 and 75:

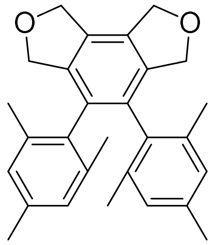

74

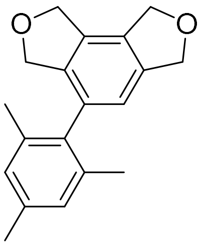

75

Yield: 50 mg (combined mass of **74** and **75**)
Eluent composition: pure *n*-heptane to pure DCM (gradient elution).

74: ^{1}H NMR (300 MHz, $CDCl_3$): δ = 6.95 (s, 4H), 5.18 (s, 4H, overlapping signals), 4.75 (s, 4H), 2.34 (s, 6H), 1.97 (s, 12H).
HRMS(ESI) calculated for $[M+NH_4]^+$: 416.2584; found: 416.2574.

75 :^{1}H NMR (300 MHz, $CDCl_3$): δ = 6.89 (s, 2H), 6.73 (s, 1H), 5.18 (s, 2H, overlapping signals), 5.13 (s, 2H), 5.11 (s, 2H), 4.69 (s, 2H) 2.21 (s, 3H), 1.96 (s, 6H).
HRMS(ESI) calculated for $[M+H]^+$: 281.1536; found: 281.1539.

74 and **75**: $^{13}C\{^1H\}$ NMR (75 MHz, $CDCl_3$): δ = 140.17, 139.76, 137.66, 137.19, 136.55, 136.24, 135.89, 135.63, 134.39, 134.13, 133.79, 132.43, 130.99, 130.48, 128.41, 128.34, 120.63, 74.31, 73.59, 73.57, 73.08, 72.91, 72.35, 21.18, 21.07, 20.68, 20.41.

2.4 Intermolecular and partially intermolecular cyclizations

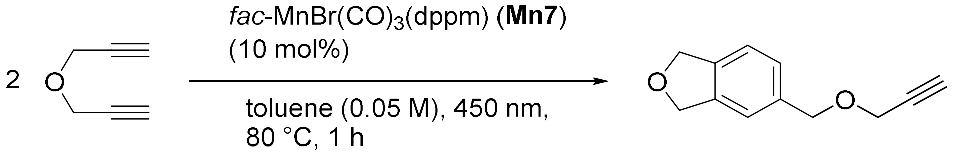

76

Synthesis of compound 76[15]

In the glovebox, **Mn7** (301 mg, 0.5 mmol, 10 mol%) and dipropargylether (514.8 µL, 5 mmol) were dissolved in 100 mL toluene and irradiated at 450 nm wavelength at 80 °C for 1 h. The solvent was distilled off and the products were isolated by flash column chromatography (gradient elution with heptane and EtOAc). The product was obtained as colourless oil (42 mg, 0.223 mmol, 9%).

^1H NMR (300 MHz, $CDCl_3$): δ = 7.29-7.21 (m, 3H), 5,11 (s, 4H), 4.63 (s, 2H), 4.19 (d, J = 2.4 Hz, 2H), 2.48 (t, J = 2.4 Hz, 1H).
$^{13}C\{^1H\}$ NMR (75 MHz, $CDCl_3$): δ = 139.71, 139.07, 136.75, 127.49, 121.05, 120.89, 79.69, 74.84, 73.53, 71.49, 57.24.

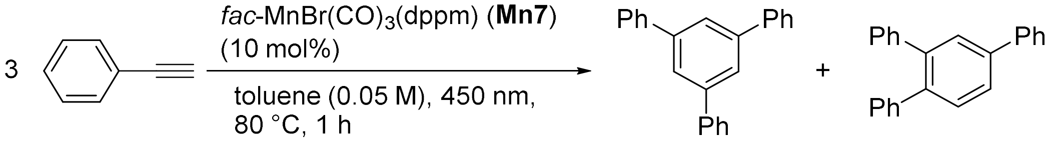

77 **78**

Synthesis of compounds 77[16] and 78[17]

In the glovebox, **Mn7** (301 mg, 0.5 mmol, 10 mol%) and phenylacetylen (549.1 µL, 5 mmol) were dissolved in 100 mL toluene and irradiated at 450 nm wavelength at 80 °C for 1 h. The solvent was distilled off and the products were isolated by flash column chromatography (gradient elution with heptane and DCM). The product were obtained as mixture as white solid (61 mg, 0.20 mmol, 12%).

77 and **78**: ^1H NMR (300 MHz, $CDCl_3$): δ = 7.80 (s, 3H), 7.76-7.65 (m, 14), 7.54-7.36 (m, 14), 7.29-7.17 (m, 12).
77 and **78**: $^{13}C\{^1H\}$ NMR (75 MHz, $CDCl_3$): δ = 142.50*, 141.64, 141.30, 141.27, 141.14*, 140.75, 140.52, 139.70, 131.25, 130.06, 130.02, 129.57, 129.00, 128.98*, 128.08, 128.05, 127.70, 127.58*, 127.51, 127.29*, 126.75, 126.67, 126.27, 125.33*.
*Signals resulting from the 1,3,5-regioisomer.
The ratio of the 1,3,5- and the 1,2,4-regiosiomer (54:46) was determined by GC-MS.

2.5 Overview of screening reactions

2.5.1 Screening reactions with PRS1 (370 W)

2.5.1.1 *In situ* screening with $MnBr(CO)_5$ (Mn1) and phosphine ligands

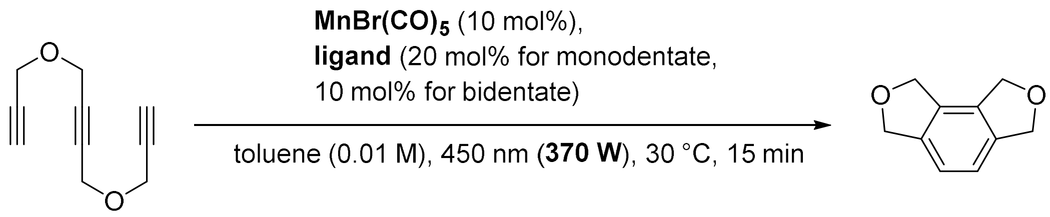

4 **5**

In the glove-box, toluene stock solutions of **Mn1**, the corresponding ligand and triyne **4** were combined in 4 mL vials and toluene was added to obtain 2 mL of a 0.01 M solution. The vials were irradiated while stirring in PRS1 for 30 min at 30 °C. The reaction mixture was filtrated over celite and the filtrate was analyzed by GC-MS.

#	Ligand	Yield 5 [%] [a]
1	Triphenylphosphine (**L1**)	98
2	Triphenylphosphite (**L2**)	46
3	Tris(1-naphthyl) phosphine (**L3**)	28
4	Tris(pentafluorophenyl) phosphine (**L4**)	18
5	Triisopropylphosphine (**L5**)	51
6	1,1-Bis(diphenylphosphino) methane (dppm, **L6**)	>99
7	1,2-Bis(diphenylphosphino) ethane (dppe, **L7**)	< 5
8	1,3-Bis(diphenylphosphino) propane (dppp, **L8**)	< 5
9	1,2-Bis(diphenylphosphino) benzene (dppbenz, **L9**)	< 5
11	No ligand	24

2.5.1.2 Solvent screening with Mn7

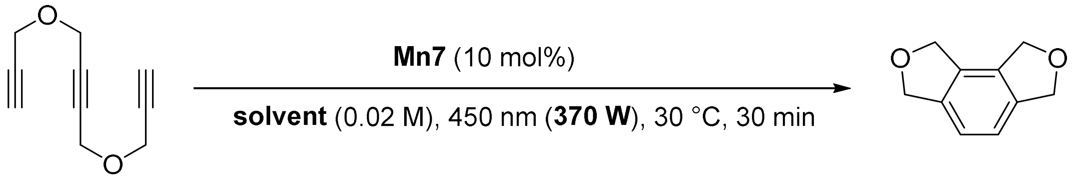

4 **5**

The reactions were performed as described in GP1 in 10 mL solvent with triyne **4** (33 mg, 0.204 mmol) and **Mn7** (12 mg, 0.020 mmol) in 20 mL scintillation vials. The reaction mixture was filtrated over celite and the filtrate was analyzed by GC-MS.

Solvent	Yield 5 [%]
toluene	>99
THF	14
acetonitrile	12
1,2-dichloroethane	no reaction

2.5.1.3 Screening of precatalysts

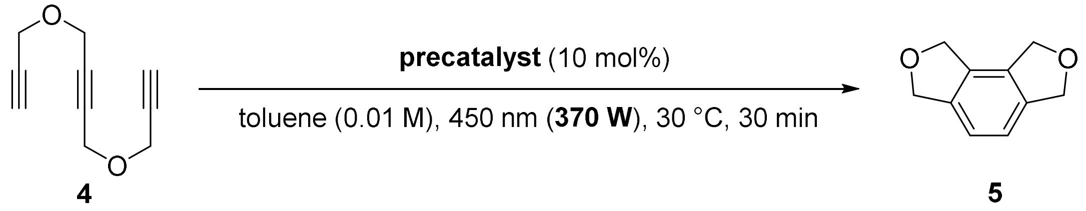

The reactions were performed according to GP1 in 20 mL solvent with triyne **4** (33 mg, 0.204 mmol) and the corresponding precatalyst (0.020 mmol, 10 mol%) in 20 mL scintillation vials. The reaction mixture was filtrated over celite, and the filtrate was analyzed by GC-MS.

**Precatalyst**	**Yield 5 [%]**
$MnBr(CO)_5$ (**Mn1**)	42
$MnCl(CO)_5$ (**Mn2**)	27
$Mn(OTf)(CO)_5$ (**Mn3**)	11
Cp'$Mn(CO)_3$ (**Mn4**)	< 5
$Mn_2(CO)_{10}$ (**Mn5**)	20
$ReBr(CO)_5$ (**Re**)	< 5
$Mo(CO)_6$ (**Mo**)	11

2.5.1.4 Comparative experiments of Mn7 or Mn1 with phosphine and isonitrile ligands

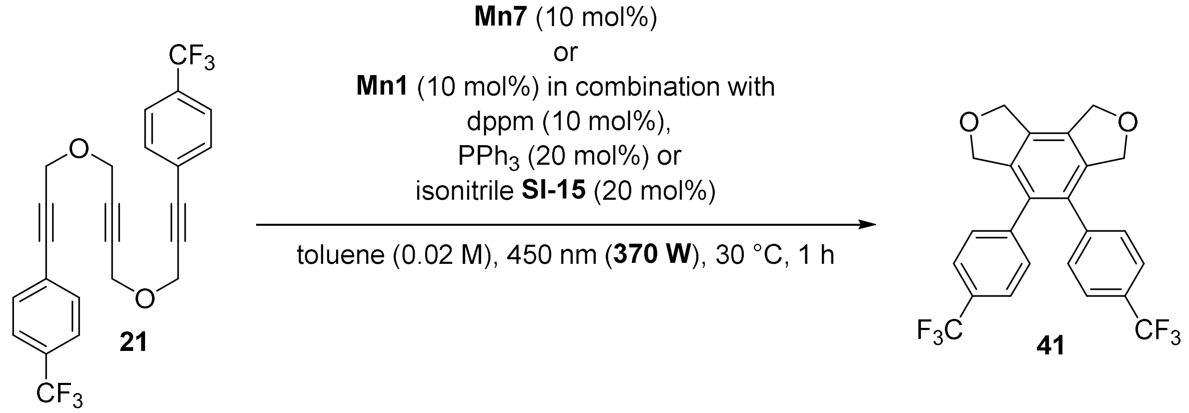

The reactions were conducted according to GP1 with Triyne **21** (180 mg, 0.4 mmol) and **Mn7** (24 mg, 0.04 mmol, 10 mol%) or **Mn1** (11 mg, 0.04 mmol, 10 mol%) in combination with dppm (15 mg, 0.04 mmol, 10 mol%), PPh_3 (21 mg, 0.08 mmol, 20 mol%) or isonitrile **SI-15** (15 mg, 0.08 mmol, 20 mol%).

**Applied catalyst system**	**Yield 41 [%]**
**Mn7**	91
**Mn1** + 10 mol% dppm	86
**Mn1** + 20 mol% PPh_3	84
**Mn1** + 20 mol% isonitrile **SI-15**	17%

2.5.1.5 Light on-off and time-conversion experiments

In the glove-box, toluene stock solutions of **Mn7** and triyne **4** were combined in 4 mL vials to obtain 2 mL of a 0.02 M solution. The vials were irradiated simultaneously while stirring in PRS1 and subjected to GC-MS analysis after filtration over celite after the indicated reaction time.

Figure SI-5: Light on-off and time-conversion experiments.

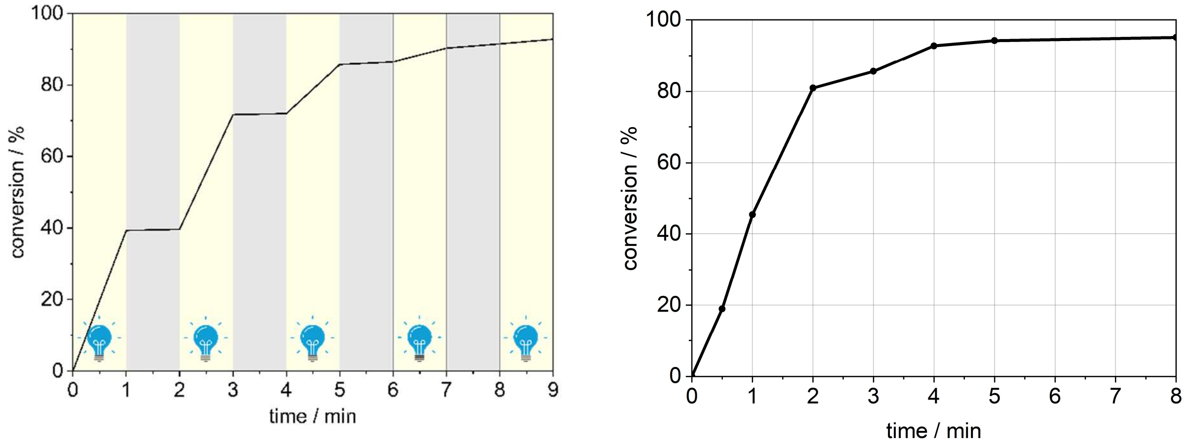

2.5.1.6 Photochemical cyclization of compound 4 followed by attempted thermal cyclization with Mn7

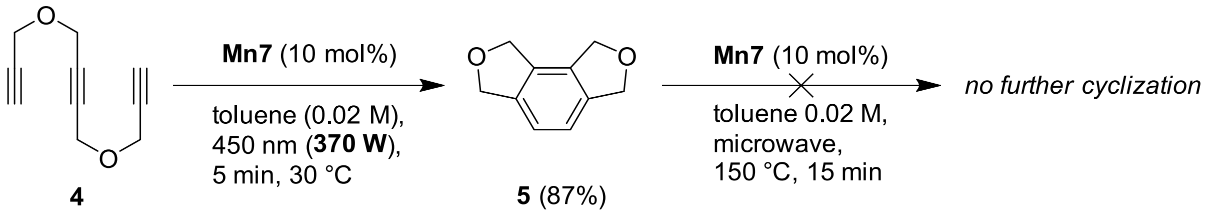

In the glovebox, two 20 mL microwave vial was charged with compound **4** (32 mg, 0.200 mmol), **Mn7** (12 mg, 0.020 mmol) and 10 mL toluene. The vial was sealed with a crimp cap and reacted in PRS1 for 5 min at 30 °C. One reaction was filtrated over celite and analyzed by GC-MS (yield: 87%), while the other was heated in the microwave at 150 °C for 15 min followed filtration over celite and GC-MS analysis. No further conversion of triyne **4** was observed in the subsequent thermal reaction.

2.5.2 Screening reactions with PRS2 (30 W)

2.5.2.1 Filtration experiment

In the glove-box, toluene stock solutions of **Mn7** and triyne **4** were combined in 4 mL vials to obtain 2 mL of a 0.02 M solution. The vials were irradiated simultaneously while stirring in PRS2 inside the glovebox. After 2 min of irradiation, 1 mL aliquots of the turbid reaction mixtures were filtrated over celite and analyzed by GC-MS. One of the reactions was filtrated with a 0.2 µm syringe filter to obtain a yellow homogeneous filtrate while the other was not filtrated. Both remainders were further irradiated simultaneously in 4 mL vials for 5 min in PRS2 in the glove-box before filtration over celite and analysis by GC-MS.

Yield after 2 min of irradiation: 22%, 21% (reaction to be filtrated).

Yield after further 5 min of irradiation: 76% (non-filtrated reaction), 86% (filtrated reaction).

2.5.3 Attempted cyclization under CO atmosphere

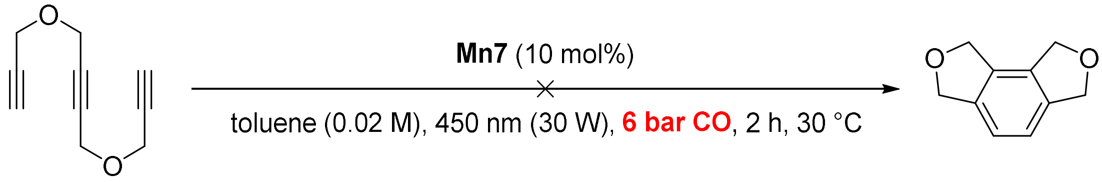

4 5

CAUTION: CO is a highly toxic gas! It is crucial to ensure proper ventilation and utilize appropriate safety measures!

In the glovebox, a 15 mL Schlenk tube (specified for a pressure of 15 bar) was charged with compound **4** (32 mg, 0.20 mmol), **Mn7** (12 mg, 0.020 mmol) and 10 mL toluene. An autoclave containing around 6 bar CO was connected to the Schlenk flask and the Schlenk line with a T-shaped cross valve via tubing. The reaction was frozen with liquid dinitrogen and the Schlenk tube as well as the tubes were evacuated and backfilled with argon three times. After a final evacuation, the Schlenk line valve was closed and the autoclave was opened to fill the Schlenk tube with CO pressure. As soon as the reaction mixture was liquid and reached room temperature the Schlenk tube was irradiated in PRS2 for 2 h at 30 °C and subjected to GC-MS analysis after filtration over celite and ensuring removal of CO by opening the reaction to a vent pipe for safe removal the CO and stirring for 15 min on air in the fume hood.

2.6 Attempted thermal cyclization with Mn7

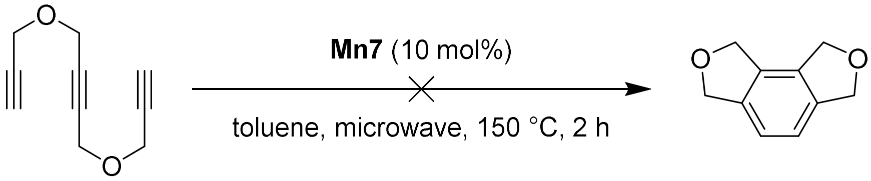

4 5

In the glovebox, a 20 mL microwave vial was charged with compound **4** (32 mg, 0.200 mmol), **Mn7** (12 mg, 0.020 mmol) and 10 mL toluene. The vial was sealed with a crimp cap and reacted in the microwave under exclusion of light for 2 h at 150 °C. The reaction was filtered through celite, and volatiles were removed *in vacuo*. ^{1}H NMR analysis of the obtained residue indicated no reaction of compound **4**.

2.7 Synthesis of new substrates and reported compounds

Table SI-1: Overview of compounds synthesized according to published literature procedures. Corresponding NMR data is provided in section 3.3.

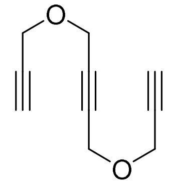<br>**4**<br>Synthesis[18]<br>Analytical data[10]	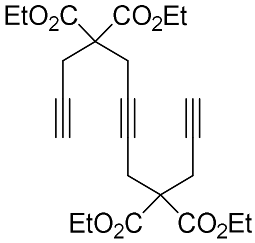<br>**6**<br>Synthesis and analytical data[5]	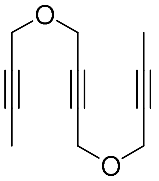<br>**16**<br>Synthesis and analytical data[19]
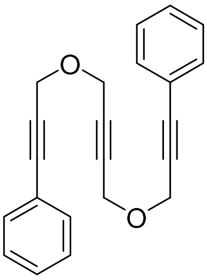<br>**17**<br>Synthesis[18]<br>Analytical data[10]	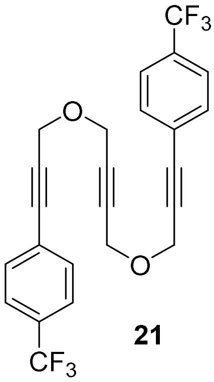<br>**21**<br>Synthesis and analytical data[9]	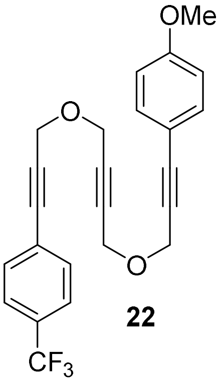<br>**22**<br>Synthesis and analytical data[9]
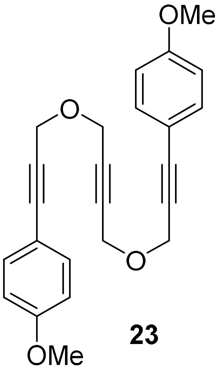<br>**23**<br>Synthesis and analytical data[9]	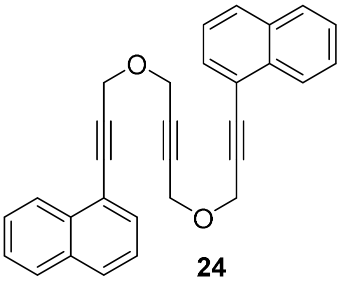<br>**24**<br>Synthesis and analytical data[20]	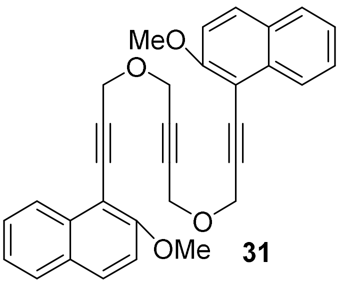<br>**31**<br>Synthesis and analytical data[20]
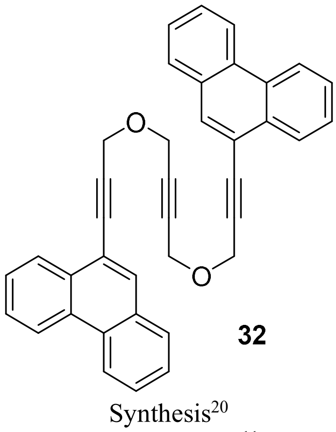<br>**32**<br>Synthesis[20]<br>Analytical data[11]	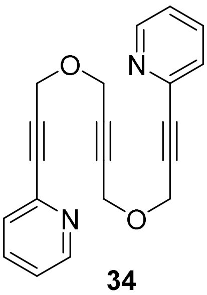<br>**34**<br>Synthesis and analytical data	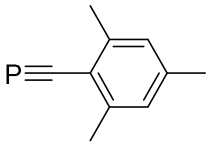<br>**PA1**<br>Synthesis[21]

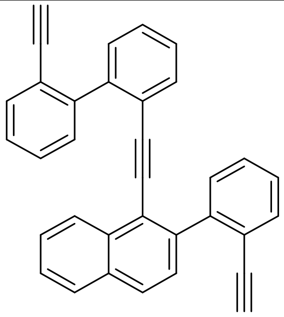<br>**64**<br>Synthesis and analytical data[12]	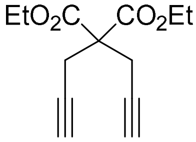<br>diyne **1**<br>Synthesis and analytical data[22]	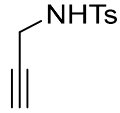<br>**SI-1**<br>Synthesis<br>and analytical data[22]
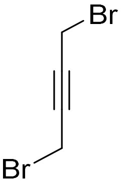<br>**SI-2**<br>Synthesis and analytical data[23]	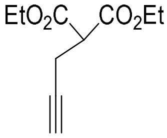<br>**SI-3**<br>Synthesis and analytical data[24]	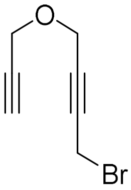<br>**SI-4**<br>Synthesis and analytical data[25]
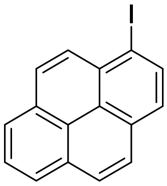<br>**SI-10**<br>Synthesis and analytical data[26]	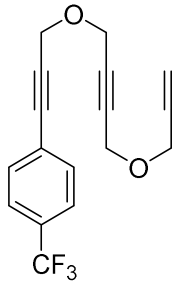<br>**SI-11**<br>Synthesis and analytical data[9]	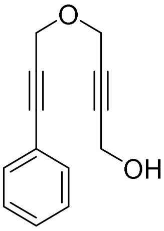<br>**SI-12**<br>Synthesis and analytical data[27]
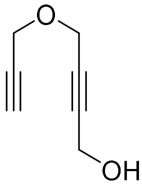<br>**SI-13**<br>Synthesis and analytical data[27]	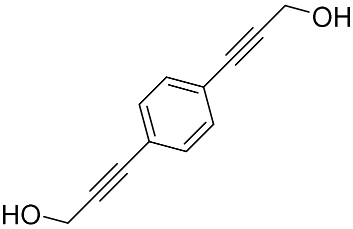<br>**SI-14**<br>Synthesis and<br>analytical data[28]	$MnCl(CO)_5$<br>Synthesis[29]<br><br>$Mn(OTf)(CO)_5$<br>Synthesis[30]
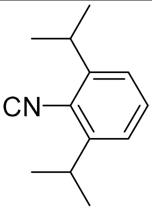<br>**SI-15**<br>Synthesis[31]	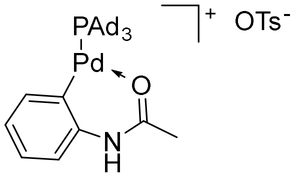<br>Synthesis and analytical data[14]	

Compound 7:[5]

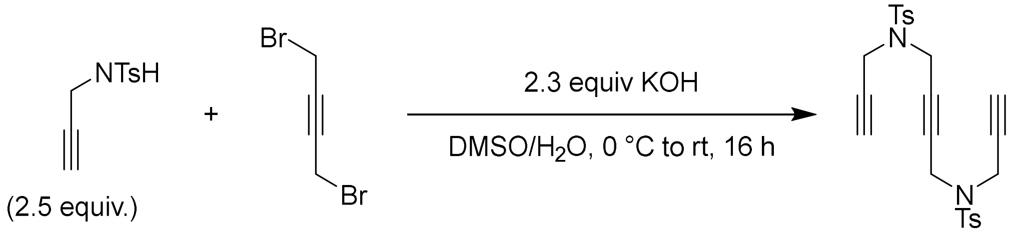

7

In a 50 mL round-bottom flask, **SI-1** (1.041 g, 5.00 mmol) was dissolved in 10 mL DMSO, aq. KOH (920 µL, 4.60 mmol, 5 M) was added and the reaction mixture was stirred at room temperature for 15 min. **SI-2** (424 mg, 2.00 mmol) as solution in 1 mL DMSO was added at 0 °C. The reaction was stirred overnight, slowly reaching room temperature. Ice, 50 mL H_2O and aq. NH_4Cl were added to quench the reaction. The aq. phase was extracted with DCM (3x30 mL), the combined organic phase was washed with H_2O (2x100 mL), dried with Na_2SO_4 and filtered. DCM was removed *in vacuo* to obtain a solid crude product. Sublimation (80° C, 0.05 mbar, 3 h) resulted in the removal of unreacted alkyne **SI-1** as white solid on the sublimation finger. The brownish product **7** was obtained as the sublimation residue (679 mg, 1.45 mmol, 73%).

^{1}H NMR (300 MHz, $CDCl_3$): δ 7.67 (d, *J* = 8.3 Hz, 2H), 7.30 (d, *J* = 8.3 Hz, 2H), 4.02 (s, 4H), 3.99 (d, *J* = 2.5 Hz, 4H) 2.43 (s, 6H), 2.12 (t, *J* = 2.4 Hz, 2H).

Compound 8:[32]

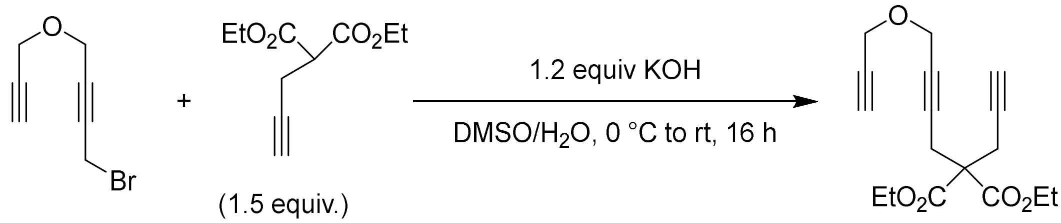

8

To a 50 mL round-bottom flask containing the **SI-3** (991 mg, 5.0 mmol) in 10 mL DMSO, aq. KOH (0.92 µL, 4.60 mmol, 5 M) was added and the reaction mixture was stirred at room temperature for 15 min. **SI-4** (935 mg, 5.00 mmol) as solution in 2 mL DMSO was added at 0 °C. The reaction was stirred overnight, slowly reaching room temperature. Ice, 50 mL H_2O and aq. NH_4Cl were added to quench the reaction. The aq. phase was extracted with EtOAc (3x50 mL), the combined organic phase was washed with H_2O (2x100 mL), dried with Na_2SO_4 and filtered. The crude product was charged on silica and purified by flash column chromatography (gradient elution from *n*-heptane to *n*-heptane:DCM (1:1)). The product 8 was obtained as yellowish oil (952 mg, 3.13 mmol, 62%).

^{1}H NMR (300 MHz, $CDCl_3$): δ = 4.26-4.18 (m, 8H), 3.03 (t, *J* = 2.1 Hz, 2H), 2.96 (d, *J* = 2.6 Hz, 2H), 2.43 (t, *J* = 2.4 Hz, 1H), (t, *J* = 2.7 Hz, 1H), 1.25 (t, *J* = 7.1 Hz, 6H).

Compound 9:[7]

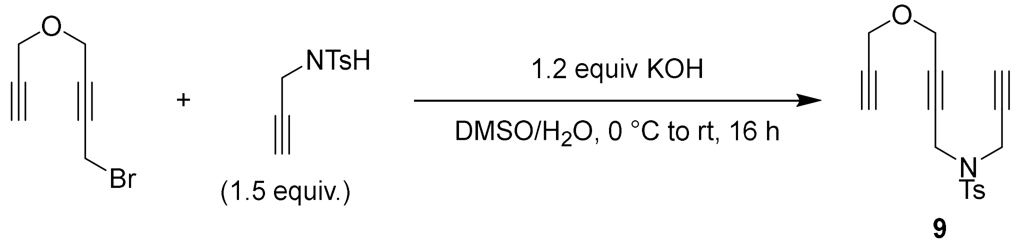

In a 50 mL round-bottom flask, **SI-1** (417 mg, 2.00 mmol) was dissolved in 5 mL DMSO, aq. KOH (0.48 mL, 2.4 mmol, 5 M) was added and the reaction mixture was stirred at room temperature for 15 min. **SI-4** (374 mg, 2.00 mmol) as solution in 1 mL DMSO was added at 0 °C. The reaction was stirred overnight, slowly reaching room temperature. Ice, 50 mL H_2O and aq. NH_4Cl were added to quench the reaction. The aq. phase was extracted with EtOAc (3x50 mL), the combined organic phase was washed with H_2O (2x100 mL), dried with Na_2SO_4 and filtered. The crude product was charged on silica and purified by flash column chromatography (gradient elution from pure *n*-heptane to pure DCM (1:1)). The product **9** was obtained as yellowish oil (451 mg, 1.43 mmol, 72%).

^{1}H NMR (300 MHz, $CDCl_3$): δ = = 7.71 (d, *J* = 8.3 Hz, 2H), 7.30 (d, *J* = 8.1 Hz, 2H), 4.2-4.07 (m, 8H), 2.45-2.36 (m, 4H), 2.16 (t, *J* = 2.4 Hz, 2H).

Compound SI-5:[33]

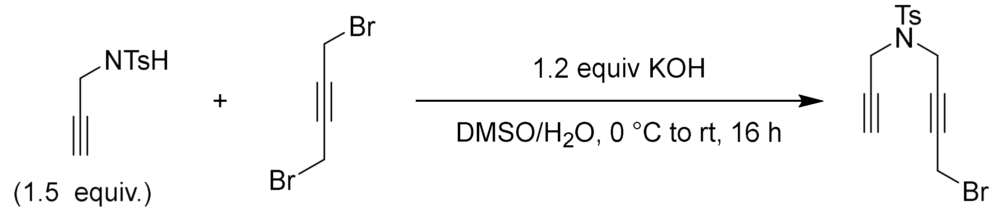

In a 50 mL round-bottom flask, **SI-1** (2.083 g, 10.0 mmol) was dissolved in 10 mL DMSO, aq. KOH (0.48 mL, 2.40 mmol, 5 M) was added and the reaction mixture was stirred at room temperature for 15 min. The solution was drawn up into a syringe and added dropwise to **SI-2** (3.179 g, 15.00 mmol) in 10 mL DMSO at 0 °C in a 100 mL round-bottom flask. The reaction was stirred overnight, slowly reaching room temperature. Ice, 50 mL H_2O and aq. NH_4Cl were added to quench the reaction. The aq. phase was extracted with EtOAc (3x50 mL), the combined organic phase was washed with H_2O (2x100 mL), dried with Na_2SO_4 and filtered. The crude product was charged on silica and purified by flash column chromatography (gradient elution from pure *n*-heptane to pure DCM (1:1)). The product was obtained as colourless oil (1.878 mg, 5.52 mmol, 55%).

^{1}H NMR (300 MHz, $CDCl_3$): δ = 7.71 (d, *J* = 8.3 Hz, 2H), 7.32 (d, (d, *J* = 8.1 Hz, 2H), 4.23 (t, *J* = 2.1 Hz 2H), 4.12 (d, *J* = 2.6 Hz, 2H), 3.71 (t, *J* = 2.1 Hz, 2H), 2.43 (s, 3H), 2.17 (t, *J* = 2.5 Hz 1H).

Compound 10:

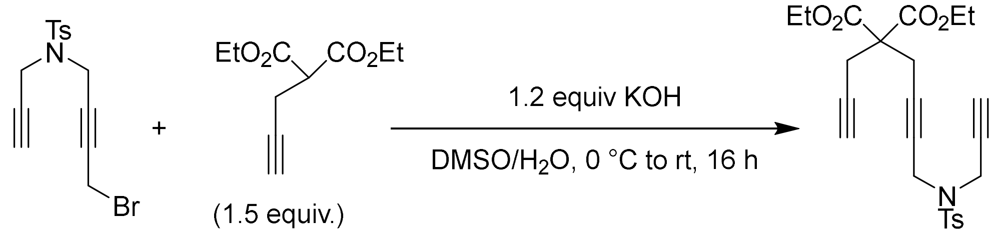

10

To a 50 mL round-bottom flask containing the **SI-4** (298 mg, 1.50 mmol) in 5 mL DMSO, aq. KOH (0.24 mL, 1.20 mmol, 5 M) was added and the reaction mixture was stirred at room temperature for 15 min. **SI-5** (341 mg, 1.00 mmol) as solution in 1 mL DMSO was added at 0 °C. The reaction was stirred overnight, slowly reaching room temperature. Ice, 50 mL H_2O and aq. NH_4Cl were added to quench the reaction. The aq. phase was extracted with EtOAc (3x50 mL), the combined organic phase was washed with H_2O (2x100 mL), dried with Na_2SO_4 and filtered. The crude product was charged on silica and purified by flash column chromatography (gradient elution from pure *n*-heptane to pure DCM (1:1)). The product **10** was obtained as yellowish oil (370.6 mg, 0.81 mmol, 81%).

^{1}H NMR (300 MHz, $CDCl_3$): δ = 7.68 (d, *J* = 8.3 Hz, 2H), 7.30 (d, *J* = 8.3 Hz, 2H), 4.18 (q, *J* = 7.1 Hz 4H), 4.12-4.08 (m, 4H), 2.83 (t, *J* = 2.1 Hz 2H), 2,75 (d, *J* = 2.5 Hz, 2H), 2.42 (s, 3H), 2.12 (t, *J* = 2.4 Hz 1H), 2.00 (t, *J* = 2.6 Hz, 1H).
$^{13}C\{^1H\}$ NMR (300 MHz, $CDCl_3$): δ = 168.64, 144.01, 135.36, 129.72, 129.65, 127.90, 80.62, 78.55, 76.42, 75.80, 73.96, 71.76, 62.17, 56.28, 36.58, 36.12, 22.82, 22.61, 21.69, 14.10.
HRMS(ESI) calculated for $[M+H]^+$: 458.1632; found: 458.1631.

Compound 18:[8]

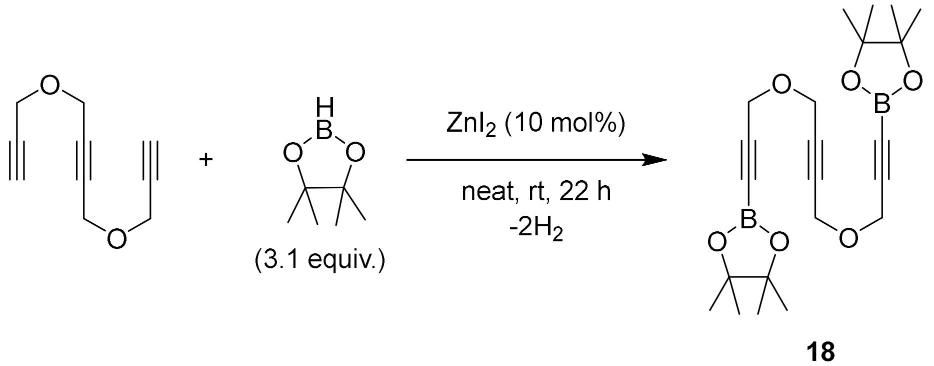

18

The attempted synthesis of triyne **18** by lithiation of triyne **4** with n-Buli in THF and quenching with iPrOBpin gave mixtures of products, including the desired diborylated and monoborylated compounds, which were difficult to separate. A detailed synthetic procedure of triyne **18** is not provided in the literature.[8] We therefore investigated and optimized a recent approach published by Ma and coworkers using a zinc-catalyzed dehydrogenative borylation with HBpin.[34]
In the glovebox, triyne **4** (1.622 g, 10.00 mmol), HBpin (4.50 mL, 31.01 mmol,) and ZnI_2 (319 mg, 1.00 mmol, 10 mol%) were added to a 250 mL Schlenk tube resulting in immediate gas evolution. The Schlenk flask was connected to the Schlenk line to ensure pressure release and the reaction mixture was stirred for 22 h at room temperature resulting in a viscous oil. 50 mL dry and degassed toluene were added, and the solution left to stand for 2 h during which a white solid accumulated on the bottom of the flask. The supernatant was transferred to a Schenk flask with a syringe and volatiles were removed *in vacuo* to obtain **18** as yellowish solid (9.1 mmol, 3.768 g, 91%).

^{1}H NMR (300 MHz, $CDCl_3$): δ = 4.30 (s, 4H), 4.28 (s, 4H), 1.27 (s, 24H).
^{11}B NMR (96 MHz, $CDCl_3$): δ = 22.50.
$^{13}C\{^1H\}$ NMR (75 MHz, $CDCl_3$): δ = 84.61, 82.26, 57.02, 56.98, 24.98. *The borylated carbon was not detectable.*

Compounds SI-6[35], SI-7[36] and SI-8[36]:

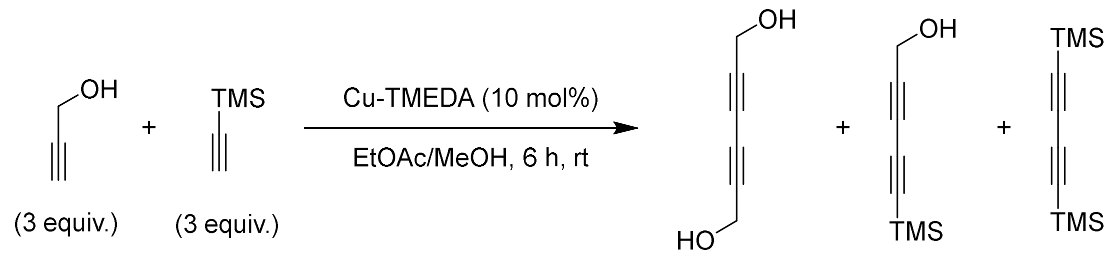

In a 500 mL round-bottom flask, propargyl alcohol (2.90 mL, 50.18 mmol) and trimethylsilylacetylene (7.20 mL, 50.58 mmol) were stirred in 100 mL MeOH and 100 mL EtOAc under air. Cu-TMEDA complex (10 mol%) was added and vigorously stirred at room temperature for 5 h. The reaction mixture was filtrated over a sintered glass frit covered with a Celite layer. 100 mL H_2O was added to the filtrate, and the aqueous phase was extracted with EtOAc (3x 50 mL). The combined organic phase was washed with H_2O (3x100 mL), dried with Na_2SO_4 and filtered. The crude product was charged on silica and the products isolated by flash column chromatography (gradient elation from pure *n*-heptane to pure acetone) to obtain product **SI-8** as white solid (1.560 mg, 8.02 mmol, 16%), product **SI-7** as yellowish oil (2.348 g, 15.42 mmol, 31%) and product **SI-6** as yellowish solid (1.94 mg, 17.62 mmol, 35%).

SI-6: ^{1}H NMR (300 MHz, DMSO.d$_6$): δ = 5.39 (t, *J* = 6.1 Hz 2H), 4.17 (d, *J* = 6.1 Hz 4H).
SI-7: ^{1}H NMR (300 MHz, $CDCl_3$): δ = 4.32 (s, 2H), 1.75 (s, 1H), 0.19 (s, 9H).
SI-8: ^{1}H NMR (300 MHz, $CDCl_3$): δ = 0.19 (s, 18H).

Compound SI-9:[37]

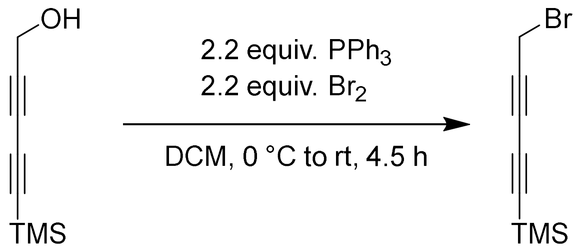

In a 250 mL Schlenk flask, PPh_3 (21.08 g, 88 mmol) was dissolved in 100 mL dry DCM, Br_2 (4.5 mL, 88 mmol) was added dropwise at 0 °C with a syringe and the reaction was stirred for 30 min at 0 °C. **SI-8** (6.106 g, 40.1 mmol) was dissolved in 15 mL dry DCM and added dropwise with a syringe. After stirring for 4 h at room temperature, the reaction mixture was poured into 500 mL of *n*-hexane and stirred for 5 min before the mixture was filtered over a frit (por 4) covered with a layer of silica (3 cm). The solvent was removed in vacuo to afford **SI-9** as yellowish oil (7.258 g, 33.7 mmol, 82%)

^{1}H NMR (300 MHz, $CDCl_3$): δ = 3.95 (s, 2H), 0.2 (s, 9H).

Compound 19:

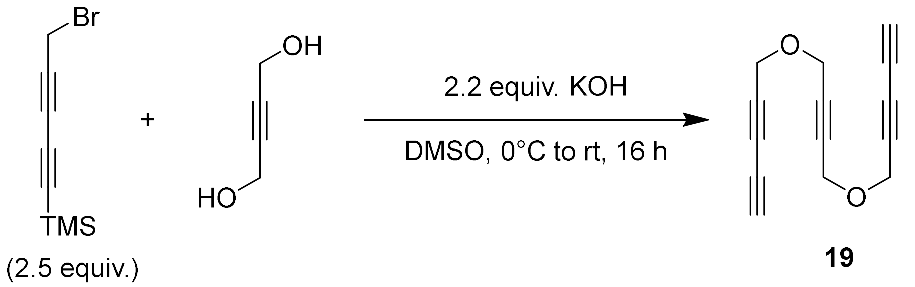

19

In a 100 mL round-bottom flask, 1,4-butynediol (310 mg, 3.6 mmol) was dissolved in 5 mL DMSO, aq. KOH (1.6 mL, 8.0 mmol, 5 M) was added and the reaction mixture was stirred at room temperature for 15 min. **SI-9** (1.942 g, 9.02 mmol) as solution in 5 mL DMSO was added at 0 °C. The reaction was stirred overnight while slowly reaching room temperature. Ice, 50 mL H_2O and aq. NH_4Cl were added to quench the reaction. The aq. phase was extracted with Et_2O (3x50 mL), the combined organic phase was washed with H_2O (2x100 mL), dried with Na_2SO_4 and filtered. The crude product was charged on silica (removal of the solvent was done below 30 °C) and purified by flash column chromatography (gradient elution from *n*-pentane to *n*-pentane:DCM (1:1)). The product **19** was obtained as yellowish oil (181.6 mg, 0.864 mmol, 24%), analysed and instantly transferred to the glovebox and used as a substrate for the cyclization reaction as described in GP1.

Notice: The compound was stored at -40 °C. Complete decomposition to a black solid was observed in vacuo at room temperature overnight. An attempt to store the compound as toluene solution in the glovebox at room temperature resulted in significant decomposition of the pentayne after 24 h.

Oligoynes with terminally unsubstituted alkyne groups can undergo uncontrolled reactions at high temperatures. It is therefore advised to not try to clean the product via distillation, e. g. also no short-path distillation at high temperatures even in vacuo.

^{1}H NMR (300 MHz, $CDCl_3$): δ = 4.31 (s, 8H), 2.19 (s, 2H).
^{13}C{^{1}H} NMR (75 MHz, $CDCl_3$): δ = 82.4, 71.9, 71.2, 68.4, 67.5, 57.2, 57.0.
HRMS(ESI) calculated for $[M+K]^+$: 249.0312; found: 249.0315.

Compound 30:[10]

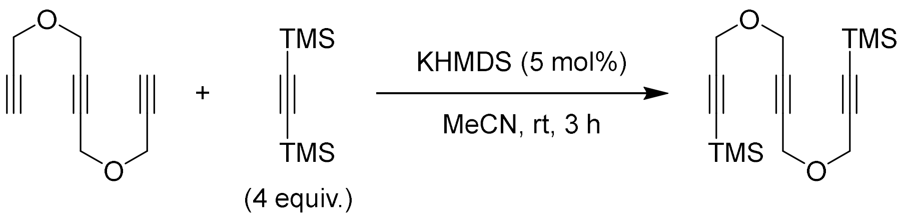

30

The synthesis of triyne **20** was conducted in analogy to a report from Hreczycho and coworkers.[38]
In a 50 mL Schlenk flask, triyne **4** (811mg, 5.00 mmol), bis(trimethylsilyl)acetylene (3.408 g , 20.0 mmol) and KHMDS (50 mg, 0.25 mmol) were dissolved in 30 mL dry MeCN and stirred at room temperature for 3 h. The crude product was directly charged on silica and purified by flash column chromatography with heptane/acetone (v/v 20:1) to obtain **20** as yellow oil (4.4 mmol, 1.349 g, 88%).

^{1}H NMR (300 MHz, $CDCl_3$): δ = 4.29 (s, 4H), 4.24 (s, 4H), 0.18 8s, 18H).

Compound 31

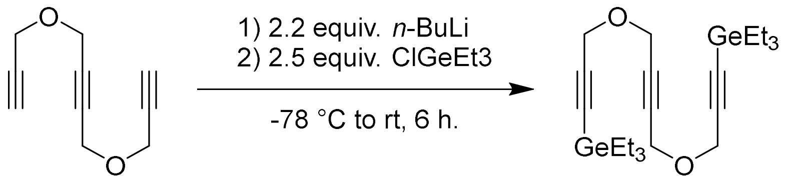

31

A 100 mL Schlenk tube containing 40 mL THF and triyne **4** (324 mg, 2.08 mmol) was cooled to -78 °C and *n*-Buli (4.60 mmol, 2.9 mL) was added with a syringe and stirring was continued for 1 h at that temperature. $ClGeEt_3$ (1.00 g, 5.12 mmol) was added with a syringe and the reaction was stirred for 5 h while reaching room temperature. The reaction was quenched with 10 mL aq. sat. NH_4Cl solution. The aqueous phase was extracted with EtOAc (3x20 mL) and the combined organic extract was dried with Na_2SO_4. The solvent was distilled of and the crude product was subjected to flash column chromatography with heptane/DCM (v/v 5:1) to isolate the product as yellowish oil.

(537 mg, 1.12 mmol, 56%).
^{1}H NMR (300 MHz, $CDCl_3$): δ = 4.61 (s, 4H), 4.26 (s, 4H), 1.13-1.03 (m, 18H), 0.91-0.80 (m, 12H).
^{13}C{^{1}H} NMR (75 MHz, $CDCl_3$): δ = 100.91, 89.91, 82.25, 57.57, 56.54, 9.07, 5.75.
HRMS(ESI) calculated for $[M+H]^+$: 481.1386; found: 481.1383.

Compound 23:

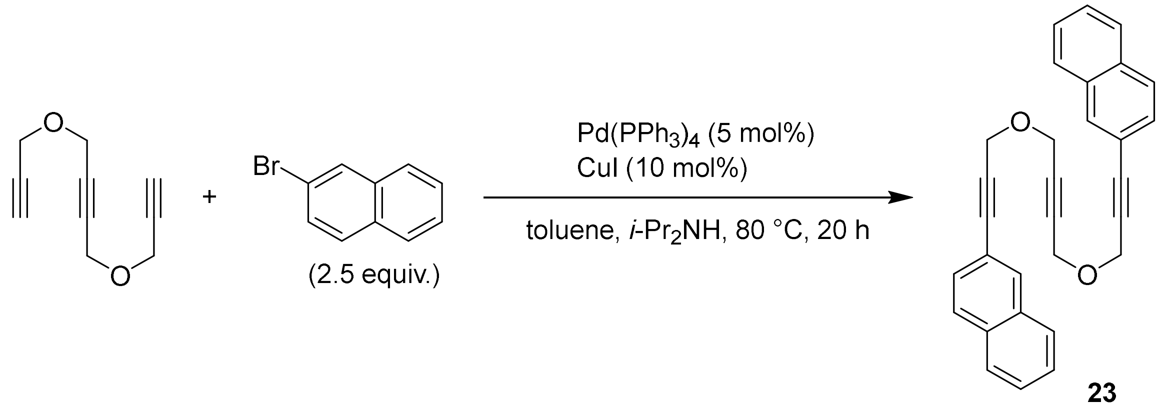

23

In a 100 mL Schlenk flask, $Pd(PPh_3)_4$ (289 mg, 0.25 mmol, 5 mol%), CuI (95 mg, 0.50 mmol, 10 mol%) diisopropylamine (2.10 mL, 14.62 mmol), triyne **4** (811 mg, 5 mmol) and 2-bromonaphthalene (2.588 g, 12.50 mmol) were stirred in 30 mL toluene at 80 °C for 20 h. Sat. aq. NH_4Cl solution was added, the resulting mixture was filtrated over a sintered glass frit covered with a Celite layer and the aqueous phase was extracted with EtOAc (3x50 mL). The combined organic phase was washed with H_2O (100 mL), dried with Na_2SO_4 and filtered. The crude product was charged on silica and purified by flash column chromatography (gradient elution from *n*-heptane to *n*-heptane:DCM (1:1)) to obtain the product **23** as yellow solid (1.409 g, 3.40 mmol, 68%).

^{1}H NMR (300 MHz, $CDCl_3$): δ = 7.99 (s, 2H), 7.84-7.74 (m, 6H), 7.53-7.46 (m, 6H), 4.56 (s, 4H), 4.44 (s, 4H).
^{13}C{^{1}H} NMR (75 MHz, $CDCl_3$): δ = 133.07, 133.01, 131.99, 128.53, 128.15, 127.92, 127.89, 126.95, 126.72, 119.84, 87.37, 84.61, 82.51, 57.66, 57.08.
HRMS(ESI) calculated for $[M+H]^+$: 432.1958; found: 432.1961.

Compound 25:

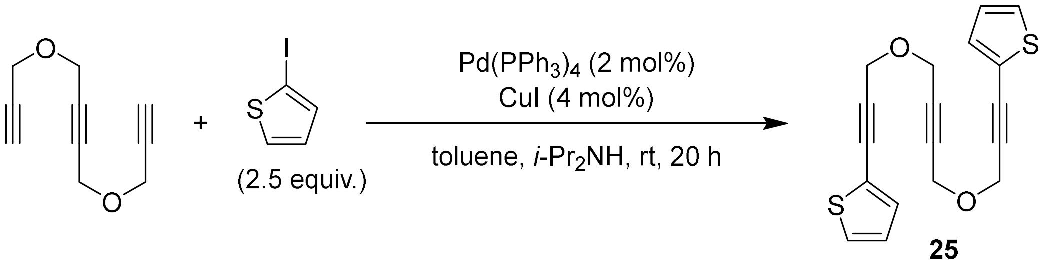

In a 100 mL Schlenk flask, $Pd(PPh_3)_4$ (92 mg, 0.08 mmol, 2 mol%), CuI (30 mg, 0.16 mmol, 4 mol%) diisopropylamine (1.70 mL, 12.00 mmol), triyne **4** (648 mg, 4.00 mmol) and 2-iodothiophene (1.30 mL, 10.21 mmol) were stirred in 50 mL toluene at room temperature for 20 h. Sat. aq. NH_4Cl solution was added and the aqueous phase was extracted with EtOAc (3x50 mL). The combined organic phase was washed with H_2O (100 mL), dried with Na_2SO_4 and filtered. The crude product was charged on silica and purified by flash column chromatography (gradient elution from *n*-heptane to *n*-heptane:DCM (1:1)) to obtain the product **25** as white solid (1.122 g, 3.44 mmol, 86%).

^{1}H NMR (300 MHz, $CDCl_3$): δ = 7.28-7.21 (m, 4H), 6.99-6.94 (m, 2H), 4.49 (s, 4H), 4.37 (s, 4H).
^{13}C{^{1}H} NMR (75 MHz, $CDCl_3$): δ = 132.76, 127.66, 127.08, 122.41, 88.35, 82.42, 80.27, 57.59, 57.03.
HRMS(ESI) calculated for $[M+H]^+$: 327.0508; found: 327.0501.

Compound 26:

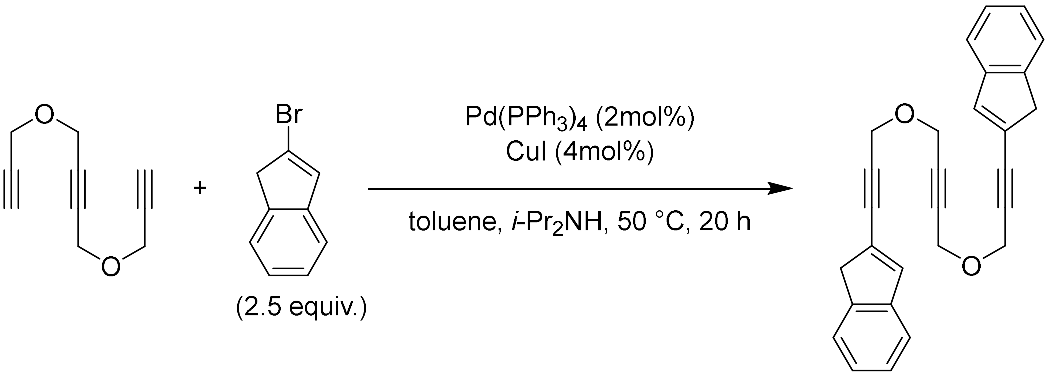

In a 100 mL Schlenk flask, $Pd(PPh_3)_4$ (46 mg, 0.04 mmol, 2 mol%), CuI (15 mg, 0.08 mmol, 4 mol%) diisopropylamine (862 µL, 6.00 mmol), triyne **4** (324 mg, 2.00 mmol) and 2-bromoindene (975 mg, 5,00 mmol) were stirred in 30 mL toluene at 50 °C for 20 h. Sat. aq. NH_4Cl solution was added and the aqueous phase was extracted with EtOAc (3x50 mL). The combined organic phase was washed with H_2O (100 mL), dried with Na_2SO_4 and filtered. The crude product was charged on silica and purified by flash column chromatography (gradient elution from *n*-heptane to *n*-heptane:DCM (1:1)) to obtain the product **26** as white solid (617 mg, 1.58 mmol, 79%).

^{1}H NMR (500 MHz, CD_2Cl_2): δ = 7.47-7.34 (m, 4H), 7.31-7.20 (m, 4H), 7.09 (t, J = 2.1 Hz 2H), 4.48 (s, 4H), 4.36 (s, 4H), 3.54 (s, 4H).
^{13}C{^{1}H} NMR (75 MHz, CD_2Cl_2) = 144.20, 143.35, 138.20, 127.13, 126.95, 126.27, 123.97, 121.81, 89.46, 84.04, 82.65, 57.89, 57.23, 43.03.
HRMS(ESI) calculated for $[M+H]^+$: 391.1693; found: 391.1695.

Compound 27:

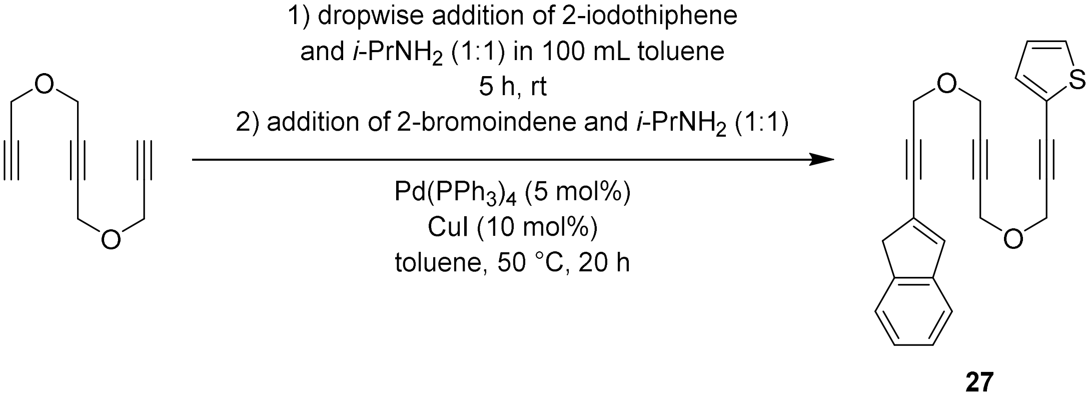

27

In a 250 mL Schlenk flask, $Pd(PPh_3)_4$ (289 mg, 0.25 mmol, 55 mol%), CuI (95 mg, 0.5 mmol, 10 mol%) triyne **4** (324 mg, 2.00 mmol) and 10 mL toluene were stirred at room temperature. To this mixture, 2-iodothiophen (635 µL, 5.00 mmol) and diisopropylamine (711 µL, 5.00 mmol) in 100 mL toluene were added dropwise over 1.5 h. After stirring for 5 h, 2-bromoindene and *i*-Pr_2NH (711 µL, 5 mmol) were added in one portion and the reaction was stirred at 50 °C for 20 h. Sat. aq. NH_4Cl solution was added and the aqueous phase was extracted with EtOAc (3x50 mL). The combined organic phase was washed with H_2O (100 mL), dried with Na_2SO_4 and filtered. The crude product was charged on silica and purified by flash column chromatography (gradient elution from *n*-heptane to *n*-heptane:DCM (1:1)) to obtain the product **27** as yellow oil (950 mg, 2.65 mmol, 53%).

^{1}H NMR (300 MHz, $CDCl_3$): δ = 7.40-7.34 (m, 2H), 7.29-7.18 (m, 4H), 7.07 (s, 1H), 6.98-6.93 (m, 1H), 4.48 (s, 4H), 4.35 (s, 4H), 3.51 (s, 2H).
^{13}C{^{1}H} NMR (75 MHz, $CDCl_3$): δ = 143.82, 142.86, 138.06, 132.67, 127.57, 126.98, 126.80, 126.40, 125.92, 123.61, 122.31, 121.53, 88.89, 88.24, 84.04, 82.38, 82.28, 80.18, 57.65, 57.50, 56.96, 56.94, 42.62.
HRMS(ESI) calculated for $[M+NH_4]^+$: 376.1366; found: 376.1368.

Compound SI-10:

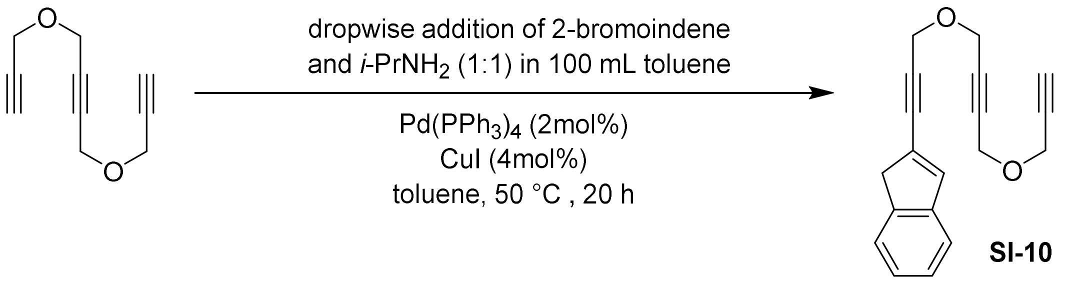

SI-10

Compound **SI-10** was prepared and isolated in a similar manner as described above for the synthesis of compound **27** with 2-bromoindene (976 mg, 5.00 mmol) instead of 2-iodothiophene and stirring at 50 °C for 20 h. Product **SI-10** was obtained as yellow oil (580 mg, 2.10 mmol, 42%).

^{1}H NMR (300 MHz, $CDCl_3$): δ = 7.45-7.35 (m, 2H), 7.33-7.20 (m, 2H), 7.10 (s, 1H), 4.50 (d, J = .4 Hz, 2H), 4.41-4.24 (m, 6H), 3.53 (s, 2H) 2.48 (t, J = 2.40 Hz, 1H).
^{13}C{^{1}H} NMR (75 MHz, $CDCl_3$): δ = 143.89, 142.94, 138.14, 126.88, 126.47, 125.99, 123.68, 121.60, 88.97, 84.14, 82.45, 82.21, 78.95, 75.20, 57.71, 56.98, 56.87, 56.82, 56.64, 56.63, 42.69.
HRMS(ESI) calculated for $[M+H]^+$: 277.1223; found: 277.1224.

Compound 28:

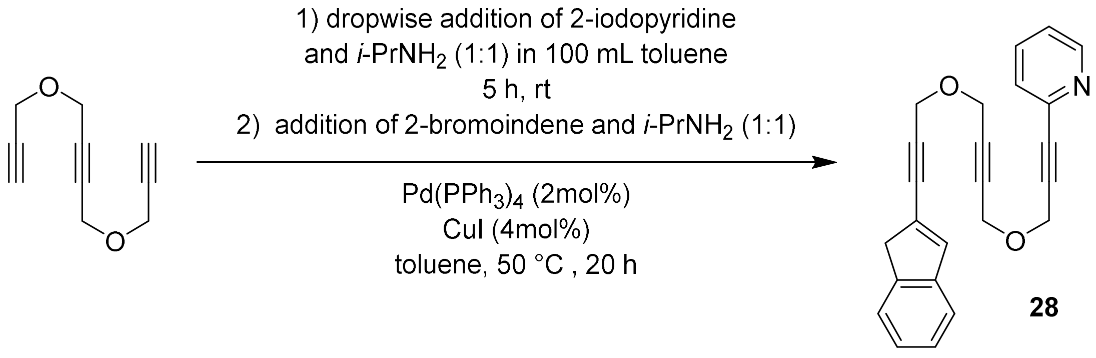

Compound **28** was prepared as described above for the synthesis of compound **26** with 2-iodopyridine (628 µL, 5.00 mmol) instead of 2-iodothiophene. Product **28** was obtained after flash column chromatography (gradient elution from DCM to DCM:acetone (10:1)) as brown solid (1.201 g, 3.40 mmol, 68%).

^{1}H NMR (300 MHz, $CDCl_3$): δ = 8.49-8.47 (m, 1H), 7.61-7.46 (m, 1H), 7.37-7.25 (m, 3H), 7.22-7.09 (m, 3H), 6.99 (s, 1H), 4.42 (s, 2H), 4.39 (s, 2H), 4.35-4.22 (m, 4H), 3.42 (s, 2H).
^{13}C{^{1}H} NMR (75 MHz, $CDCl_3$): δ = 150.14, 143.91, 142.95, 142.71, 138.12, 136.31, 127.32, 126.87, 126.51, 125.98, 123.68, 123.29, 121.60, 88.98, 86.19, 84.41, 84.10, 82.55, 82.27, 57.71, 57.25, 57.16, 56.99, 42.70.
HRMS(ESI) calculated for $[M+H]^+$: 354.1489; found: 354,1489.

Compound 29:

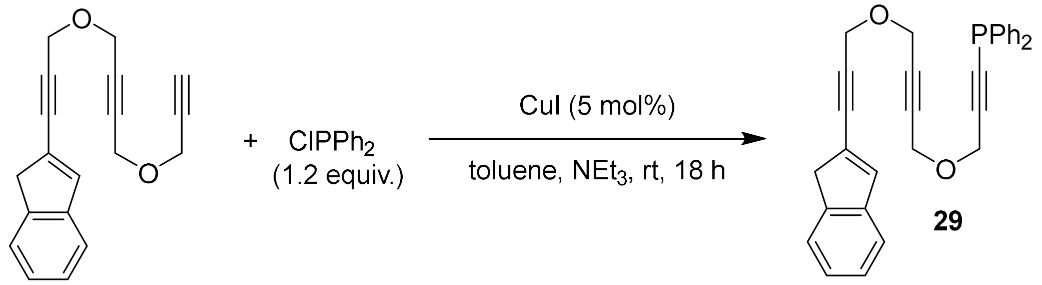

In a 100 mL Schlenk flask, CuI (19 mg, 0.1 mmol, 5 mol%), compound **SI-10** (553 mg, 2.00 mmol), triethylamine (333 µL, 2.40 mmol) and chlorodiphenylphosphine (538 µL. 3.00 mmol) were stirred at room temperature for 18 h. Sat. aq. NH_4Cl solution was added, and the aqueous phase was extracted with EtOAc (3x50 mL). The combined organic phase was washed with H_2O (100 mL), dried with Na_2SO_4 and filtered. The crude product was charged on silica and purified by flash column chromatography (gradient elution from *n*-heptane to *n*-heptane:DCM (1:1)) to obtain product **29** as yellow oil (267 mg, 0.58 mmol, 29%).

^{1}H NMR (300 MHz, $CDCl_3$): δ = 7.69-7.56 (m, 2H), 7.48-7.22 (m, 12H), 7.13 (s, 1H), 4.56-4.32, (m, 8H), 3.54 (s, 2H).
^{13}C{^{1}H} NMR (75 MHz, $CDCl_3$): δ = 143.92, 142.97, 138.16, 135.80 (d, J = 6.5 Hz), 132.71 (d, J = 20.2 Hz), 129.27, 128.78 (d, J = 7.9 Hz), 126.92, 126.51, 126.02, 123.71, 121.63, 103.70 (d, J = 1.6 Hz), 84.62, 84.47, 84.14, 82.45, 82.40 (d, J = 25.2 Hz), 57.75, 57.72, 57.06, 57.00, 53.56, 42.72.
^{31}P{^{1}H} NMR (75 MHz, $CDCl_3$): δ = -34.06.
HRMS(ESI) calculated for $[M+H]^+$: 361.1665; found: 361.1662.

Compound SI-16:

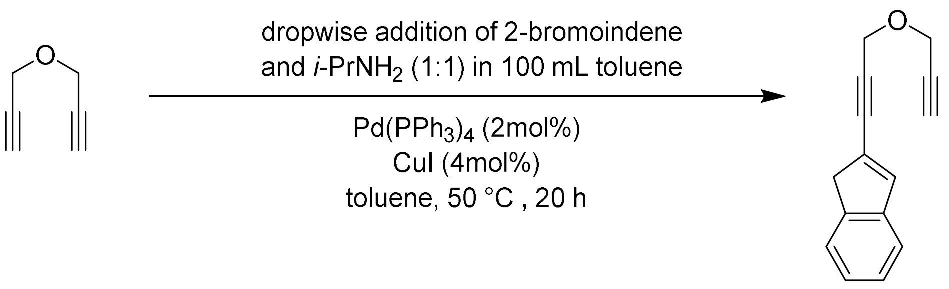

SI-16

SI-16 was prepared and isolated as described above for the synthesis of compound **SI-10** with dipropargylether (628 µL, 5.00 mmol) instead of triyne **4**. The product **SI-16** was obtained as yellow oil (158 mg, 0.76 mmol, 38%).

^{1}H NMR (300 MHz, $CDCl_3$): δ = 7.29-7.22, 7.17-7.06, 6.96 (s, 1H), 4.36 (s, 2H), 4.18 (d, 2H), 3.39 (s, 2H), 2.35 (t, 1H).
^{13}C{^{1}H} NMR (75 MHz, $CDCl_3$): δ = 143.93, 142.98, 138.16, 126.92, 126.52, 126.04, 123.72, 121.64, 88.90, 84.13, 79.11, 75.18, 57.68, 56.76, 42.73.
HRMS(ESI) calculated for $[M+NH_4]^+$: 226.1226; found: 463.1221.

Compound 35:

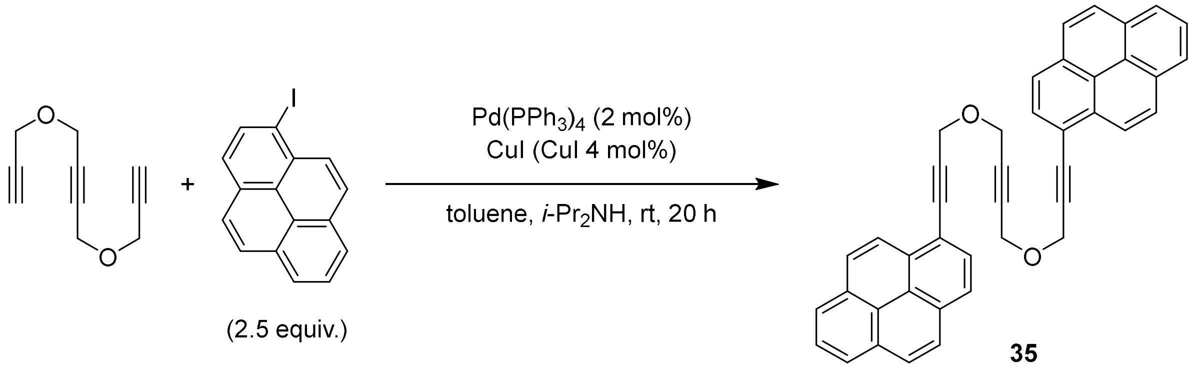

35

In a 100 mL Schlenk flask, $Pd(PPh_3)_4$ (46 mg, 0.04 mmol, 2 mol%), CuI (15 mg, 0.08 mmol, 4 mol%) diisopropylamine (862 µL, 6.00 mmol), triyne **4** (324 mg, 2.00 mmol) and **SI-10** (1.641 g, 5.00 mmol) were stirred in 30 mL toluene at room temperature for 20 h. Sat. aq. NH_4Cl solution was added and the aqueous phase was extracted with EtOAc (3x50 mL). The combined organic phase was washed with H_2O (100 mL), dried with Na_2SO_4 and filtered. The crude product was charged on silica and purified by flash column chromatography (gradient elution from pure *n*-heptane to pure DCM) to obtain the product **35** as yellow solid (597 mg, 1.70 mmol, 85%).

^{1}H NMR (500 MHz, $CDCl_3$): δ = 8.53 (d, J = 9.3 Hz 2H), 8.17-7.96 (m, 16H), 4.78 (s, 4H), 4.59 (s, 4H).
^{13}C{^{1}H} NMR (126 MHz, $CDCl_3$): δ = 132.26, 131.56, 131.27, 131.08, 129.99, 128.58, 128.40, 127.27, 126.33, 125.79, 125.73, 125.43, 124.52, 124.47, 124.32, 116.95, 89.86, 86.16, 82.71, 57.97, 57.23.
HRMS(ESI) calculated for $[M+H]^+$: 463.2006; found: 463.2005.

Compound 36:

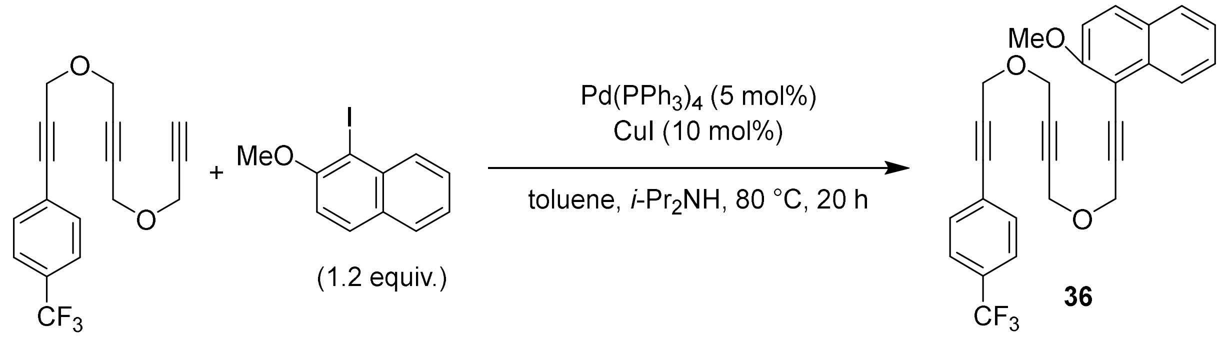

36

In a 100 mL Schlenk flask, $Pd(PPh_3)_4$ (46 mg, 0.05 mmol, 5 mol%), CuI (19 mg, 0.10 mmol, 10 mol%) diisopropylamine (287 µL, 2.00 mmol), **SI-11** (306 mg, 1.00 mmol) and 1-iodo-2-methoxynaphthalene (341 mg, 1.20 mmol) were stirred in 20 mL toluene at 80 °C for 20 h. Sat. aq. NH_4Cl solution was added and the aqueous phase was extracted with EtOAc (3x50 mL). The combined organic phase was washed with H_2O (100 mL), dried with Na_2SO_4 and filtered. The crude product was charged on silica and purified by flash column chromatography (gradient elution from pure *n*-heptane to pure DCM) to obtain the product **36** as yellow solid (444 mg, 0.96 mmol, 48%).

^{1}H NMR (500 MHz, $CDCl_3$): δ = 8.24 (d, *J* = 8.6 Hz, 1H), 7.83 (d, *J* = 9.1 Hz, 1H), 7.78 (d, *J* = 8.3 Hz, 1H), 7.57-7.53 (m, 4H), 7.41-7.35 (m, 1H), 7.25 (d, *J* = 9.1 Hz 1H), 4.71 (s, 2H), 4.51 (s, 4H), 4.40 (t, *J* = 1.8 Hz, 1H), 2H), 4.02 (s, 3H).
^{19}F {^{1}H} NMR (471 MHz, $CDCl_3$) δ = -62.71.
HRMS(ESI) calculated for $[M+H]^+$: 463.1516; found: 463.1516.

Compound 58:

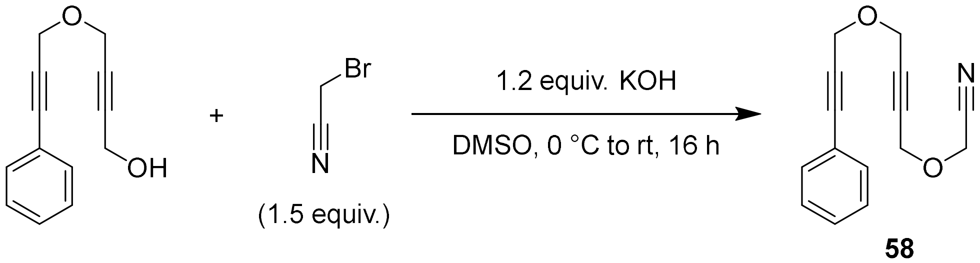

58

In a 50 mL round-bottom flask, to **SI-12** (400 mg, 2 mmol) in 5 mL DMSO was added aqueous KOH (2 mL, 2.40 mmol, 5 M) at room temperature and the resulting reaction mixture was stirred for 15 min. Bromoacetonitrile (279 µL, 4 mmol) as solution in 2 mL DMSO was added dropwise with a syringe and the reaction was stirred for 16 h while reaching room temperature. An aqueous saturated. NH_4Cl solution was added to quench the reaction. The aq. phase was extracted with EtOAc (3x20 mL), the combined organic phase was washed with H_2O (2x50 mL), dried with Na_2SO_4 and filtered. The crude product was charged on silica and purified by flash column chromatography (gradient elution from *n*-heptane to *n*-heptane:DCM (1:1)). The product **58** was obtained as yellowish oil (124 mg, 0.52 mmol, 24%).

^{1}H NMR (500 MHz, $CDCl_3$): δ = 7.47-7.43 (m, 2H), 7.36-7.30 (m, 3H), 4.48 (s, 2H), 4.39 (s, 4H), 4.37 (s, 2H).
^{13}C{^{1}H} NMR (126 MHz, $CDCl_3$): δ = 131.94, 128.82, 128.49, 122.45, 115.53, 87.24, 84.58, 84.01, 80.25, 58.49, 57.79, 56.82, 54.29.
HRMS(ESI) calculated for $[M+H]^+$: 240.1019; found: 240.1019.

Compound 60:

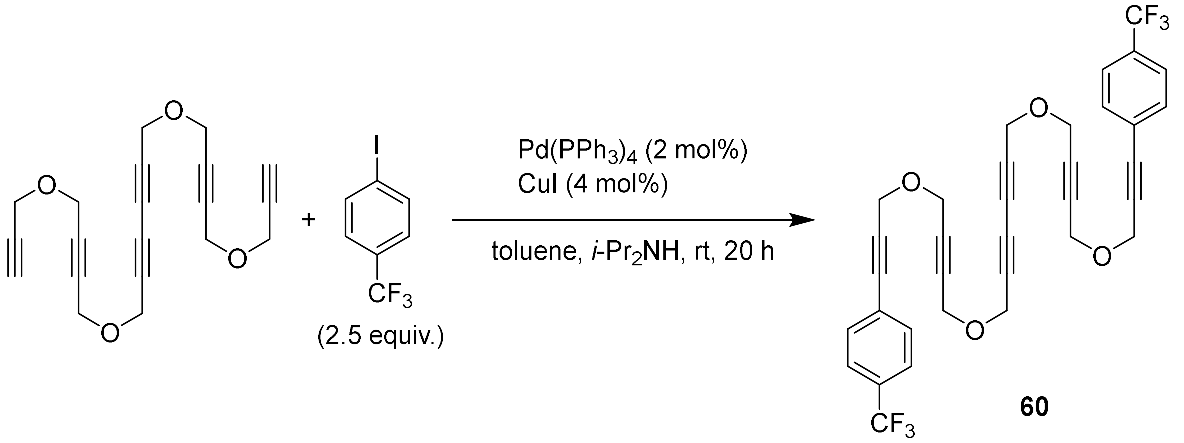

60

In a 100 mL Schlenk flask, $Pd(PPh_3)_4$ (46 mg, 0.04 mmol, 2 mol%), CuI (15 mg, 0.08 mmol, 4 mol%) diisopropylamine (862 µL, 6.00 mmol), hexayne **62** (645 mg, 2.00 mmol) and 4-iodobenzotrifluoride (1.360 g, 5 mmol) were stirred in toluene at room temperature for 20 h. Sat. aq. NH_4Cl solution was added and the aqueous phase was extracted with EtOAc (3x 20 mL). The combined organic phase was washed with H_2O (100 mL), dried with Na_2SO_4 and filtered. The crude product was charged on silica and purified by flash column chromatography (gradient elution from *n*-heptane to *n*-heptane:DCM (1:1)). to obtain the product **60** as yellow oil (928 mg, 1.52 mmol, 76%).

^{1}H NMR (500 MHz, $CDCl_3$): δ = 7.58-7.51 (m, 8H), 4.48 (s, 4H), 4.36 (t, J = 1.7 Hz, 4H), 4.33 (s, 4H), 4.31 (t, J = 1.7 Hz, 4H).
^{13}C{^{1}H} NMR (126 MHz, $CDCl_3$) δ 132.10, 130.38 (q, J = 32.6 Hz), 126.28 125.34 (q, J = 3.7 Hz, 4H), 123.92 (q, J = 272.5 Hz), 86.80, 85.53, 82.57, 82.08, 74.84, 70.90, 57.35, 57.08, 57.06.
^{19}F {^{1}H} NMR (471 MHz, $CDCl_3$) δ = -62.89.
HRMS(ESI) calculated for $[M+Na]^+$: 633.1471; found: 633.1469.

Compound 62:

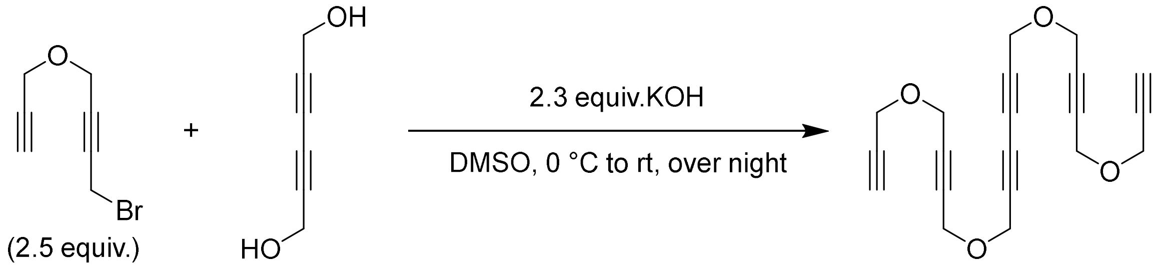

62

In a 50 mL round-bottom flask, compound **SI-6** (330 mg, 3.00 mmol) was dissolved in 10 mL DMSO, aq. KOH (1.40 mL, 7.00 mmol, 5 M) was added and the reaction mixture was stirred at room temperature for 15 min. **SI-2** (1.403 g, 7.50 mmol) as solution in 2 mL DMSO was added at 0 °C. The reaction was stirred overnight while slowly reaching room temperature. Ice, 50 mL H_2O and aq. NH_4Cl were added to quench the reaction. The aq. phase was extracted with Et_2O (3x50 mL), the combined organic phase was washed with H_2O (2x100 mL), dried with Na_2SO_4 and filtered. The crude product was charged on silica and purified by flash column chromatography (gradient elution from *n*-heptane to *n*-heptane:DCM (1:1)). The product **62** was obtained as yellowish oil which solidified after standing for two days in the glovebox (832 mg, 2.58 mmol, 86%).
Oligoynes with conjugated and terminal unsubstituted alkynes are sensitive to heating and should only be handled at temperatures below 50 °C and never heated up higher for distillation or condensation.

^{1}H NMR (500 MHz, $CDCl_3$): δ = 4.34-4.24 (m, 16H), 2.46 (t, J = 2.4 Hz, 2H).

$^{13}C\{^1H\}$ NMR (126 MHz, $CDCl_3$): δ = 82.57, 81.95, 78.88, 75.25, 74.82, 70.93, 57.09, 57.07, 56.82, 56.68.
HRMS(ESI) calculated for $[M+H]^-$: 321.1132; found: 321.1131.

Compound 64:

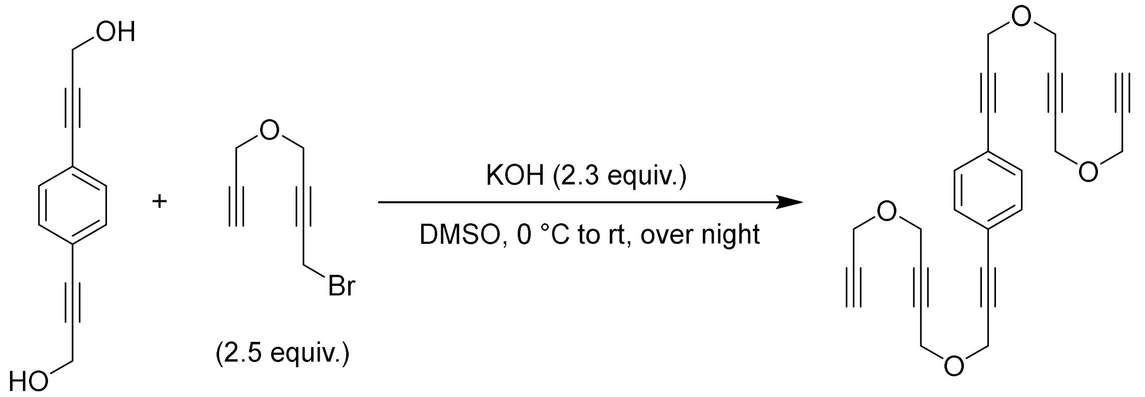

64

In a 50 mL round-bottom flask, **SI-13** (559 mg, 3.00 mmol) was dissolved in 10 mL DMSO, aq. KOH (1.40 mL, 7.00 mmol, 5 M) was added and the reaction mixture was stirred at room temperature for 15 min. **SI-2** (1.403 g, 7.50 mmol) as solution in 2 mL DMSO was added at 0 °C. The reaction was stirred overnight while slowly reaching room temperature. Ice, 50 mL H_2O and aq. NH_4Cl were added to quench the reaction. The aq. phase was extracted with Et_2O (3x50 mL), the combined organic phase was washed with H_2O (2x100 mL), dried with Na_2SO_4 and filtered. The crude product was charged on silica and purified by flash column chromatography (gradient elution from *n*-heptane to *n*-heptane:DCM (1:1)). The product **64** was obtained as yellowish oil (753 mg, 1.89 mmol, 86%).

^1H NMR (300 MHz, $CDCl_3$): δ = 7.38 (s, 4H), 4.47 (s, 4H), 4.35 (t, *J* = 1.7 Hz, 4H), 4.32 (t, *J* = 1.7 Hz, 4H), 4.25 (t, *J* = 2.4 Hz, 4H), 2.45 (t, *J* = 2.4 Hz, 2H).
$^{13}C\{^1H\}$ NMR (75 MHz, $CDCl_3$): δ = 131.80, 122.73, 86.42, 86.21, 82.36, 82.25, 78.91, 75.22, 57.47, 57.01, 56.87, 56.67
HRMS(ESI) calculated for $[M+H]^+$: 399.1591; found: 399.1595.

3 NMR Spectroscopic Data

3.1 NMR spectra of Mn7

^{1}H NMR spectrum of compound **Mn7**

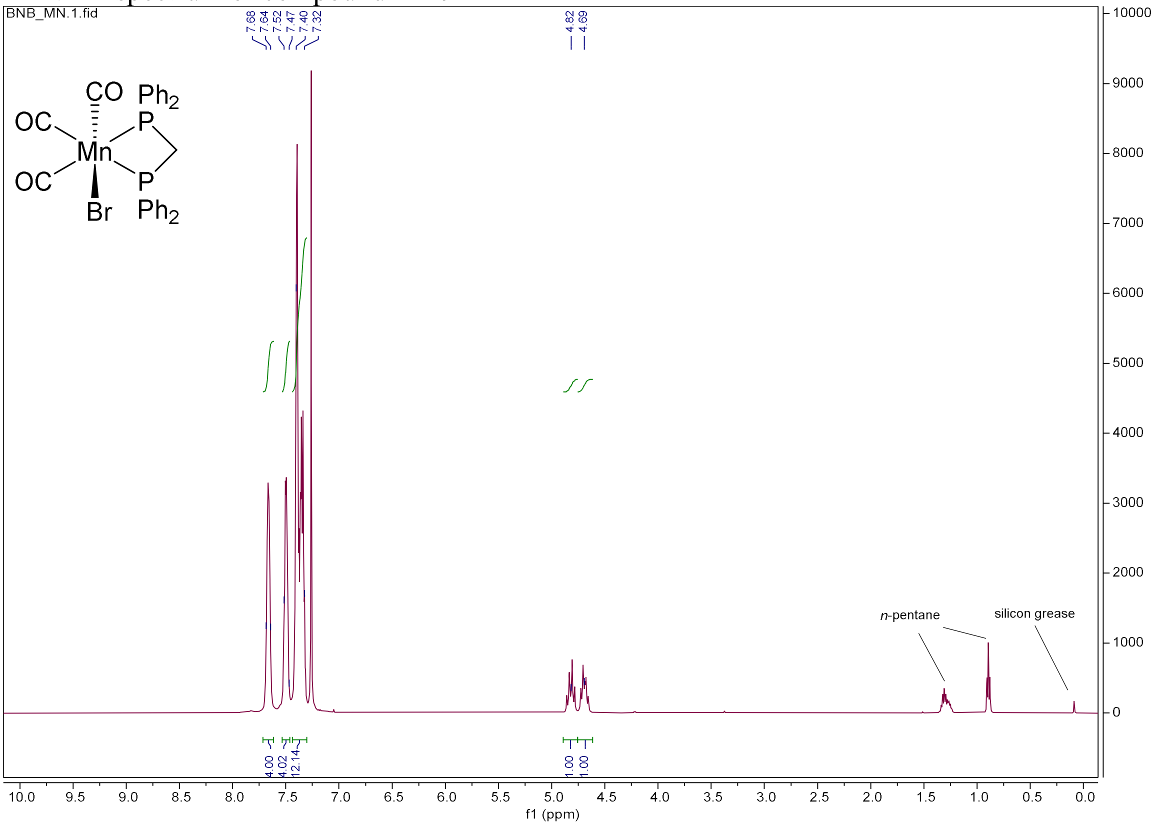

^{13}C{^{1}H} NMR spectrum of **Mn7**

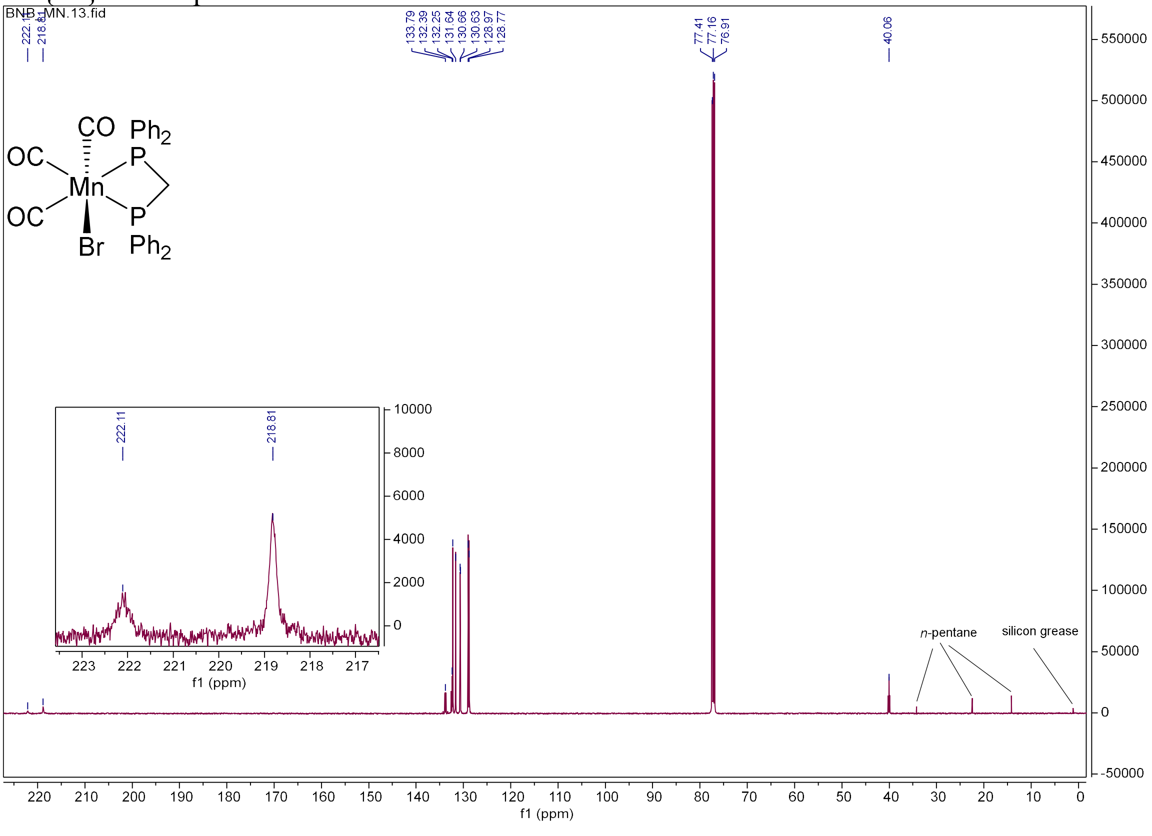

$^{31}P\{^1H\}$ NMR spectrum of compound **Mn7**

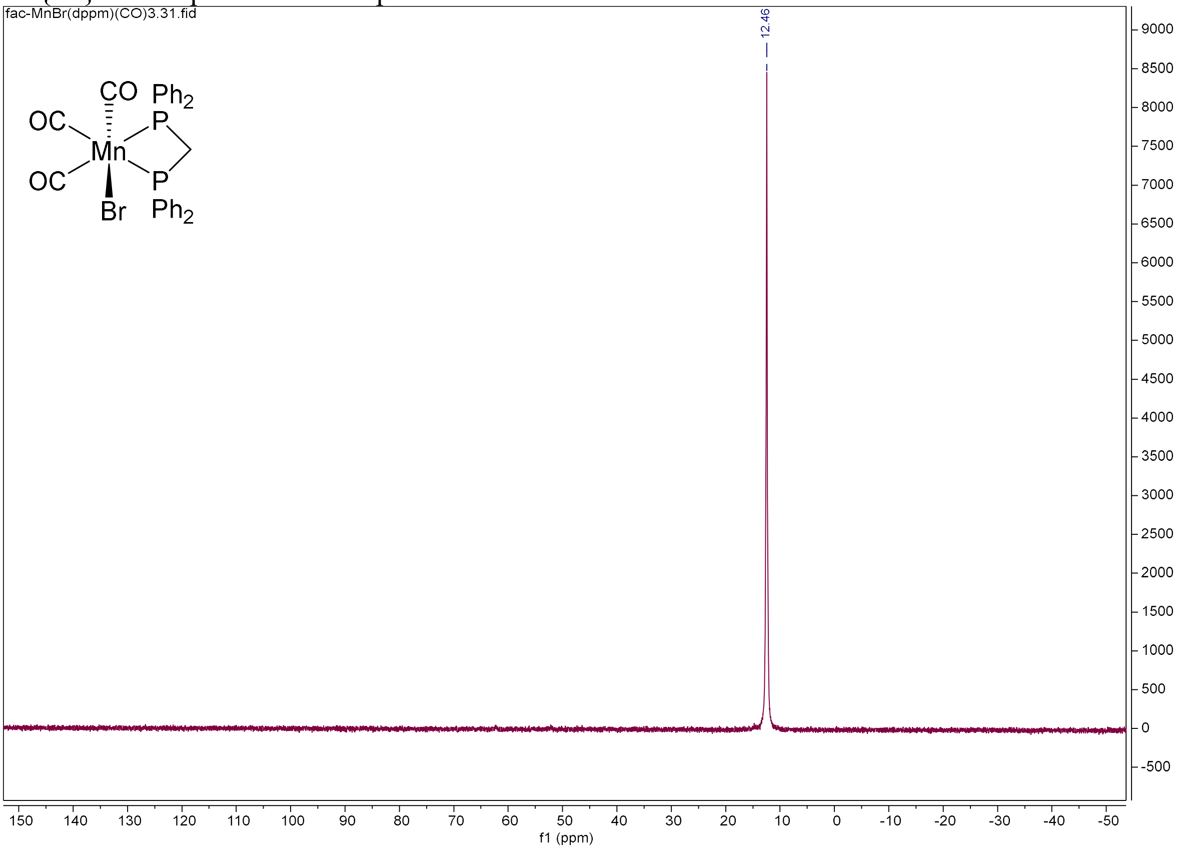

3.2 NMR spectra of cyclotrimerization products

^1H NMR spectrum of compound **5**

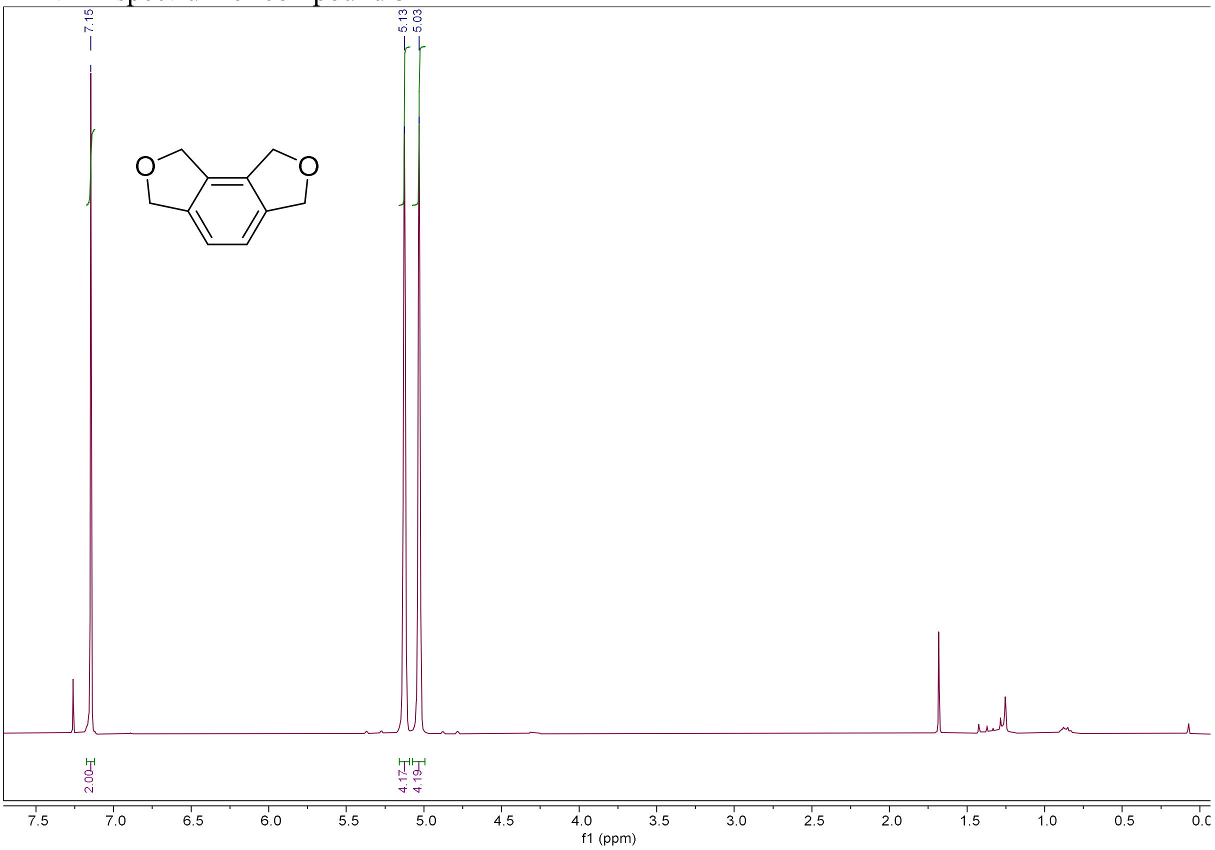

^{1}H NMR spectrum of compound **11**

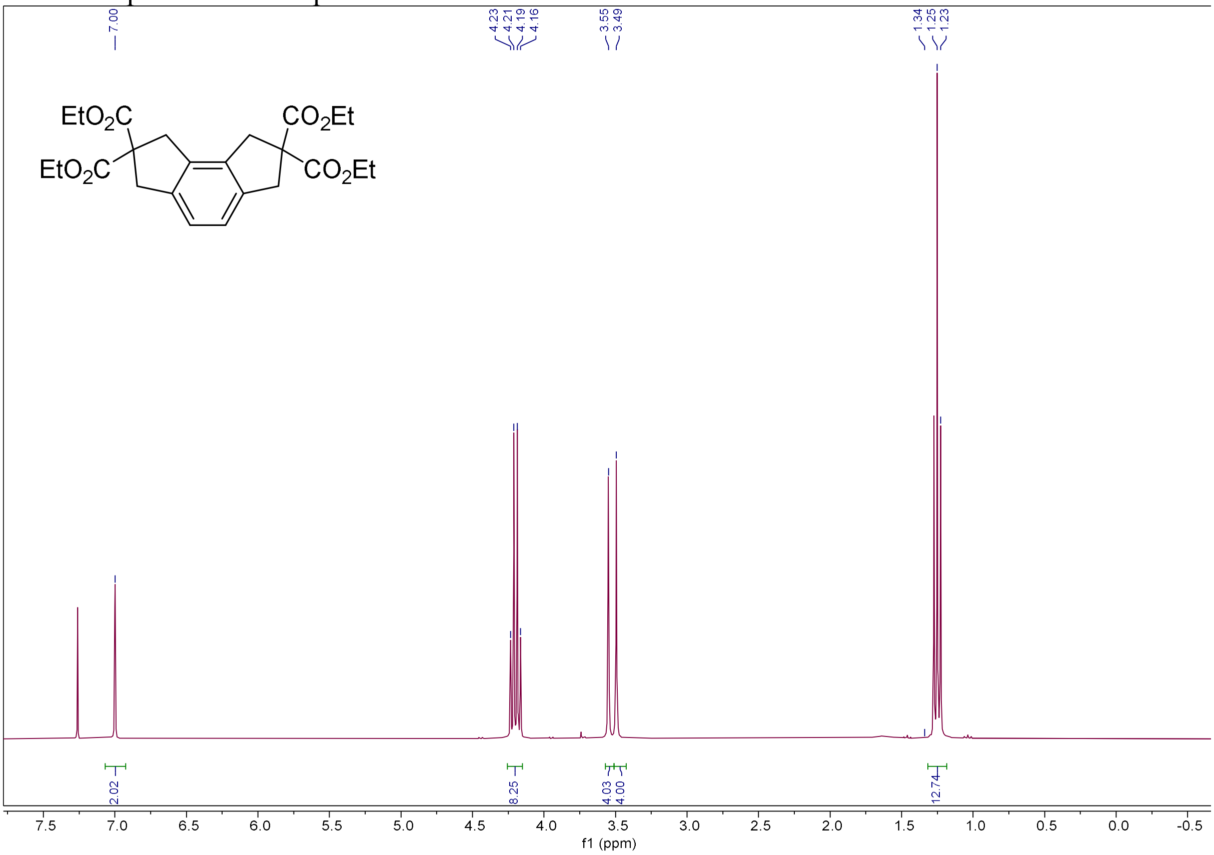

^{1}H NMR spectrum of compound **12**

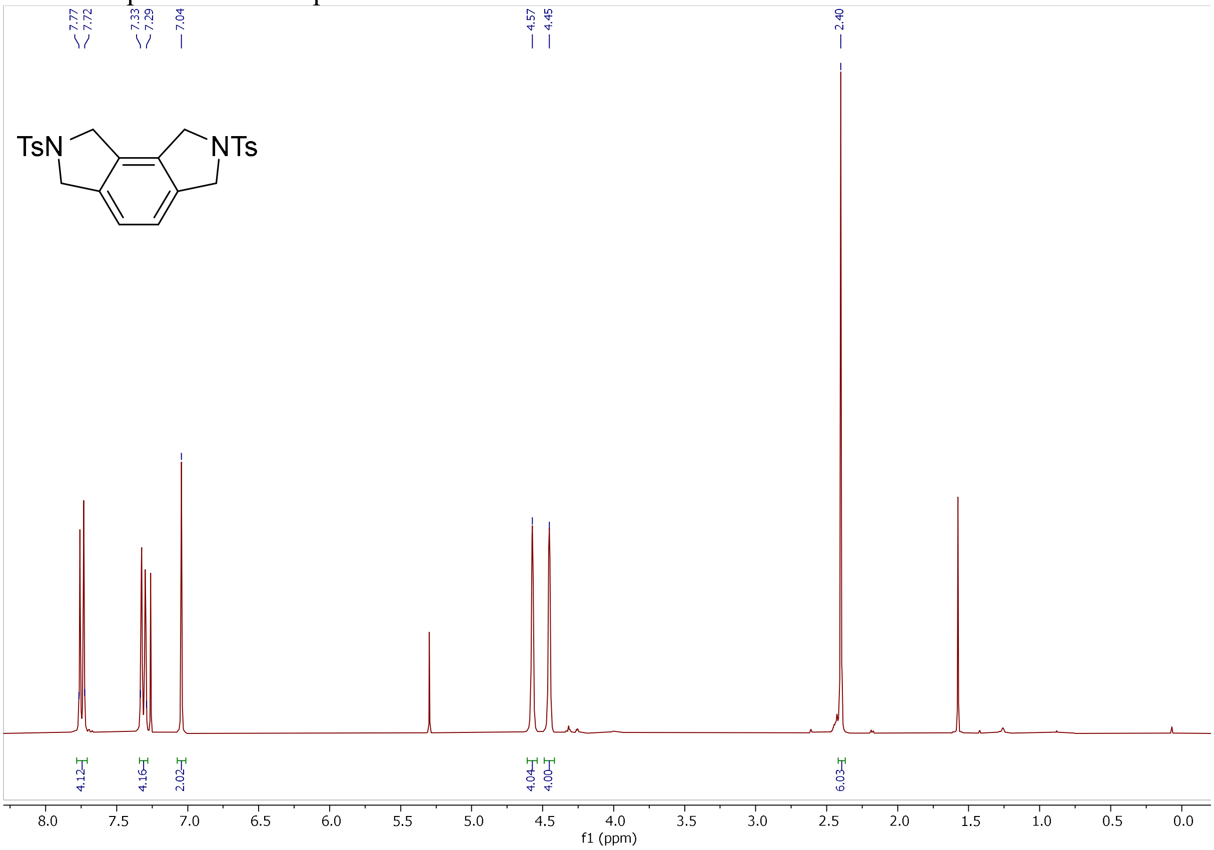

^{1}H NMR spectrum of compound **13**

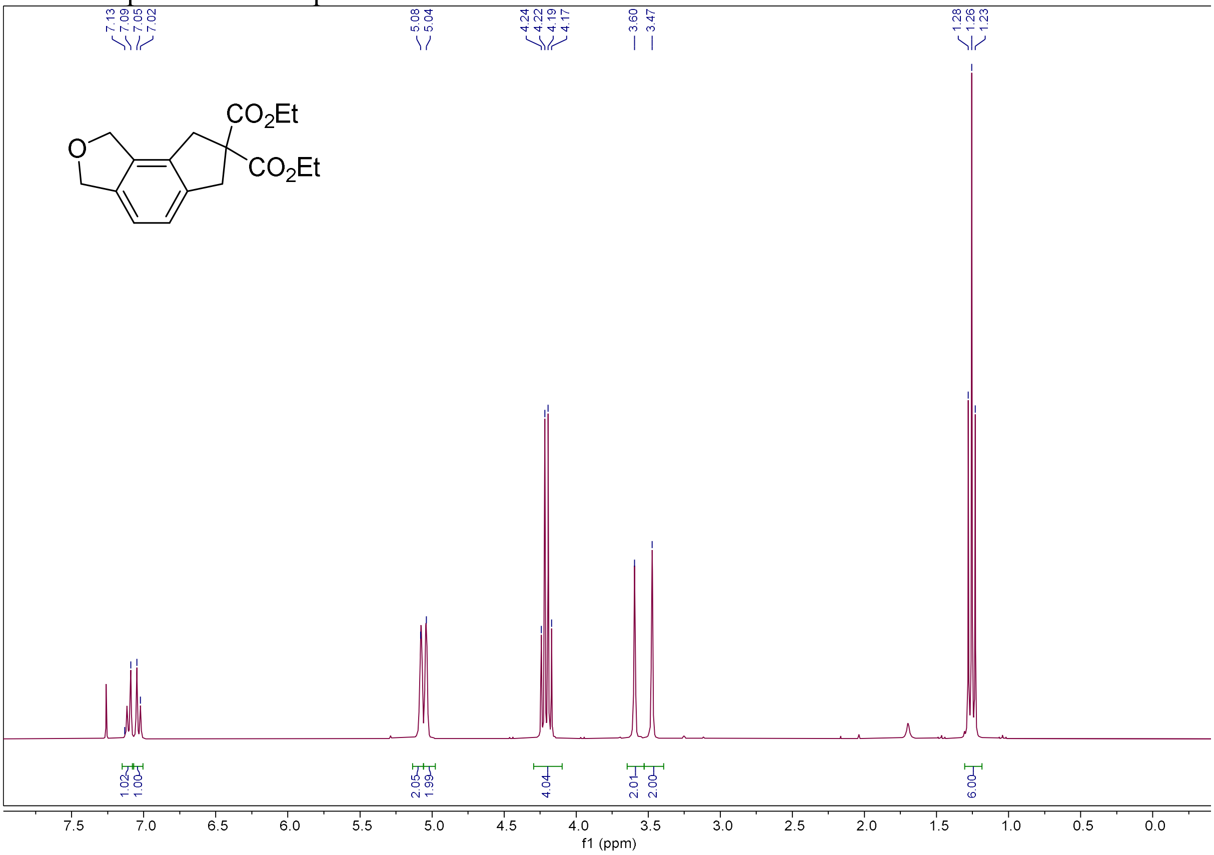

^{1}H NMR spectrum of compound **14**

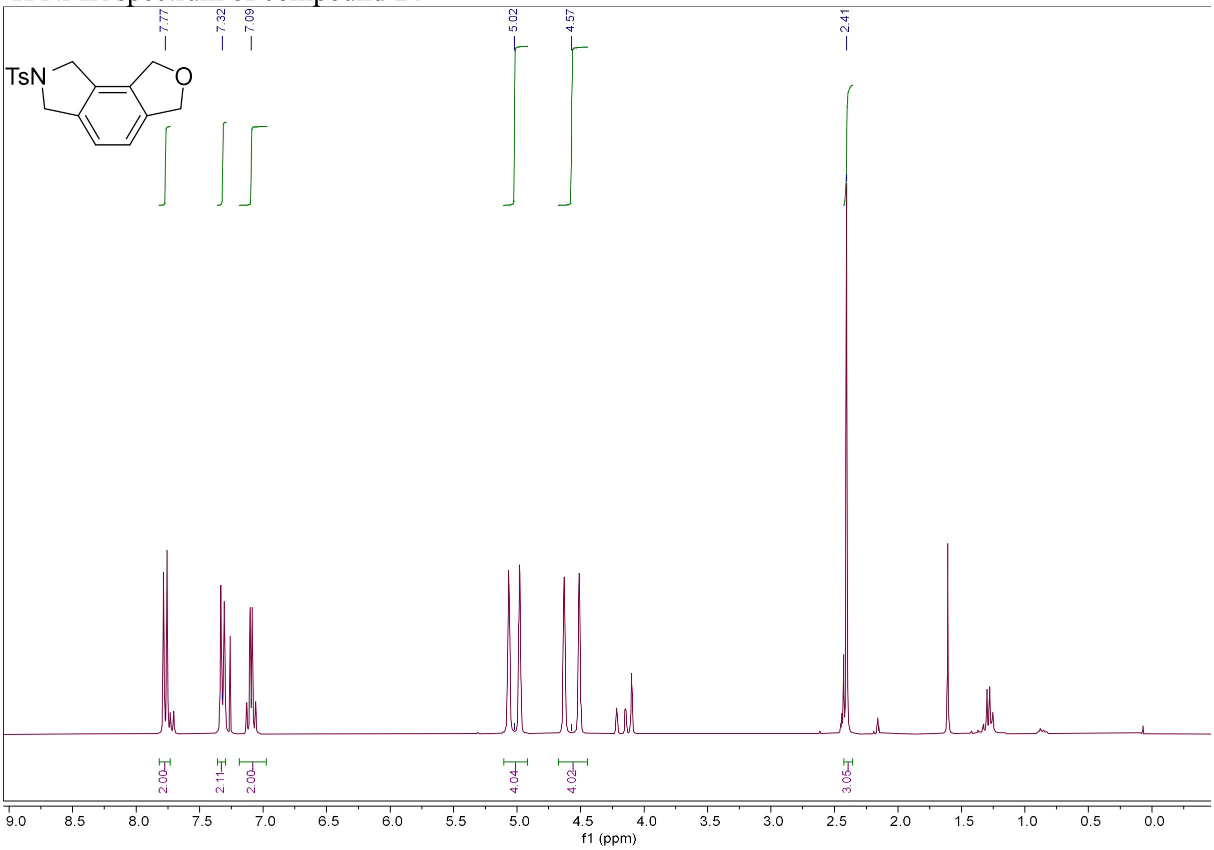

^{1}H NMR spectrum of compound **15**

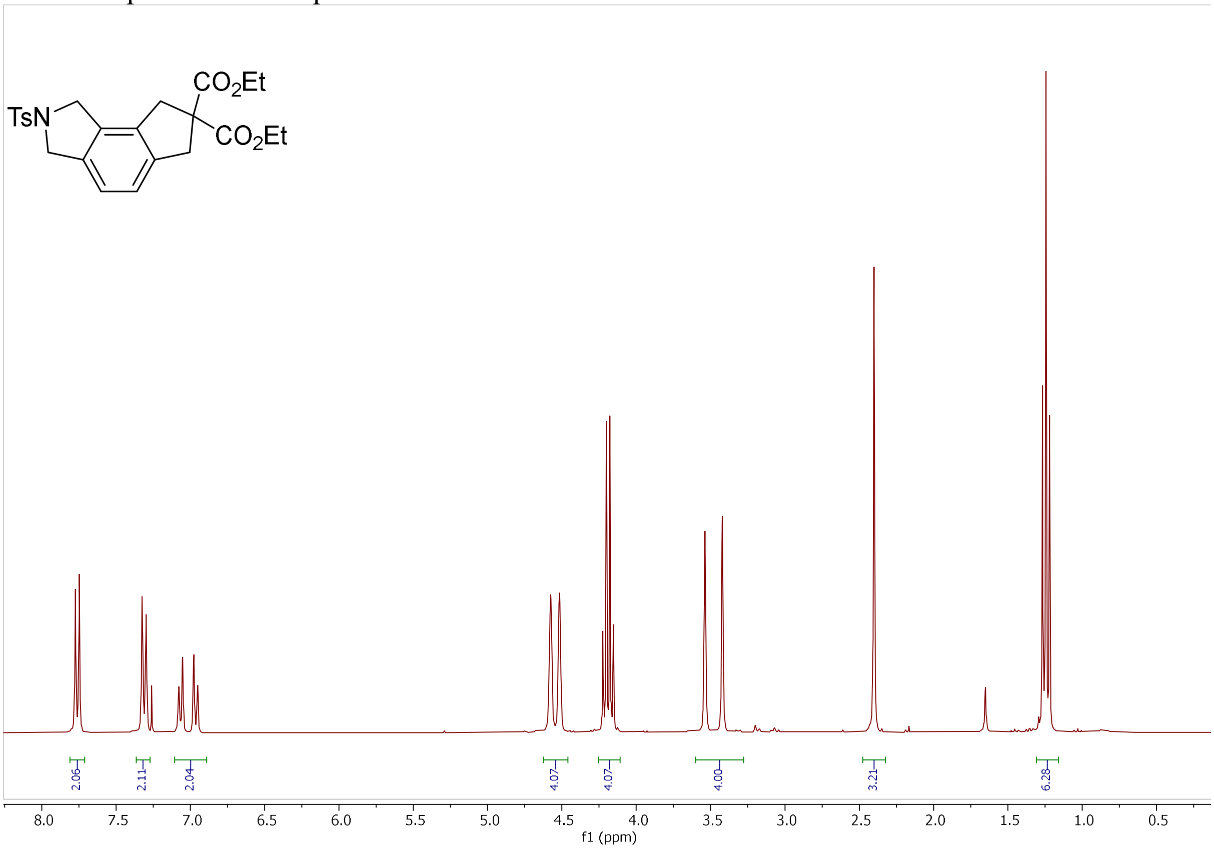

$^{13}C\{^1H\}$ NMR spectrum of **15**

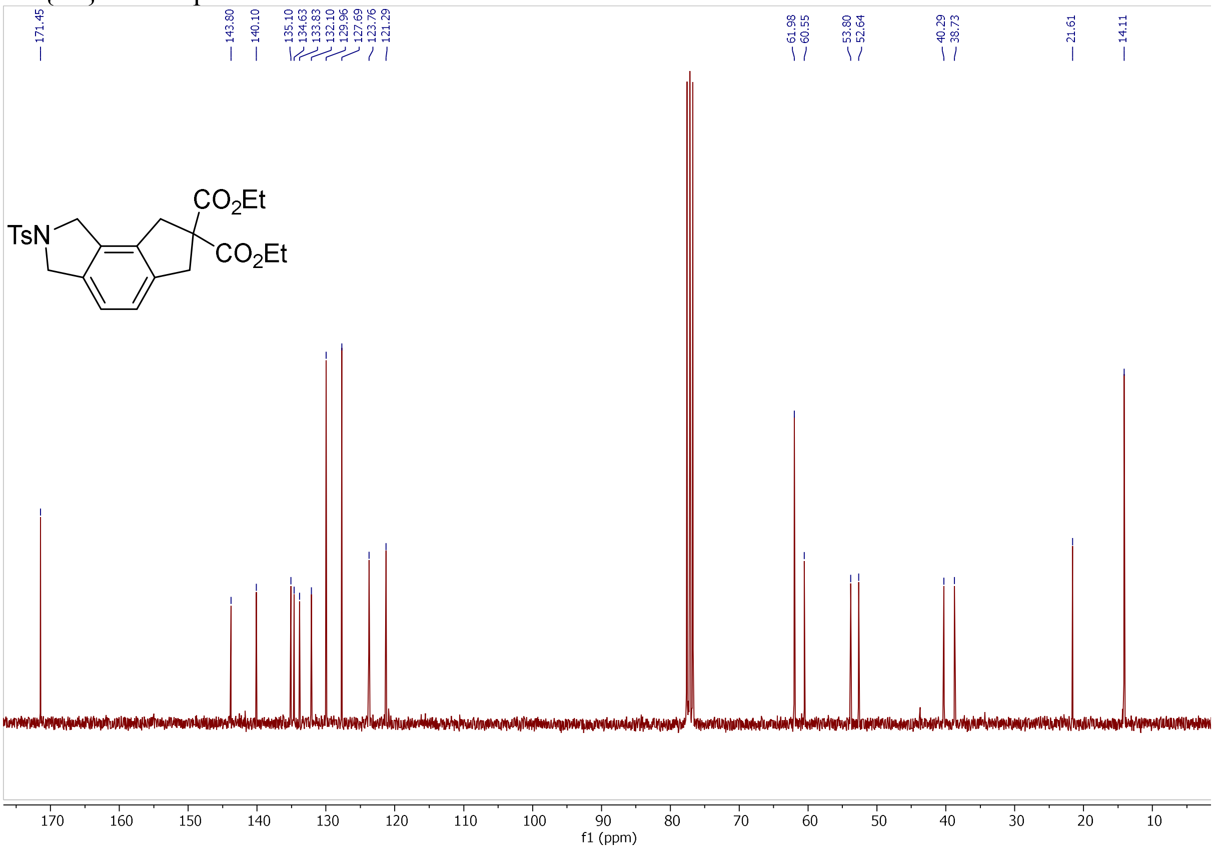

^{1}H NMR spectrum of compound **37**

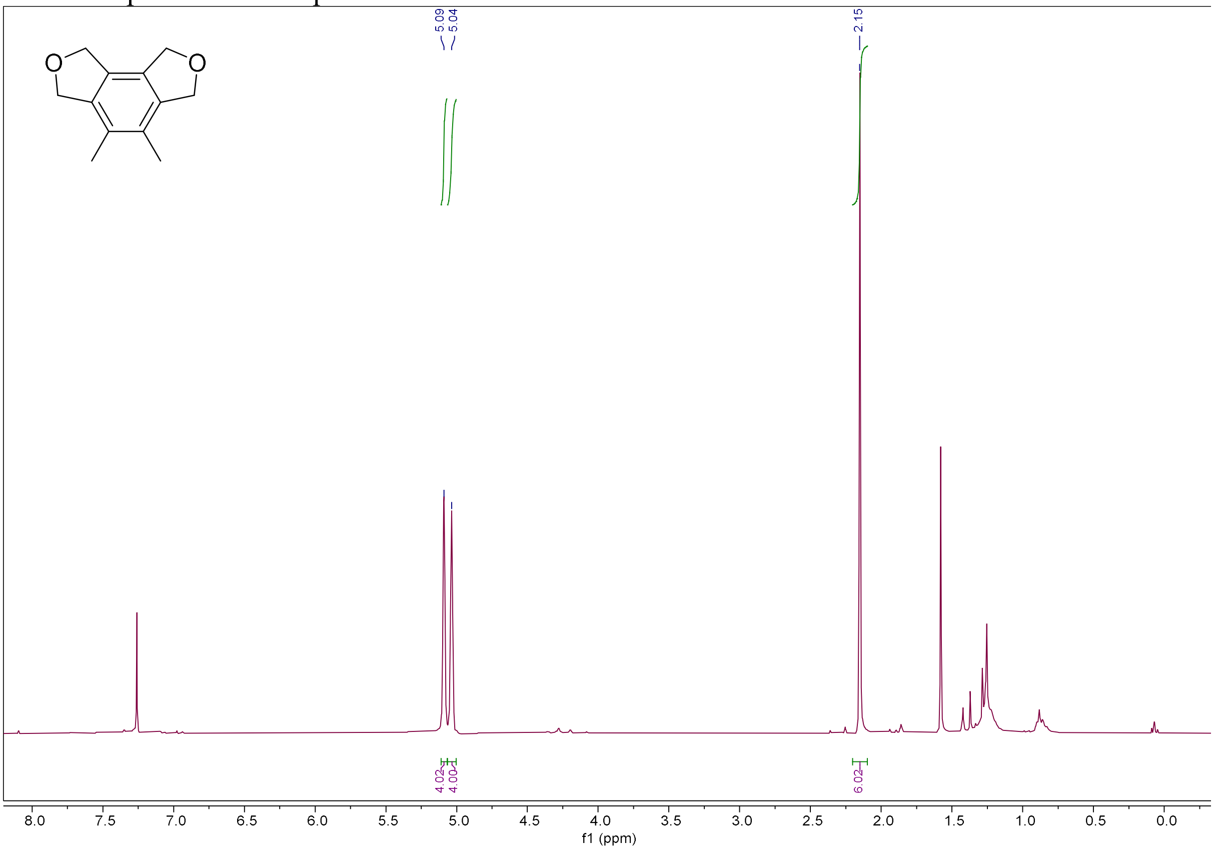

^{1}H NMR spectrum of compound **38**

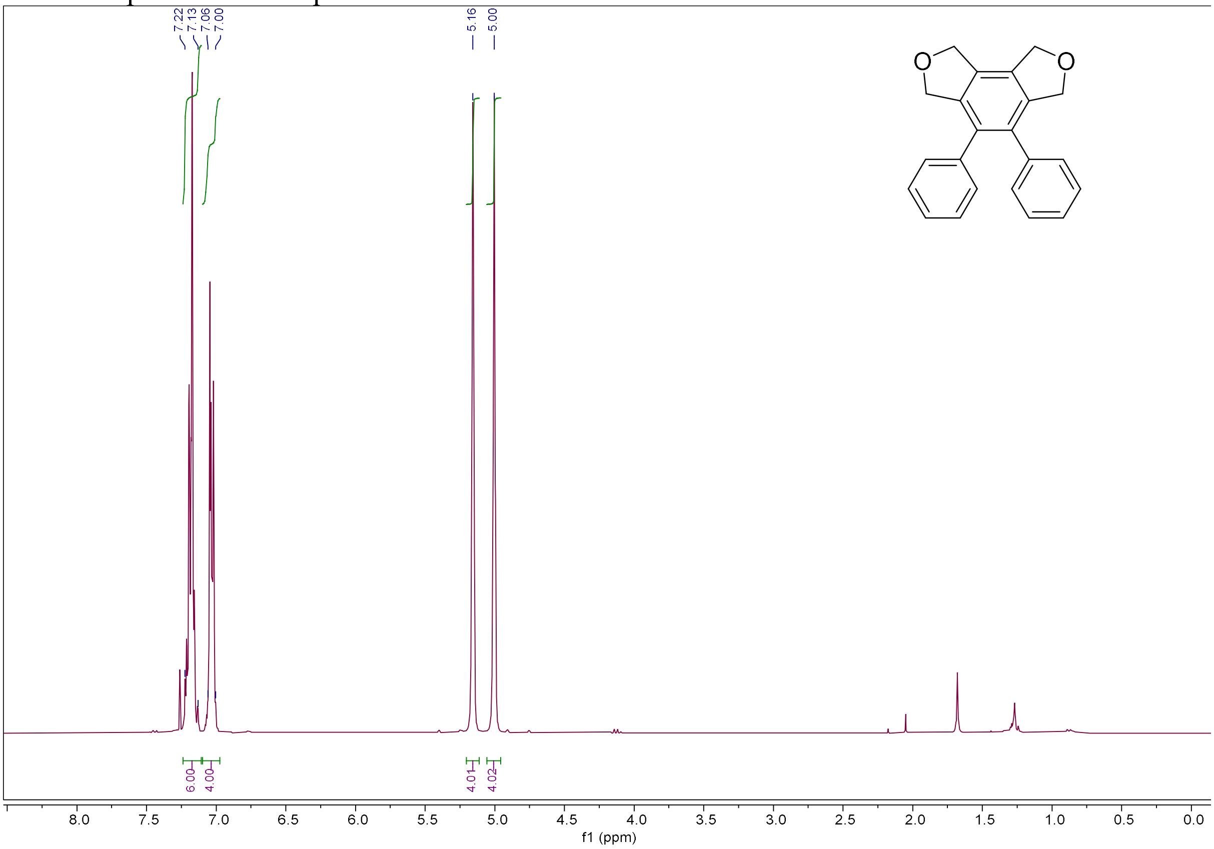

^{1}H NMR spectrum of compound **39**

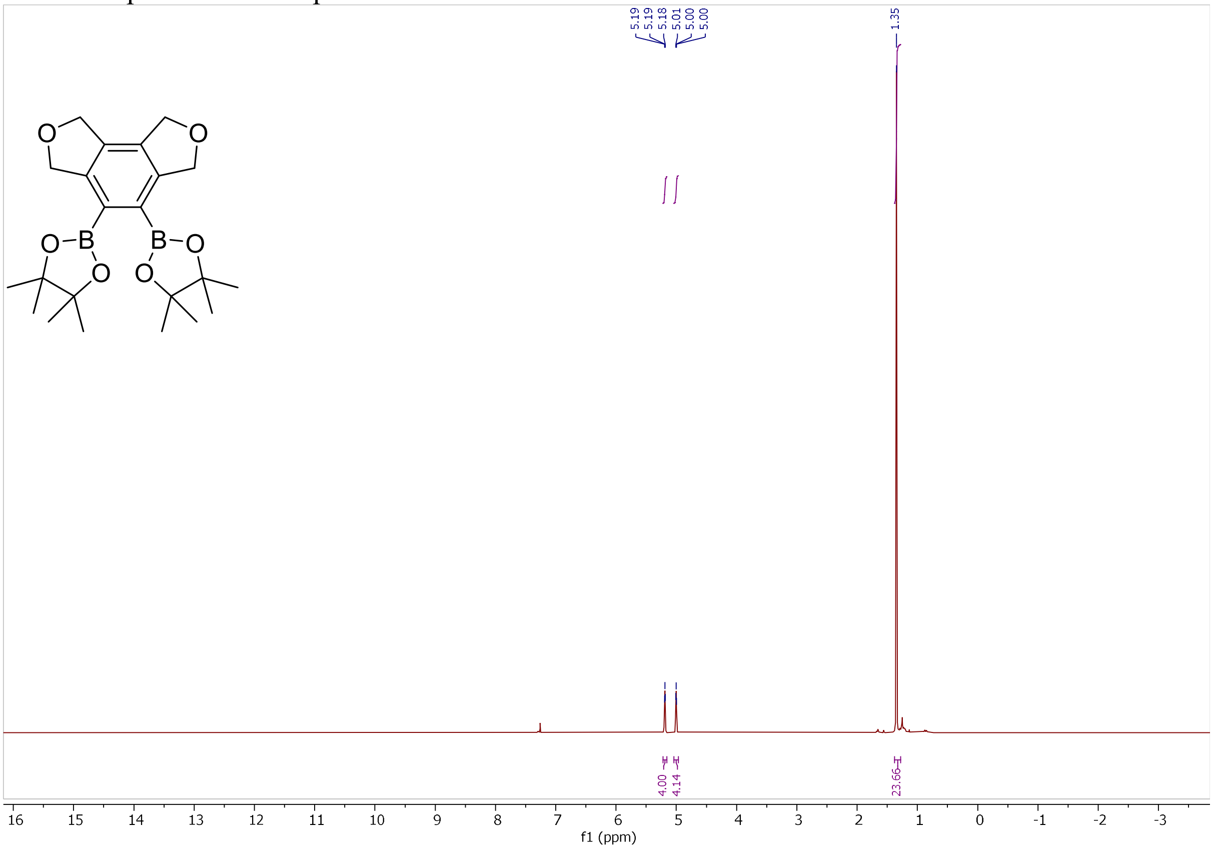

^{11}B NMR spectrum of compound **39**

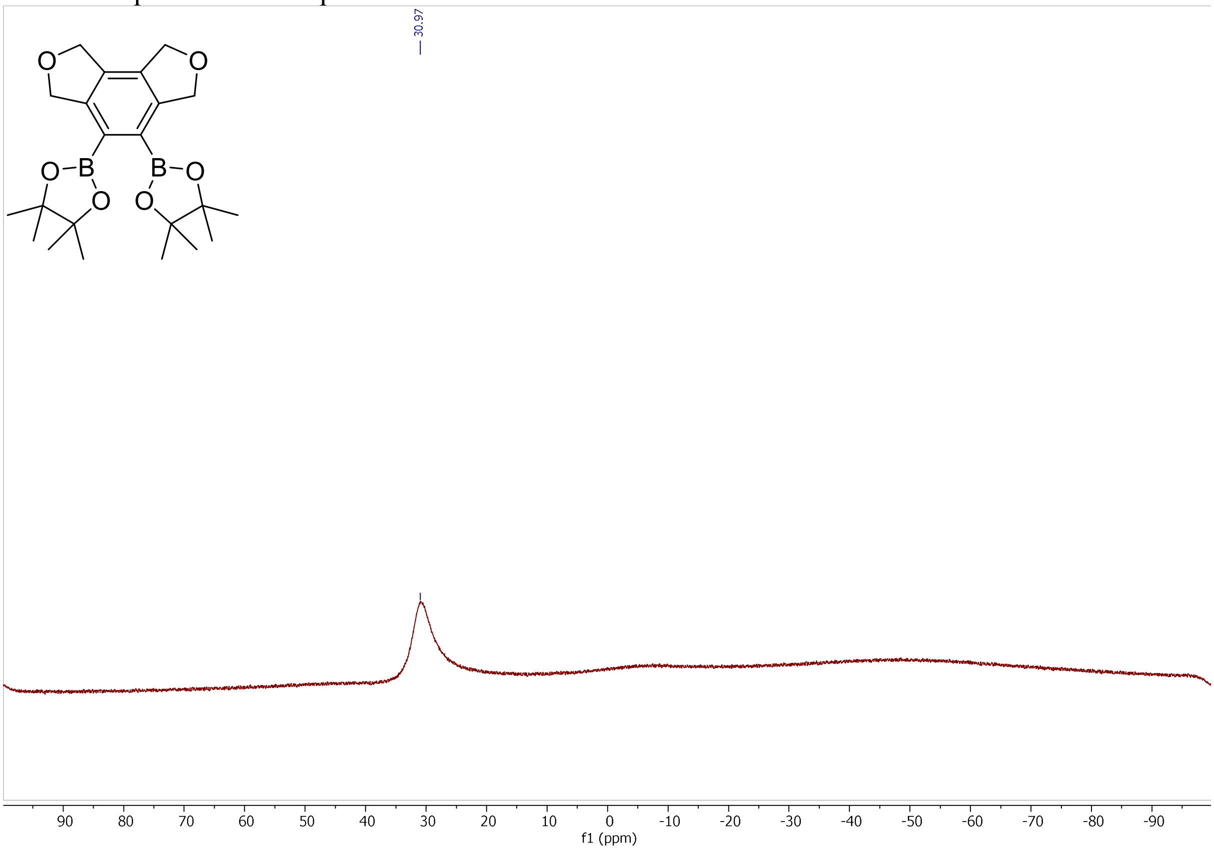

$^{13}C\{^1H\}$ NMR spectrum of **39**

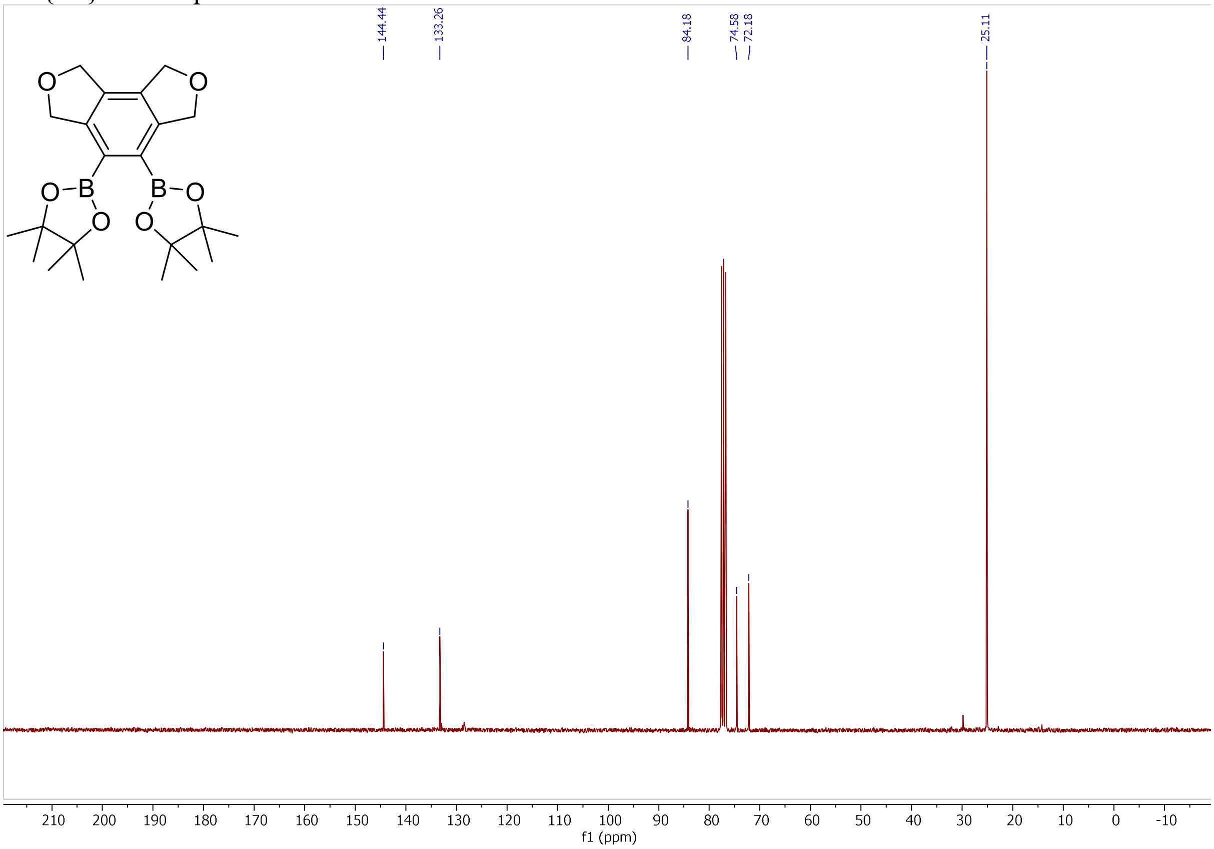

^1H NMR spectrum of compound **40**

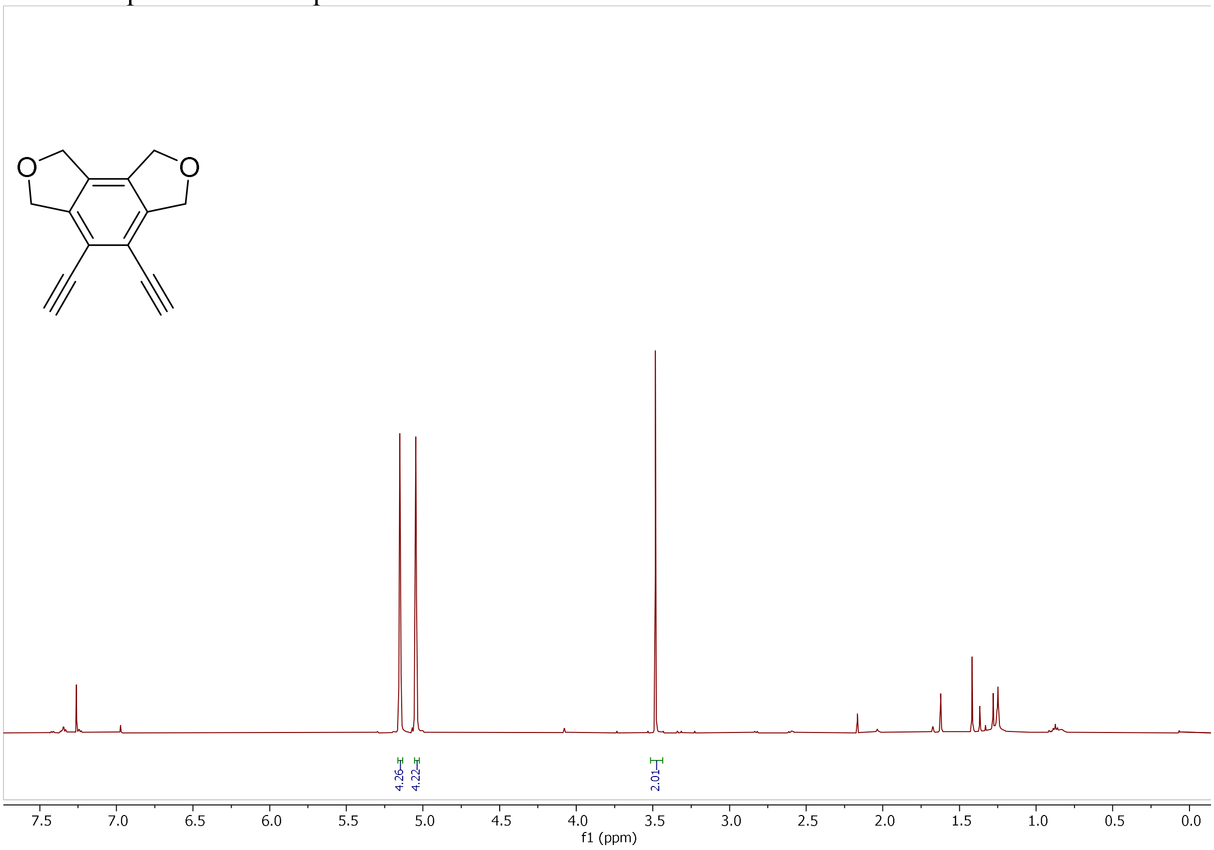

$^{13}C\{^1H\}$ NMR spectrum of **40**

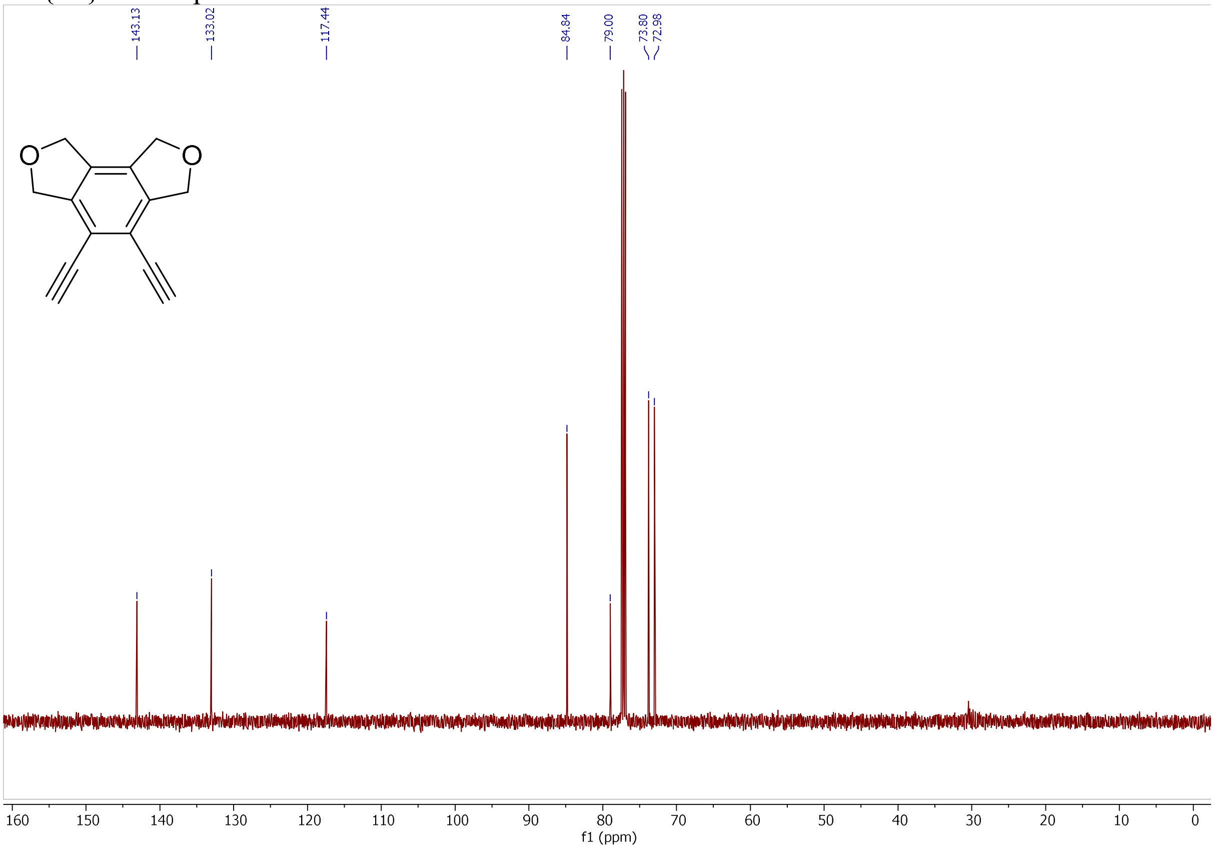

^1H NMR spectrum of compound **41**

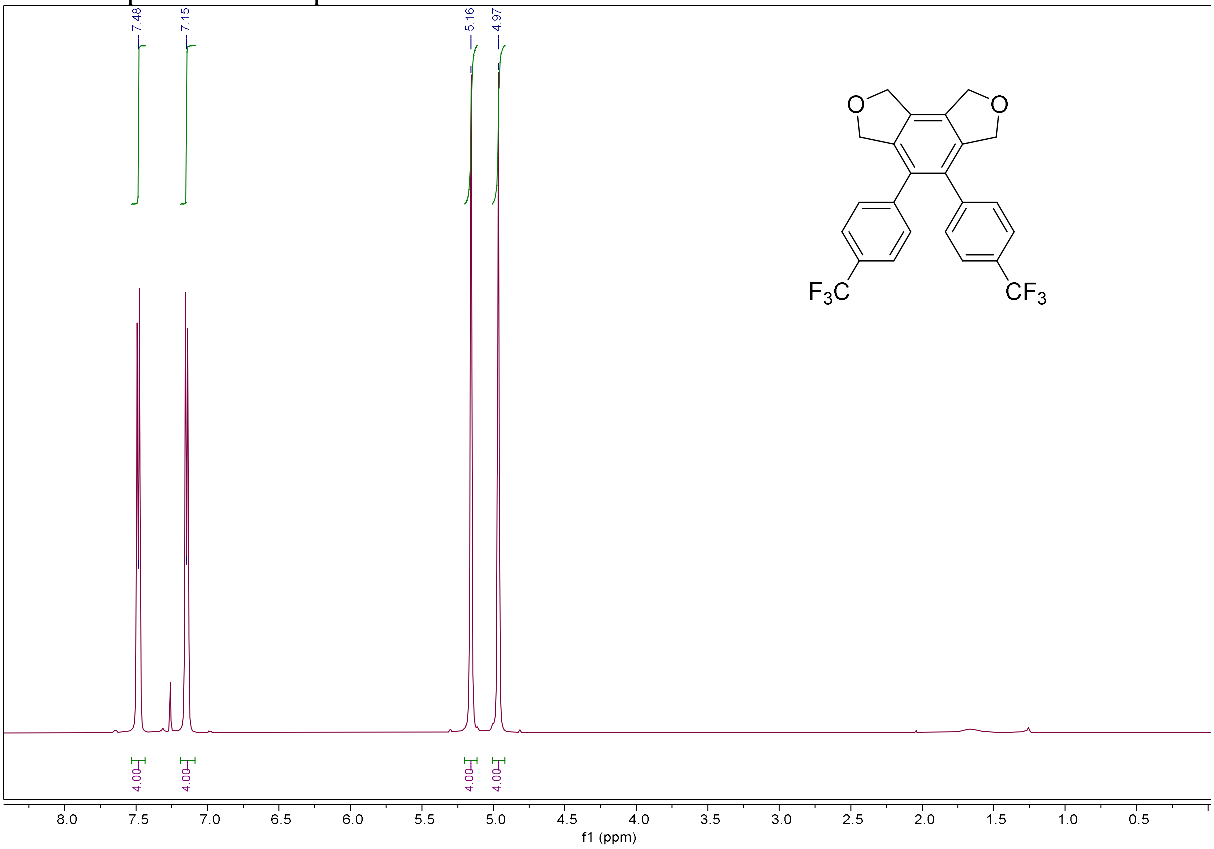

^{1}H NMR spectrum of compound **42**

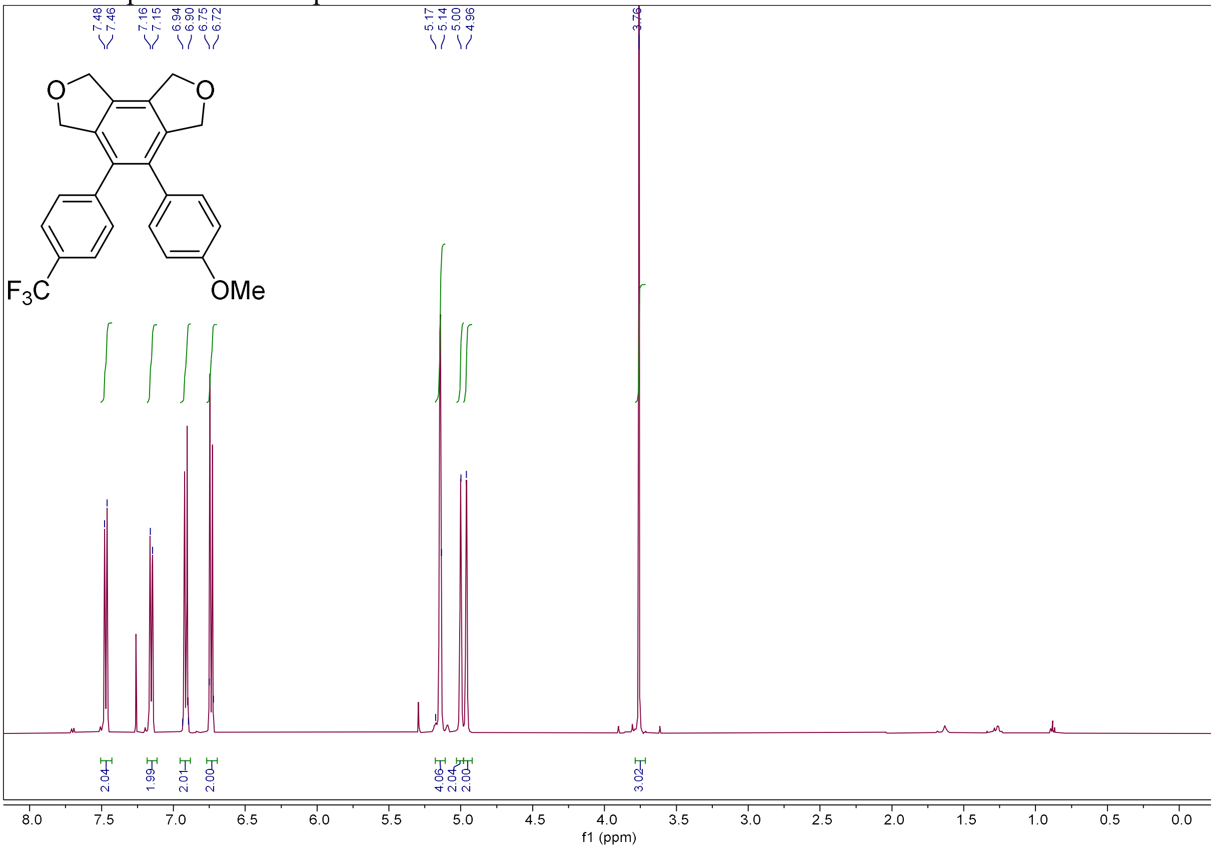

^{13}C{^{1}H} NMR spectrum of **42**

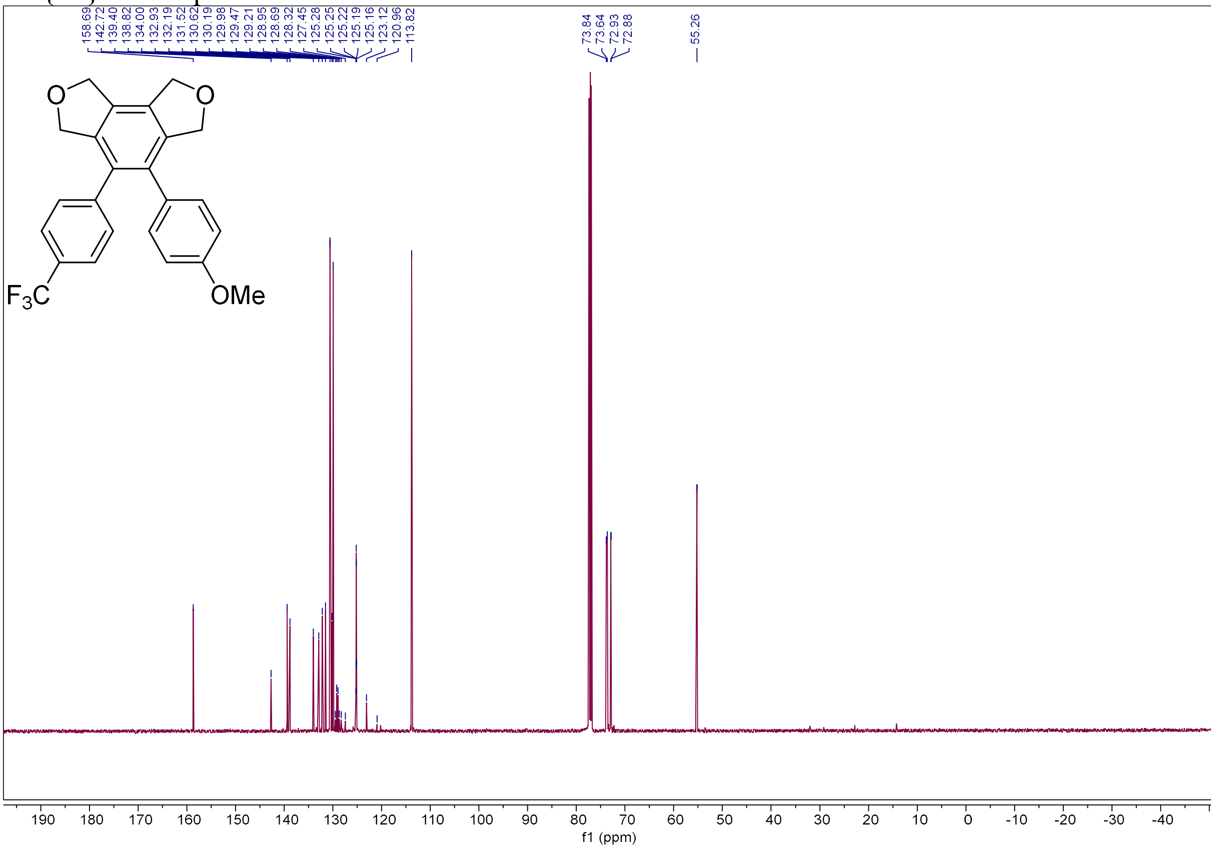

^{19}F NMR spectrum of compound **42**

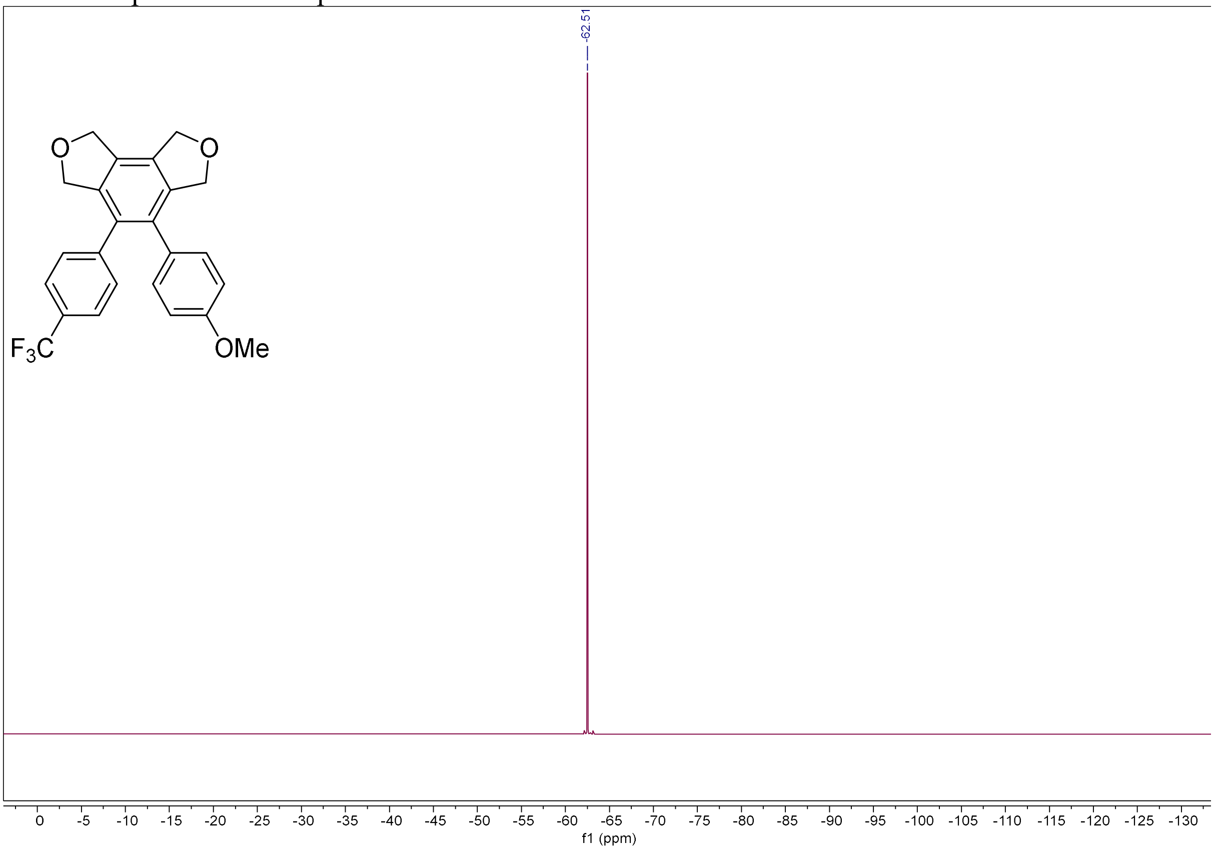

^{1}H NMR spectrum of compound **44**

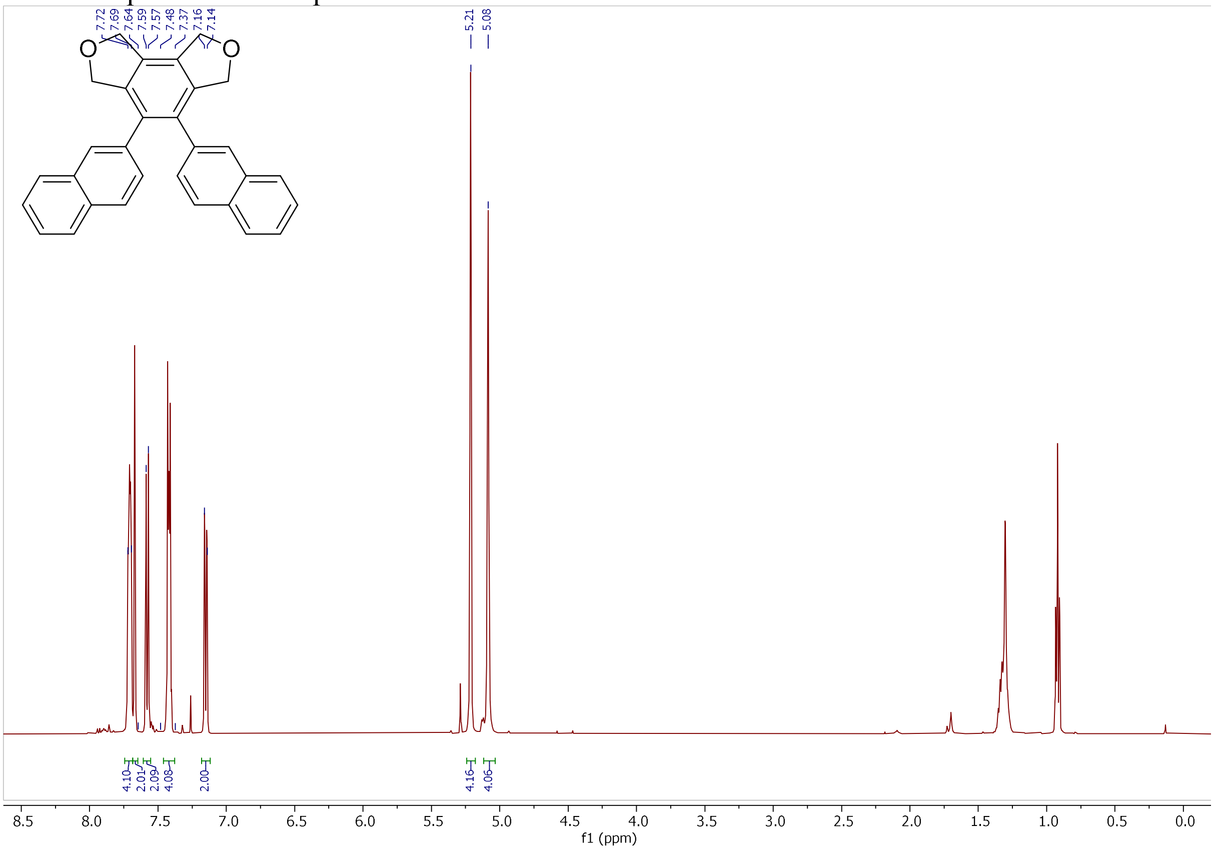

$^{13}C\{^{1}H\}$ NMR spectrum of **44**

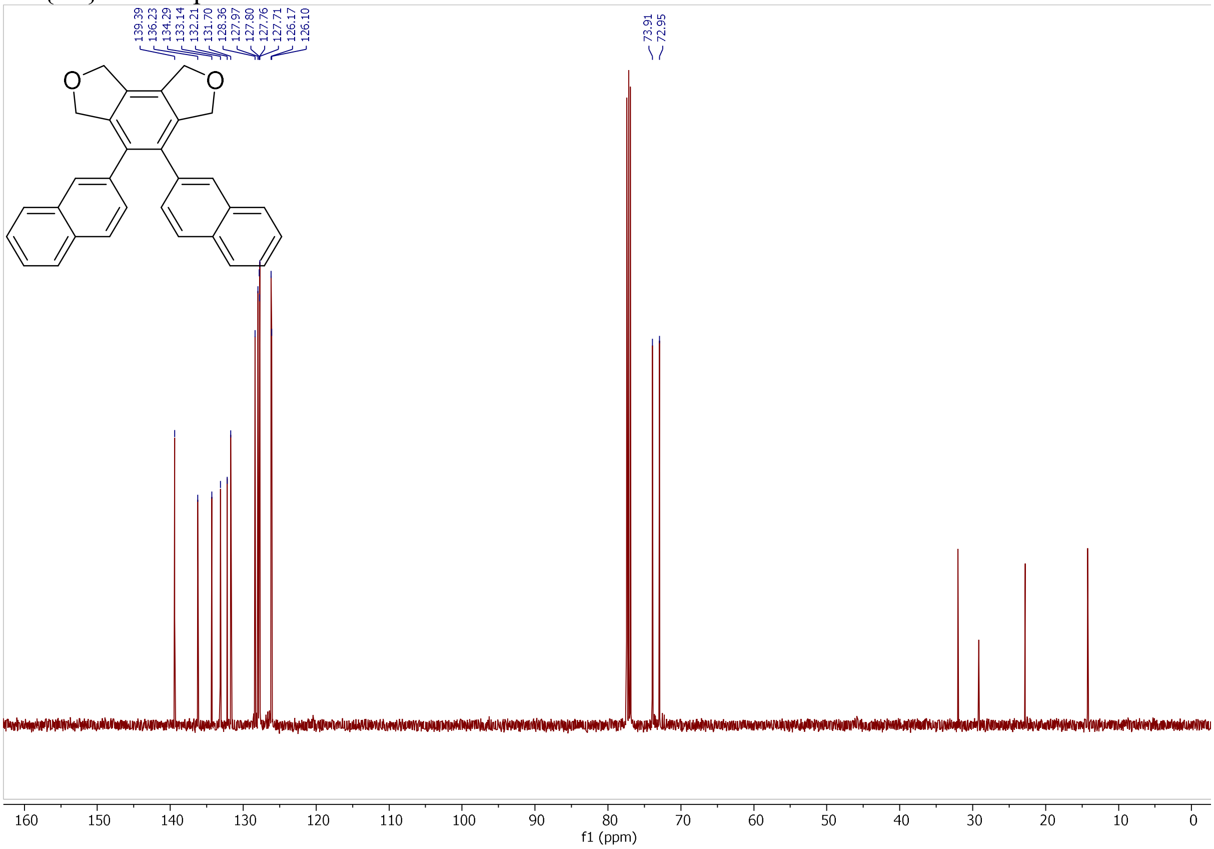

^{1}H NMR spectrum of compound **45** (mixture of *d*, *l* and *meso* compounds).

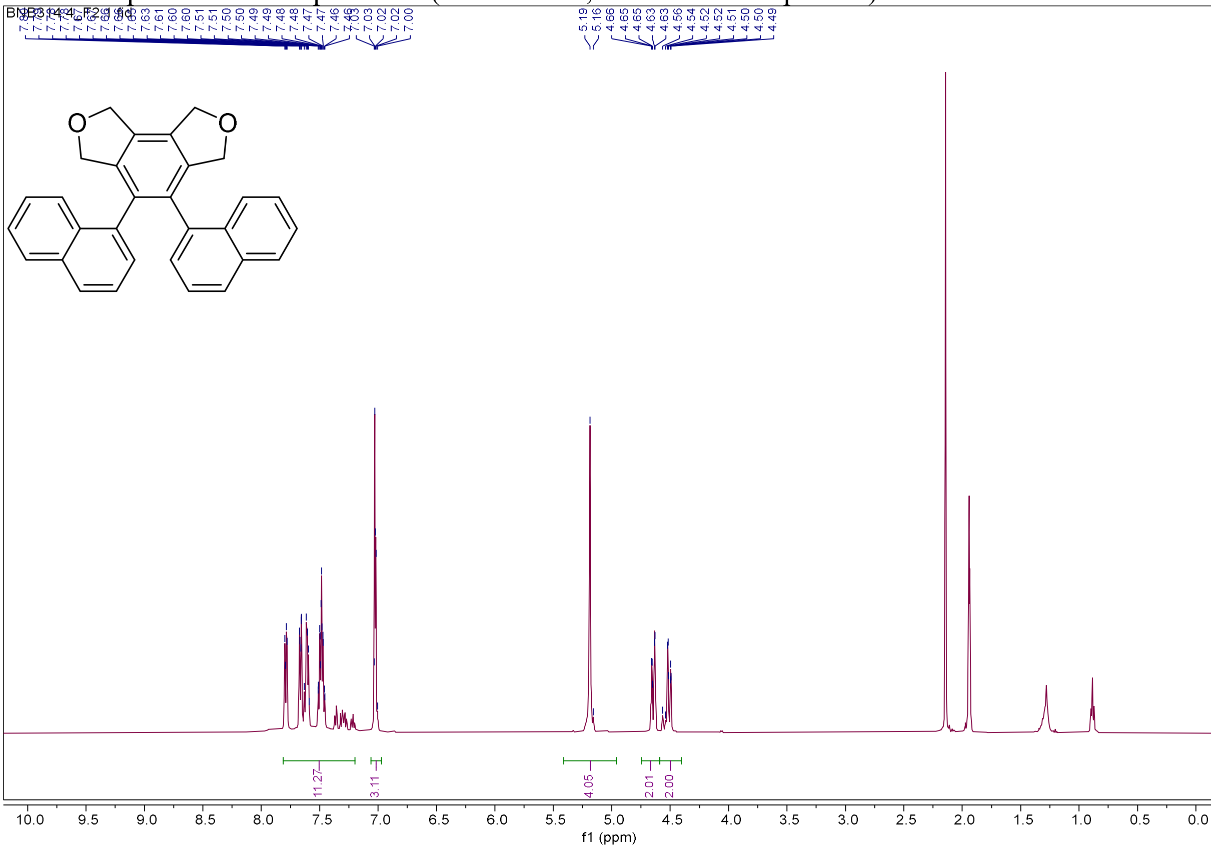

^{1}H NMR spectrum of compound **46**

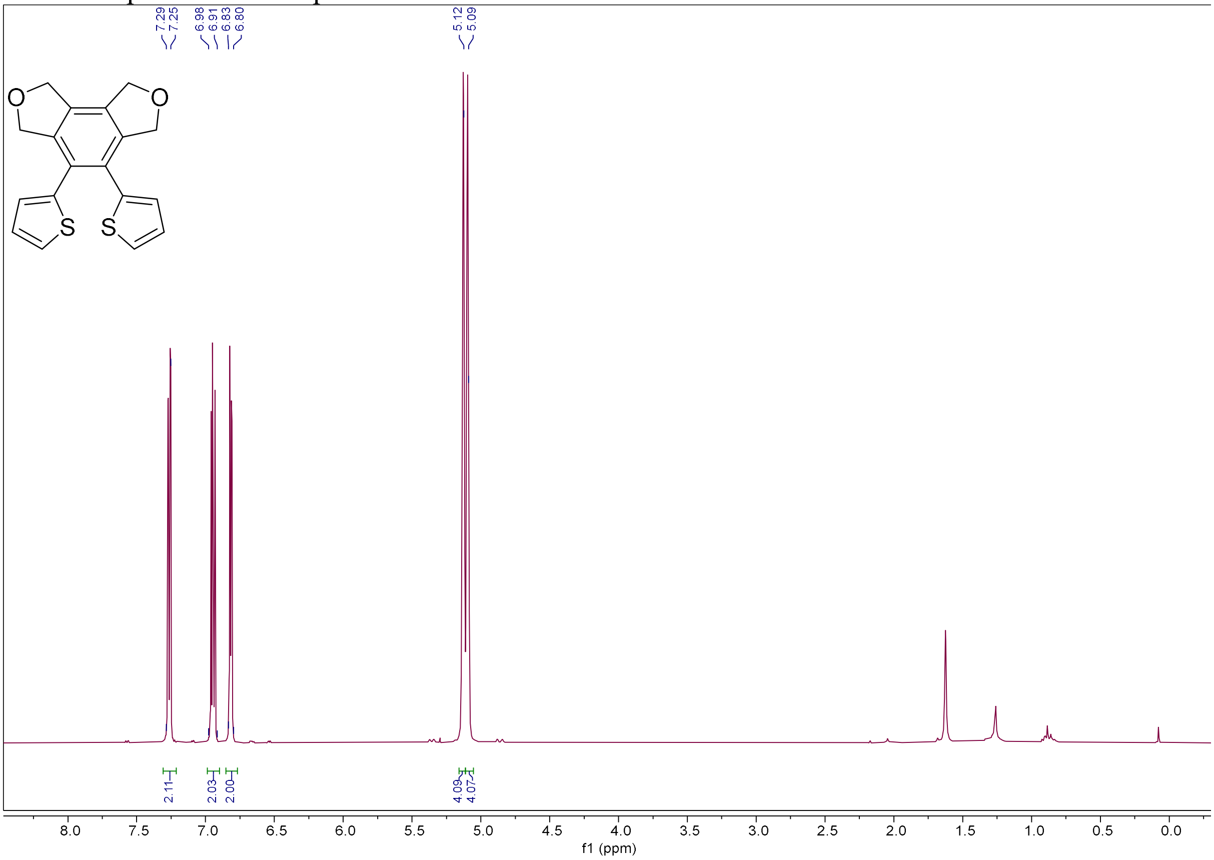

^{13}C{^{1}H} NMR spectrum of **46**

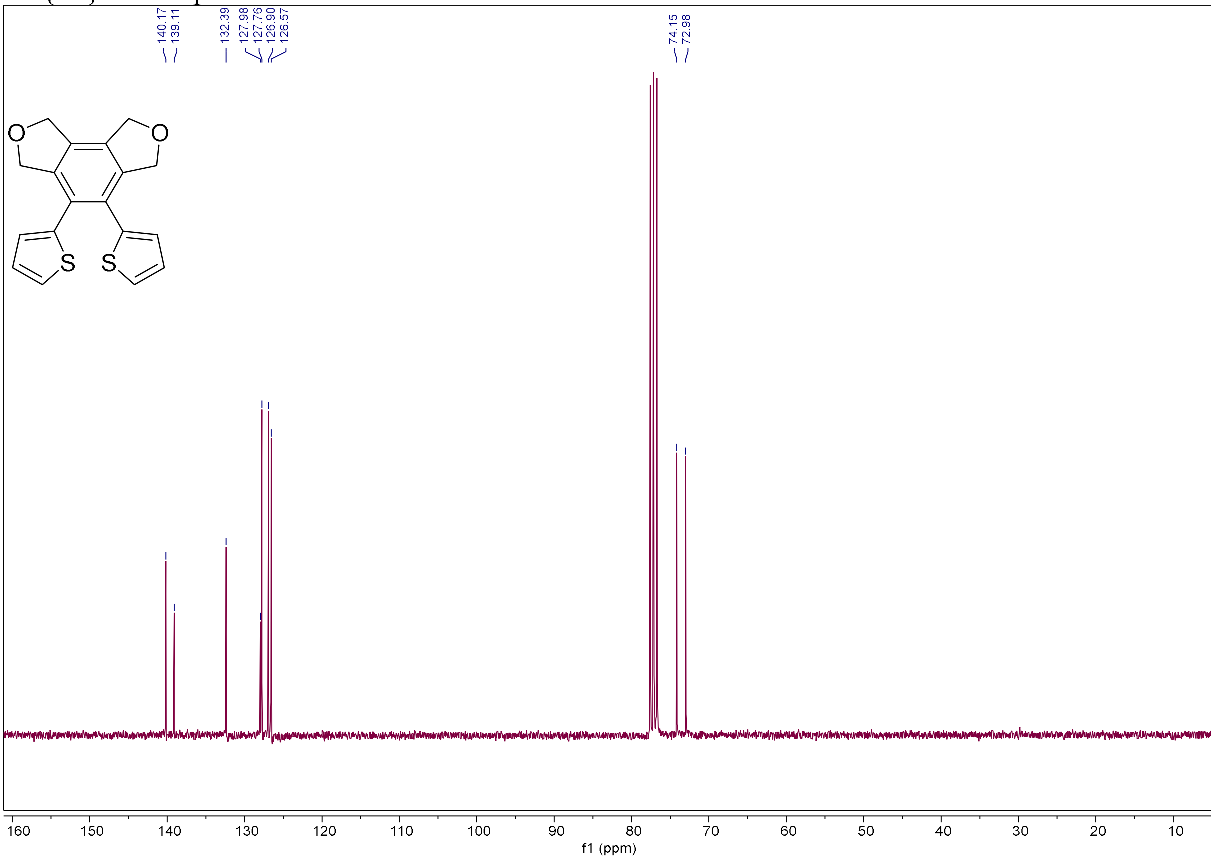

^{1}H NMR spectrum of compound **47**

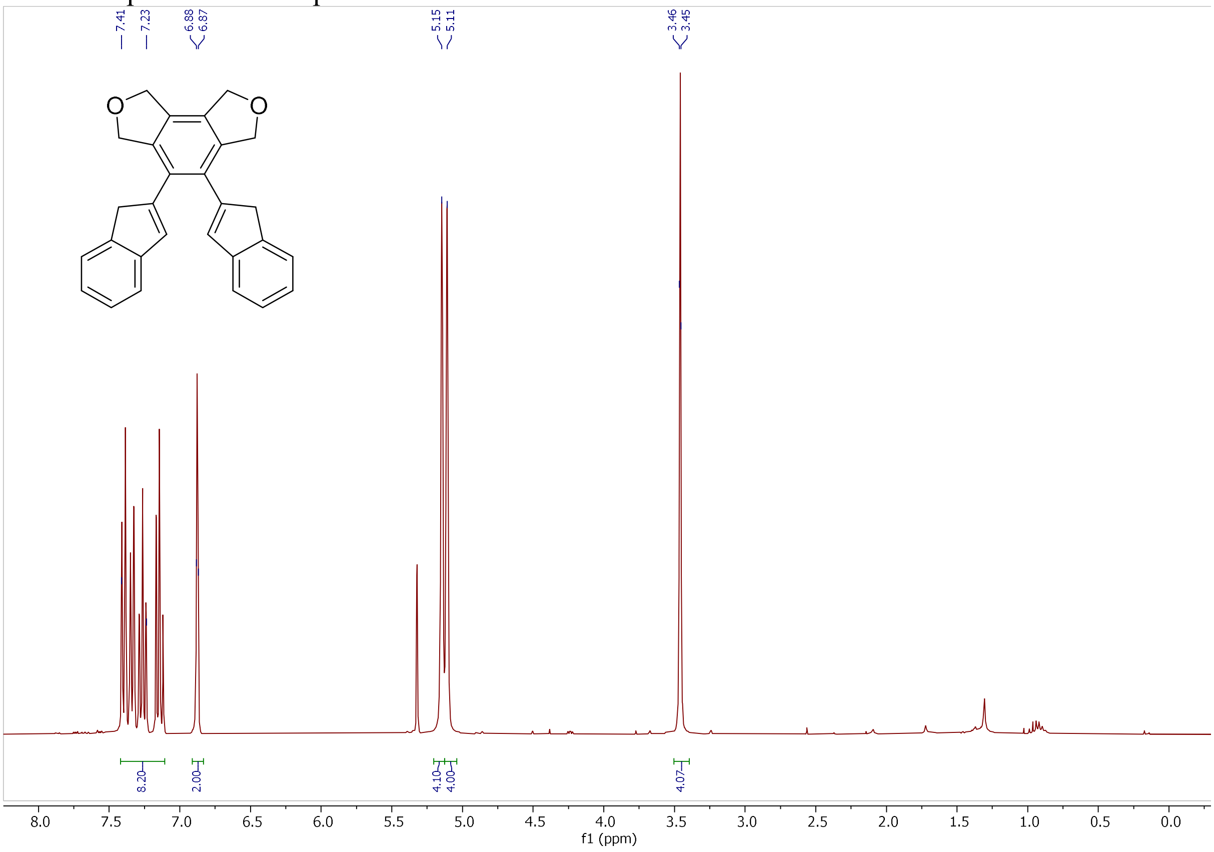

^{13}C{^{1}H} NMR spectrum of **47**

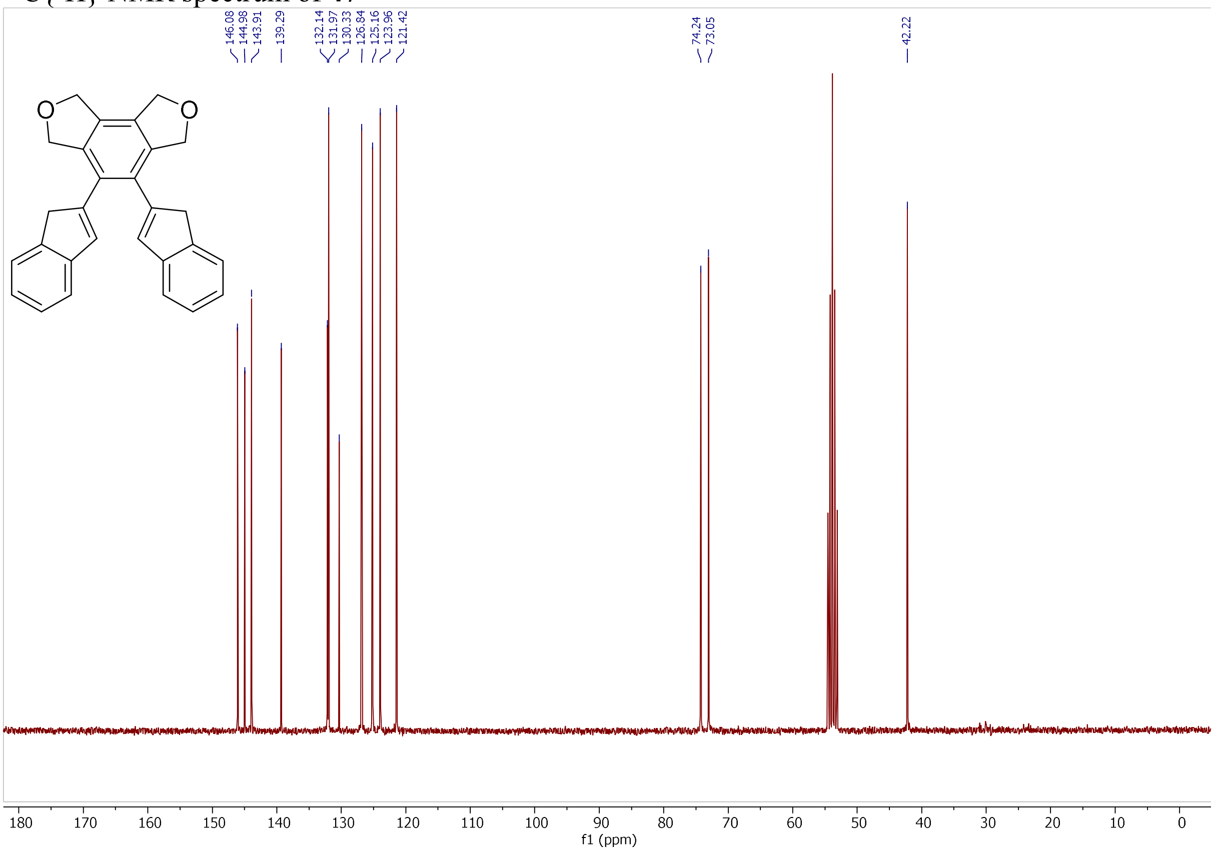

^{1}H NMR spectrum of compound **48**

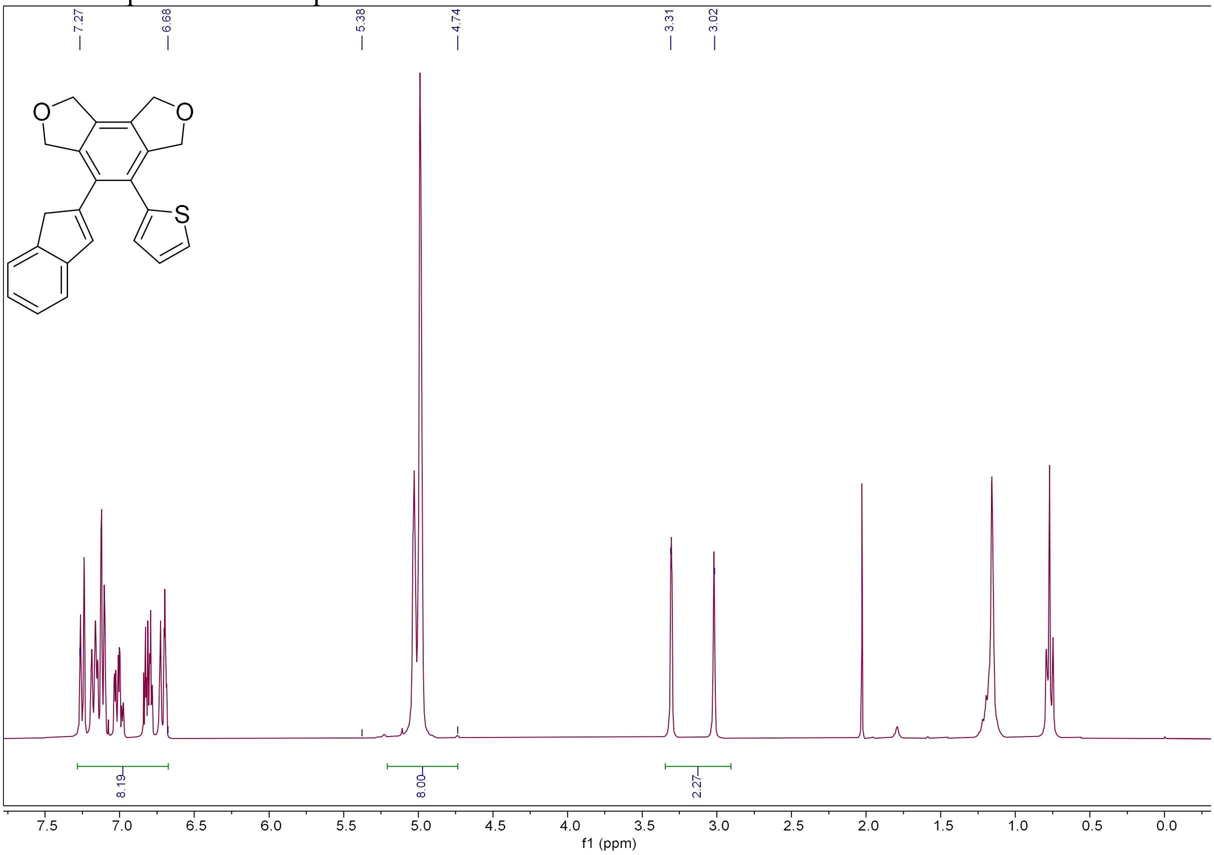

^{1}H NMR spectrum of compound **49**

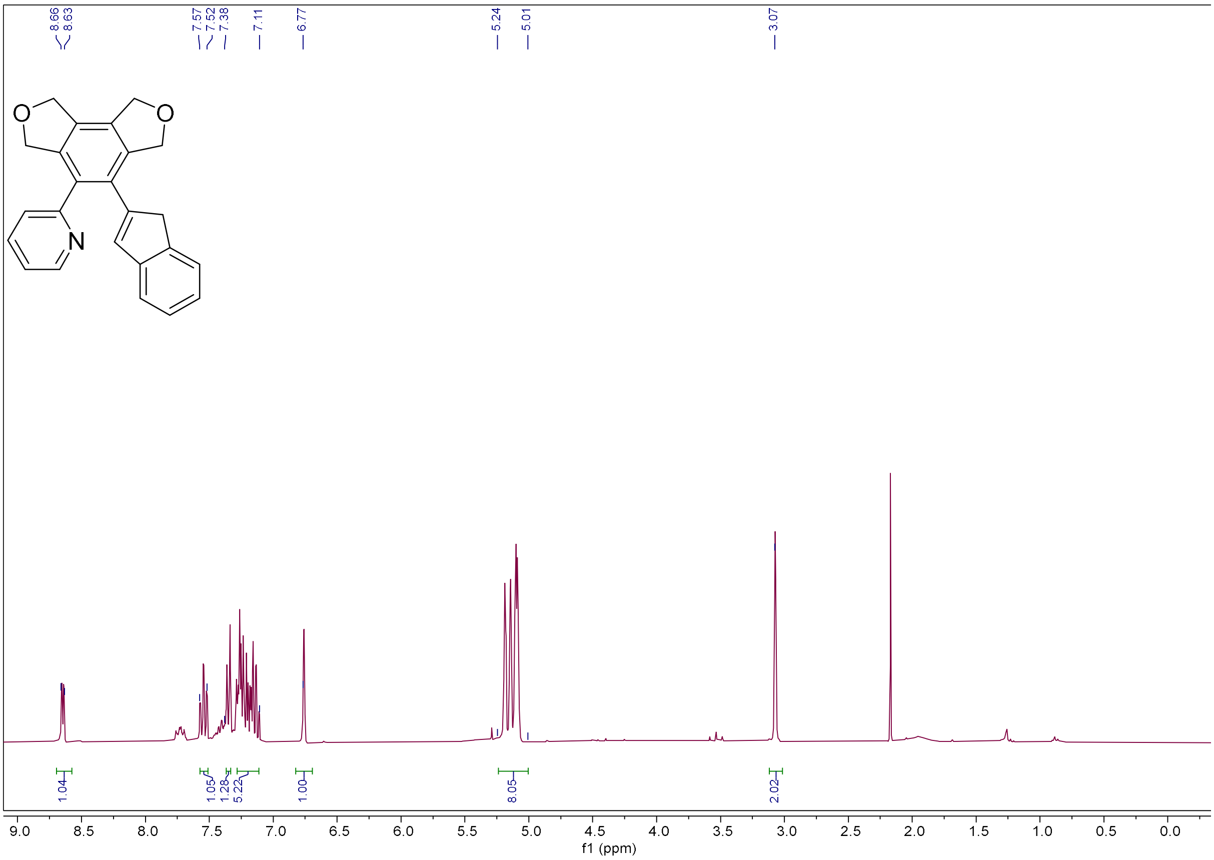

$^{13}C\{^1H\}$ NMR spectrum of **49**

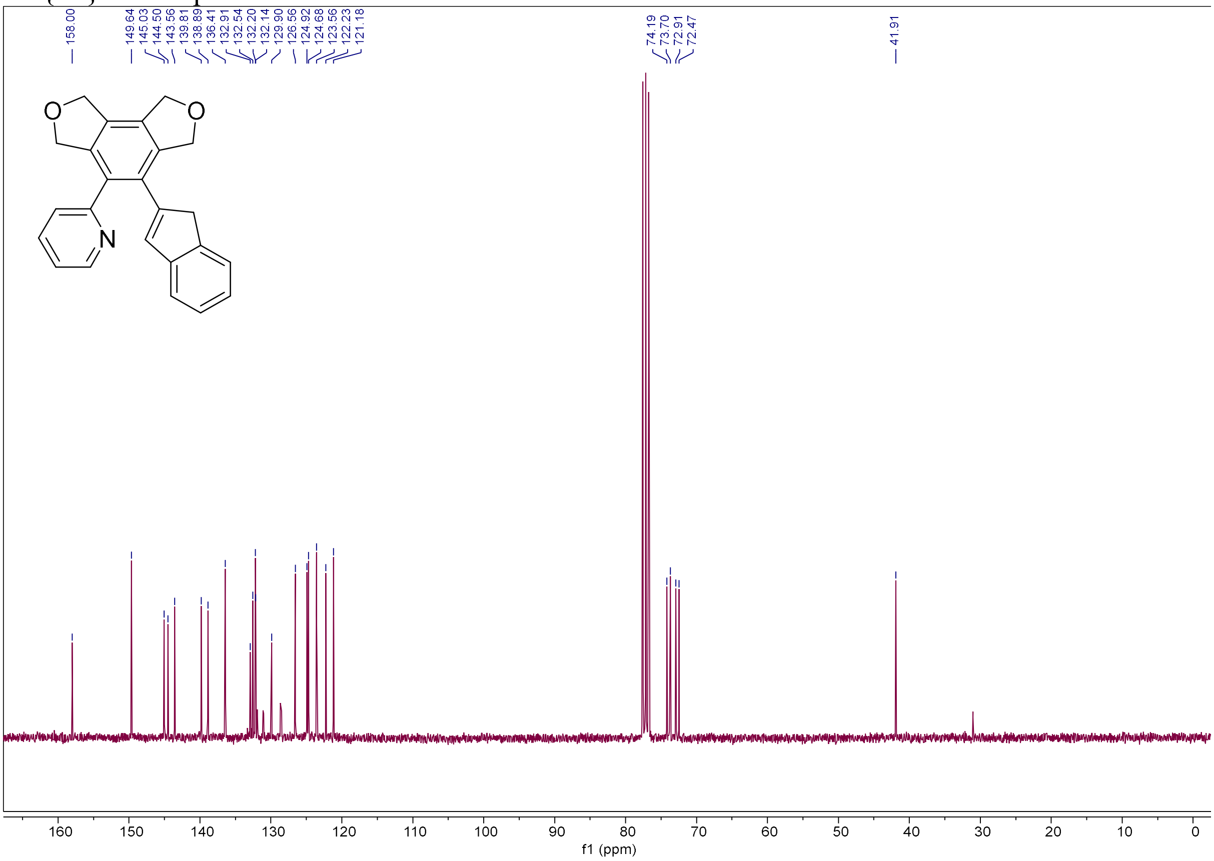

^1H NMR spectrum of compound **50**

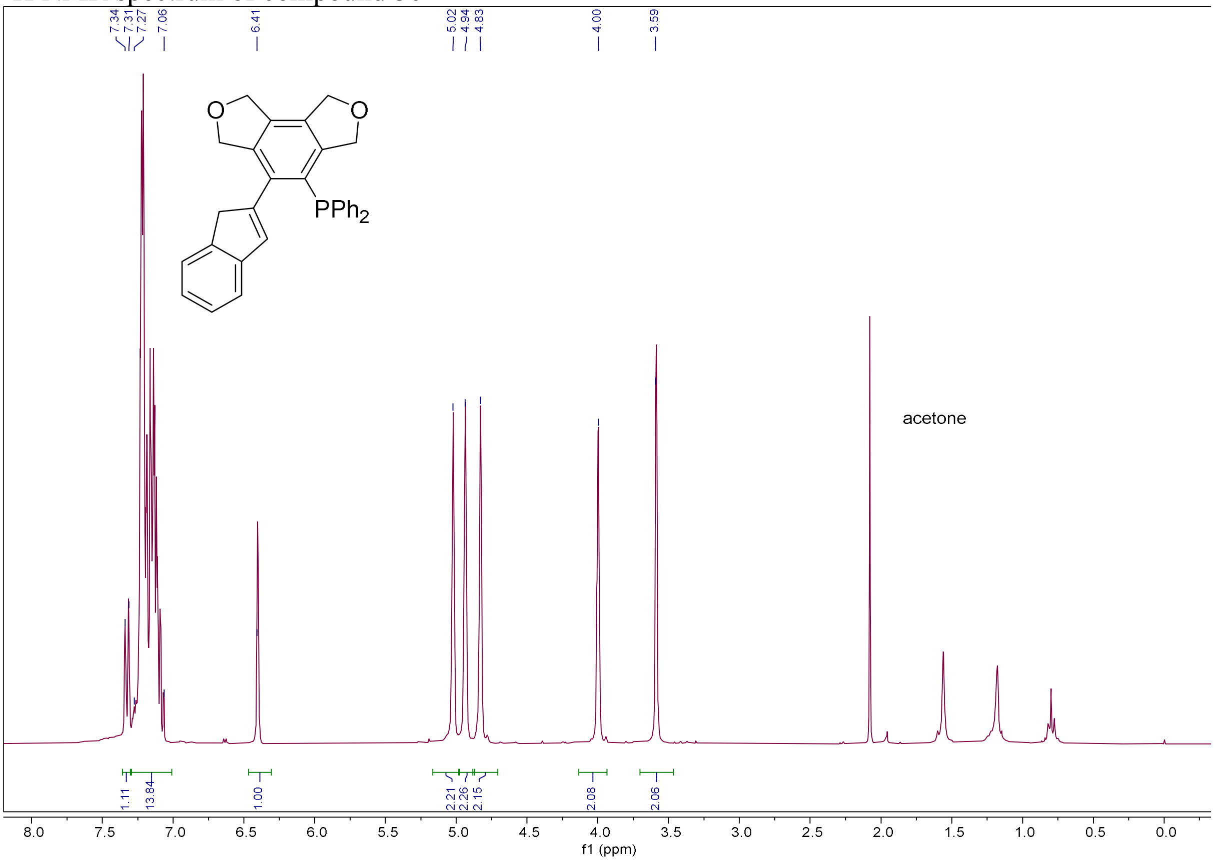

$^{13}C\{^1H\}$ NMR spectrum of **50**

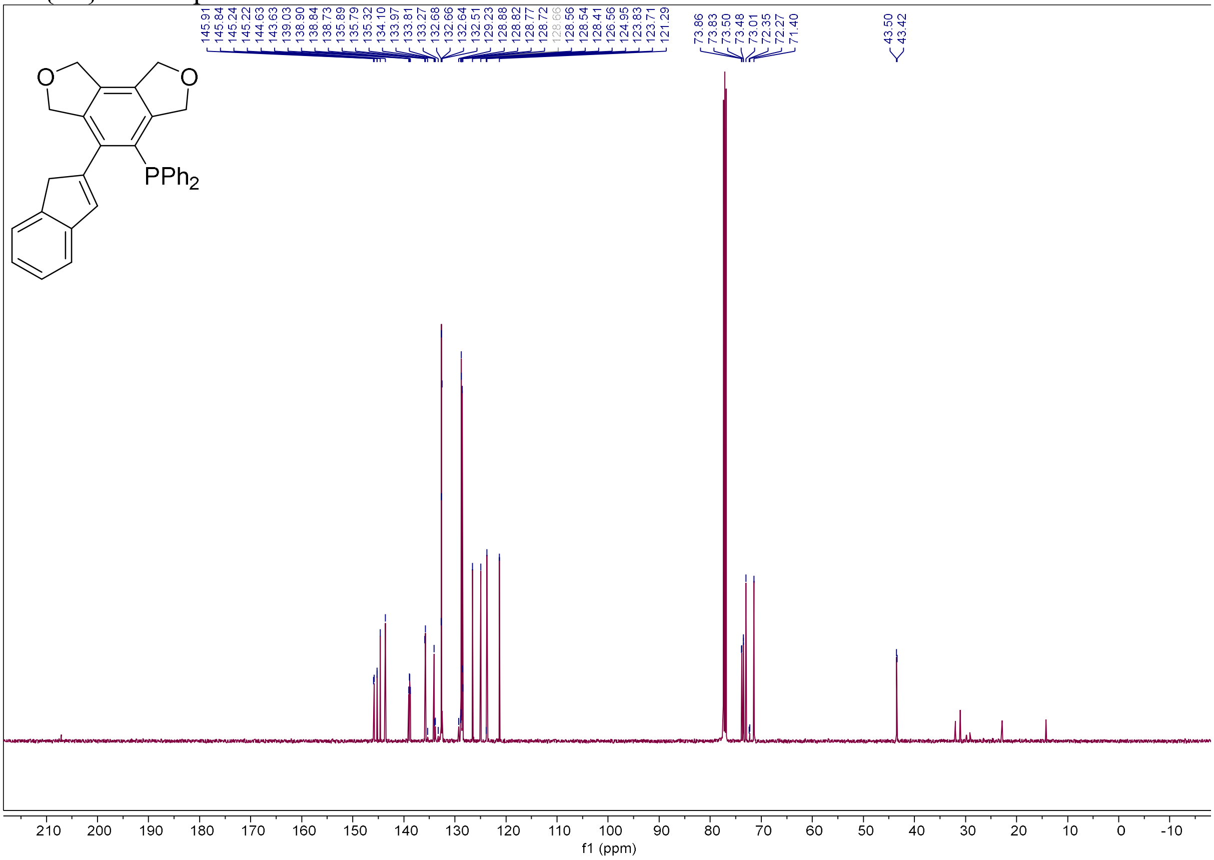

$^{31}P\{^1H\}$ NMR spectrum of compound **50**

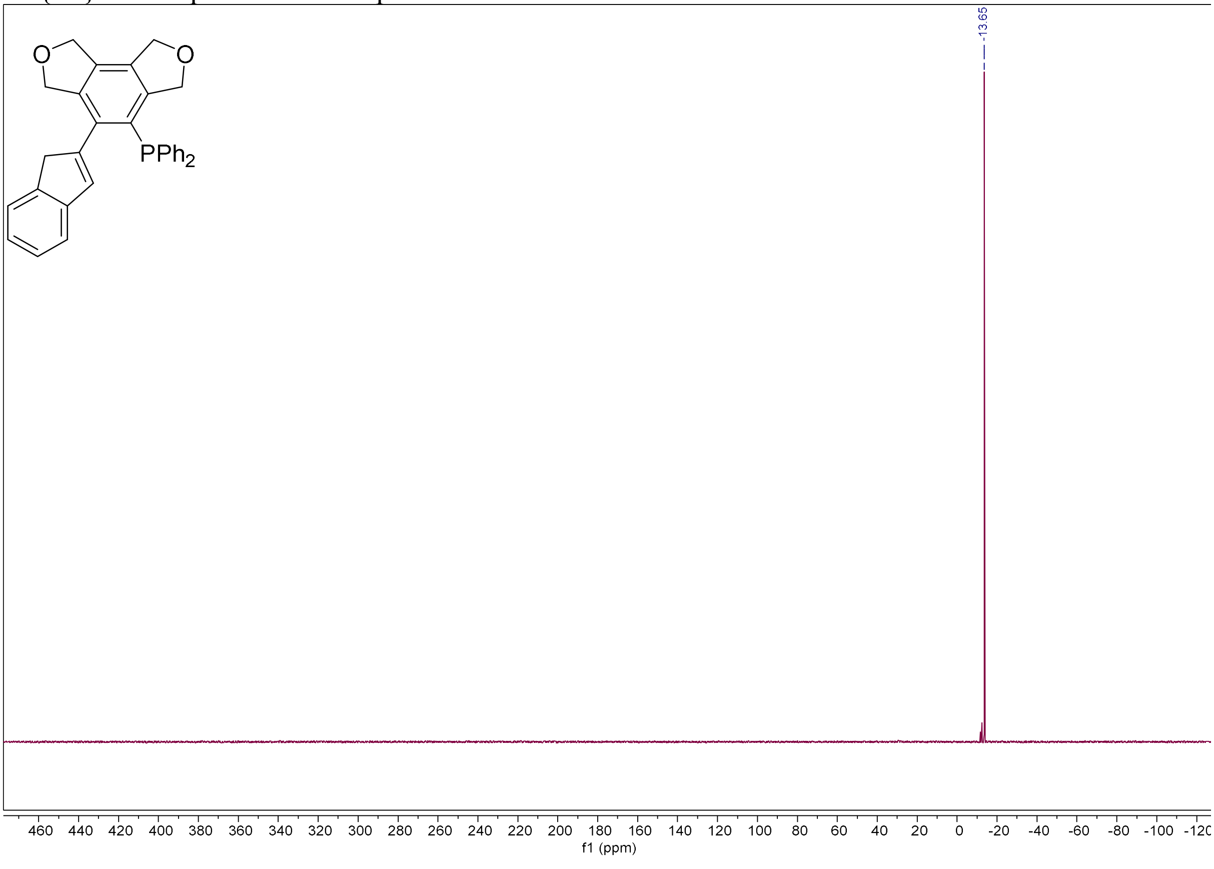

^{1}H NMR NMR spectrum of compound **51**

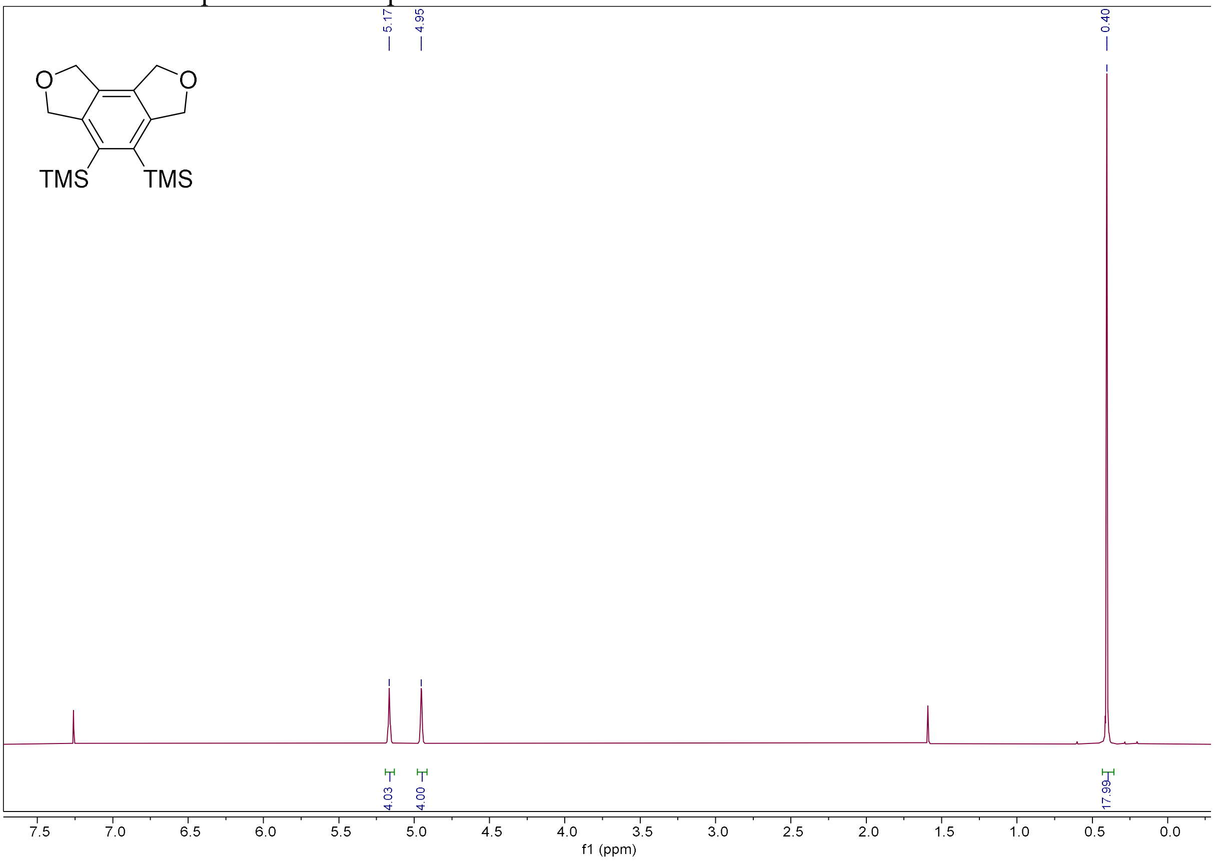

^{1}H NMR NMR spectrum of compound **52**

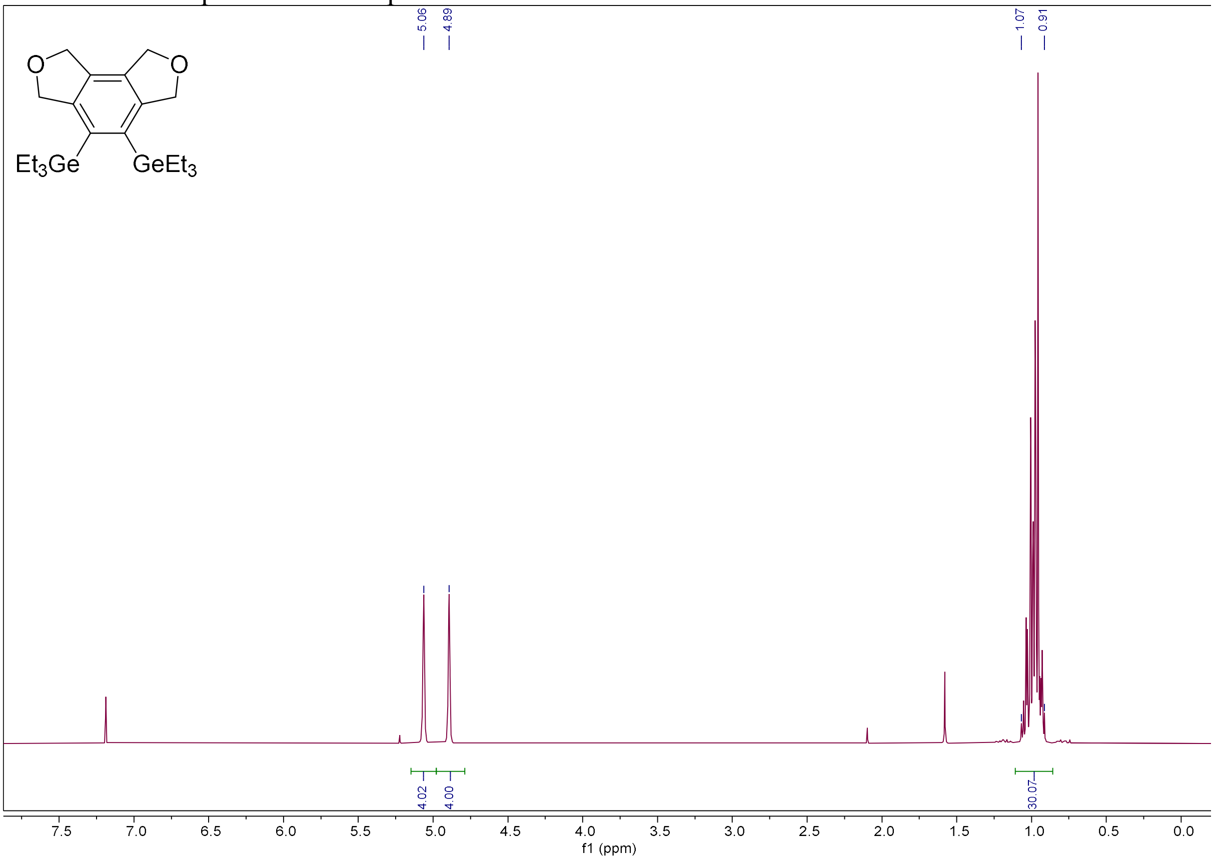

$^{13}C\{^1H\}$ NMR spectrum of compound **52**

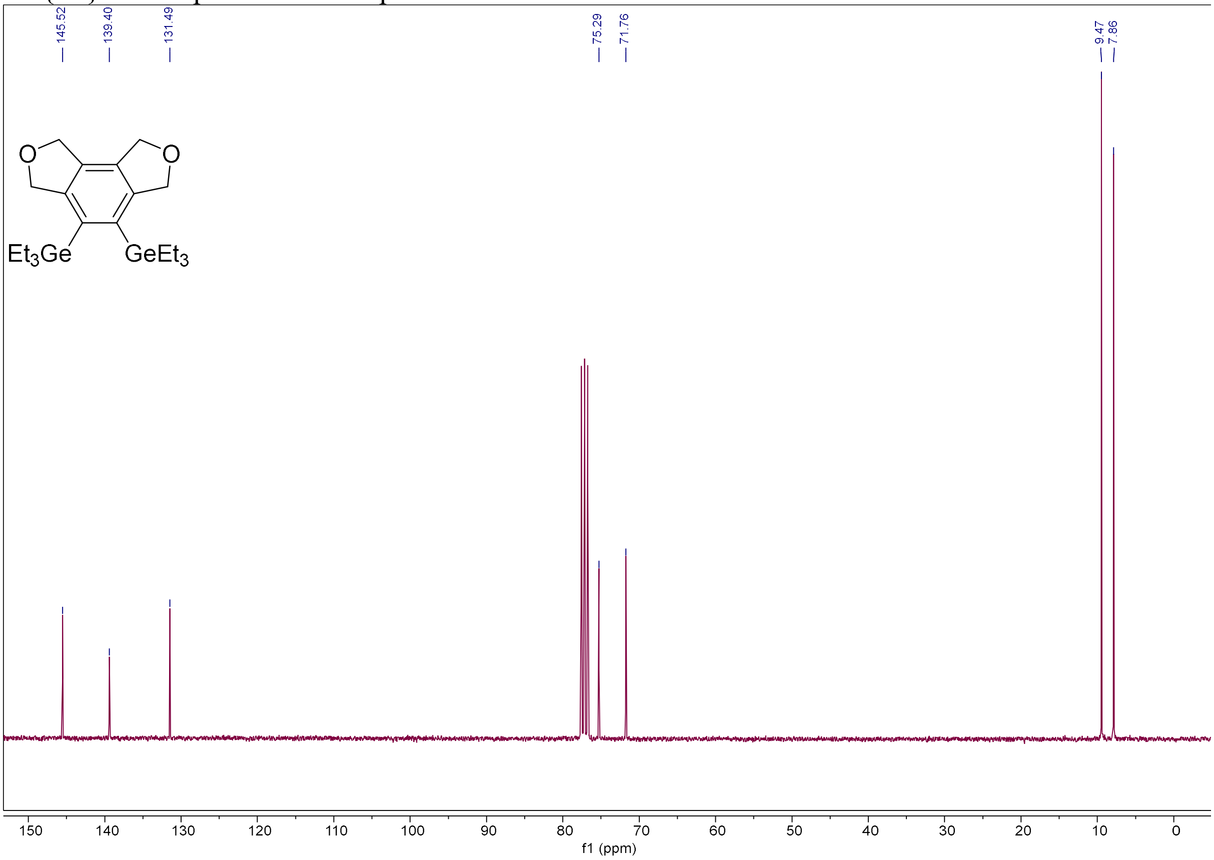

^1H NMR spectrum of compound **53** (mixture of *d*, *l* and *meso* compounds. Ratio of *d*,*l*:*meso* = 3:1).

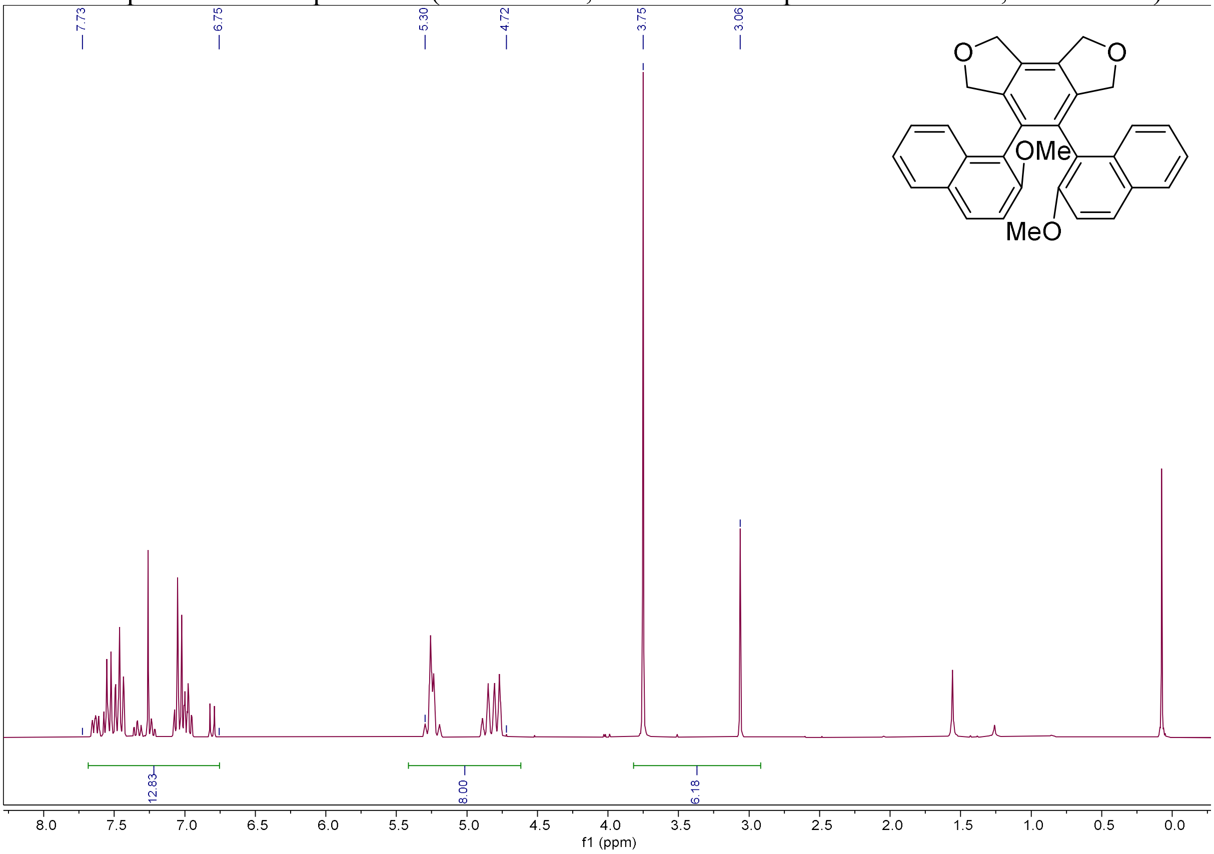

^{1}H NMR spectrum of compound **53** (mixture of *d* and *l* compounds).

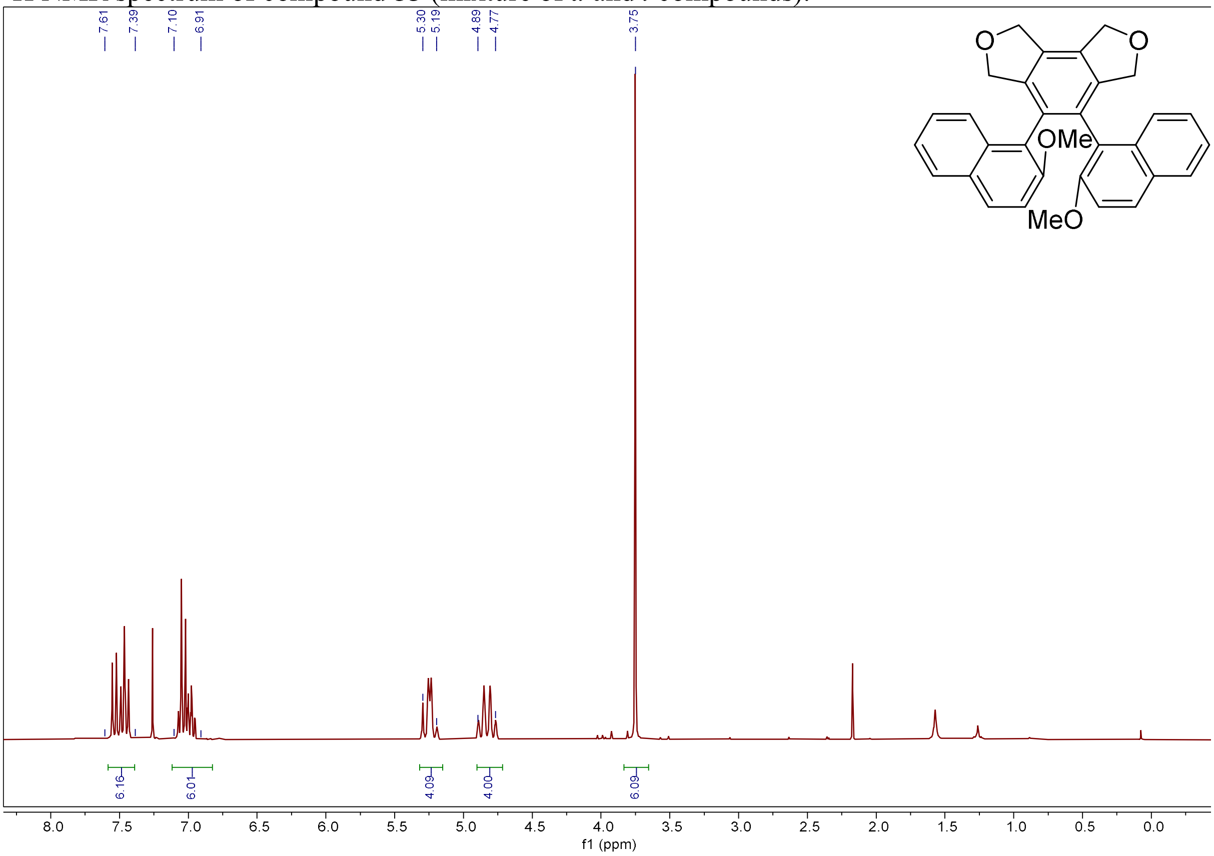

$^{13}C\{^{1}H\}$ NMR spectrum of compound **53** (mixture of *d*, *l* and *meso* compounds. Ratio of *d*,*l*:*meso* = 3:1).

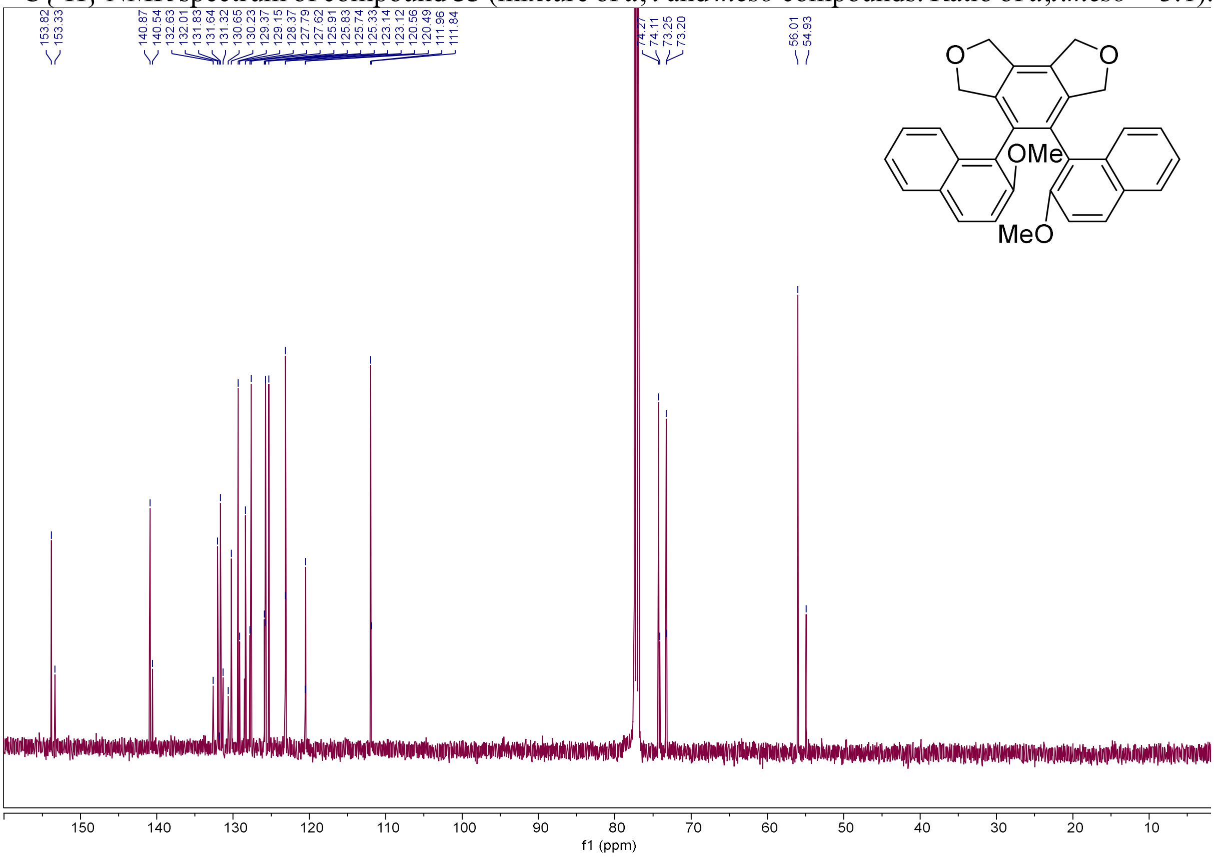

$^{13}C\{^1H\}$ NMR spectrum of compound **53** (mixture of *d* and *l* compounds).

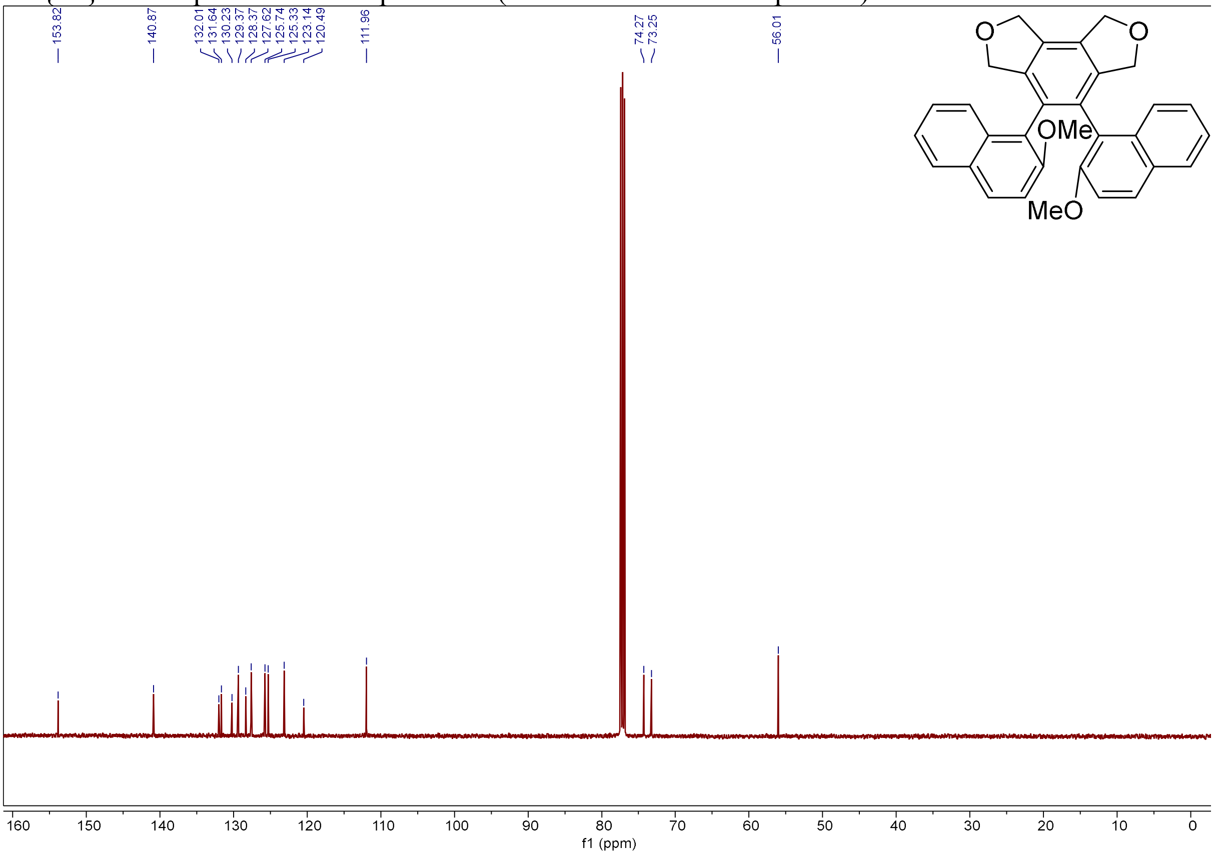

^1H NMR spectrum of compound **54** (mixture of *d*, *l* and *meso* compounds).

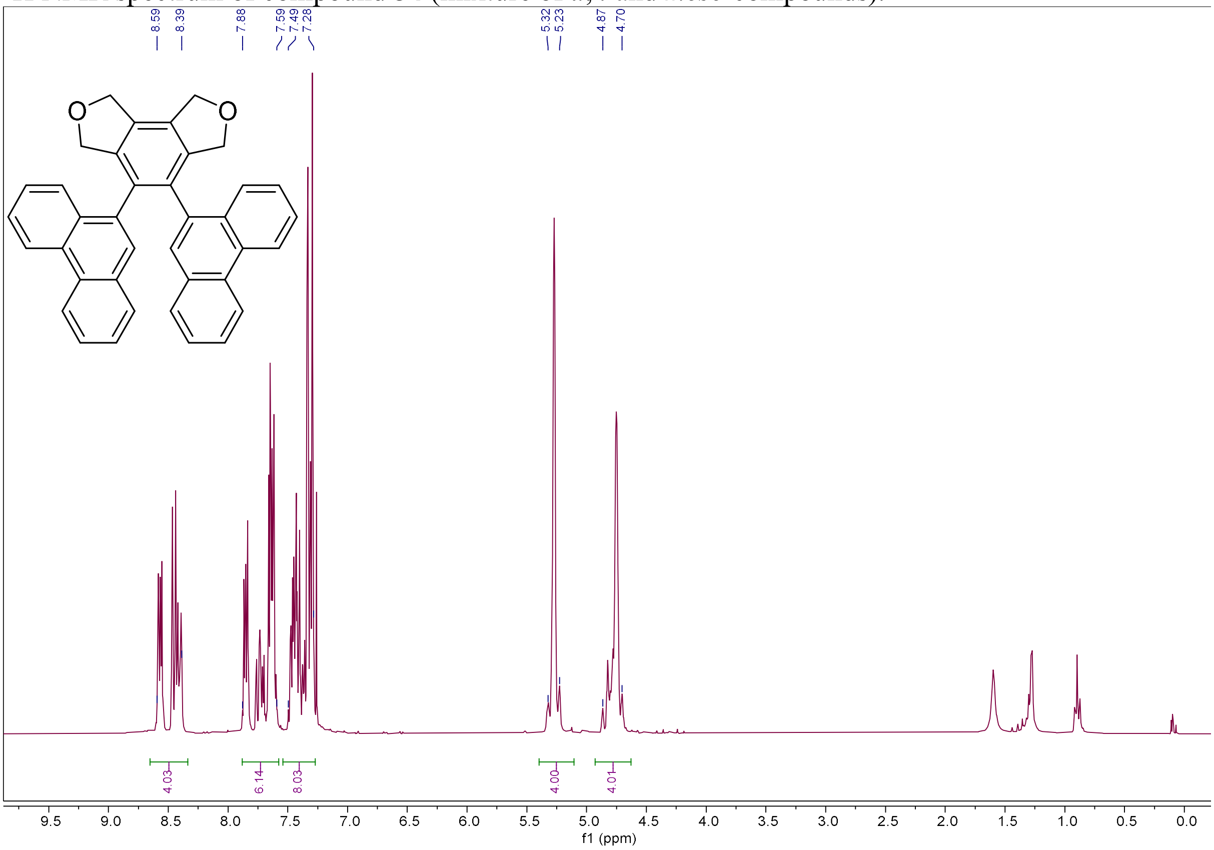

^{1}H NMR spectrum of compound **55** (mixture of *d*, *l* and *meso* compounds).

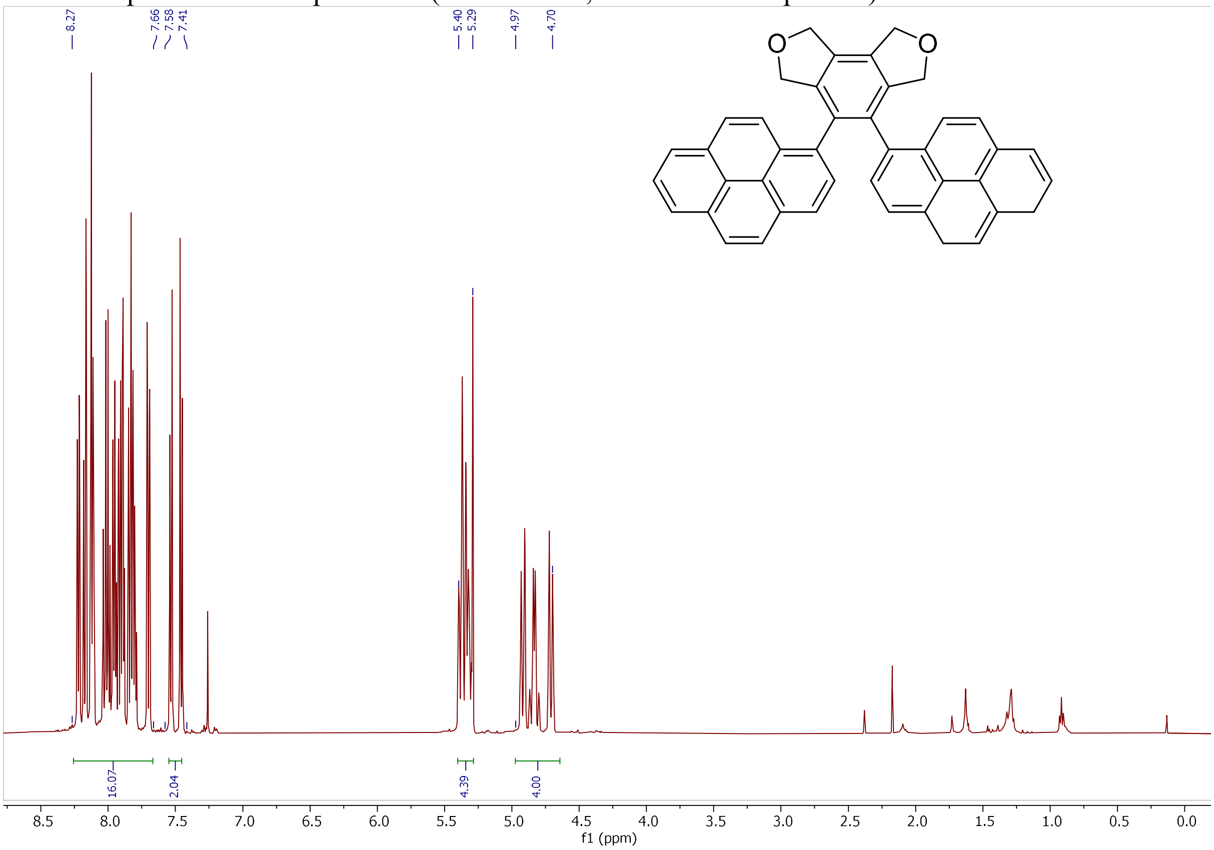

^{13}C{^{1}H} NMR spectrum of compound **55** (mixture of *d*, *l* and *meso* compounds).

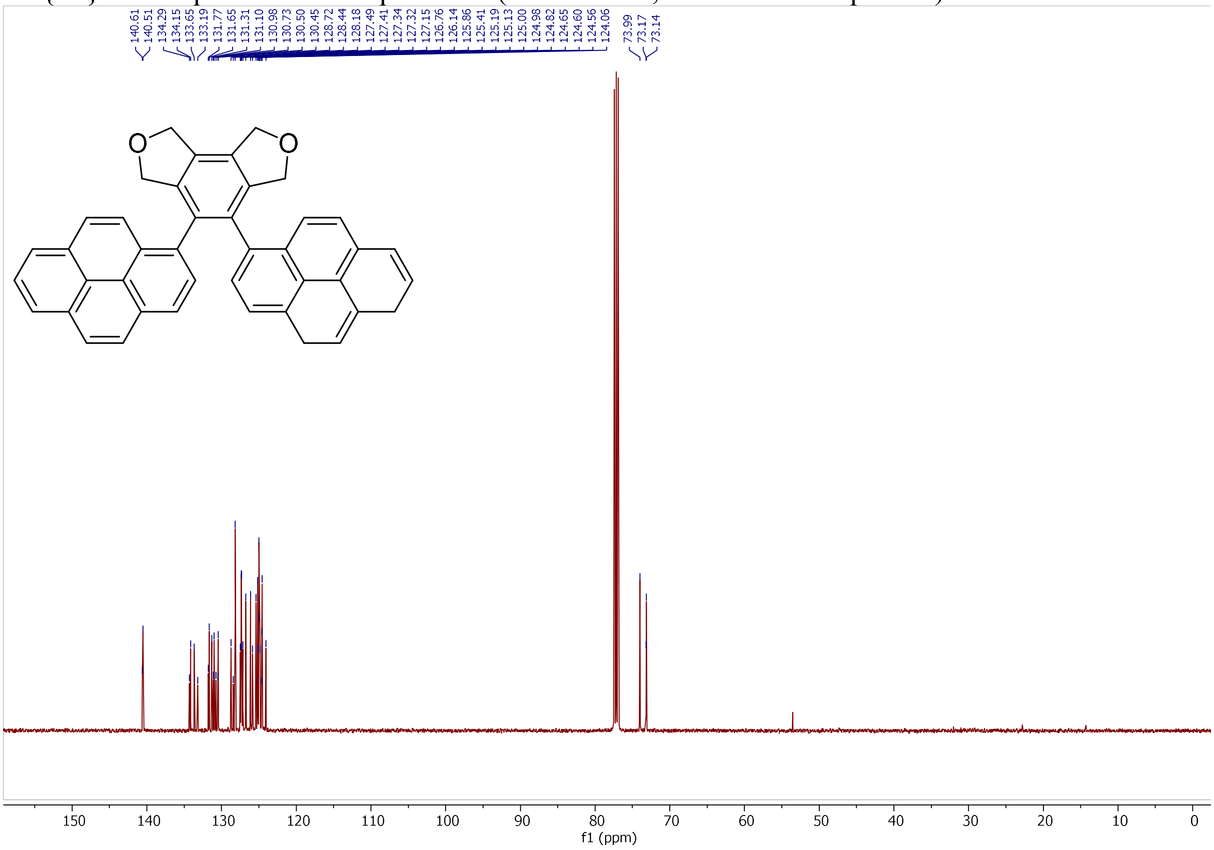

^{1}H NMR spectrum of compound **56**

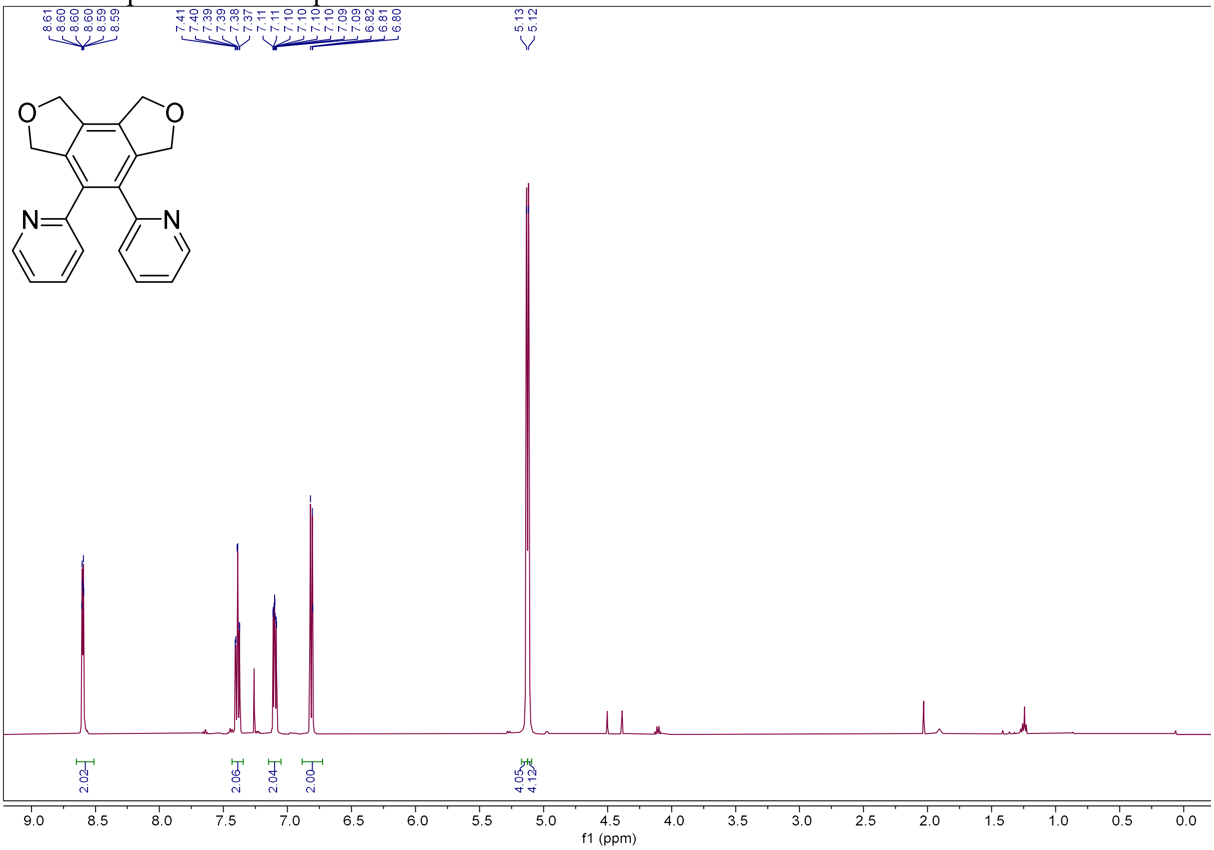

^{13}C{^{1}H} NMR spectrum of **56**

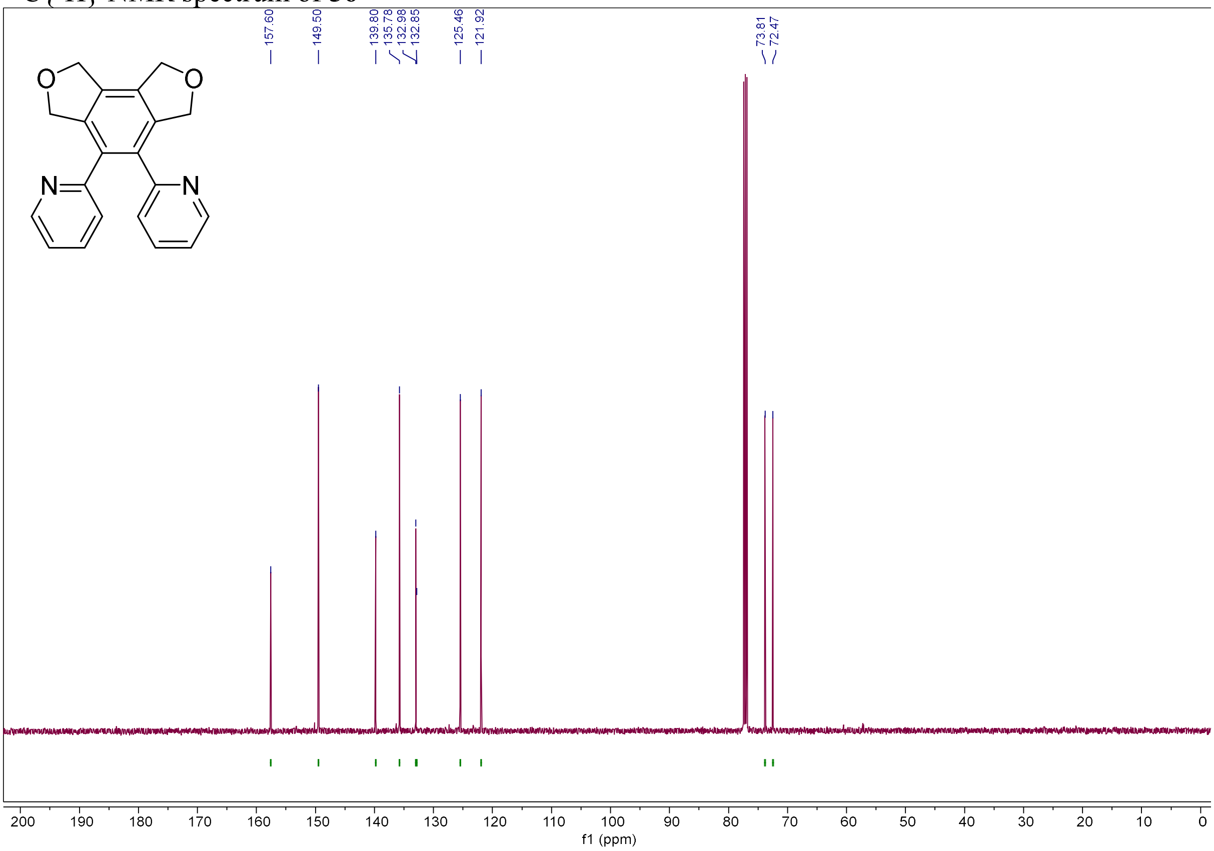

^{1}H NMR spectrum of compound **57**

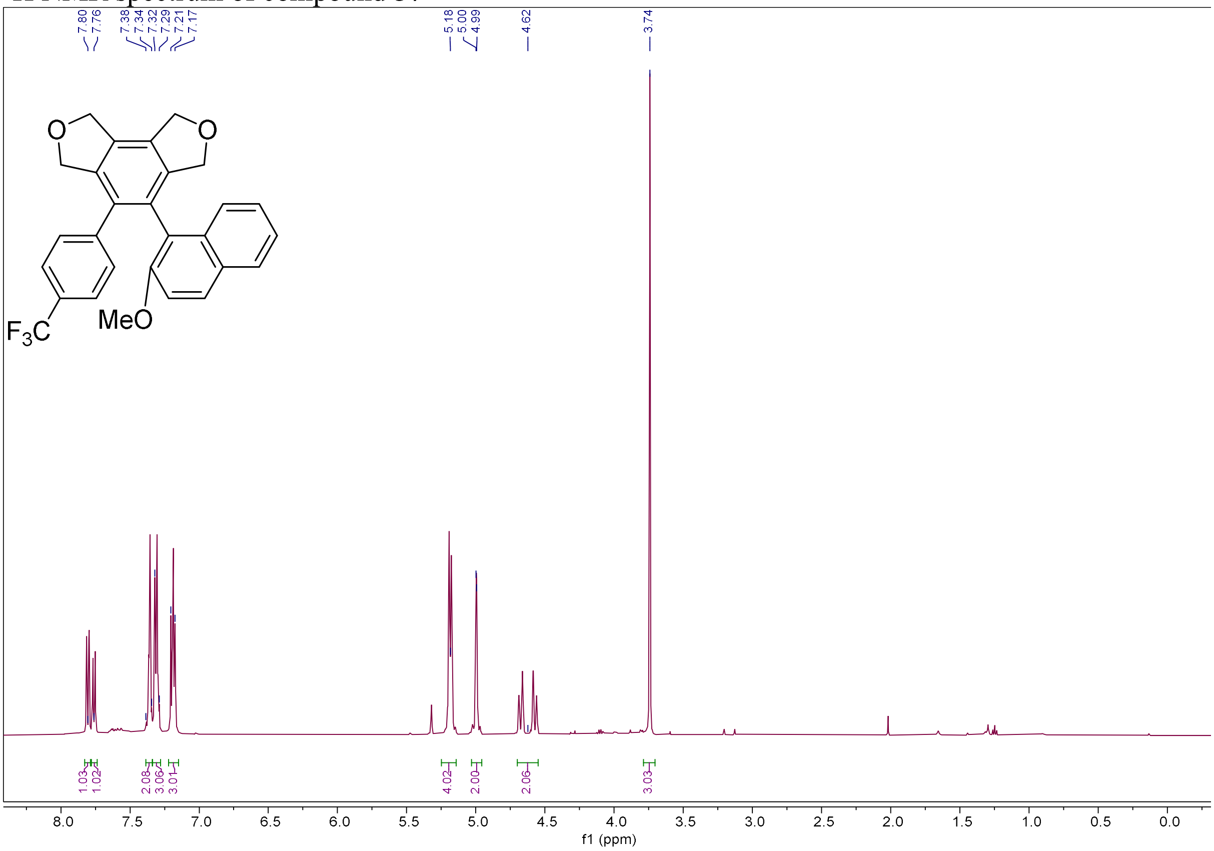

$^{13}C\{^1H\}$ NMR spectrum of **57**

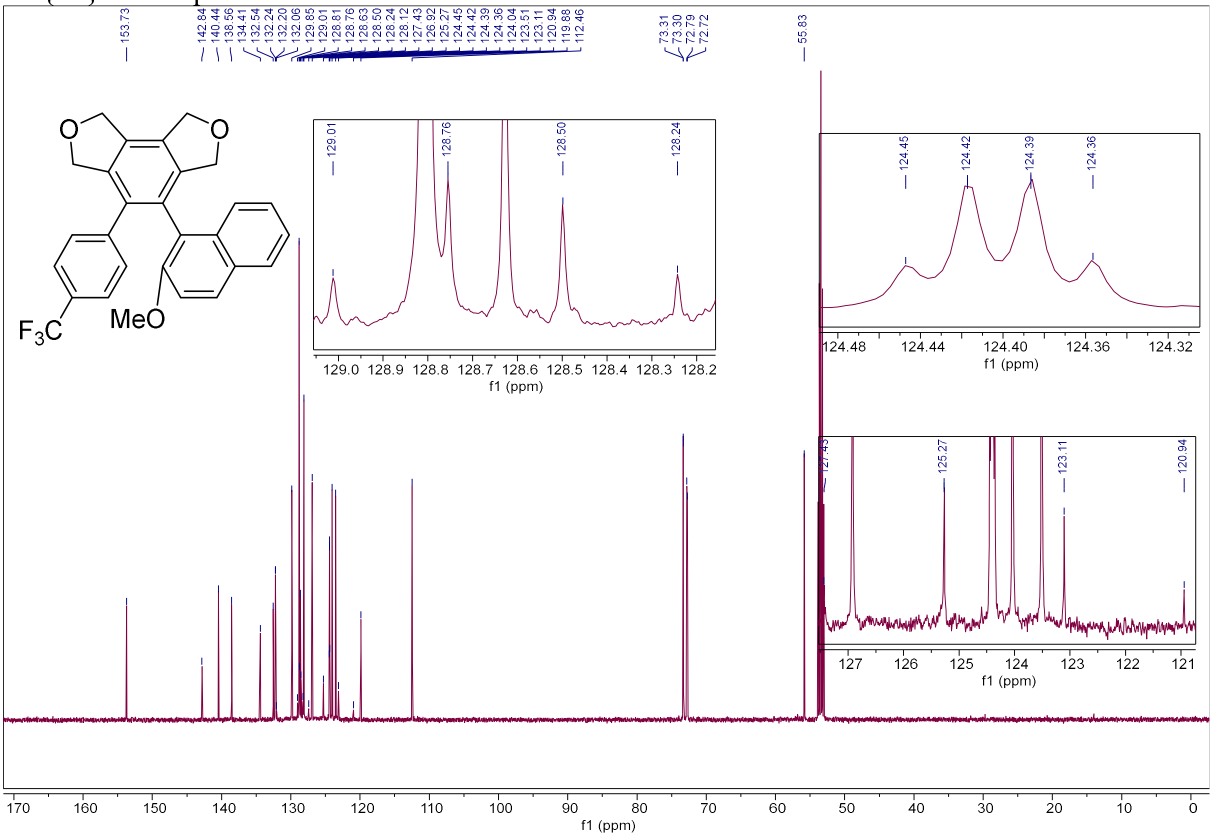

^{19}F NMR spectrum of compound **57**

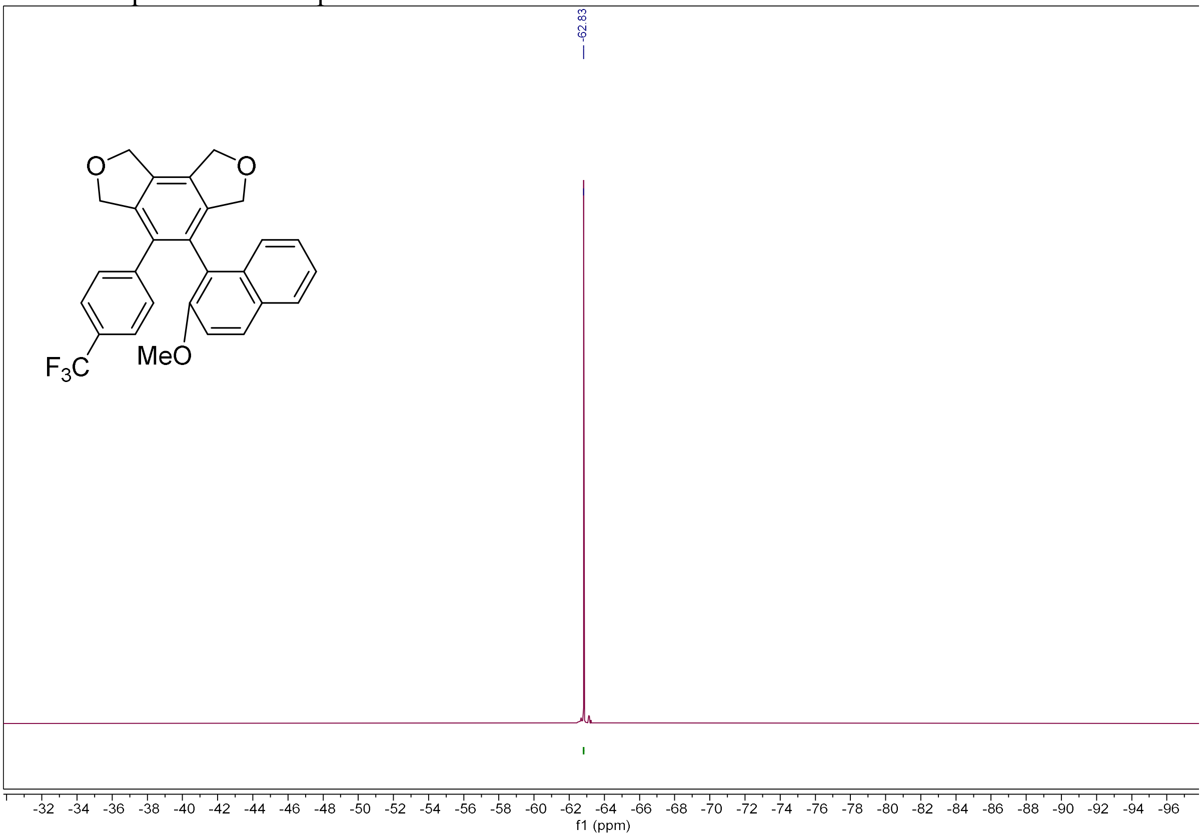

^{1}H NMR spectrum of compound **59**

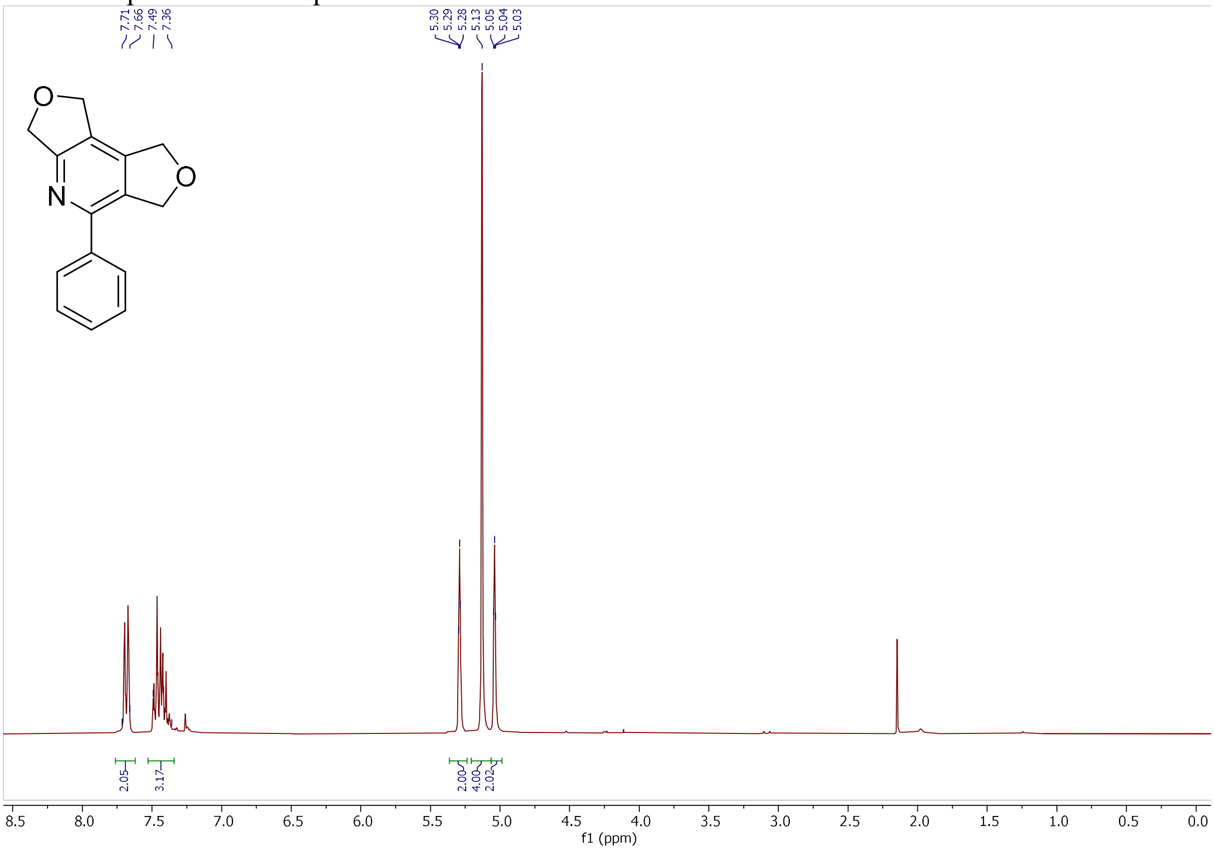

$^{13}C\{^1H\}$ NMR spectrum of **59**

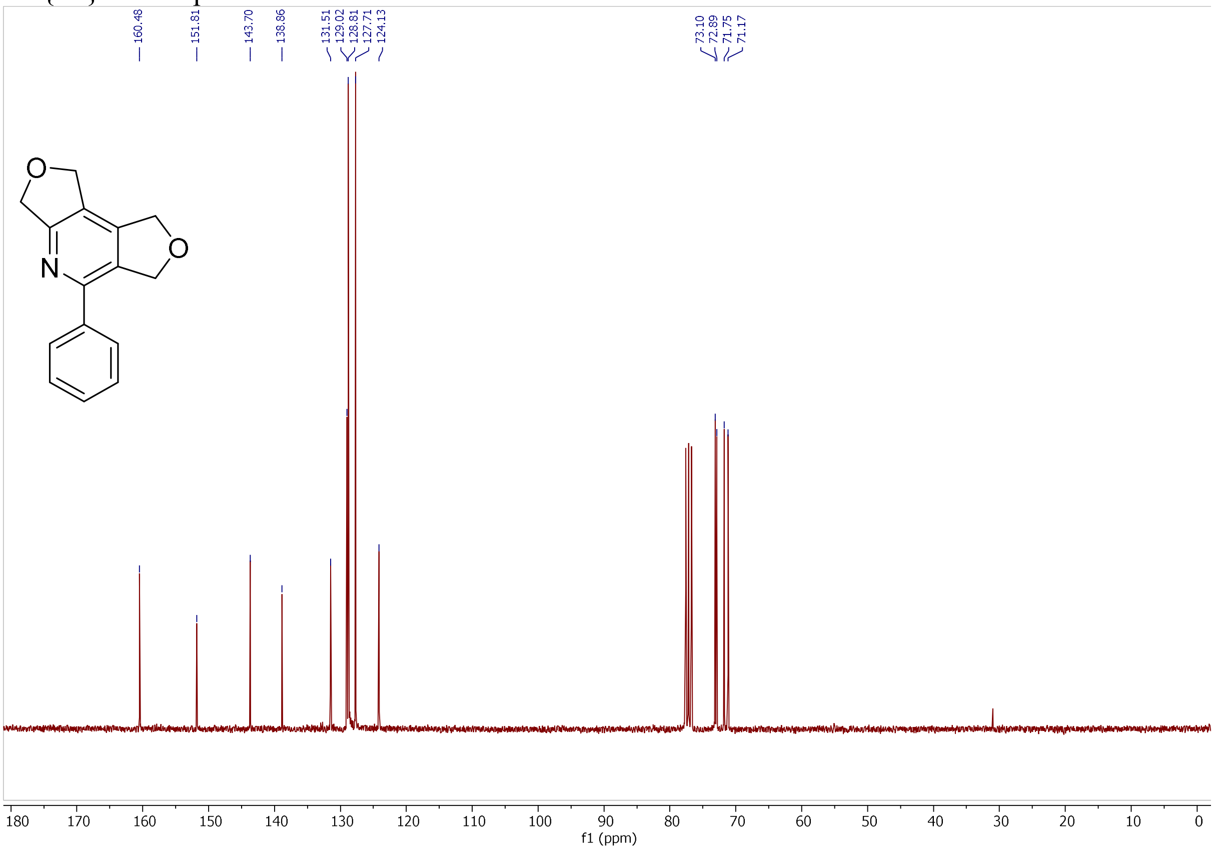

^1H NMR spectrum of compound **61**

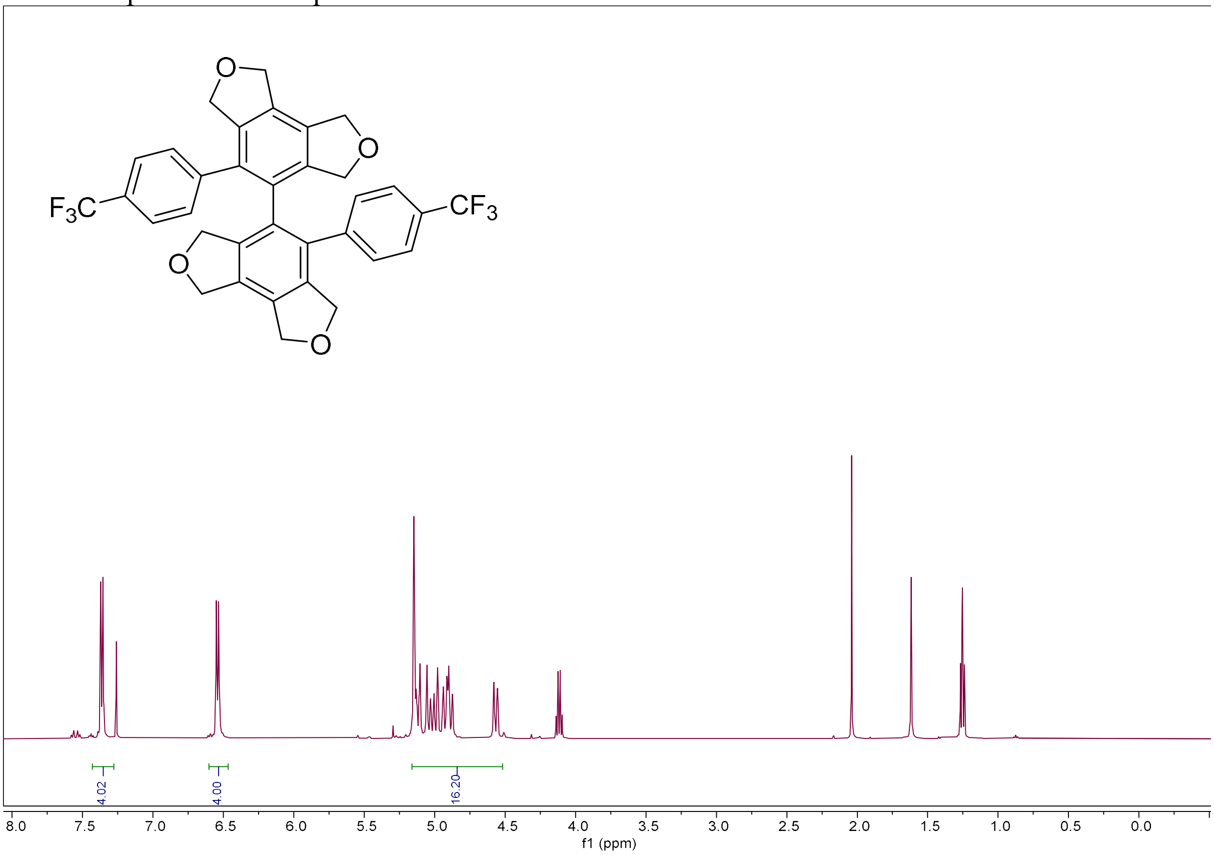

$^{13}C\{^1H\}$ NMR spectrum of **63**

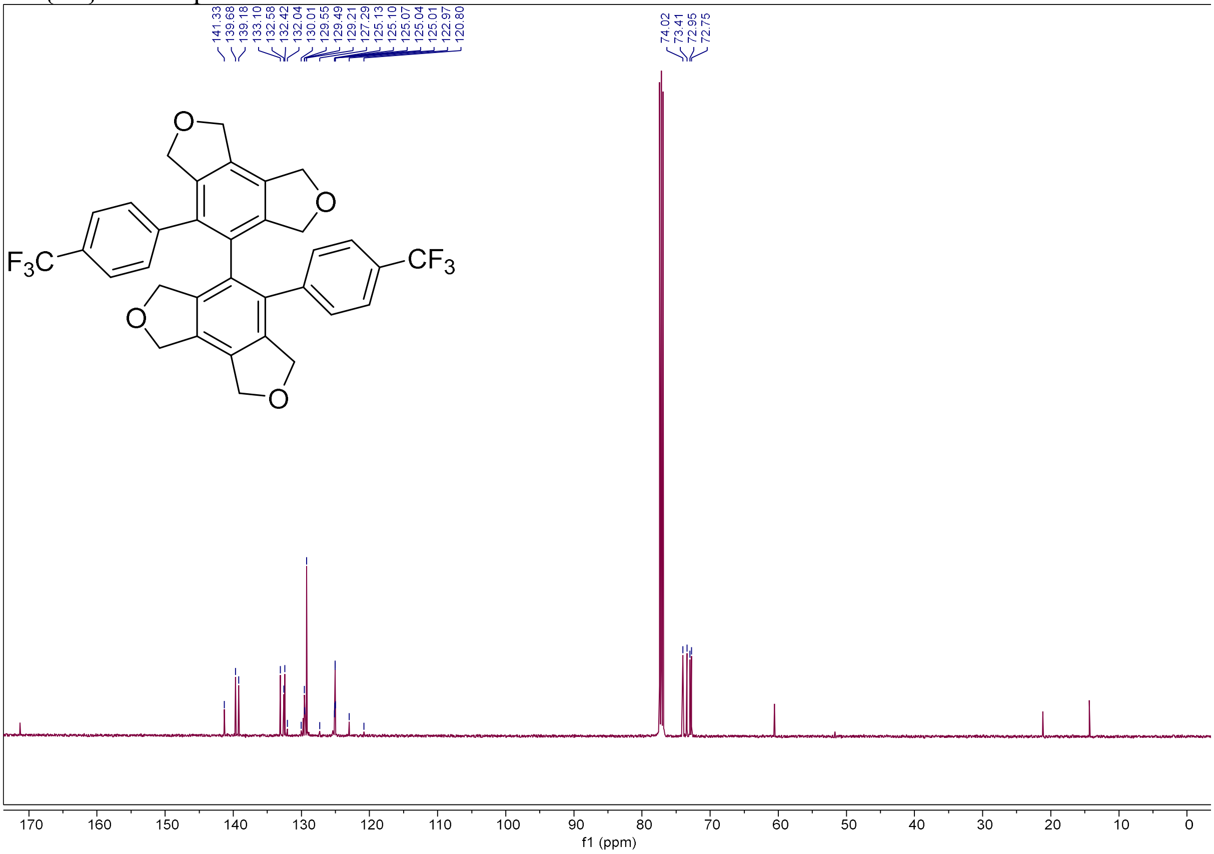

^1H NMR spectrum of compound **63**

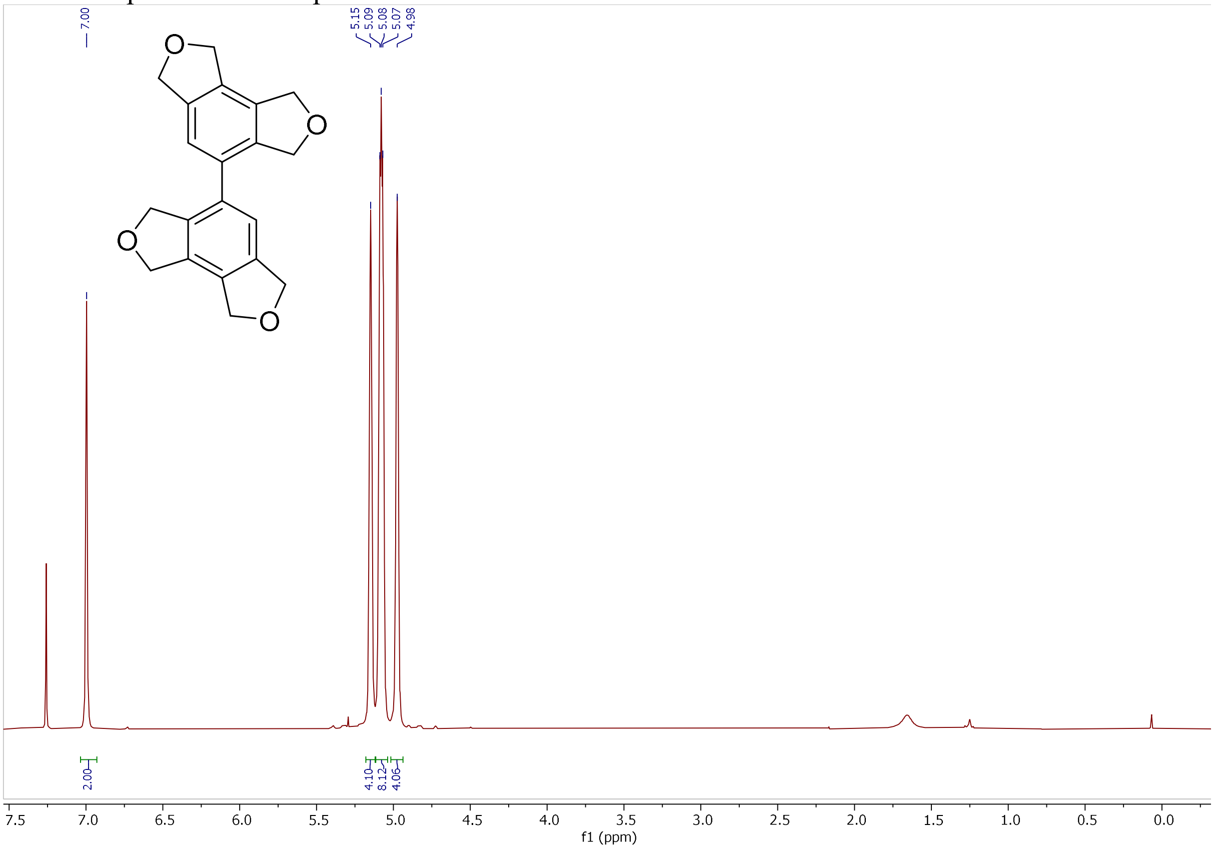

$^{13}C\{^{1}H\}$ NMR spectrum of **63**

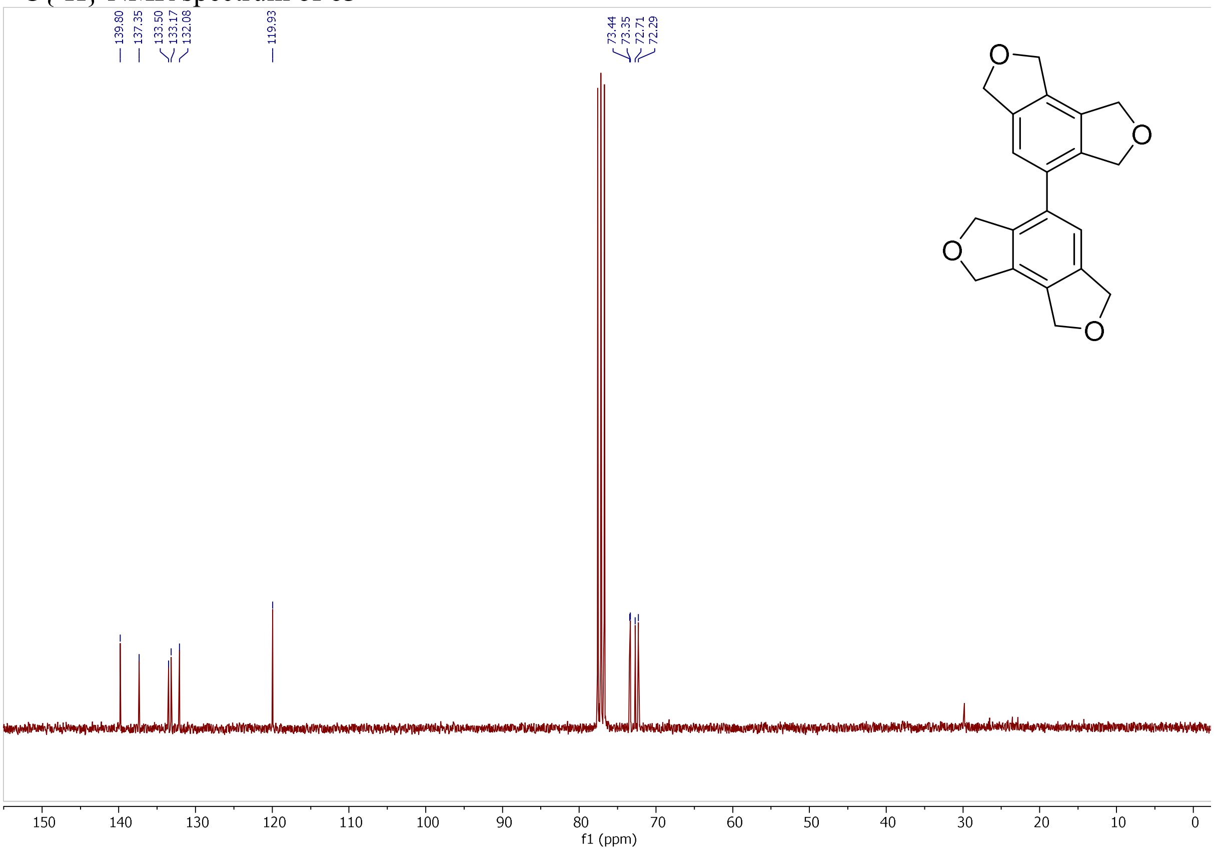

^{1}H NMR spectrum of compound **65**

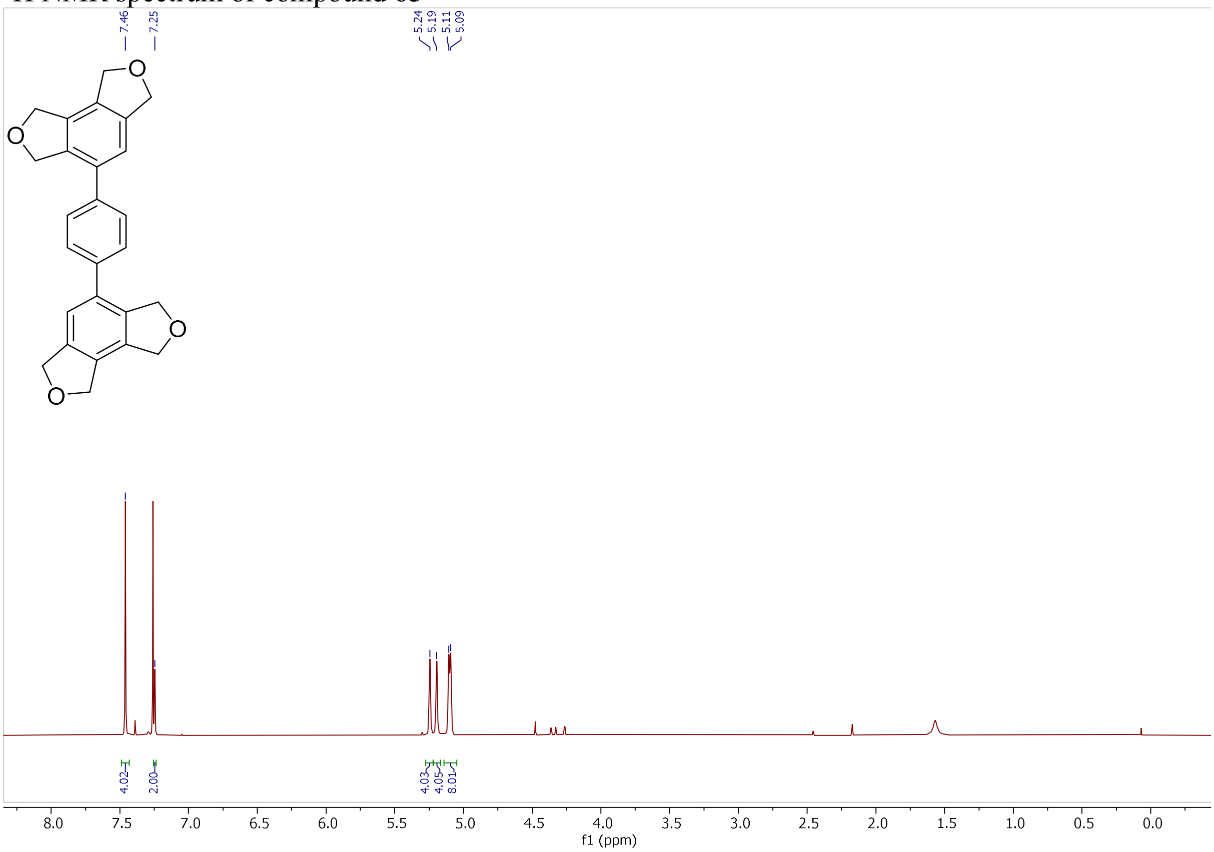

$^{13}C\{^{1}H\}$ NMR spectrum of **65**

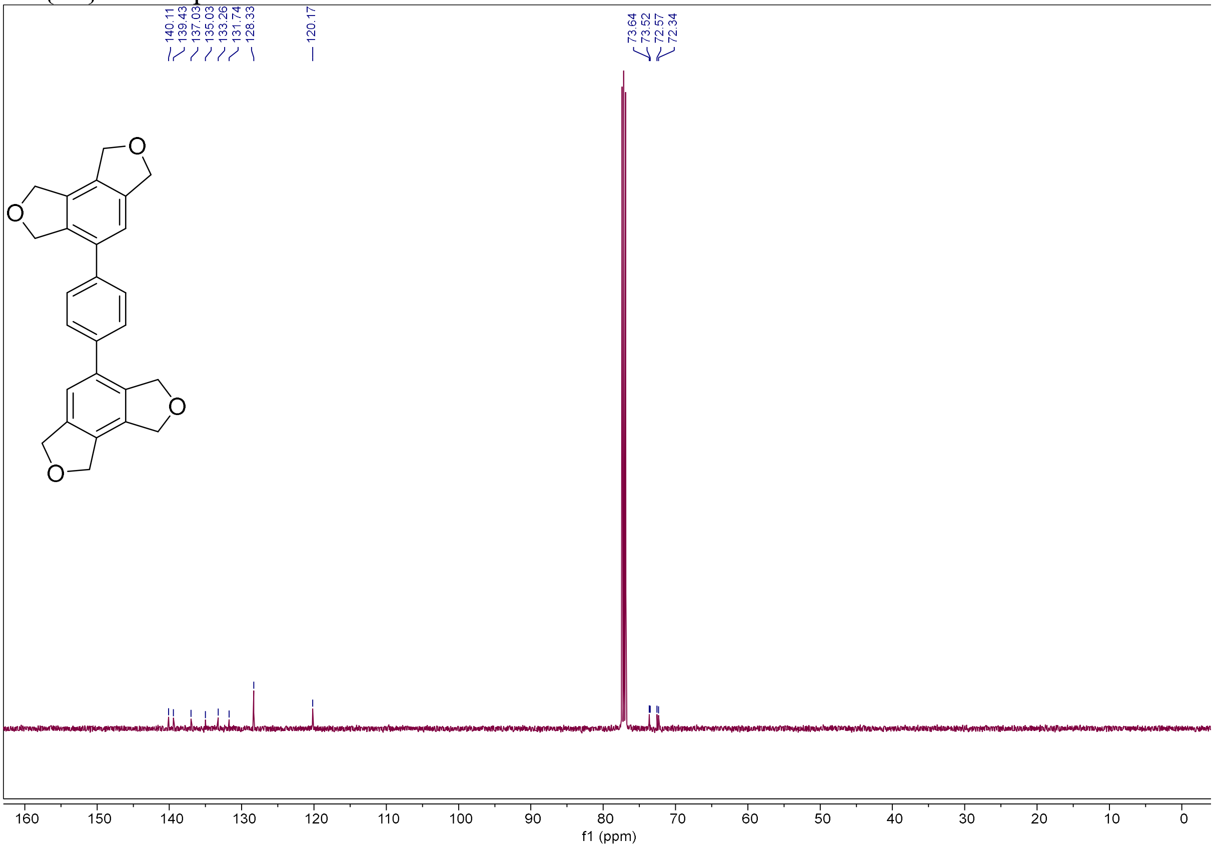

^{1}H NMR spectrum of compound **67**

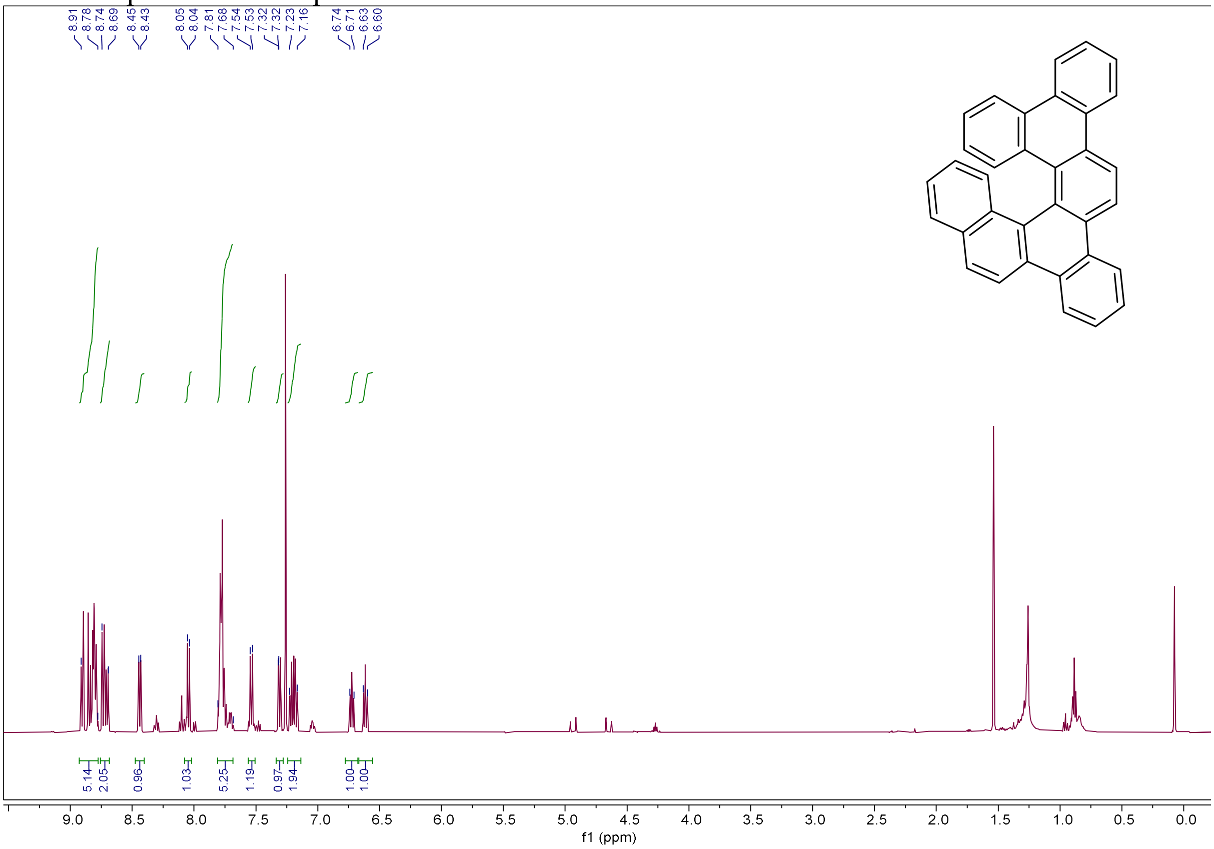

^{1}H NMR spectrum of compound **68**

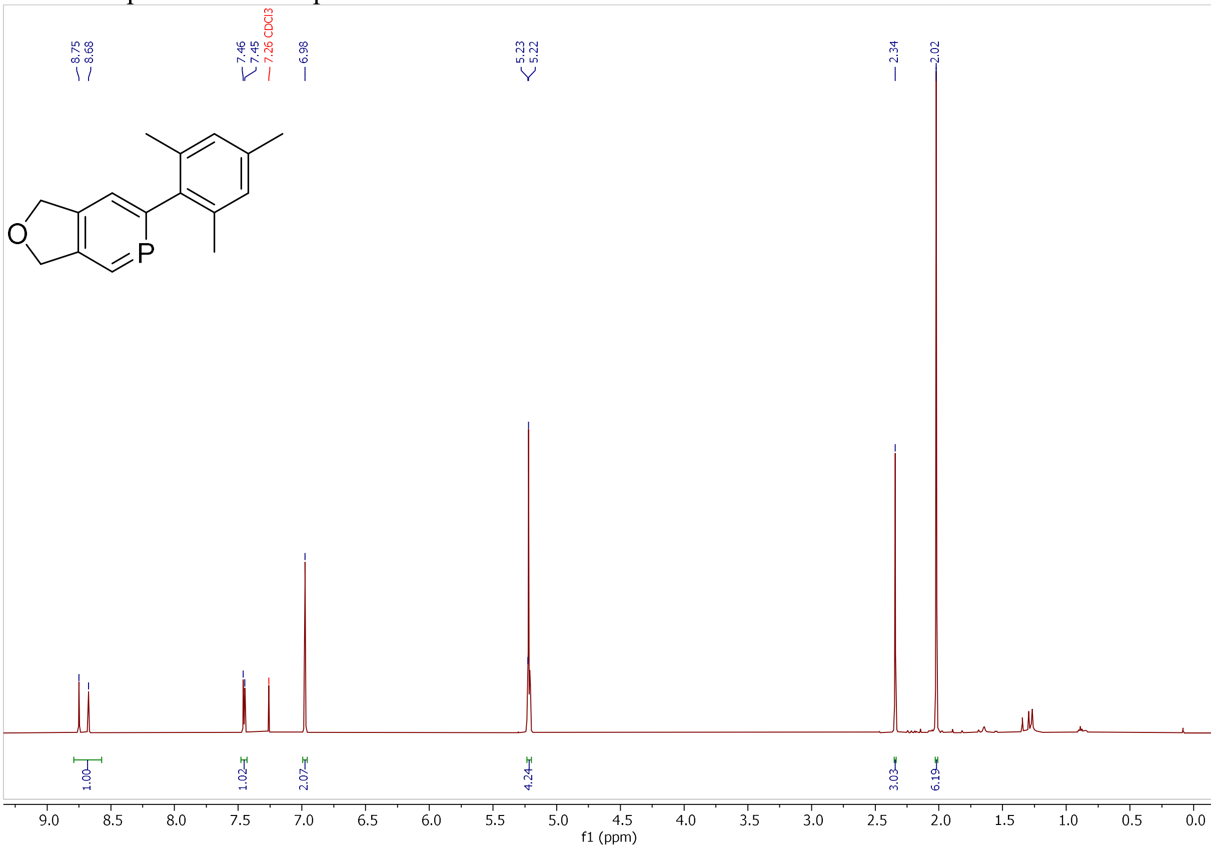

^{13}C{^{1}H} NMR spectrum of **68**

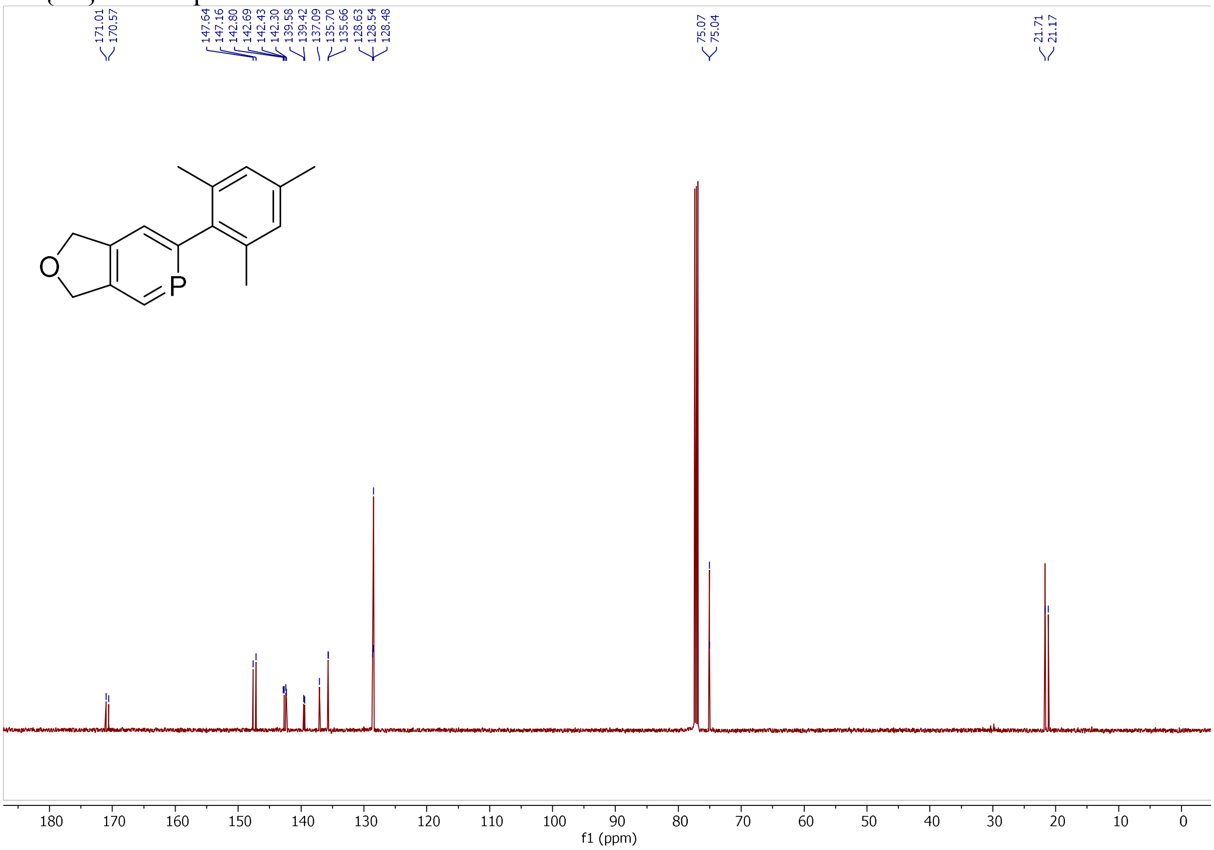

$^{31}P\{^{1}H\}$ NMR spectrum of compound **68**

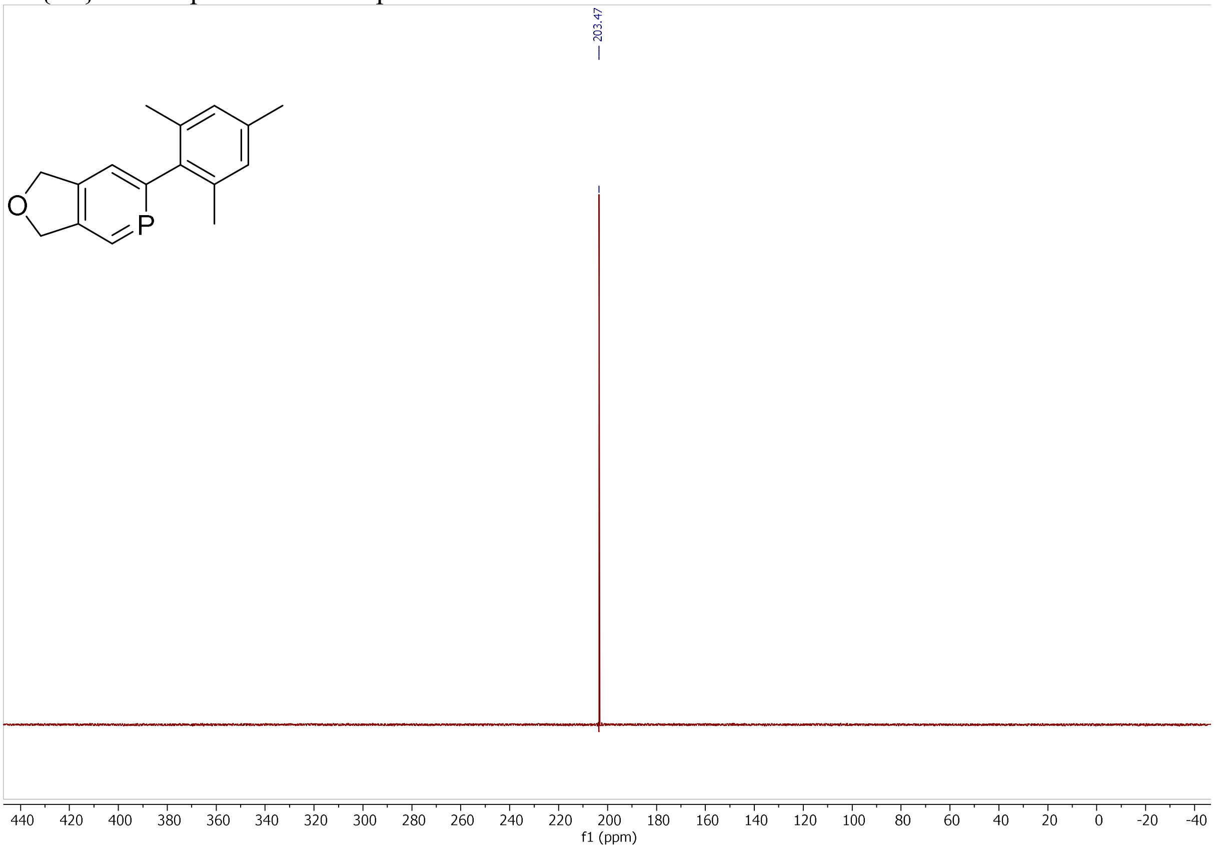

^{1}H NMR spectrum of compound **70**

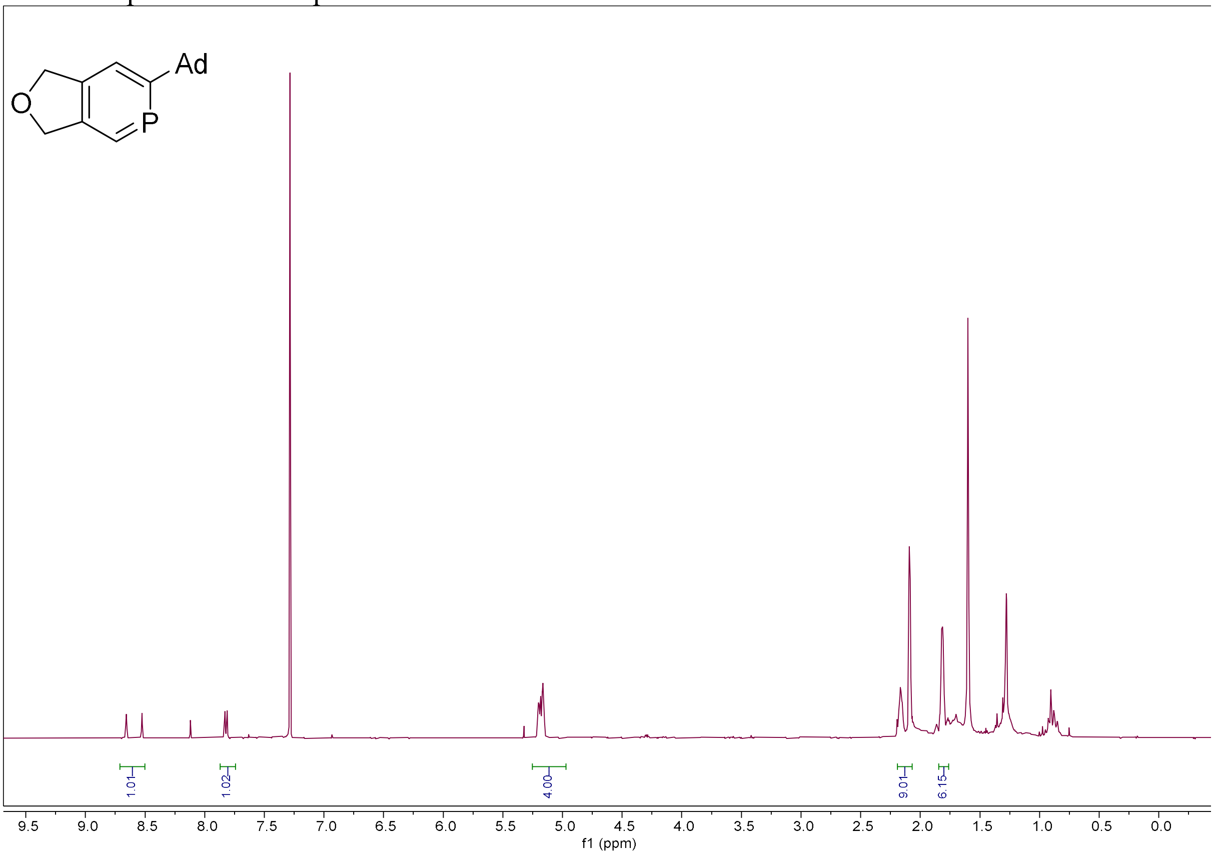

$^{31}P\{^1H\}$ NMR spectrum of compound **70**

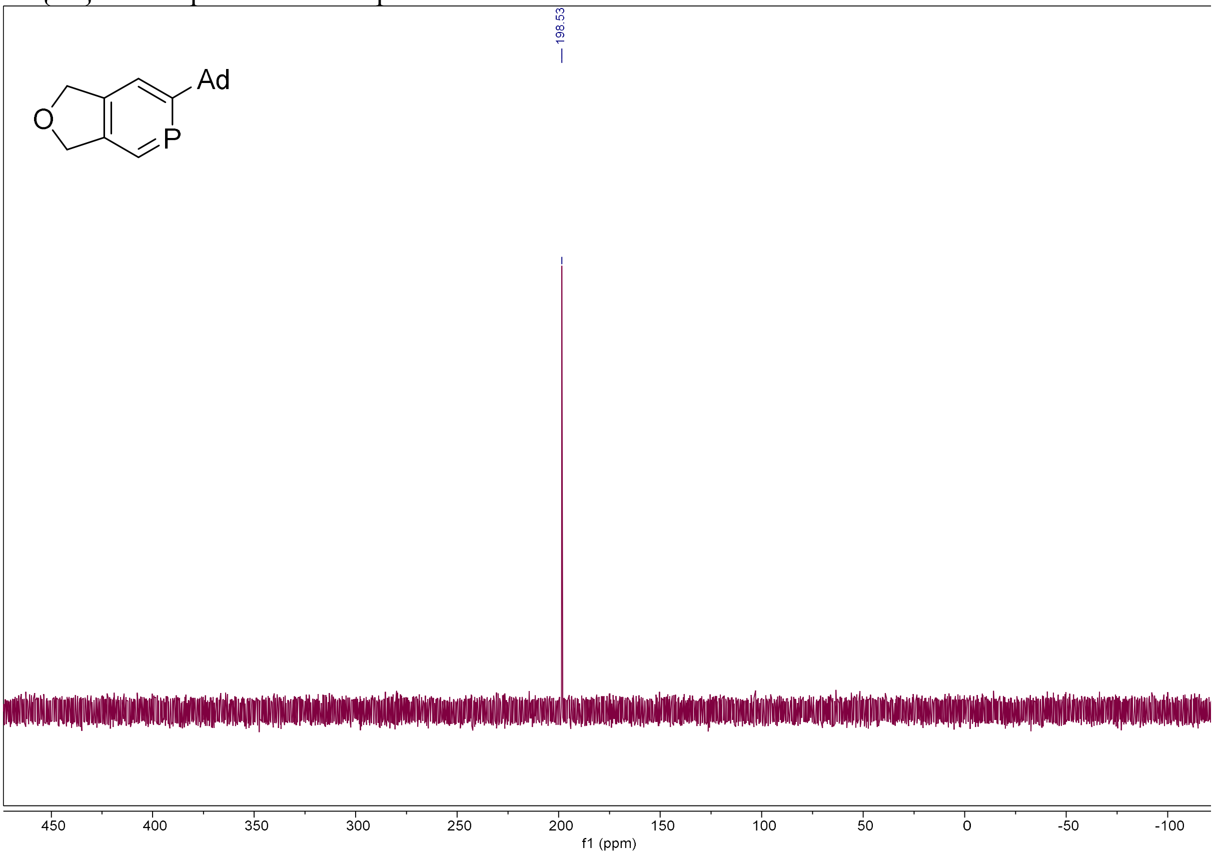

^1H NMR spectrum of compound **71a** and **71b**

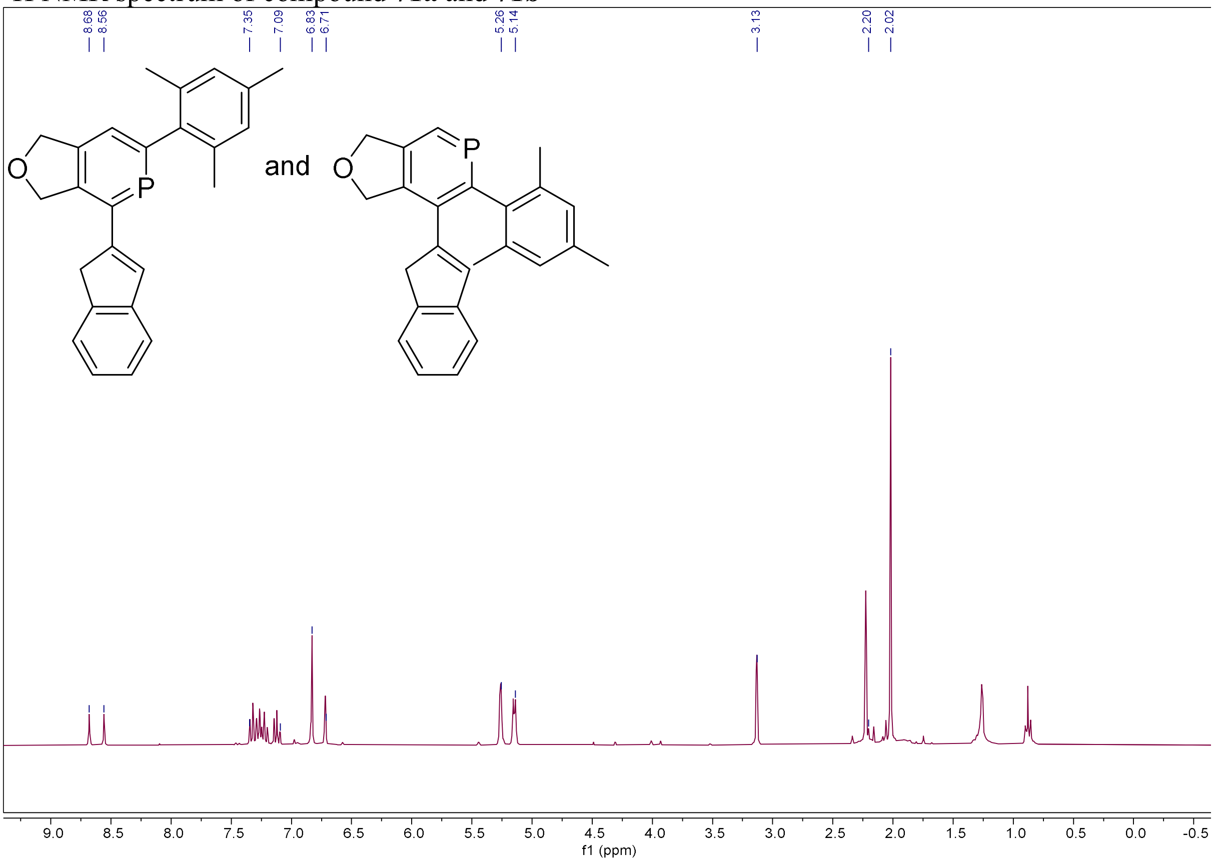

$^{13}C\{^1H\}$ NMR spectrum of **71a** and **71b**

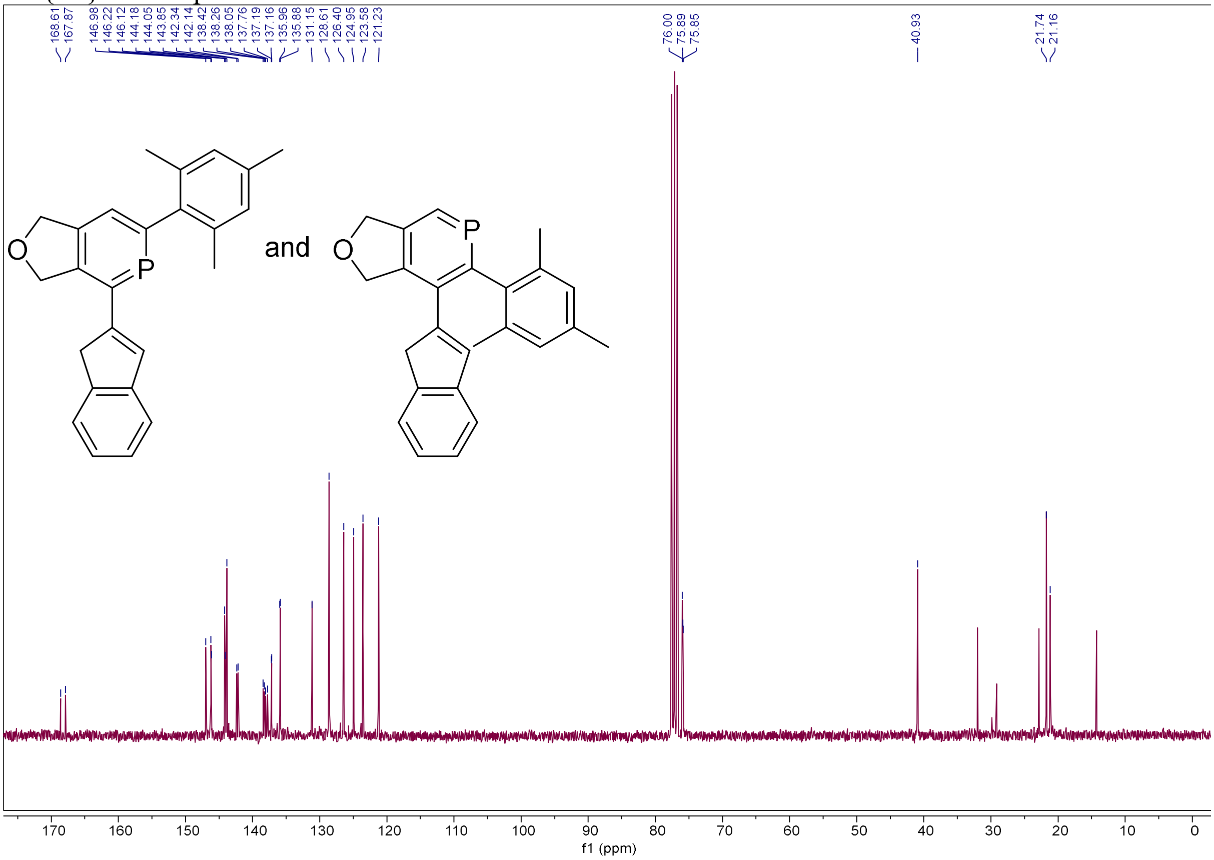

$^{31}P\{^1H\}$ NMR spectrum of compound **71a** and **71b**

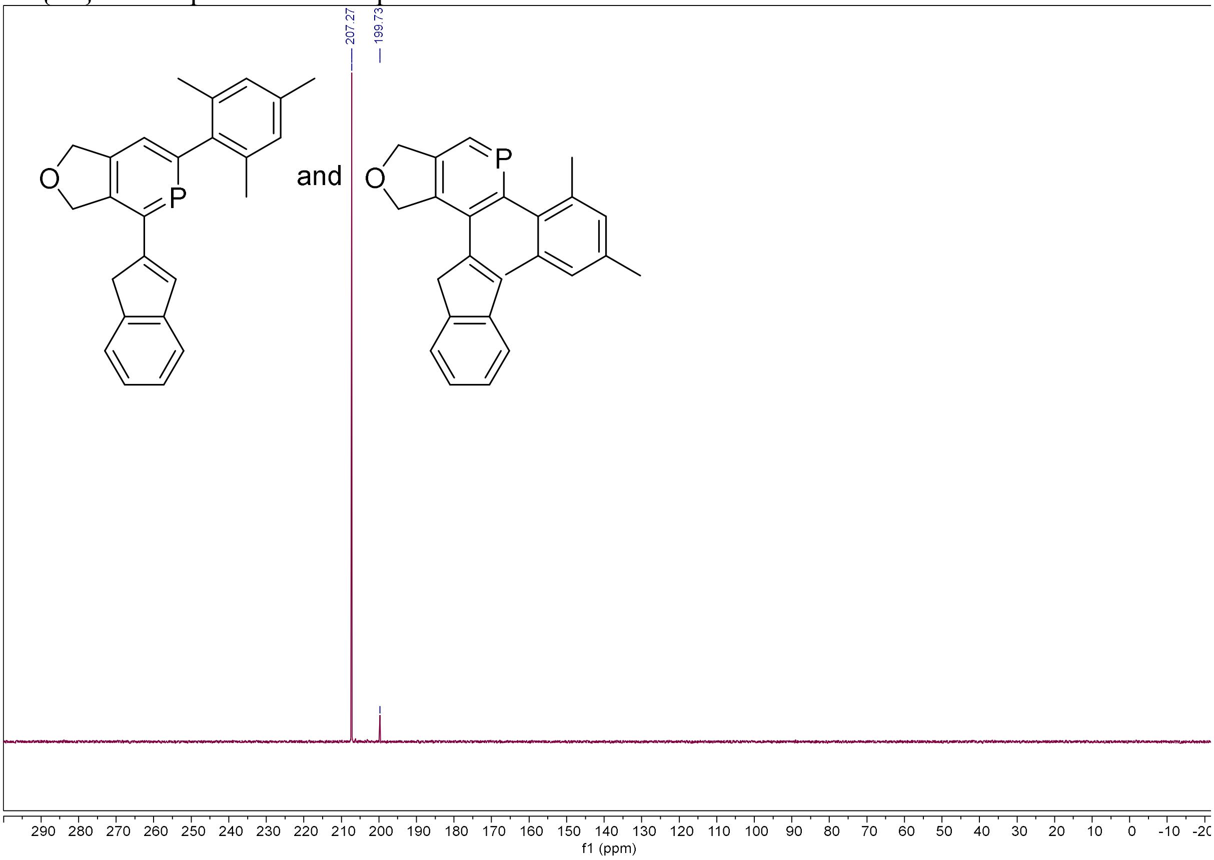

^{1}H NMR spectrum of compound **72**

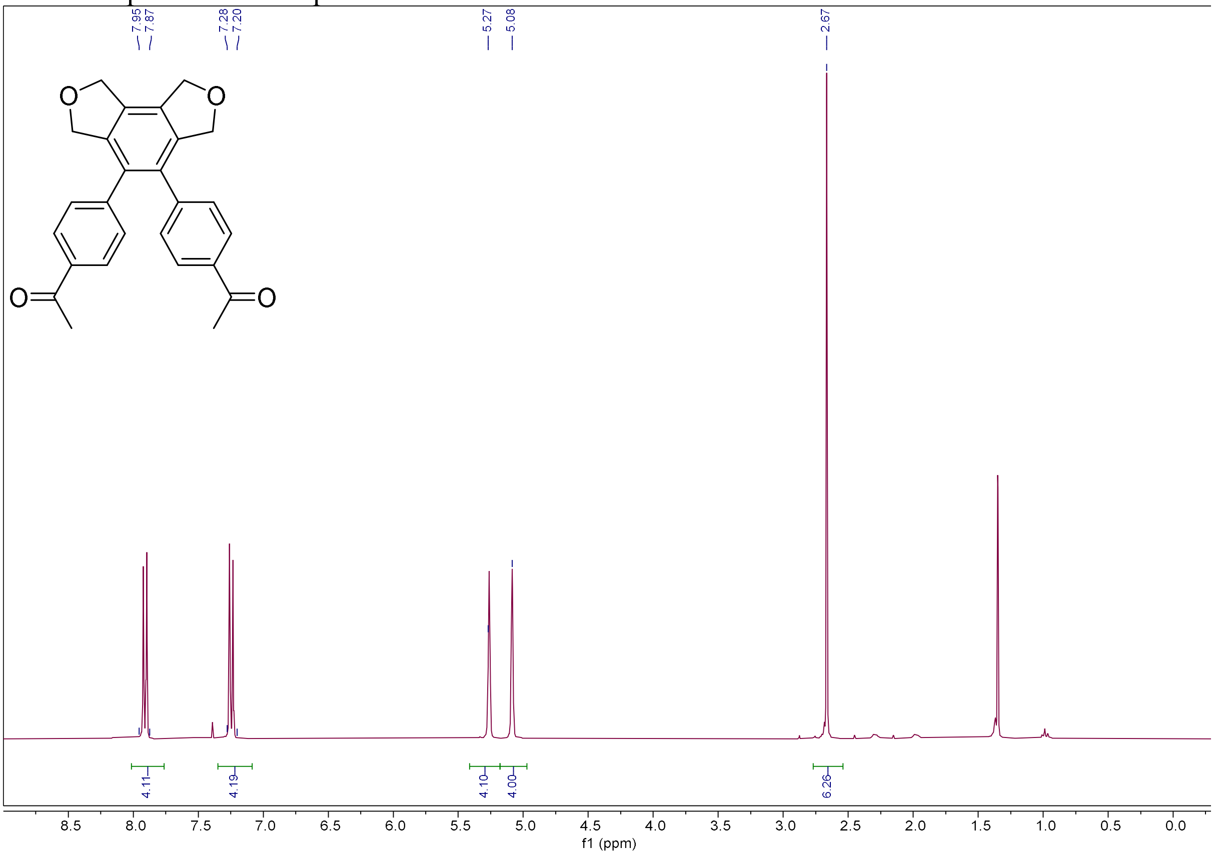

^{13}C{^{1}H} NMR spectrum of **72**

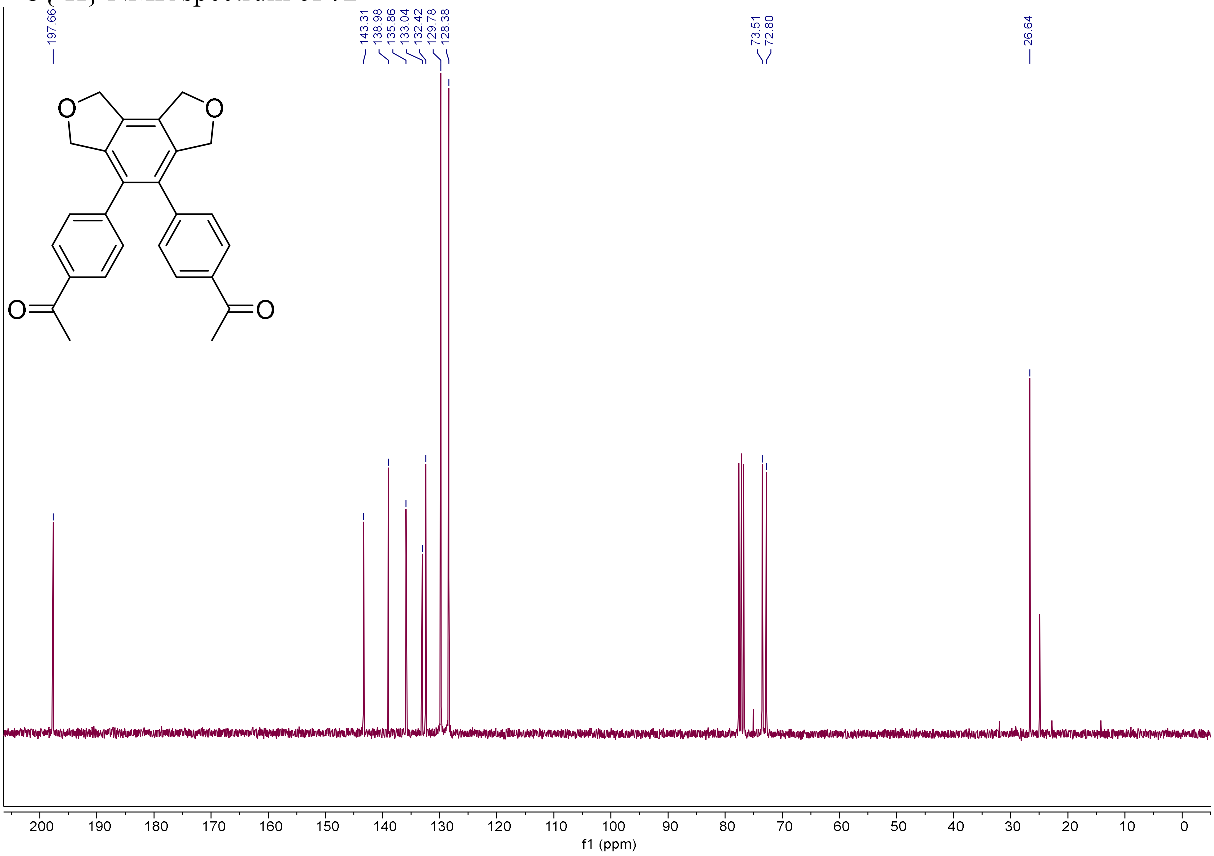

^{1}H NMR spectrum of compound **73**

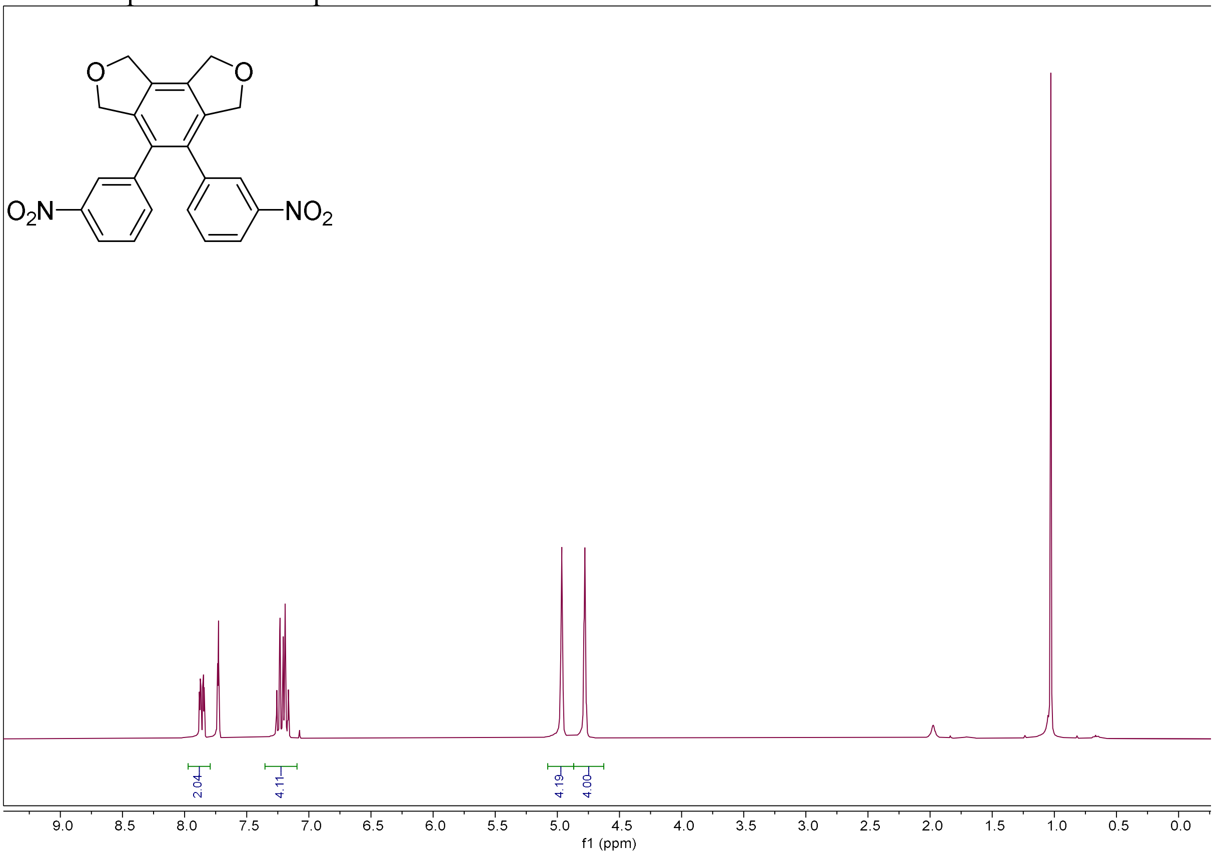

^{13}C{^{1}H} NMR spectrum of **73**

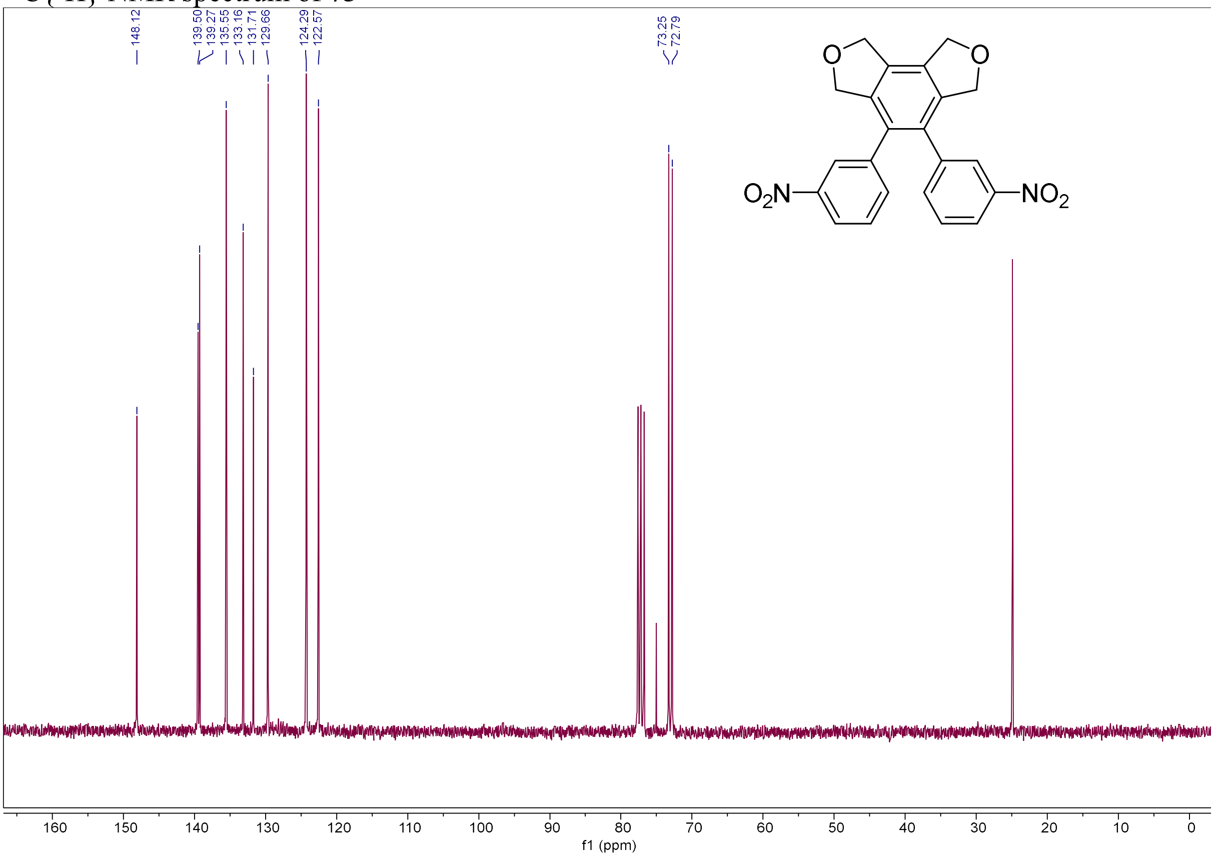

^{1}H NMR spectrum of **74** and **75**

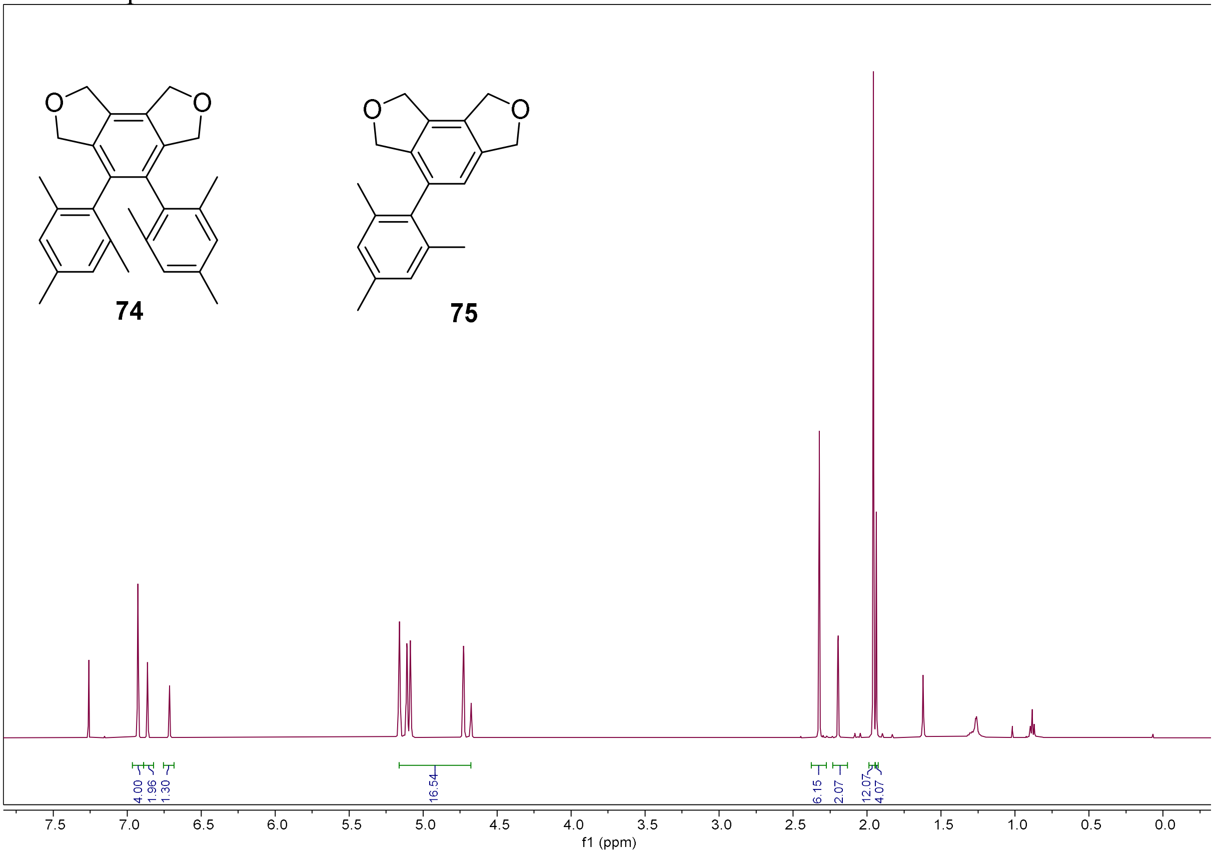

^{13}C{^{1}H} NMR spectrum of **74** and **75**

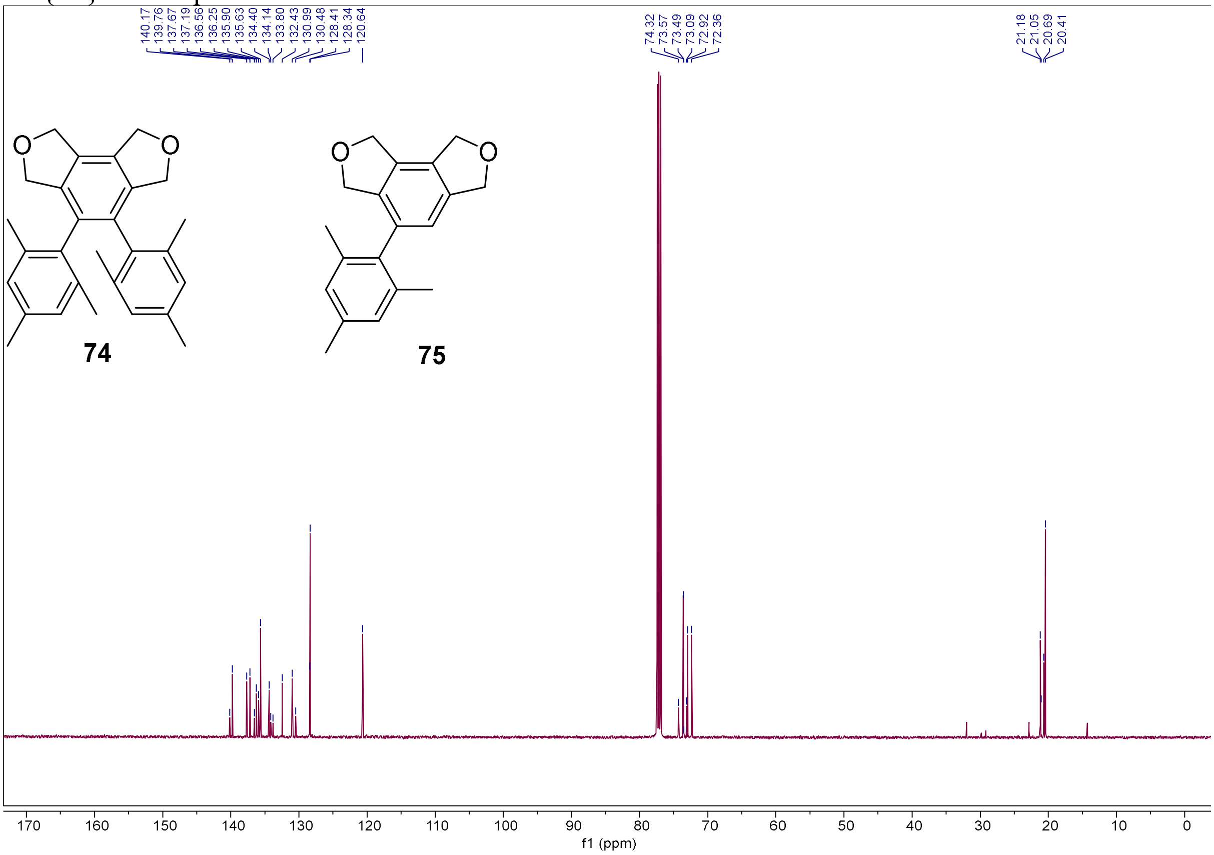

^{1}H NMR spectrum of **76**

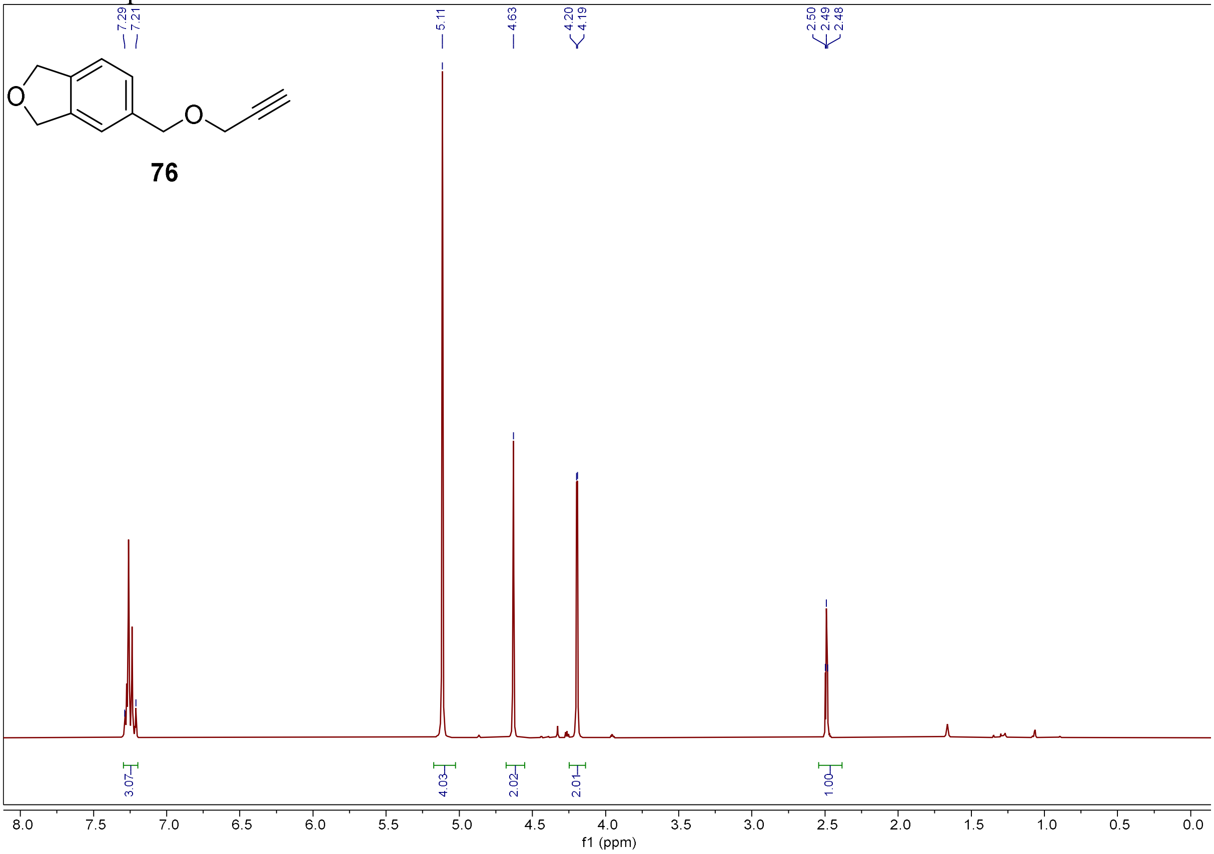

^{13}C{^{1}H} NMR spectrum of **76**

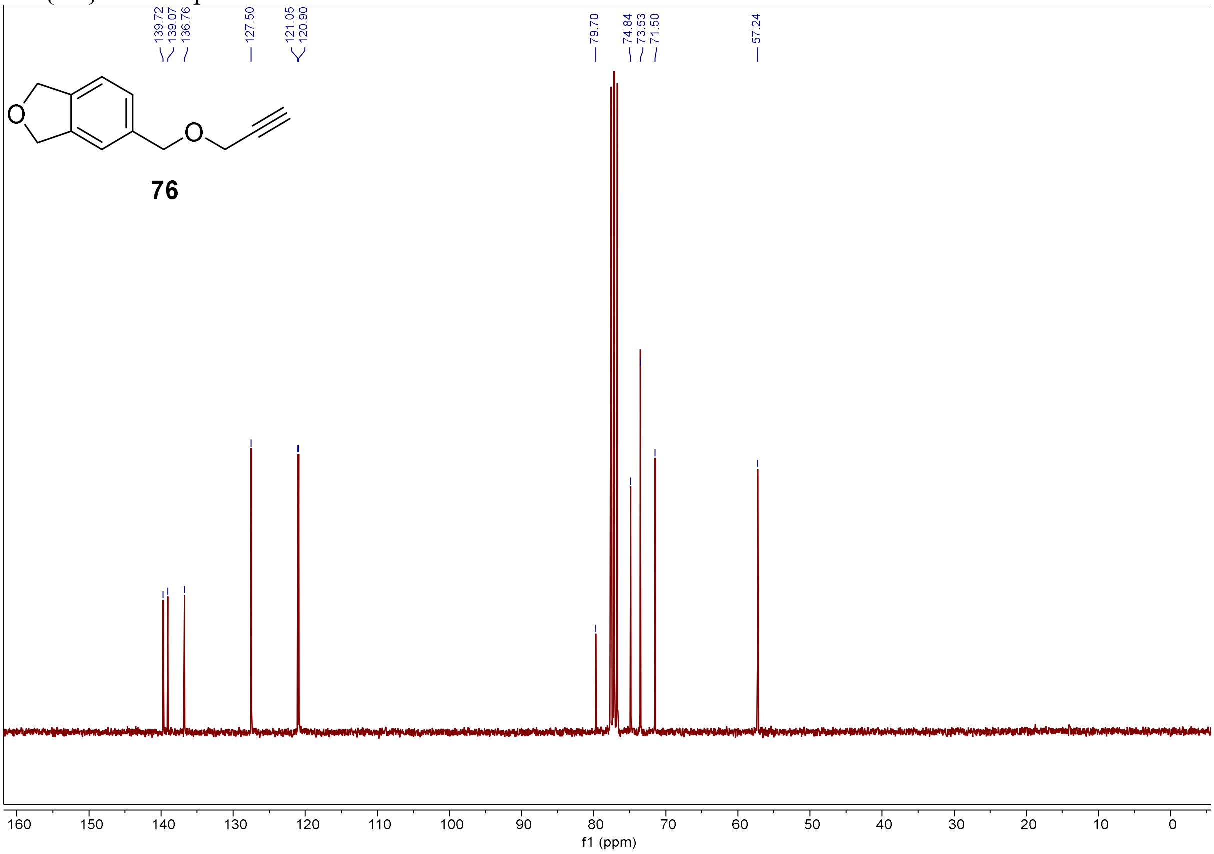

^{1}H NMR spectrum of **77** and **78**

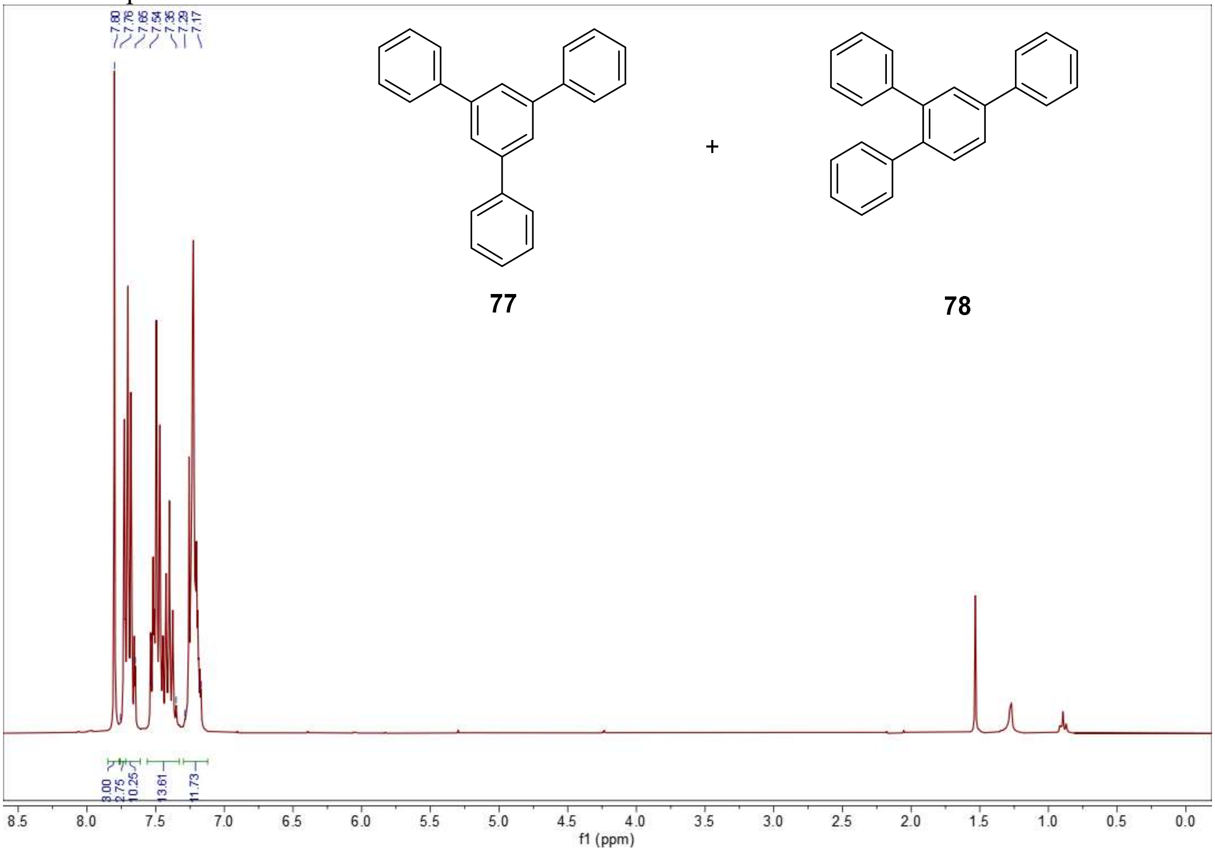

^{13}C{^{1}H} NMR spectrum of **77** and **78**

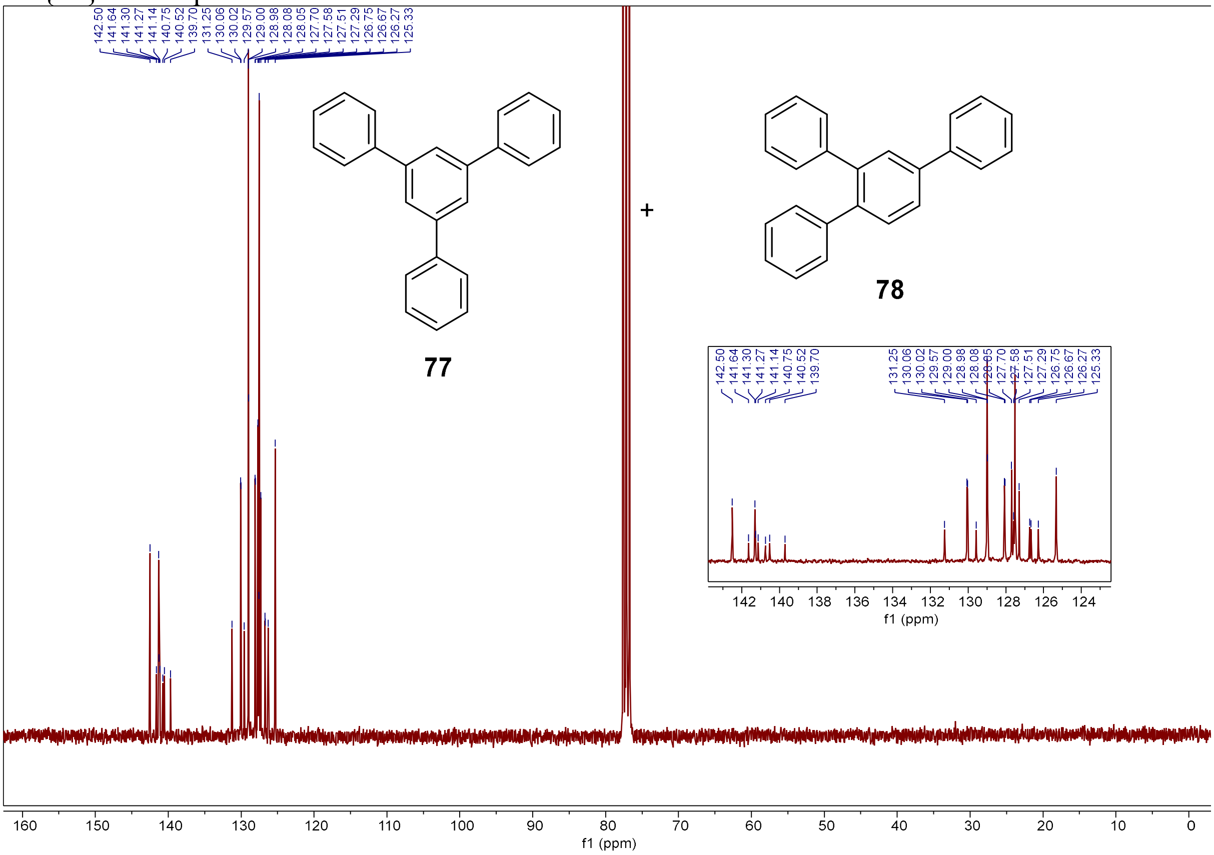

3.3 NMR spectra of new substrates and reported compounds

^{1}H NMR spectrum of compound **4**

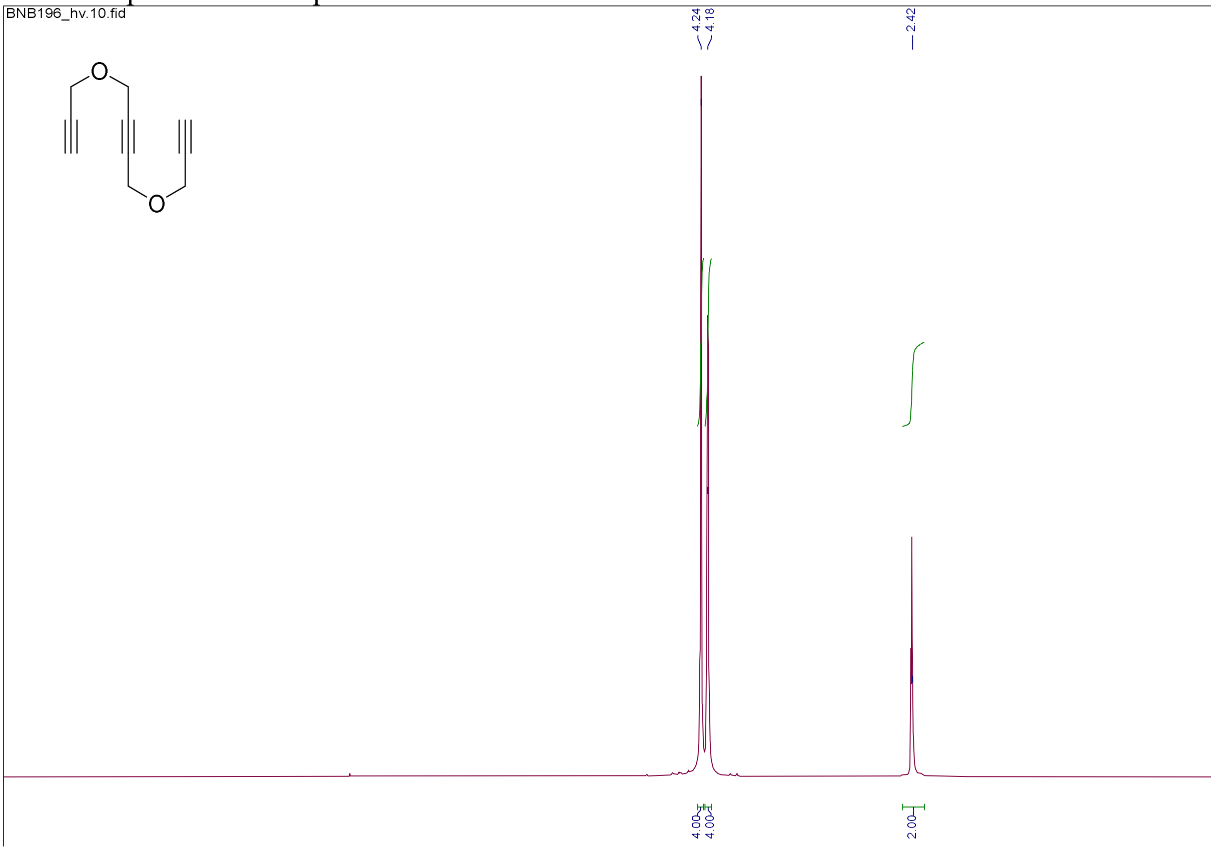

^{1}H NMR spectrum of compound **7**

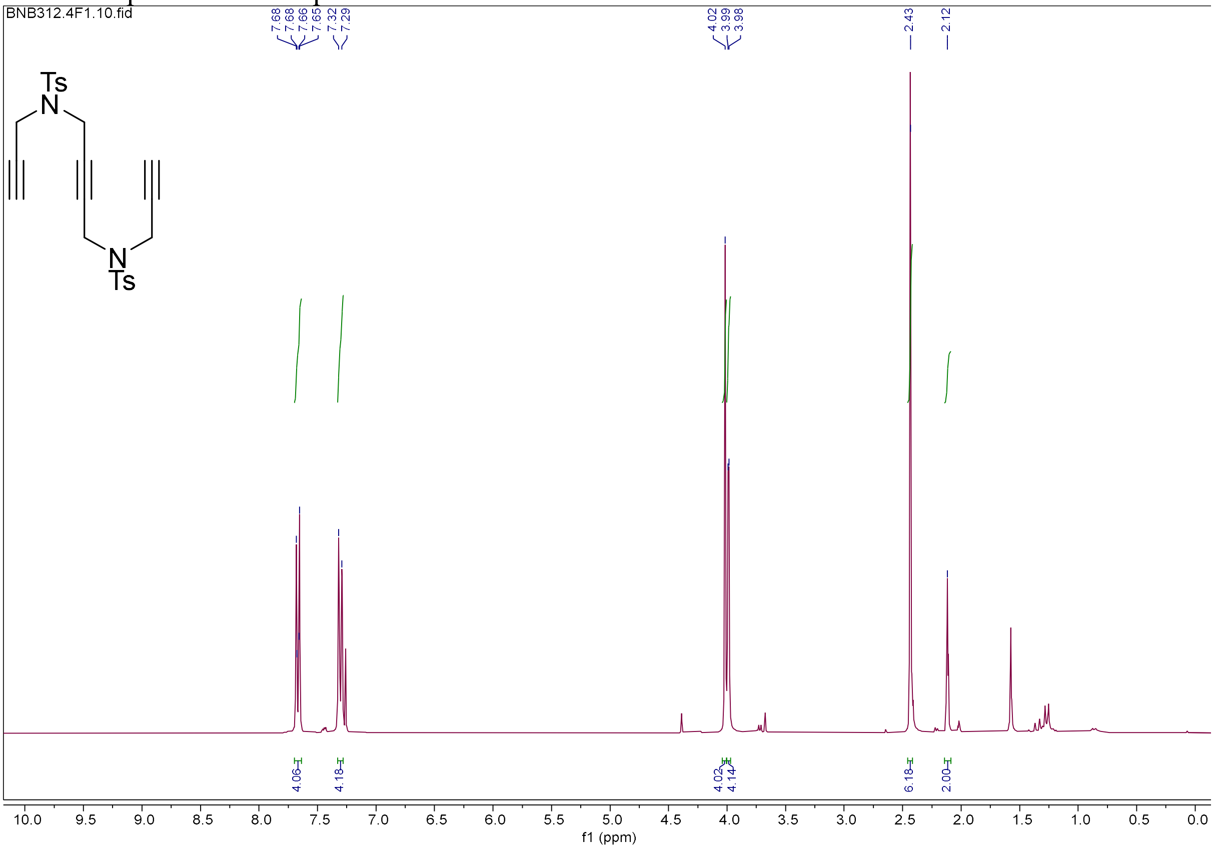

^{1}H NMR spectrum of compound **8**

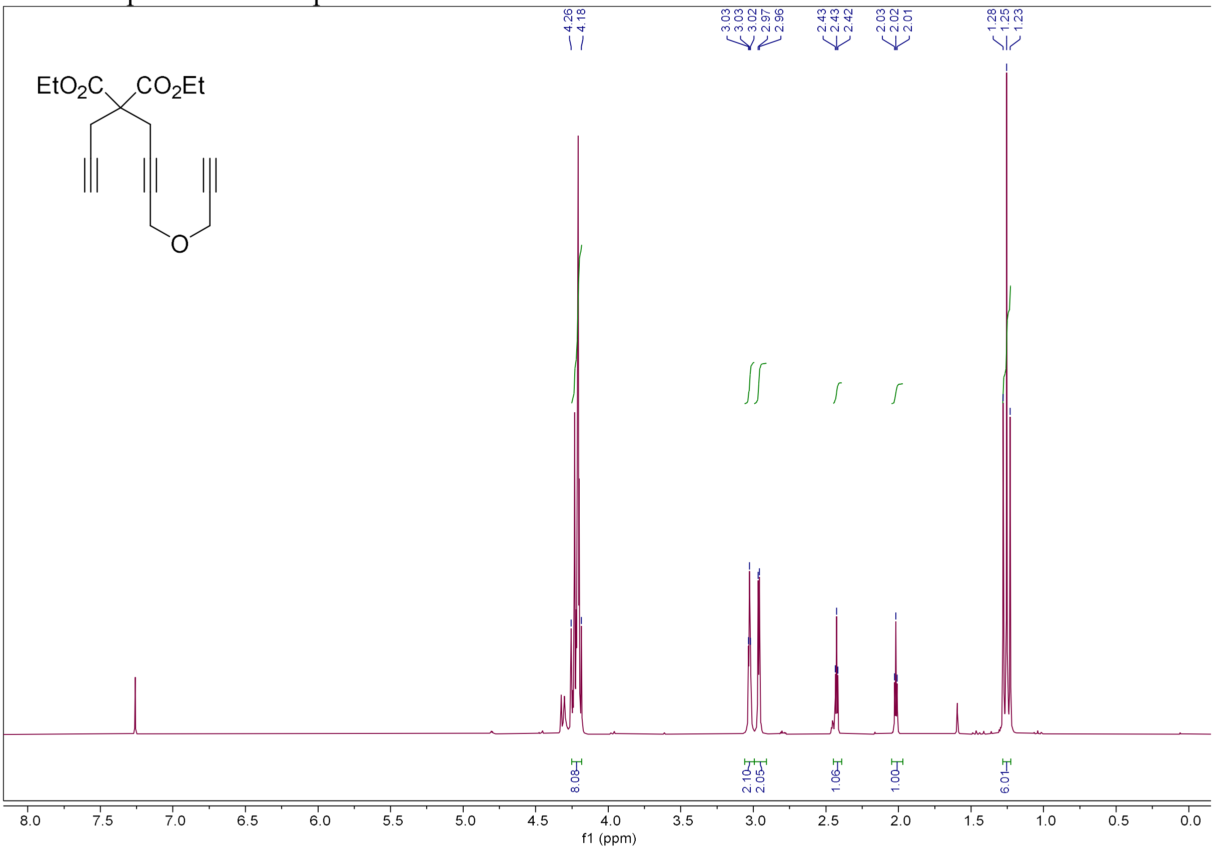

^{1}H NMR spectrum of compound **9**

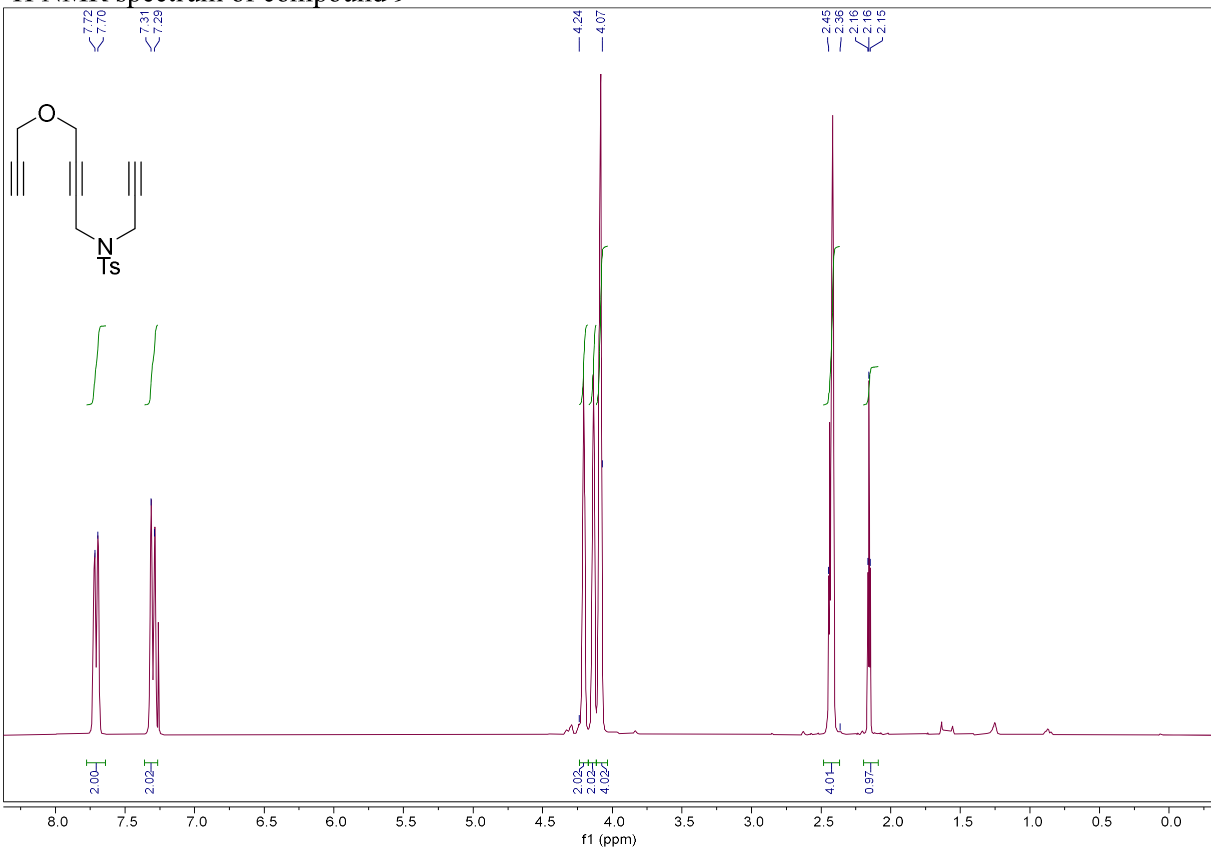

^{1}H NMR spectrum of compound **10**

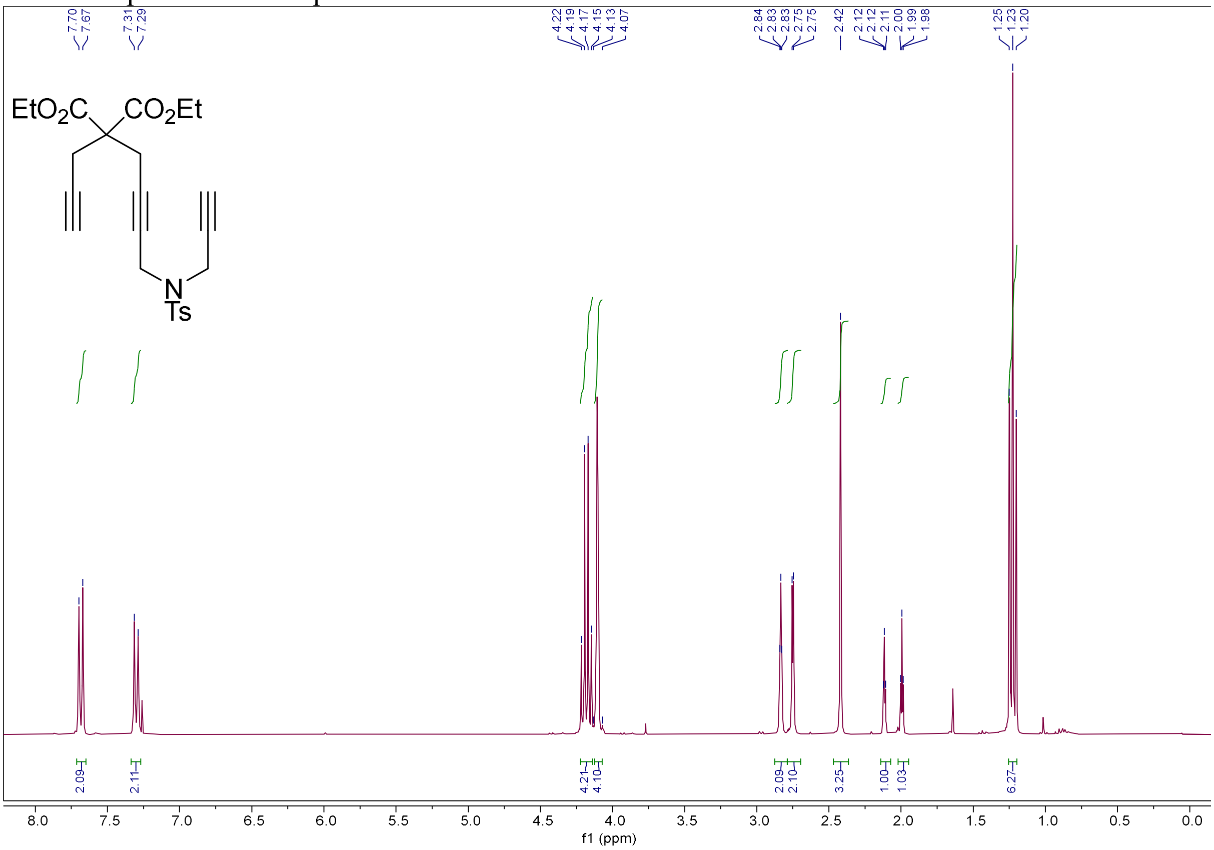

^{13}C{^{1}H} NMR spectrum of **10**

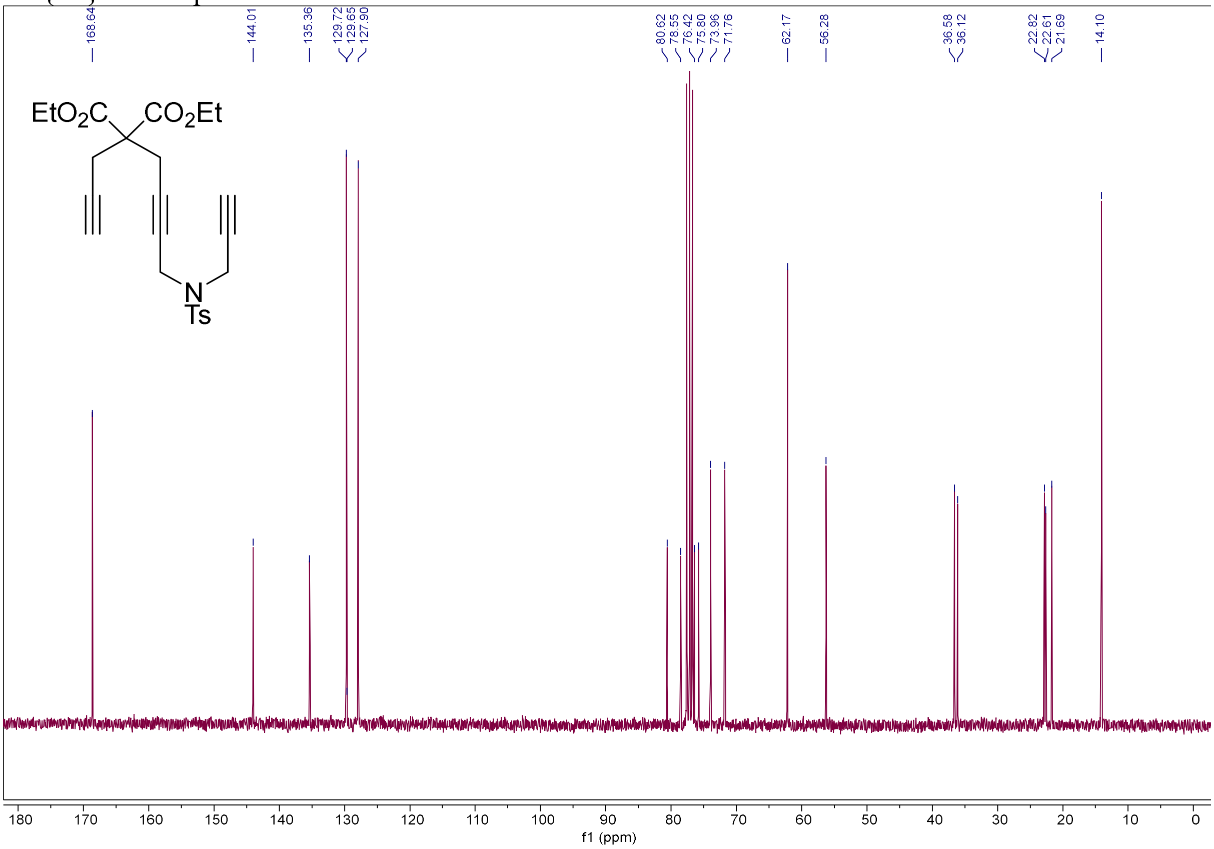

^{1}H NMR spectrum of compound **16**

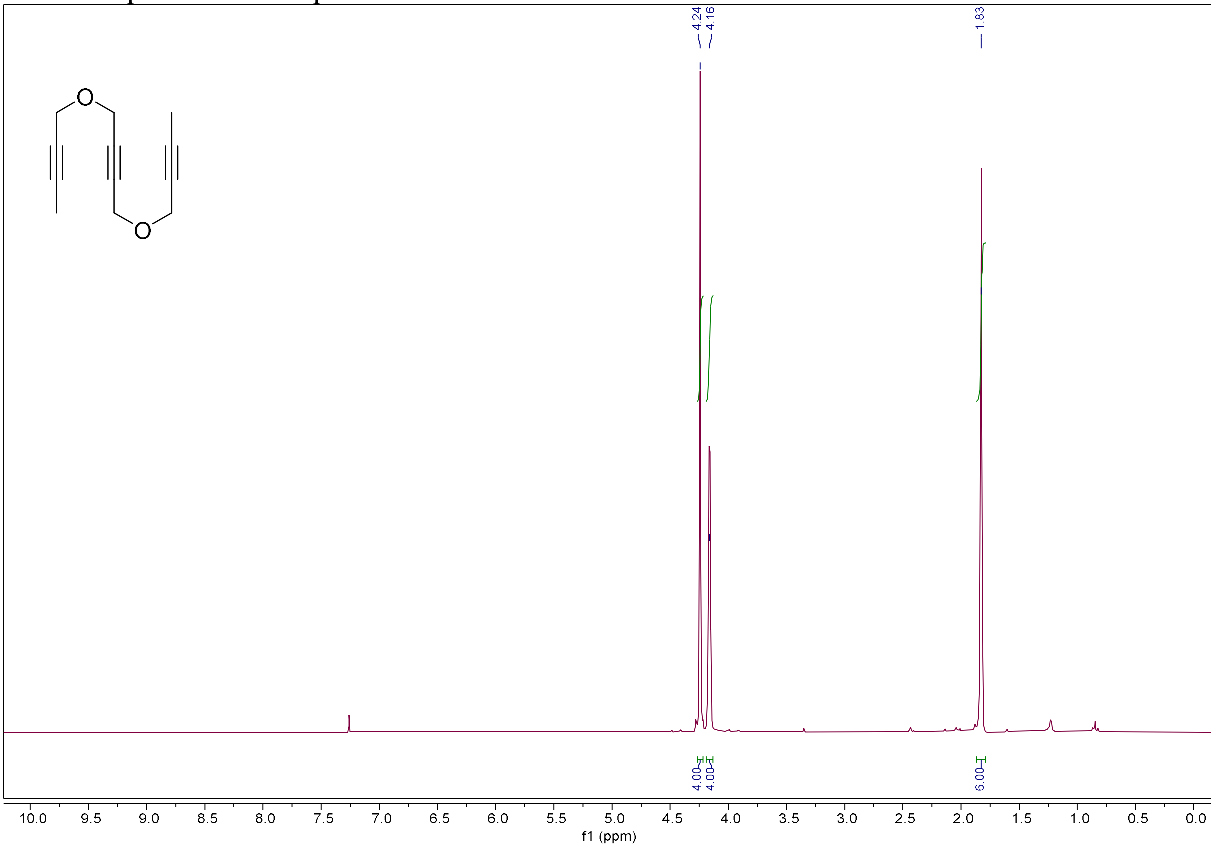

^{1}H NMR spectrum of compound **17**

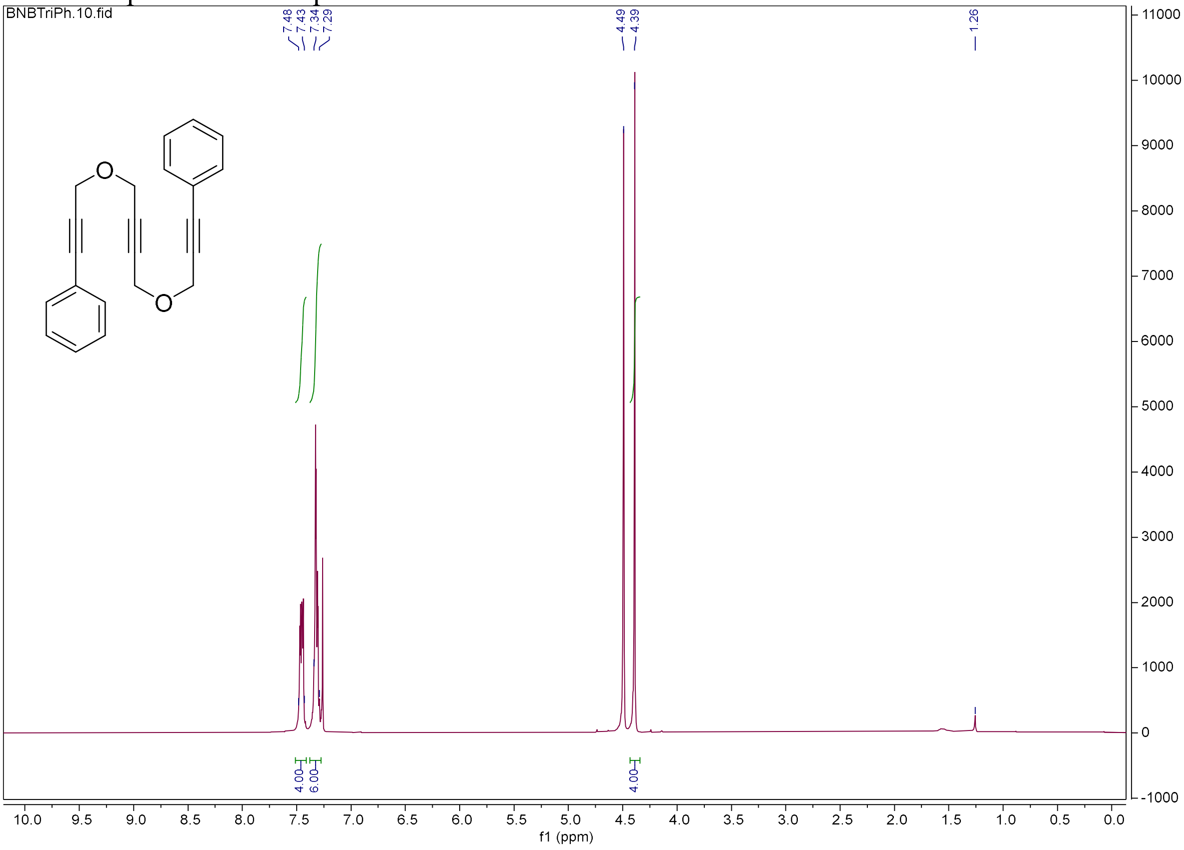

^{1}H NMR spectrum of compound **18**

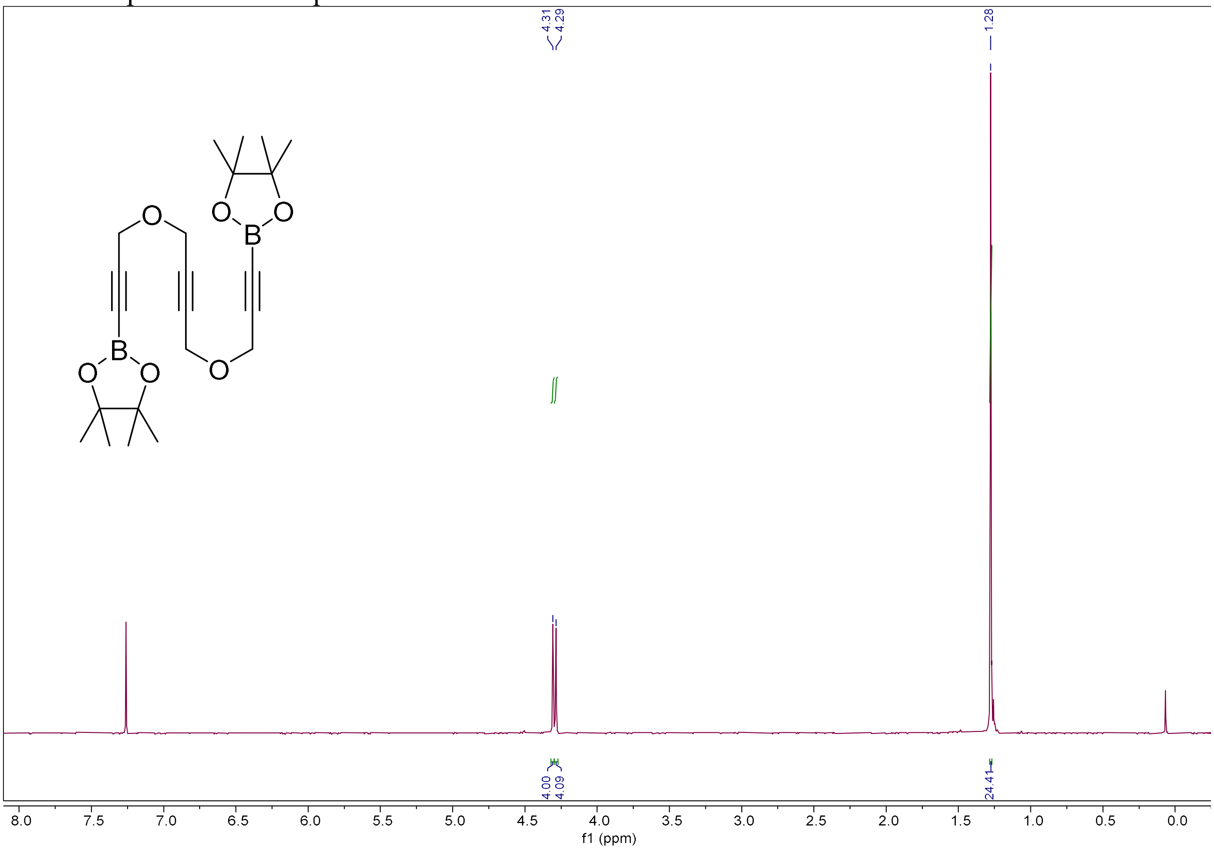

^{1}H NMR spectrum of compound **19**

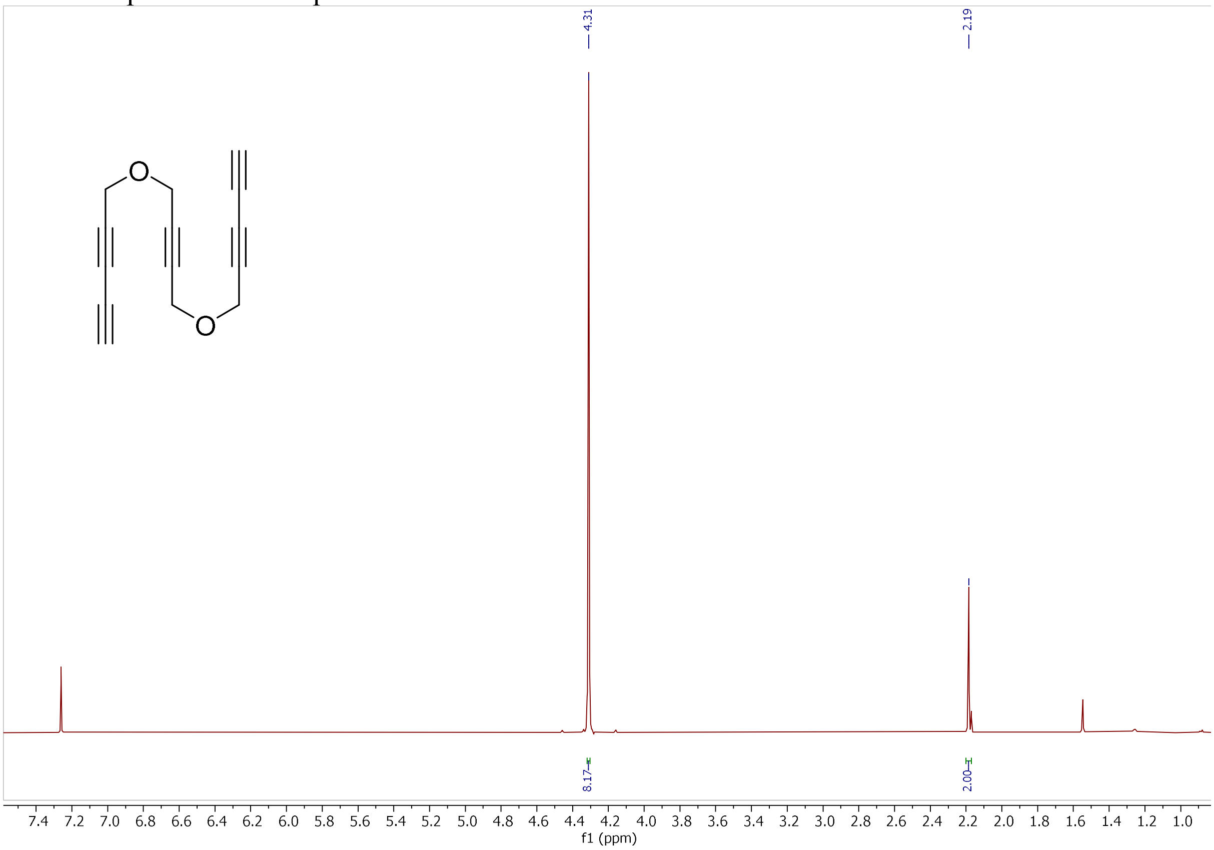

$^{13}C\{^1H\}$ NMR spectrum of **19**

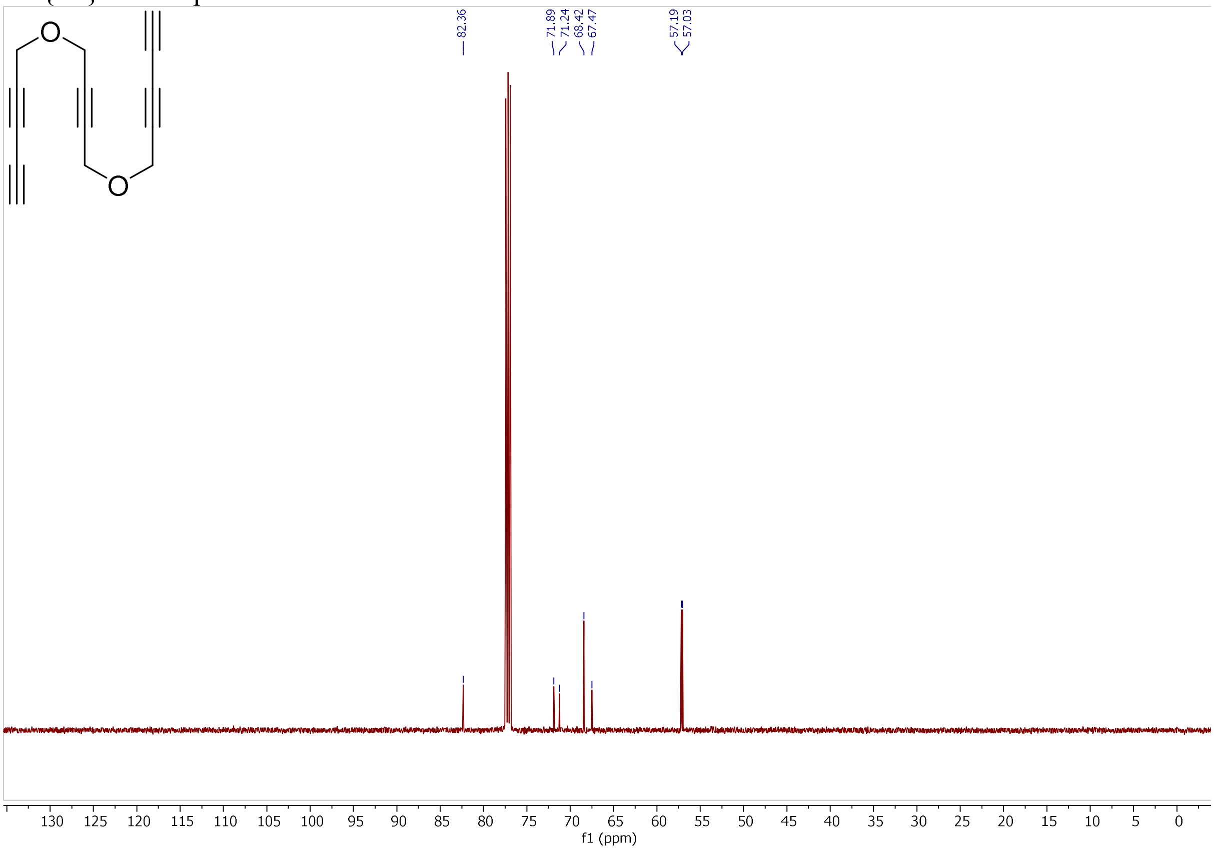

^1H NMR spectrum of compound **20**

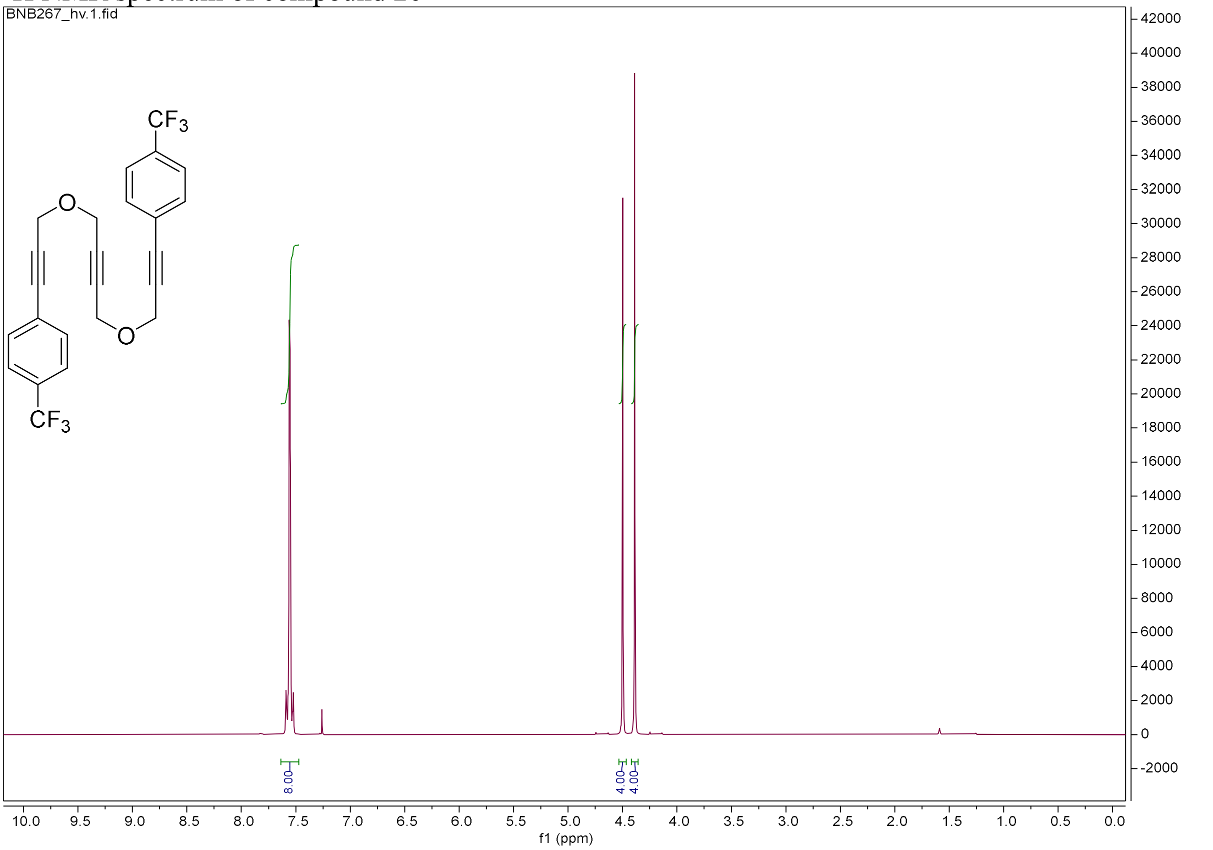

^{1}H NMR spectrum of compound **21**

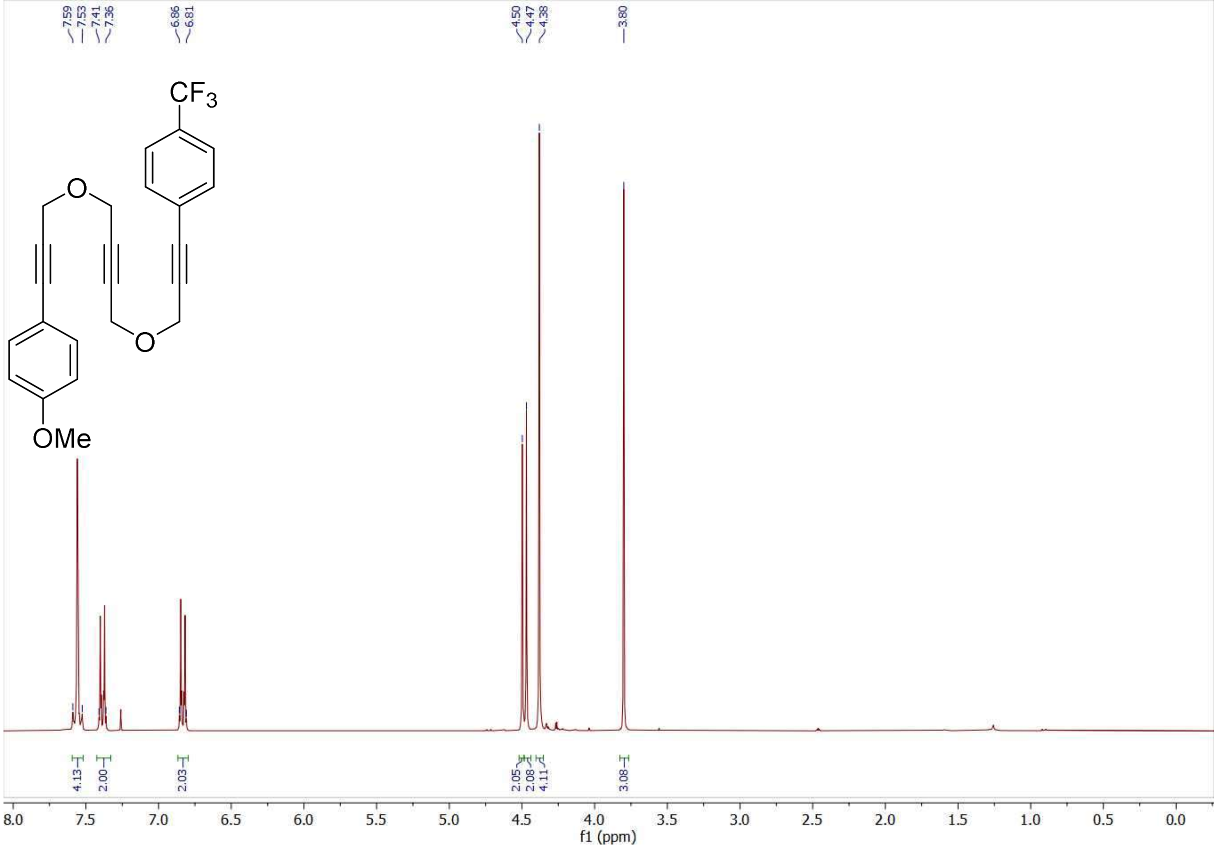

^{1}H NMR spectrum of compound **23**

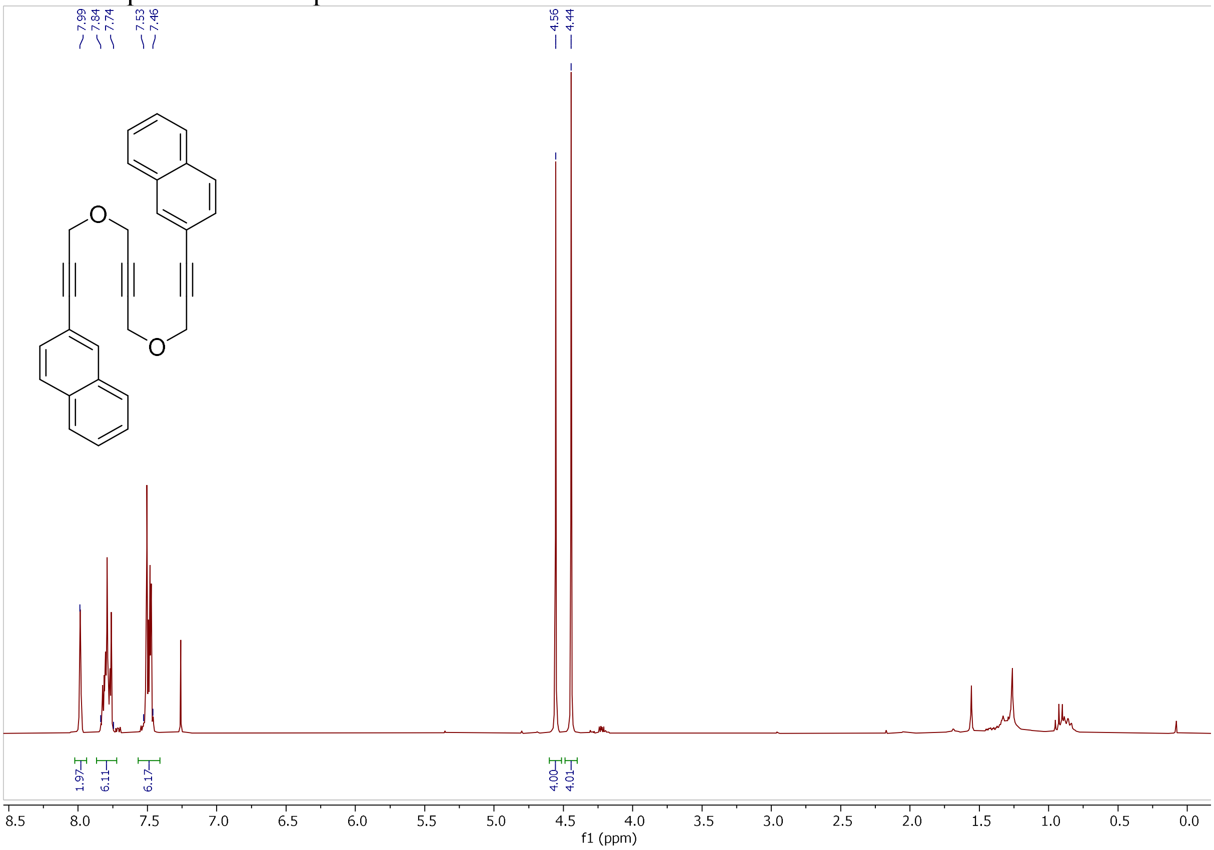

$^{13}C\{^{1}H\}$ NMR spectrum of **23**

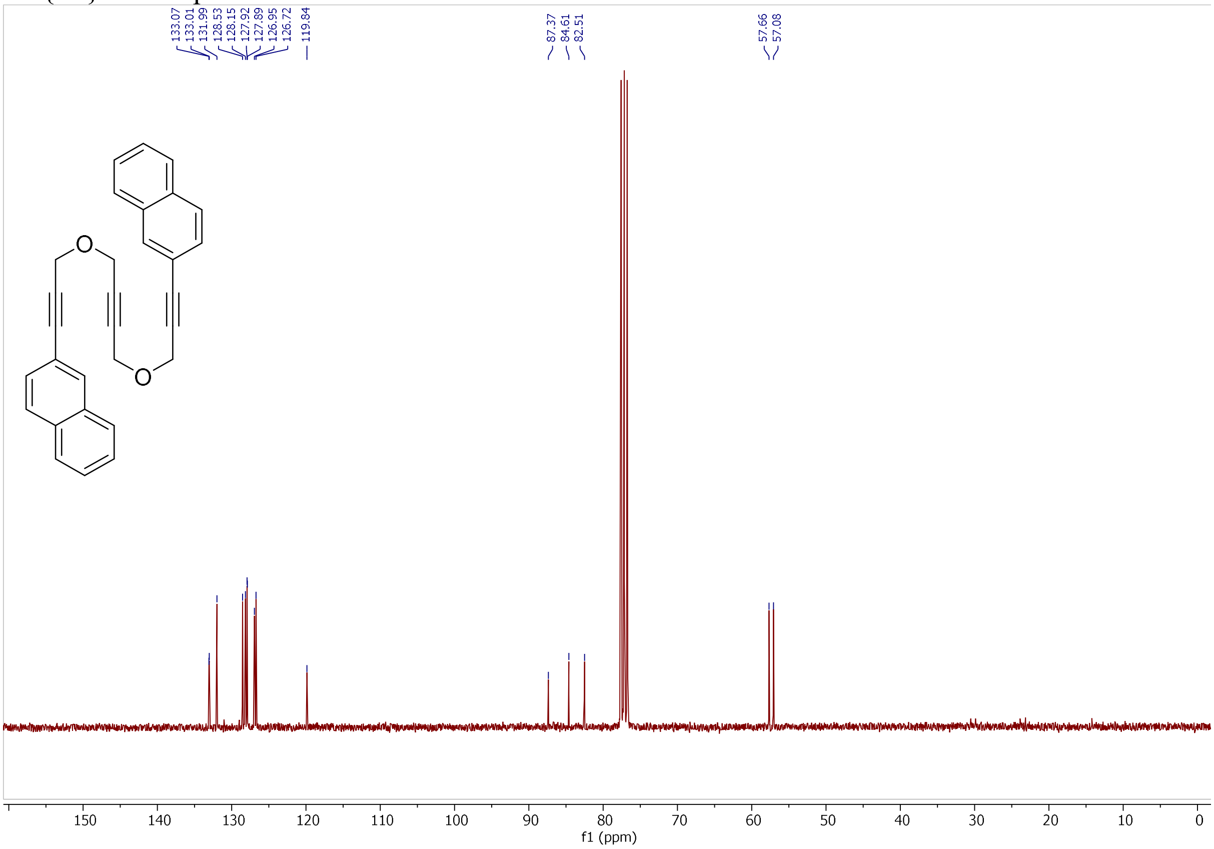

^{1}H NMR spectrum of compound **24**

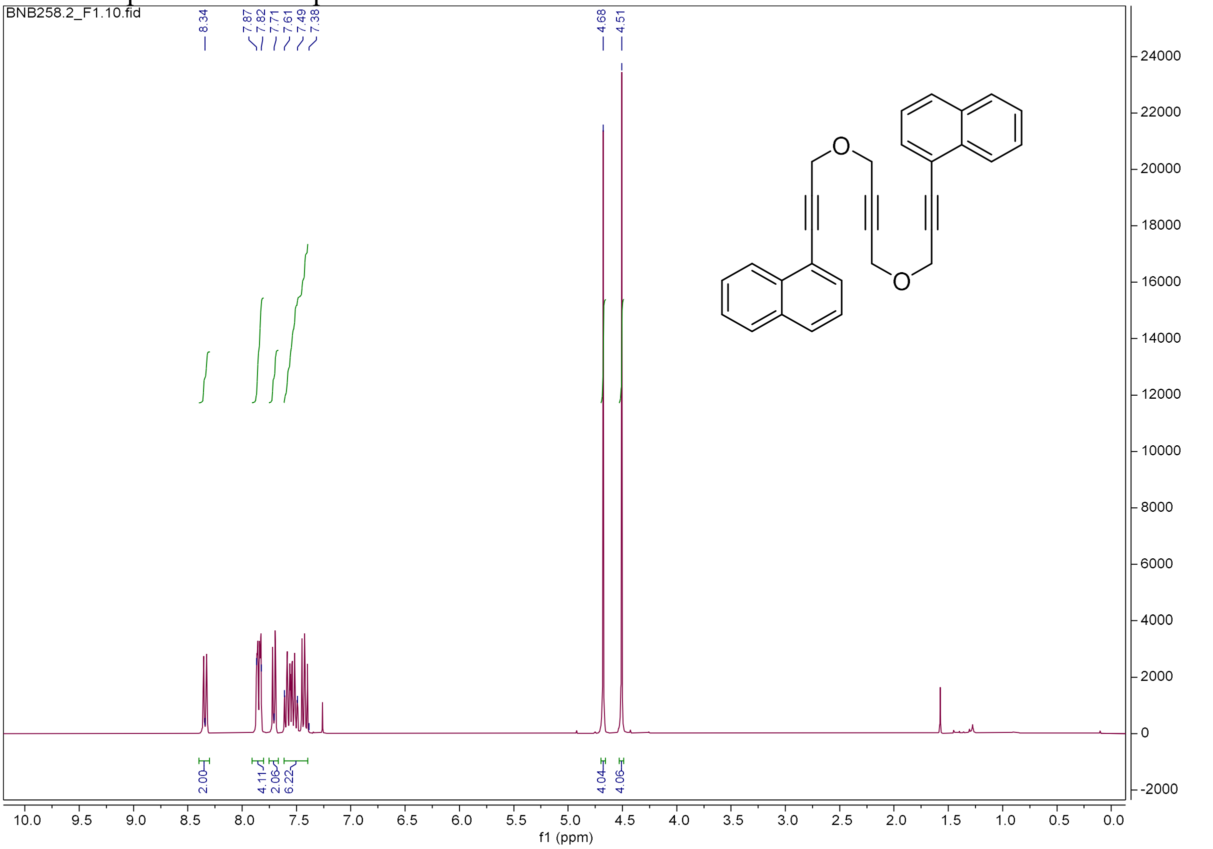

^{1}H NMR spectrum of compound **25**

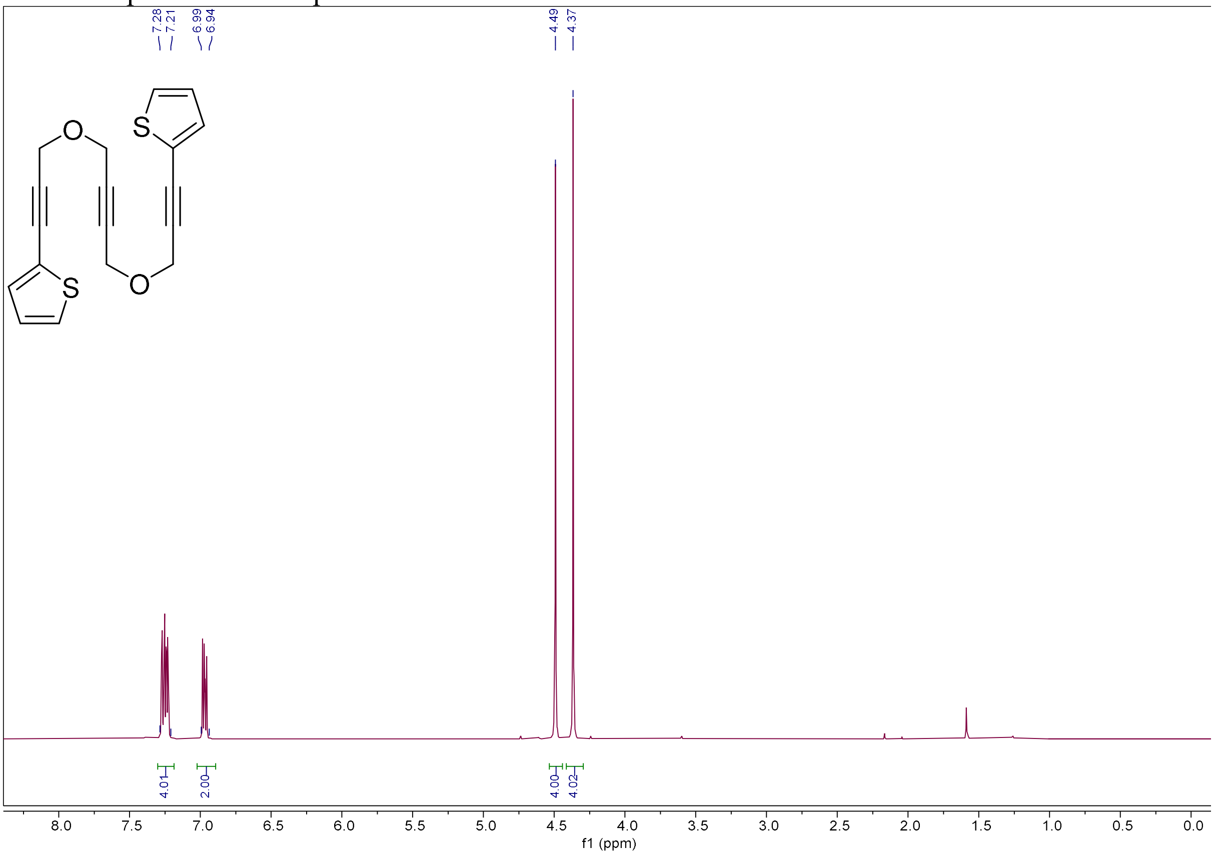

$^{13}C\{^{1}H\}$ NMR spectrum of **25**

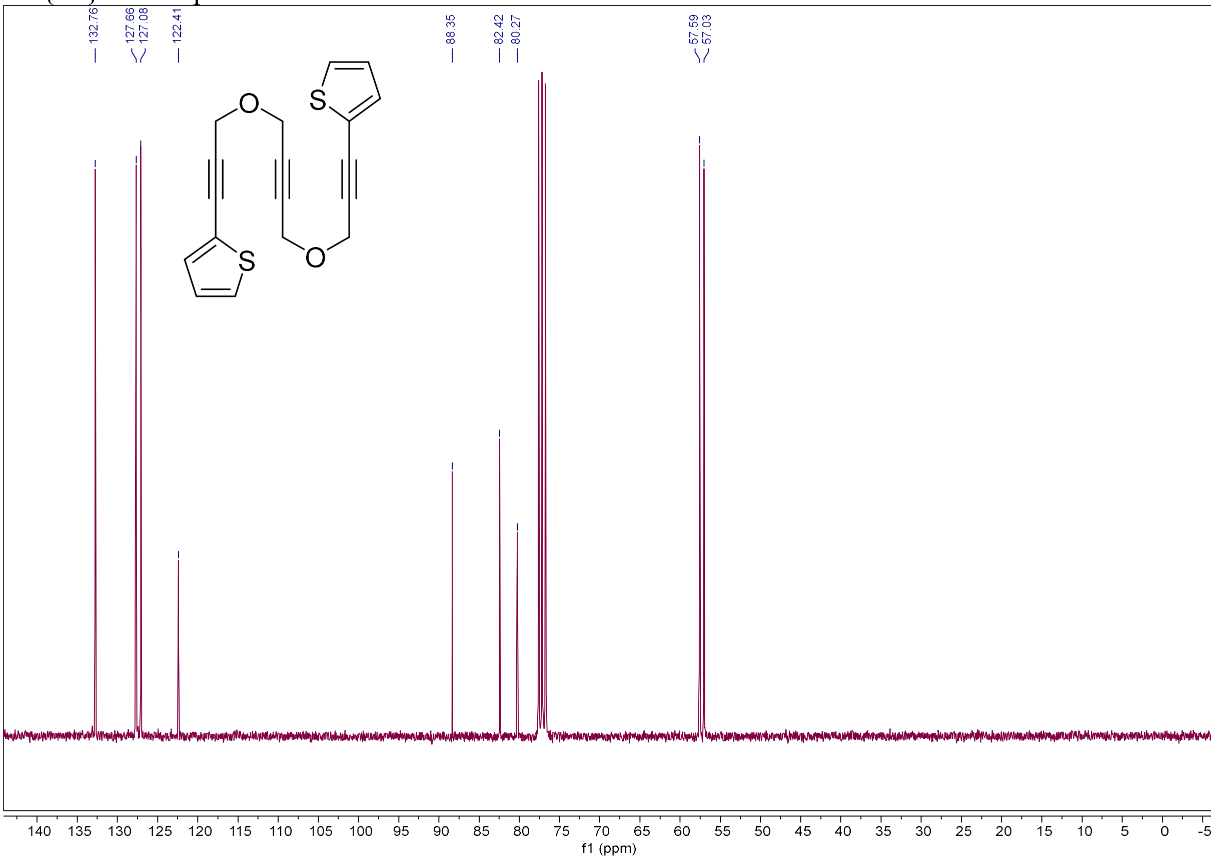

^{1}H NMR spectrum of compound **26**

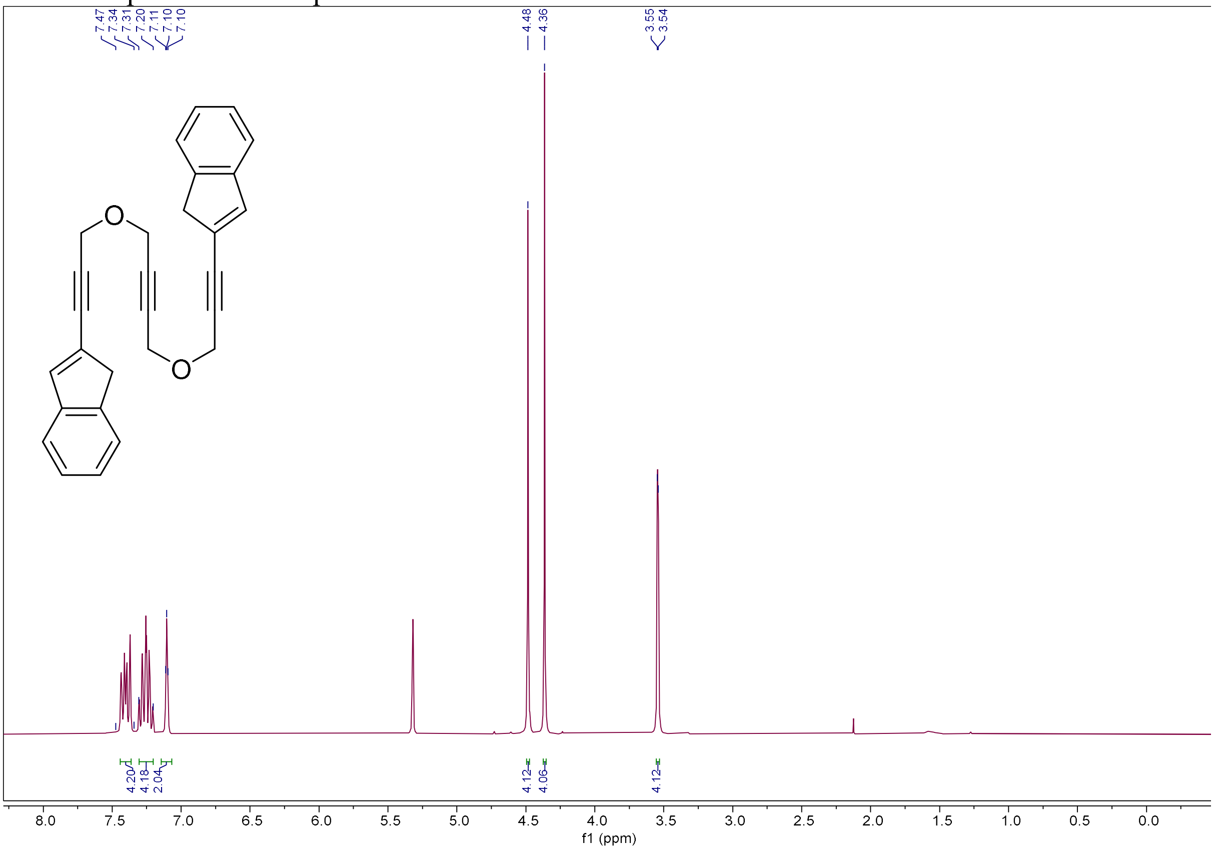

^{13}C{^{1}H} NMR spectrum of **26**

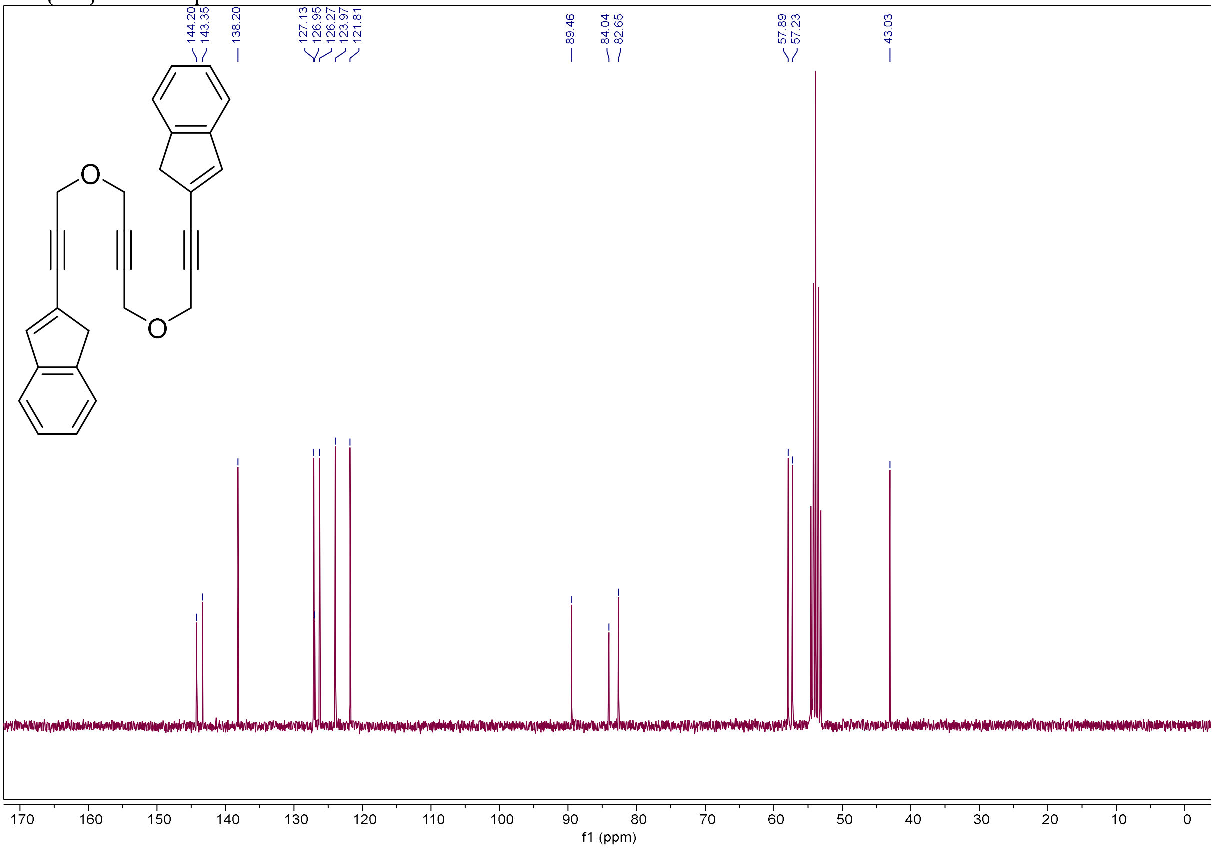

^{1}H NMR spectrum of compound **27**

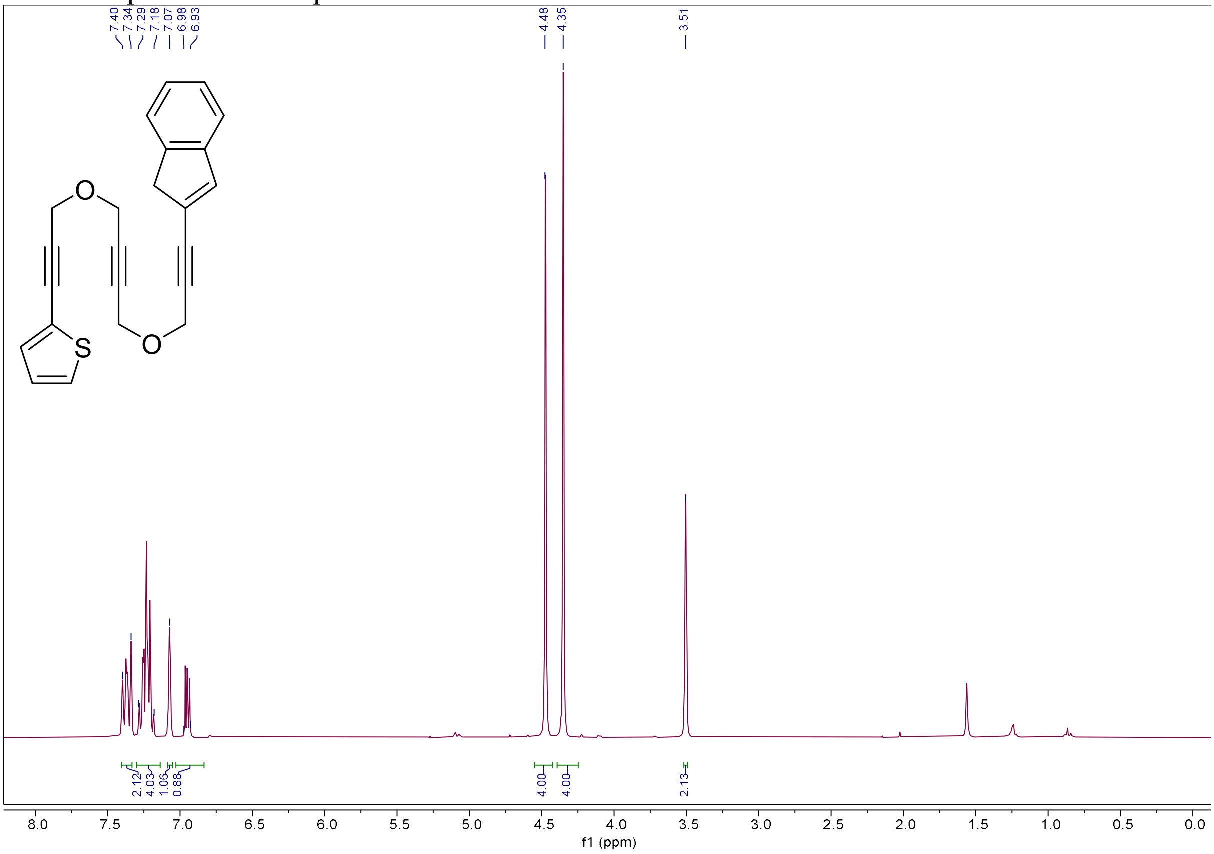

^{13}C{^{1}H} NMR spectrum of **27**

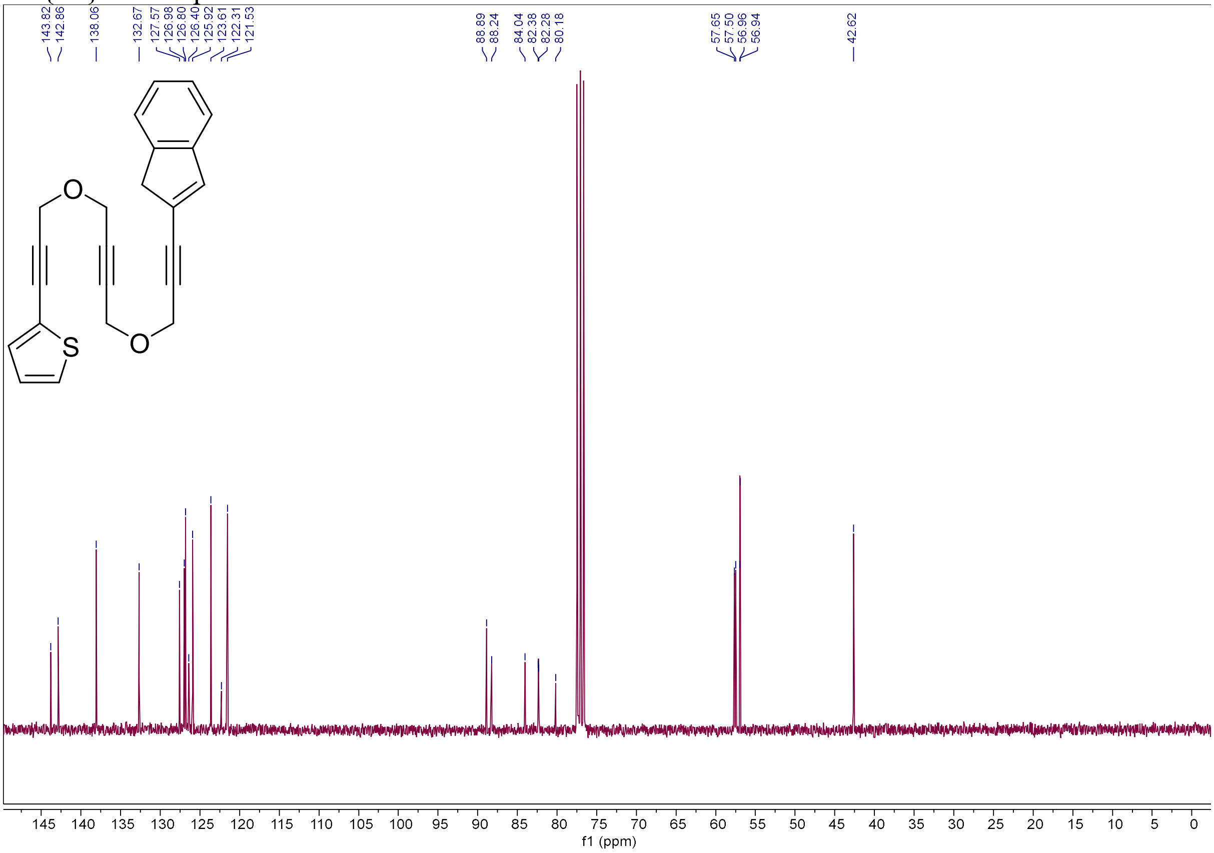

^{1}H NMR spectrum of compound **28**

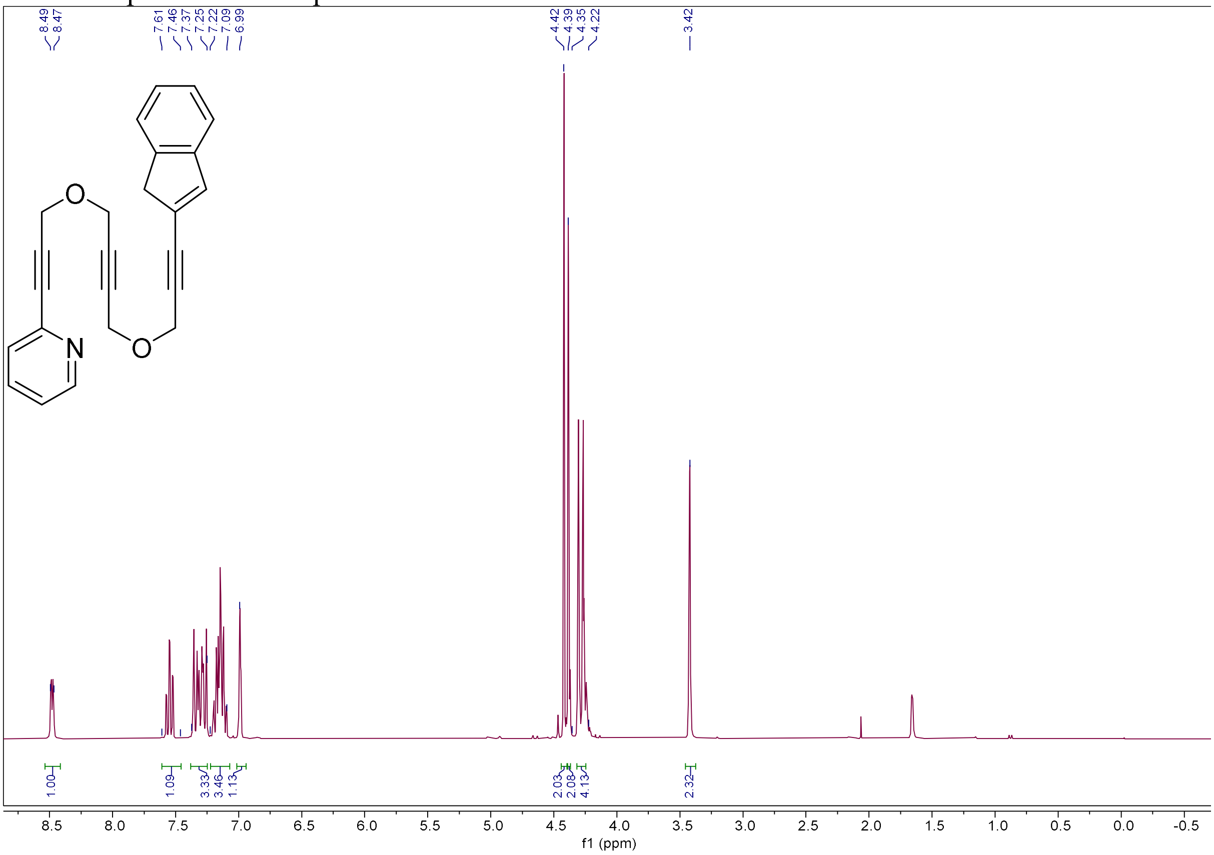

^{13}C{^{1}H} NMR spectrum of **28**

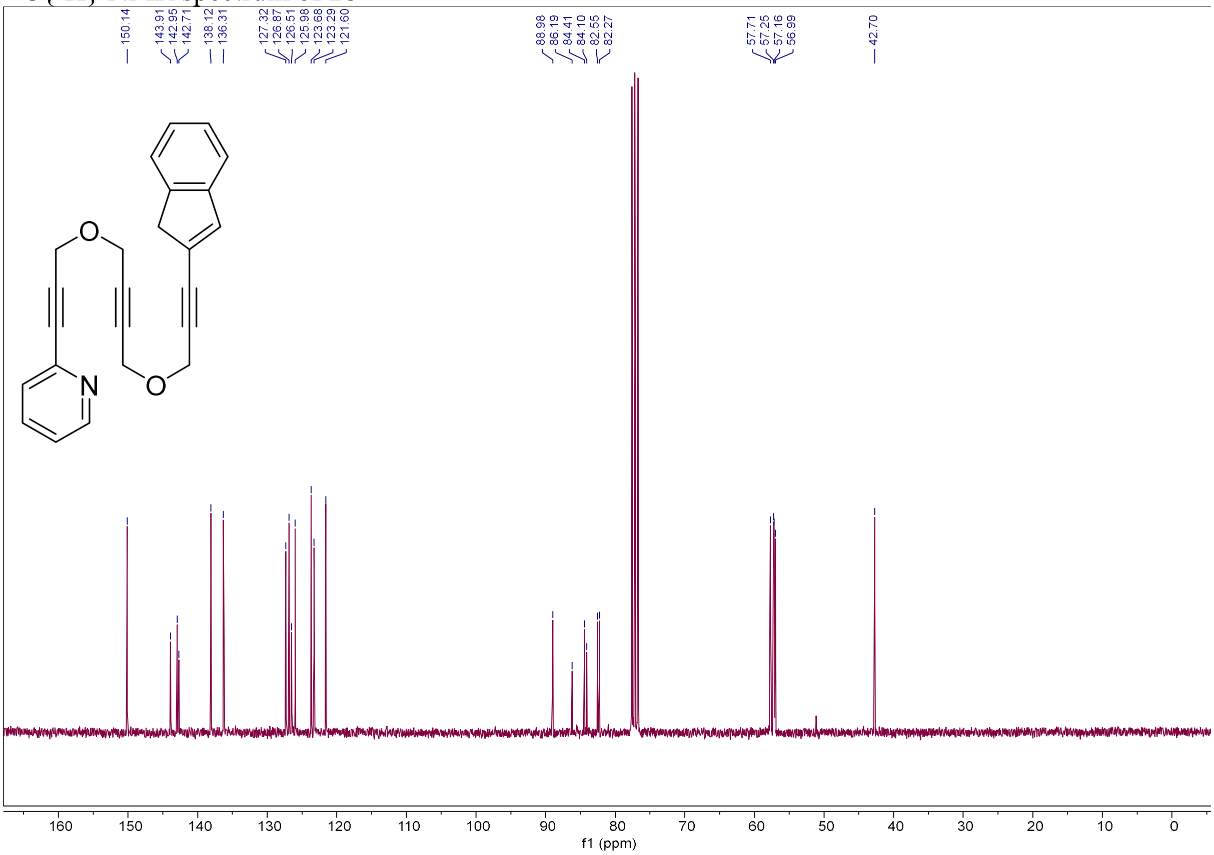

^{1}H NMR spectrum of compound **29**

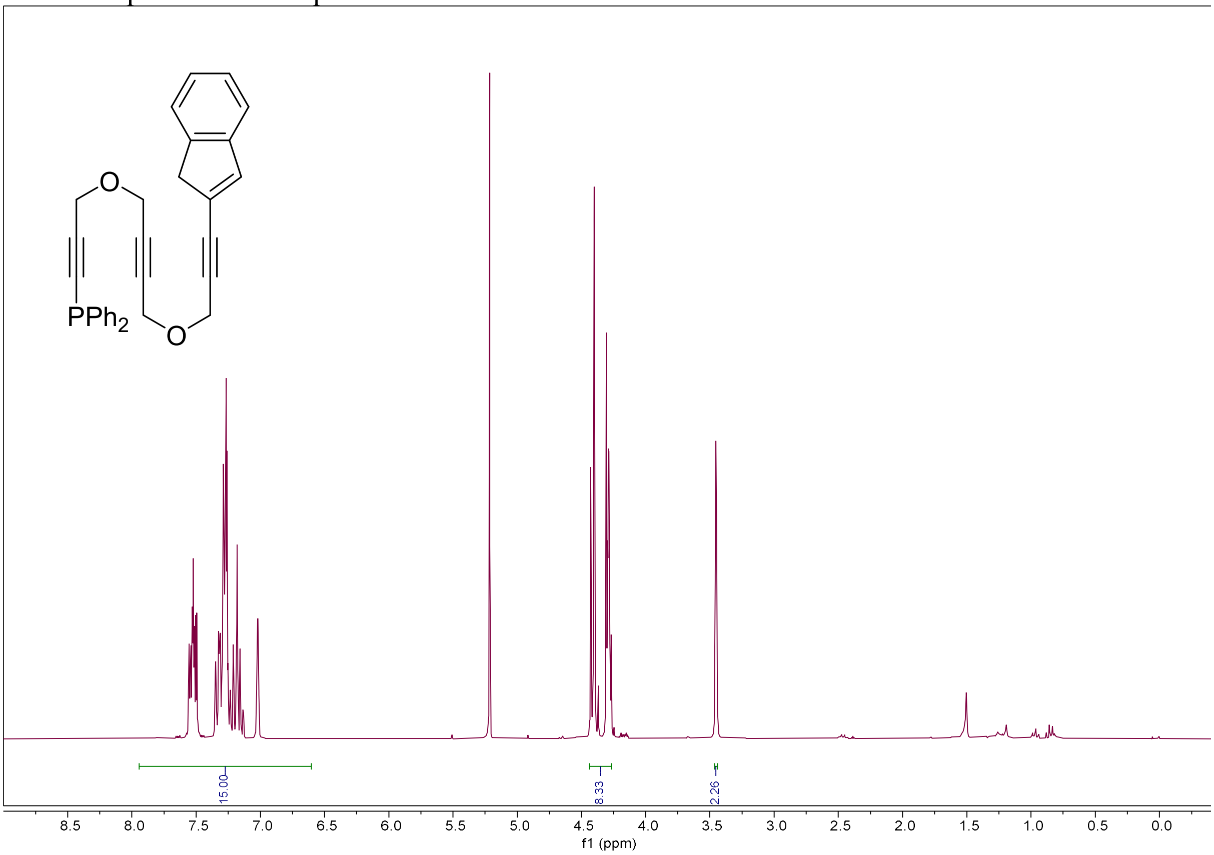

^{13}C{^{1}H} NMR spectrum of **29**

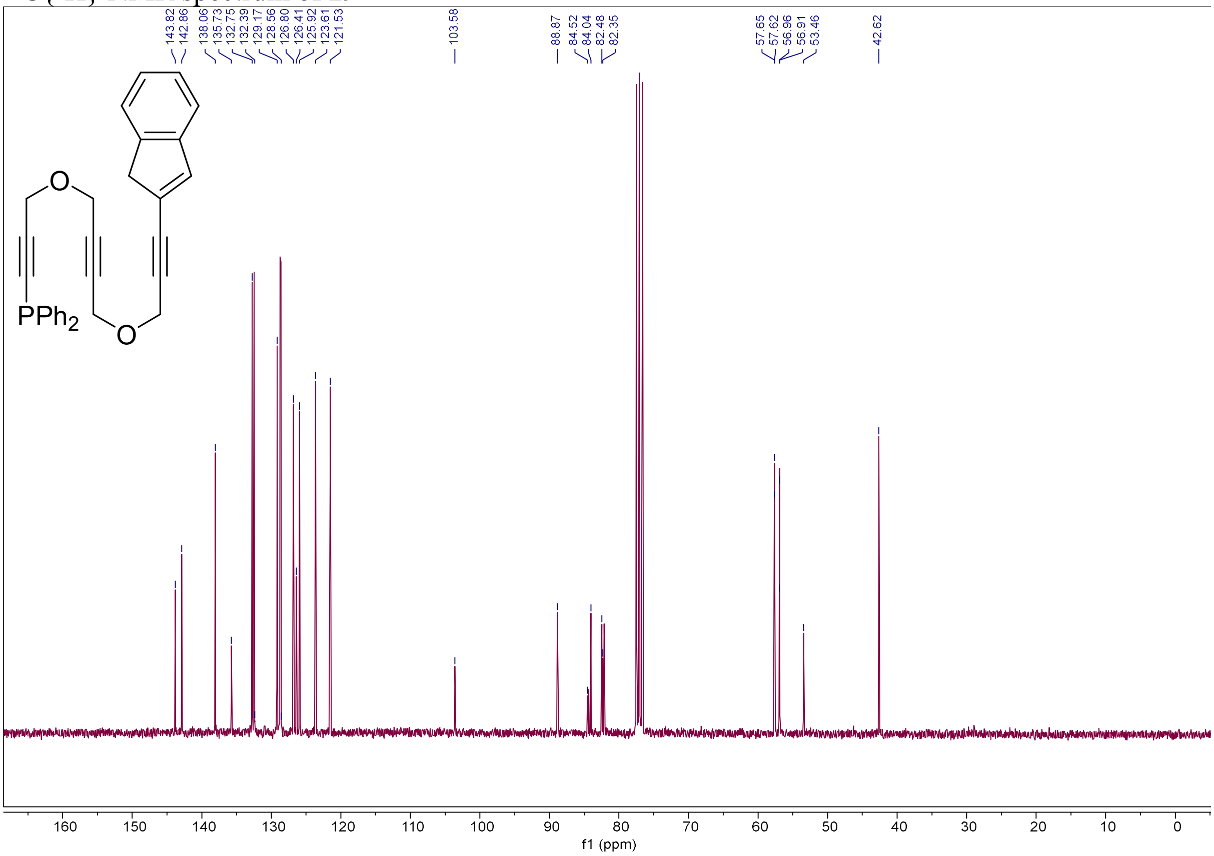

$^{31}P\{^{1}H\}$ NMR spectrum of compound **29**

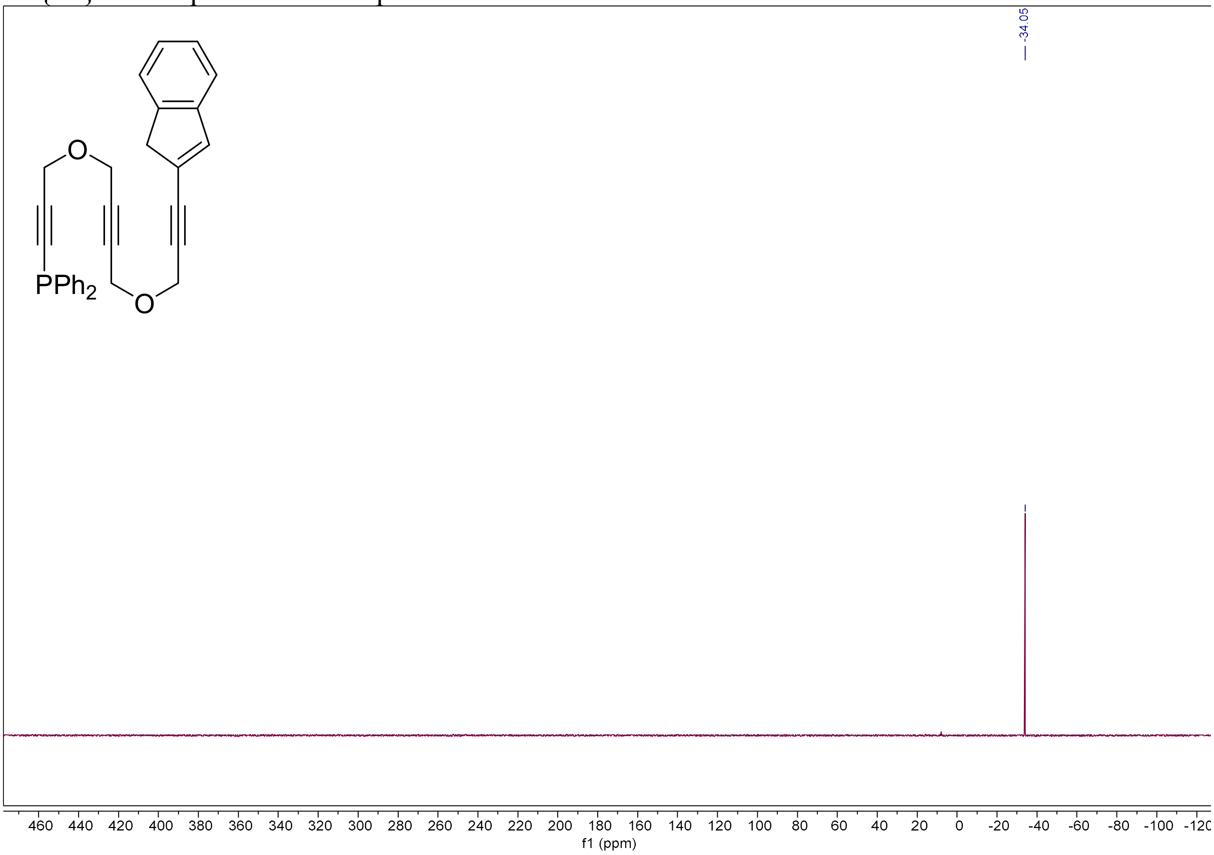

^{1}H NMR spectrum of compound **30**

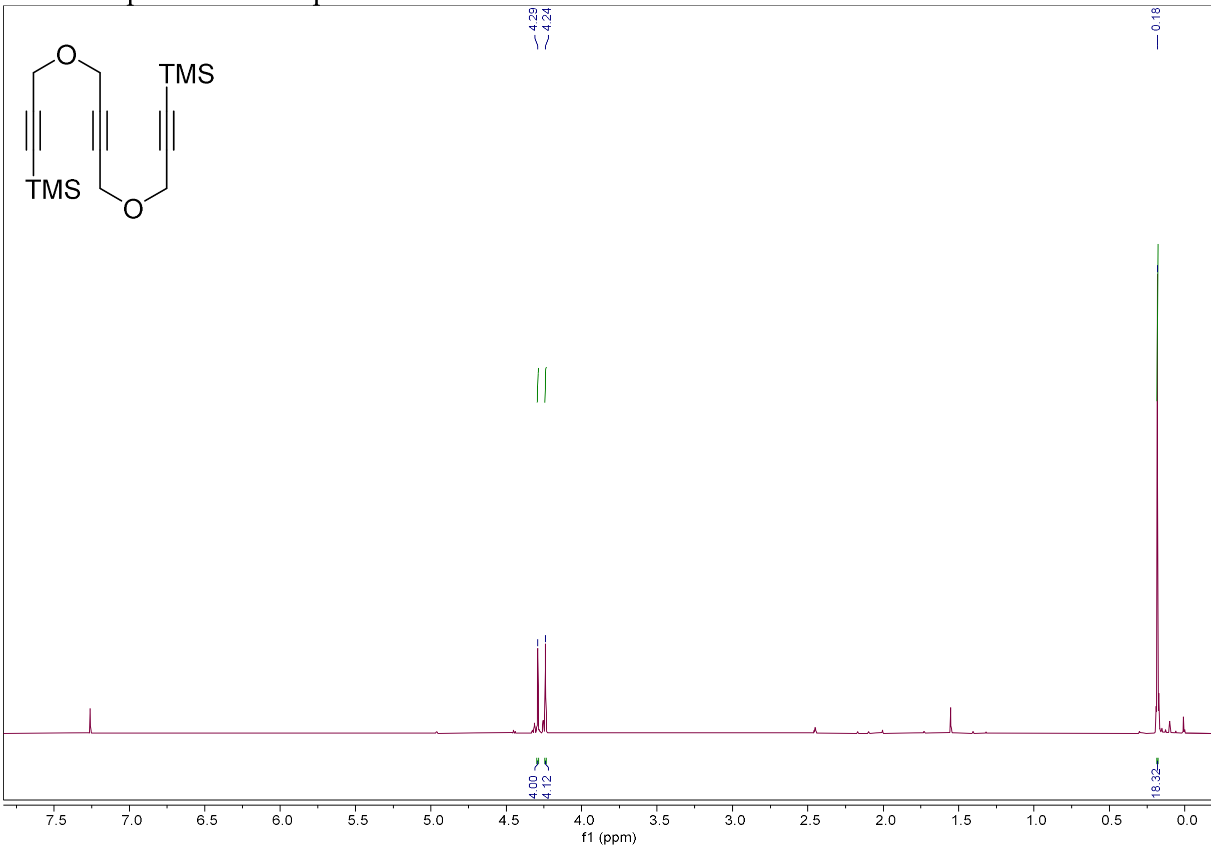

^{1}H NMR spectrum of compound **31**

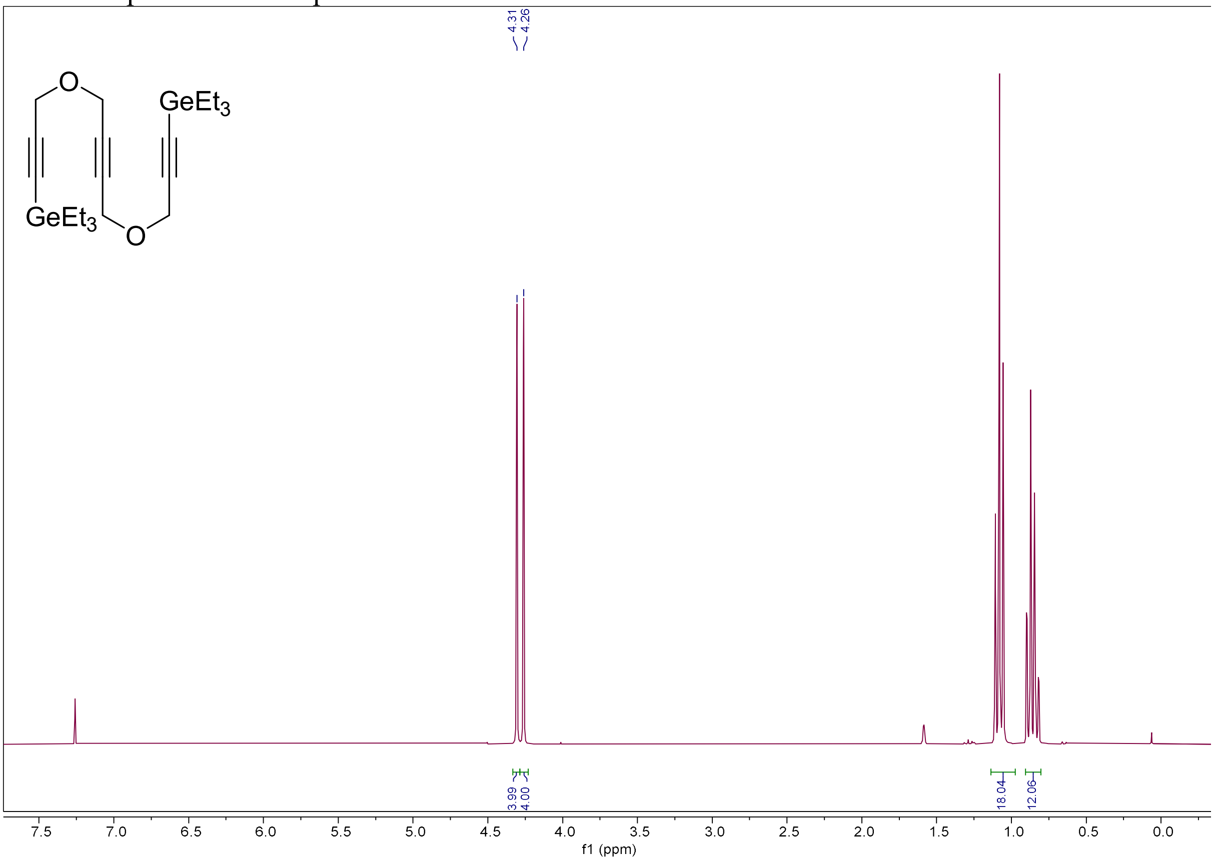

^{13}C{^{1}H} NMR spectrum of **31**

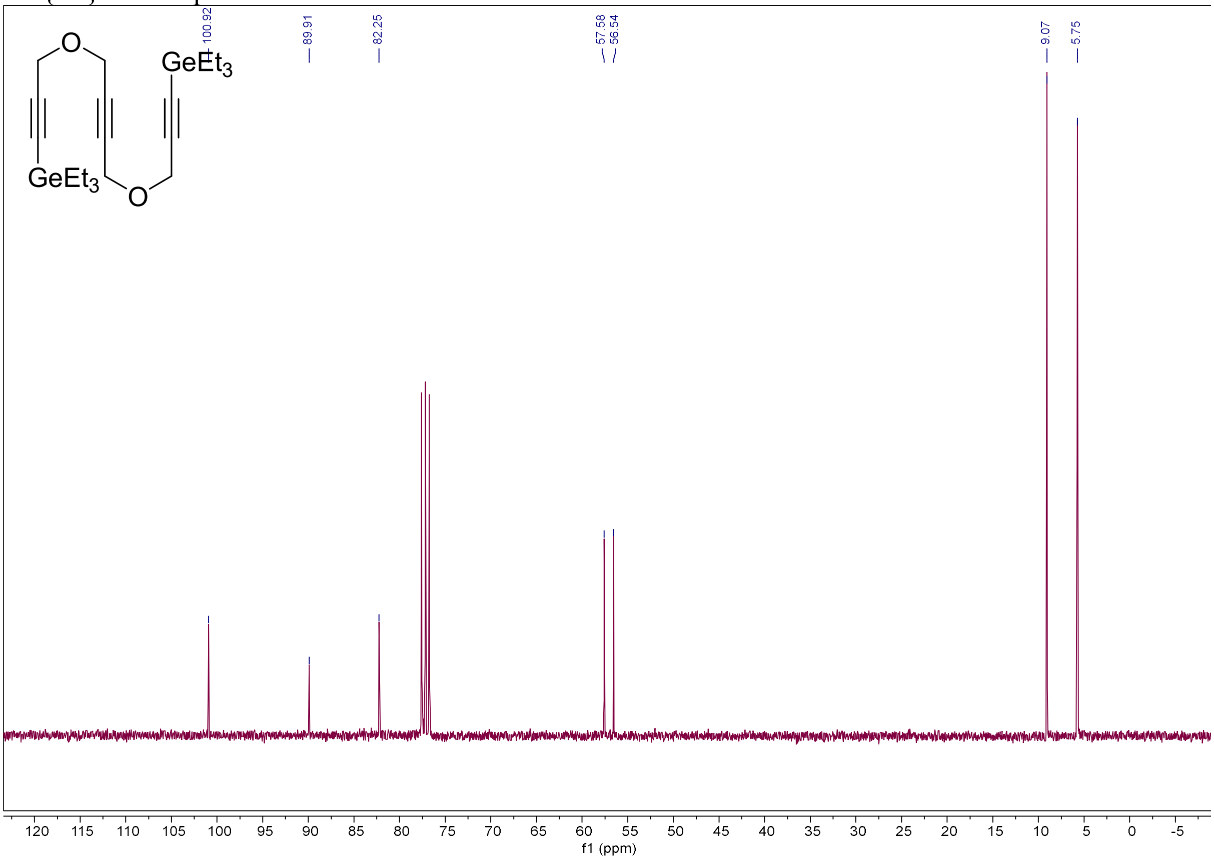

^{1}H NMR spectrum of compound **34**

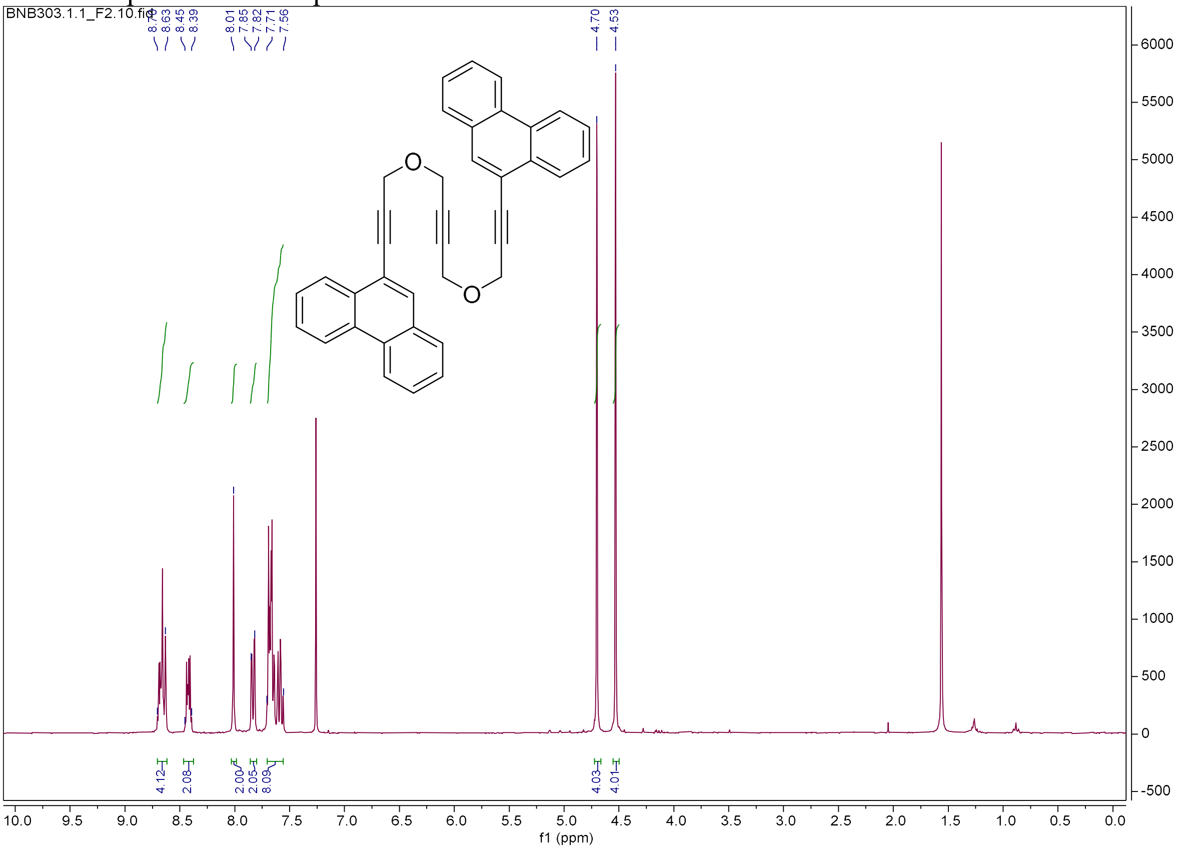

^{1}H NMR spectrum of compound **35**

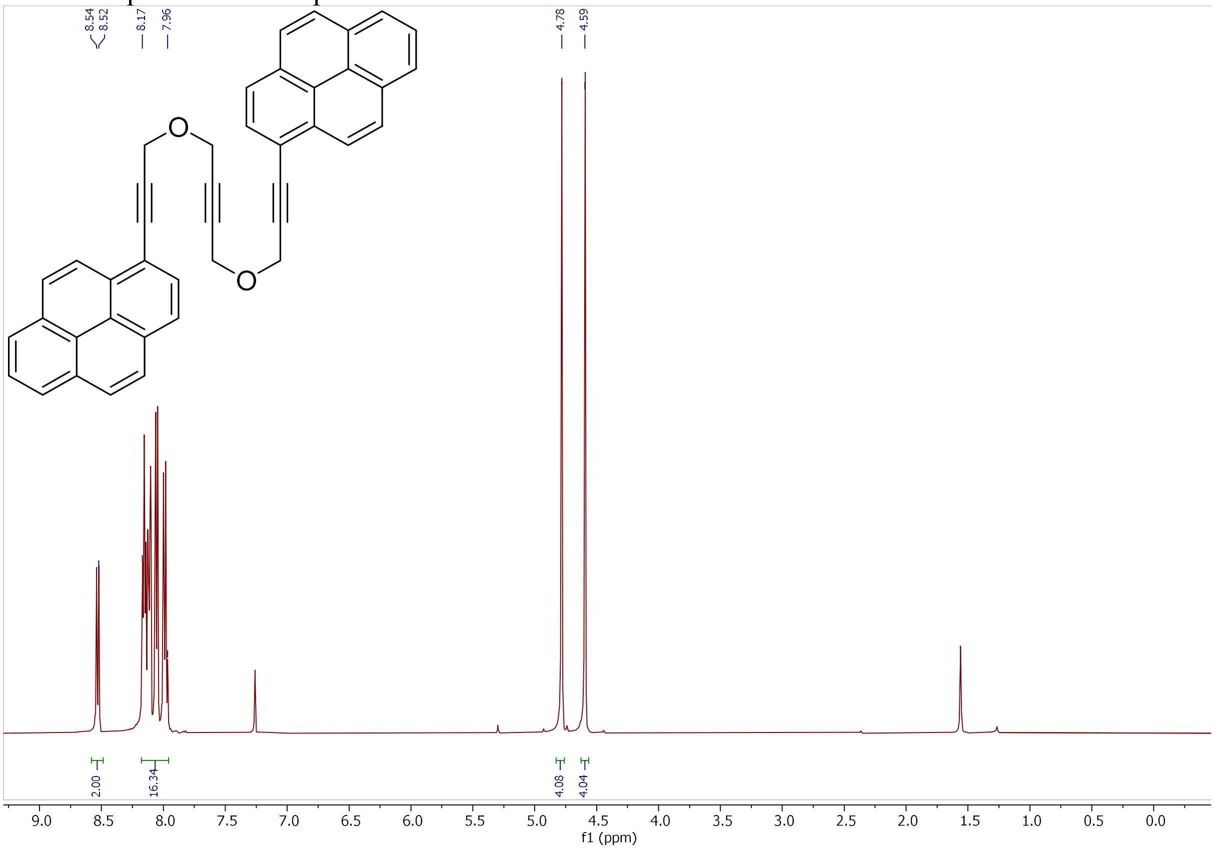

$^{13}C\{^1H\}$ NMR spectrum of **35**

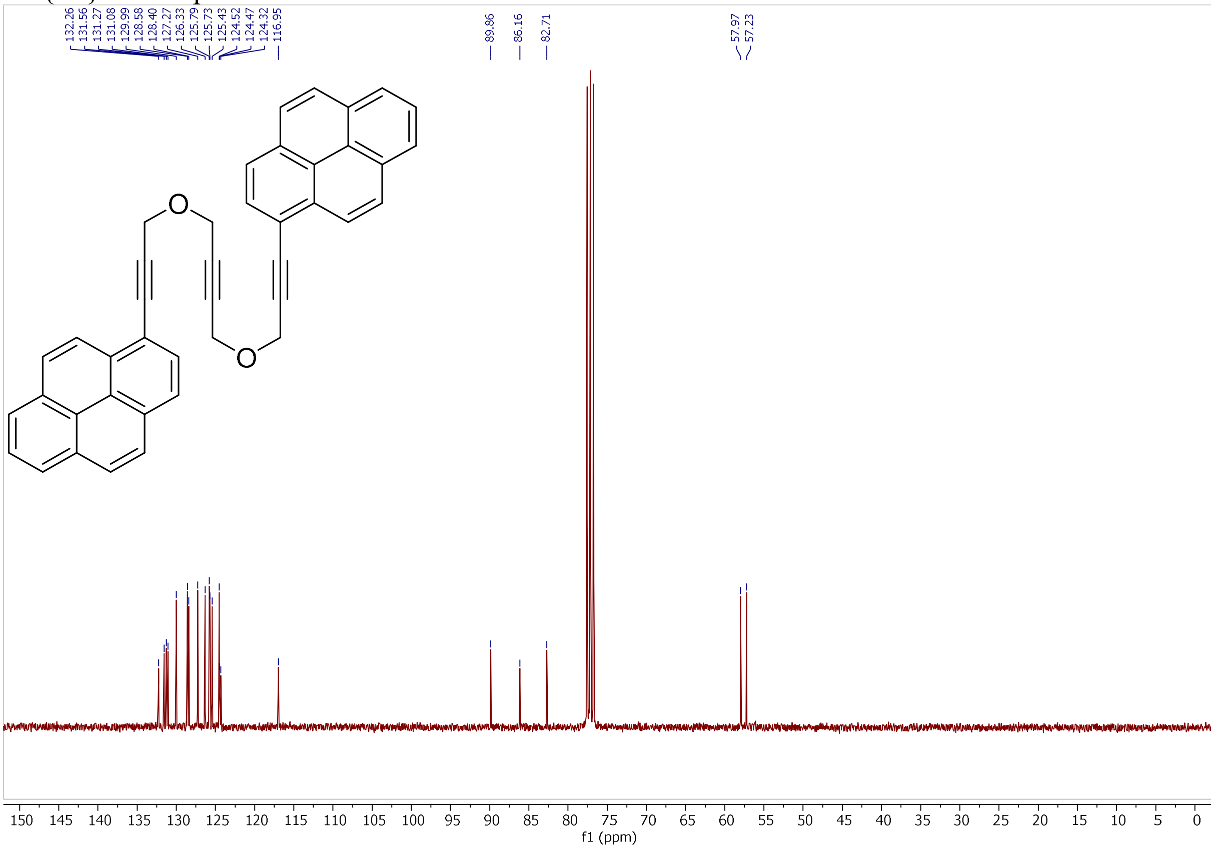

^1H NMR spectrum of compound **36**

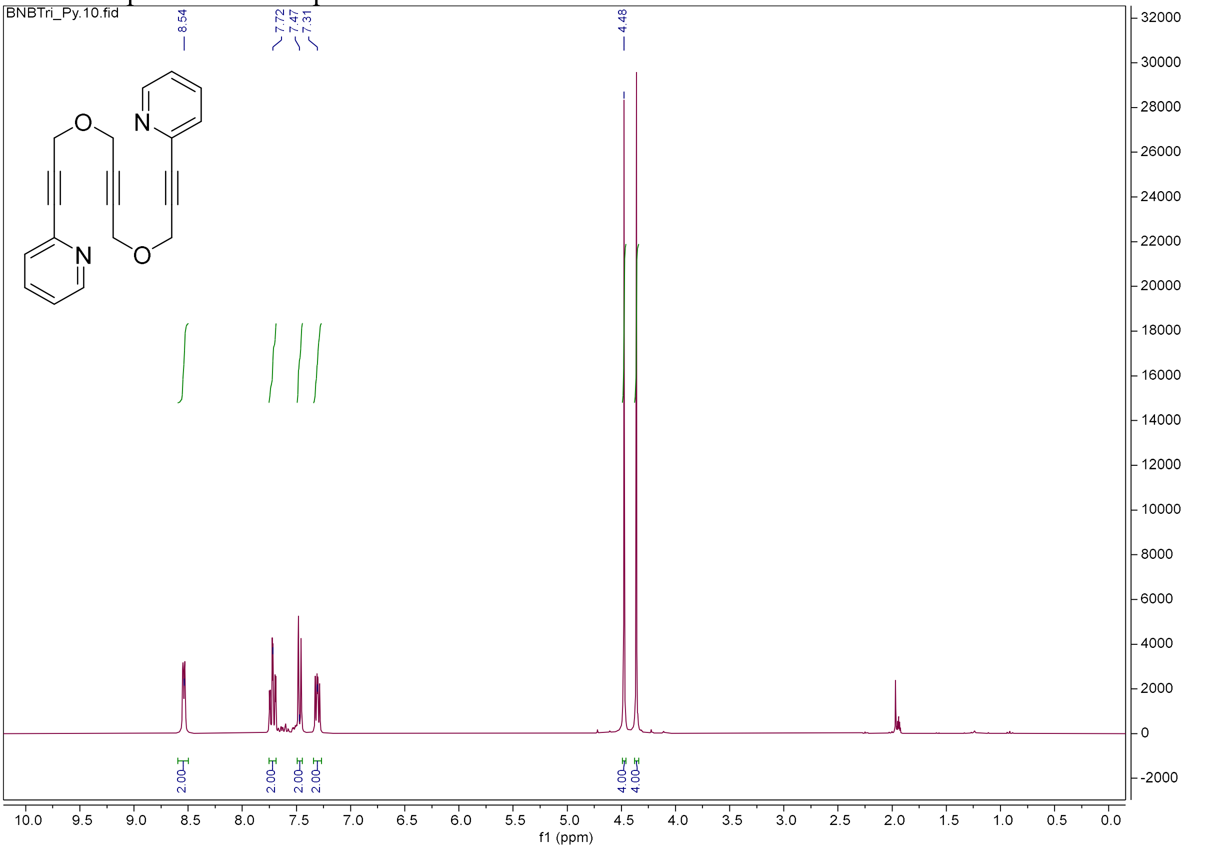

^{1}H NMR spectrum of compound **37**

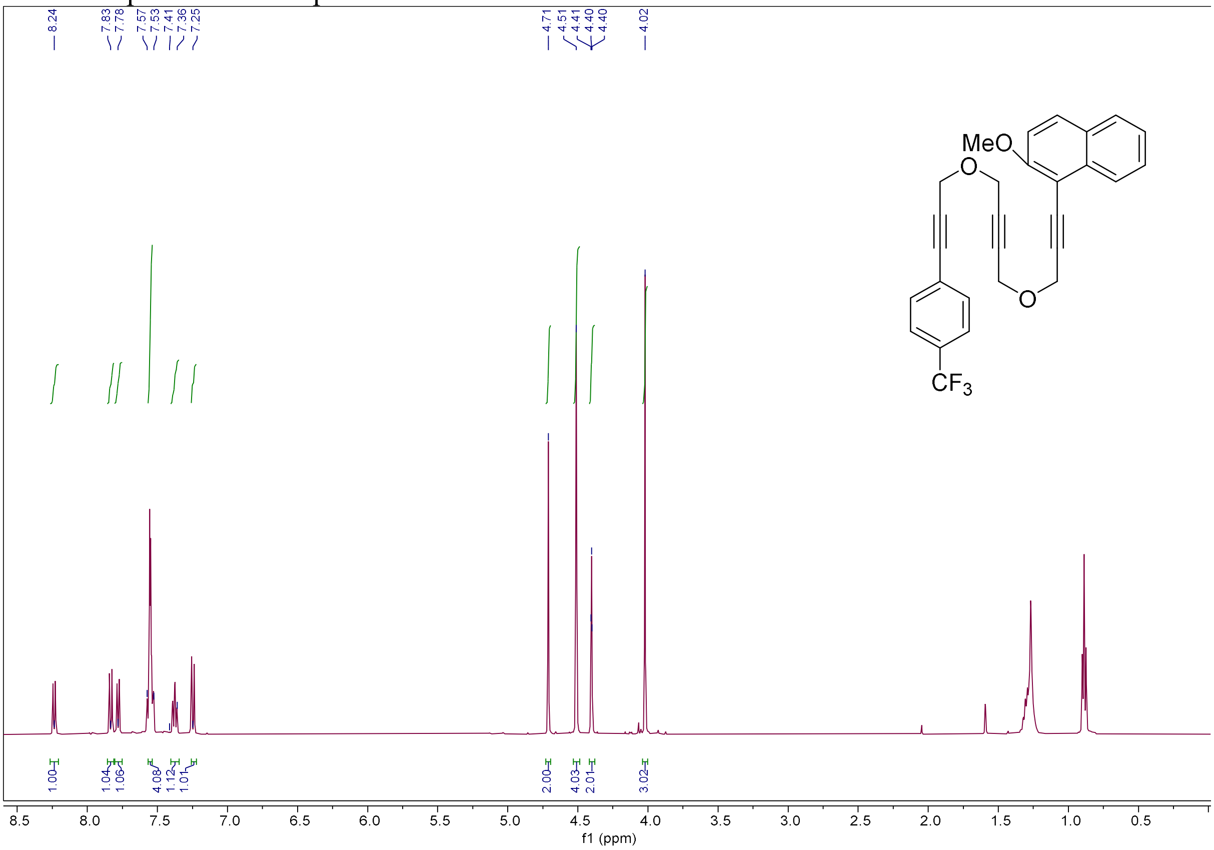

^{1}H NMR spectrum of compound **58**

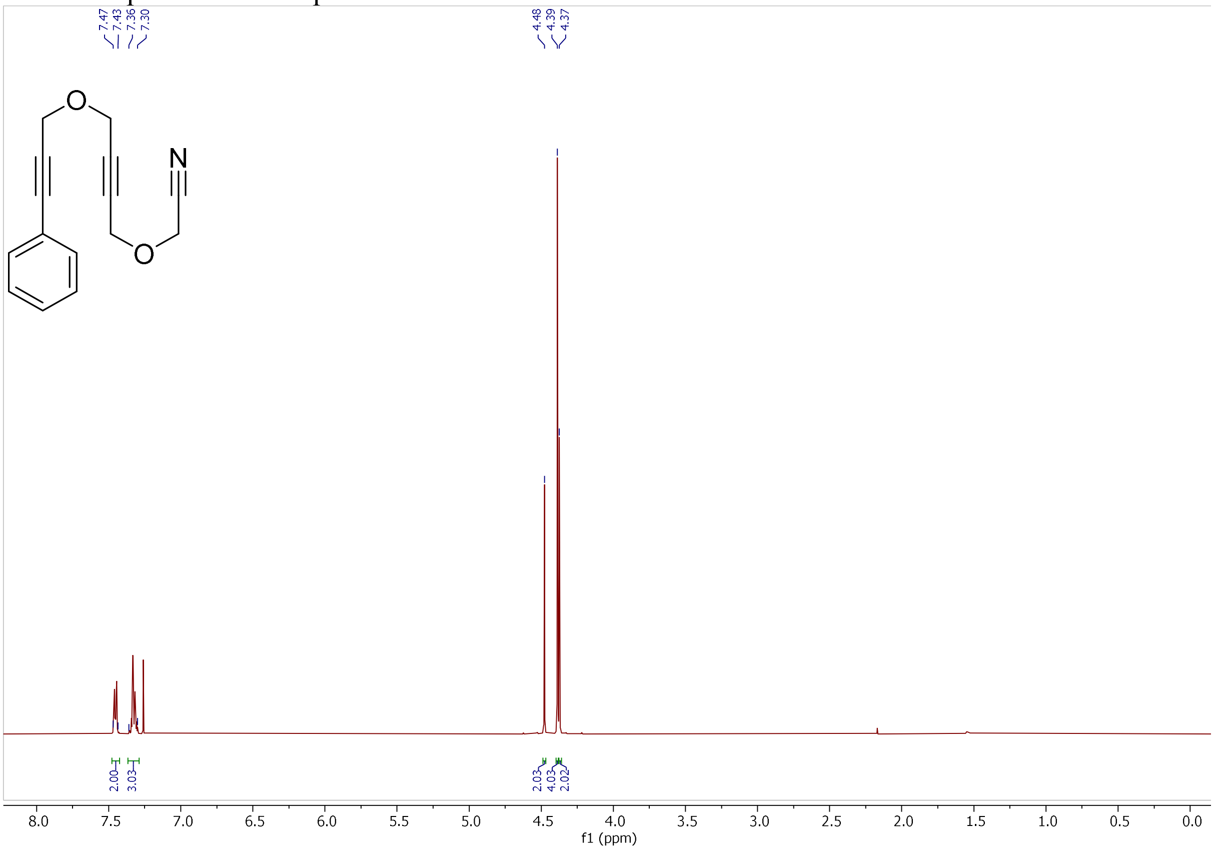

$^{13}C\{^1H\}$ NMR spectrum of **58**

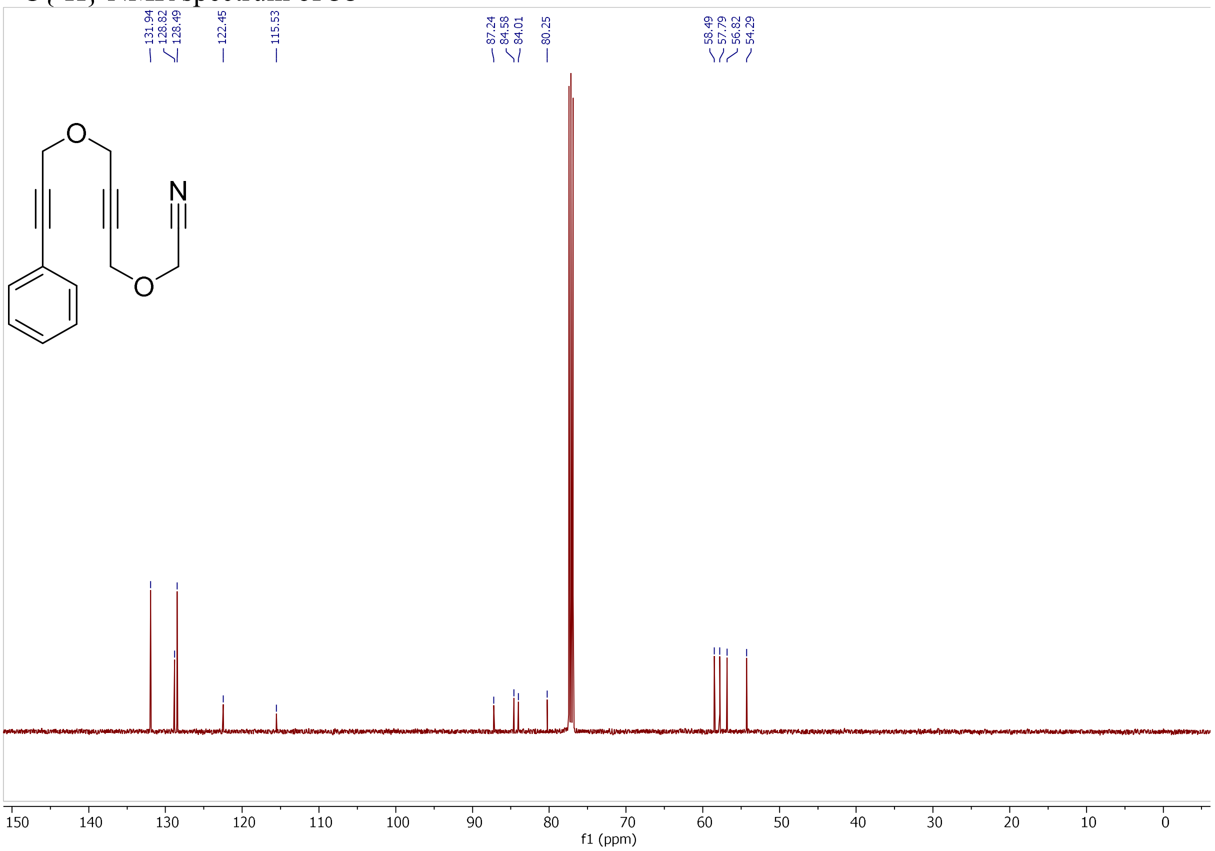

^1H NMR spectrum of compound **60**

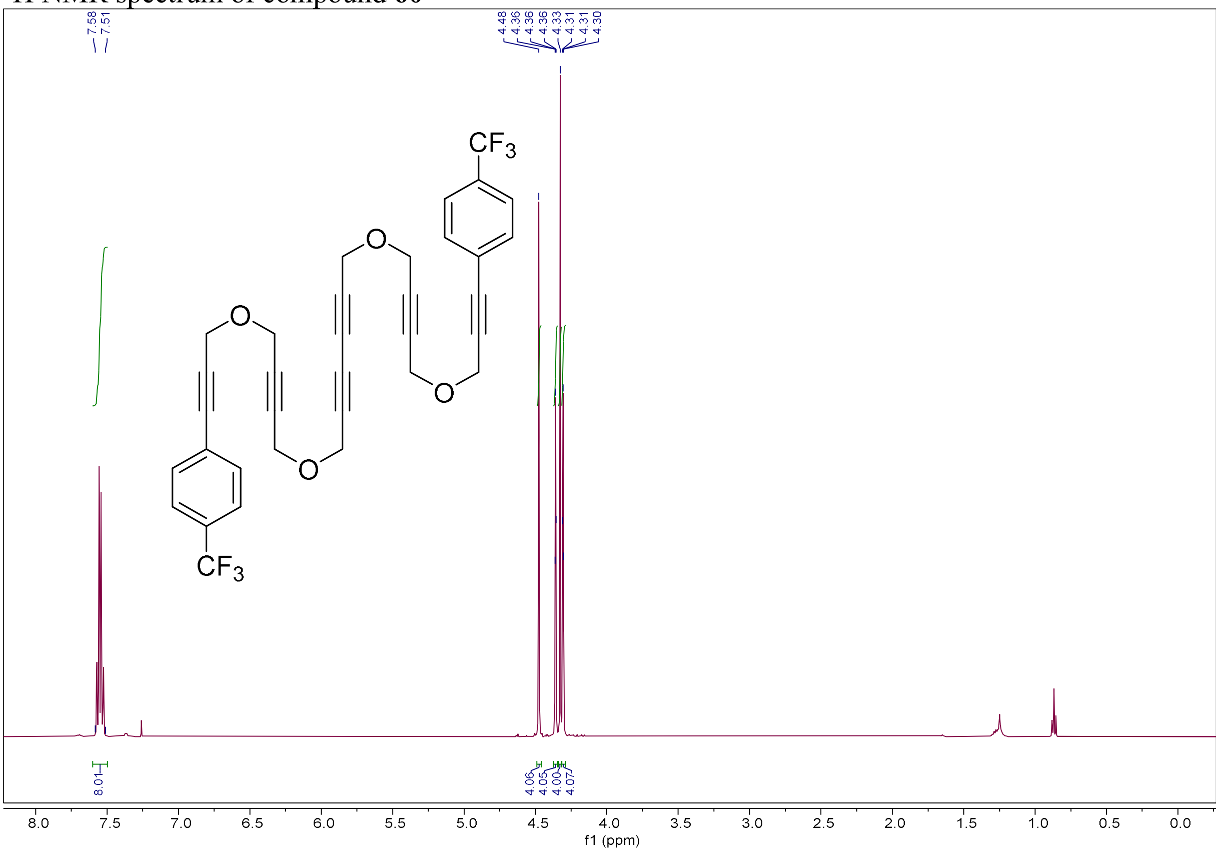

$^{13}C\{^{1}H\}$ NMR spectrum of **60**

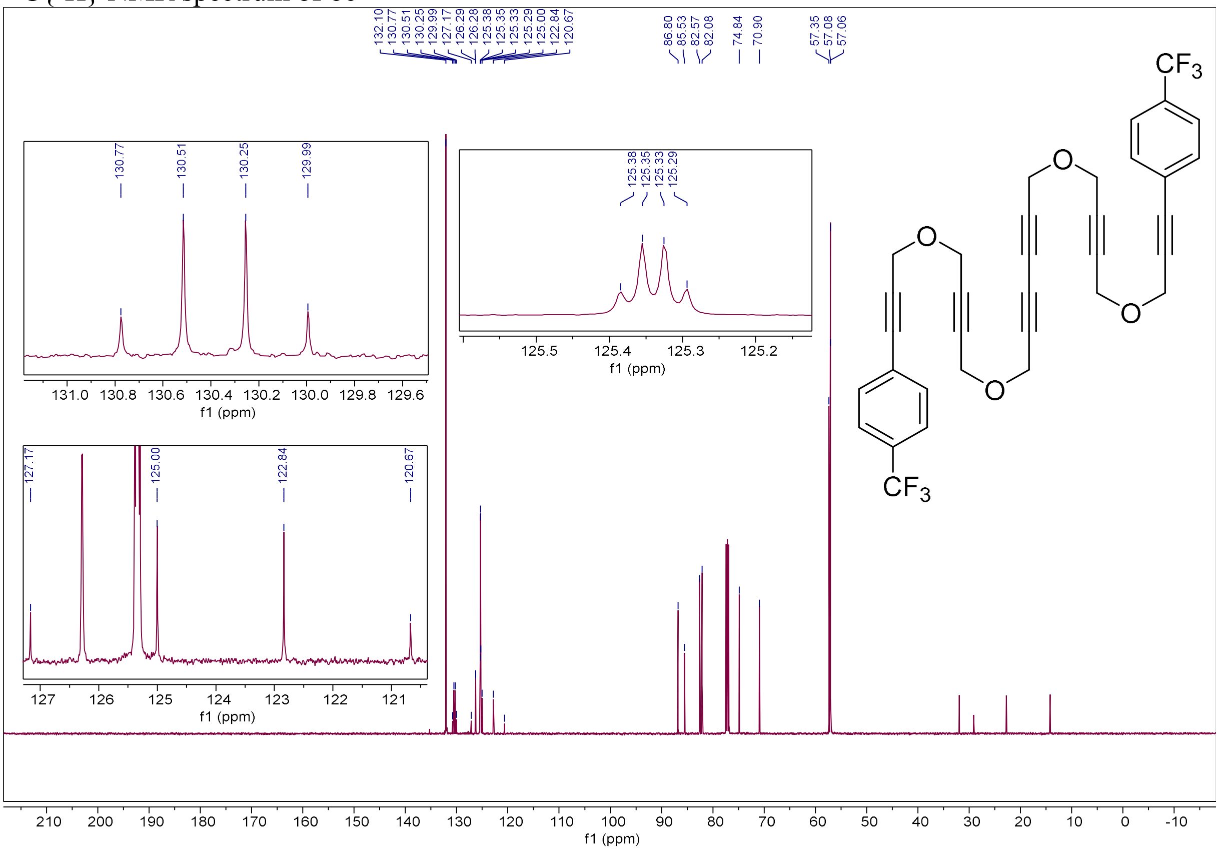

^{19}F NMR spectrum of compound **60**

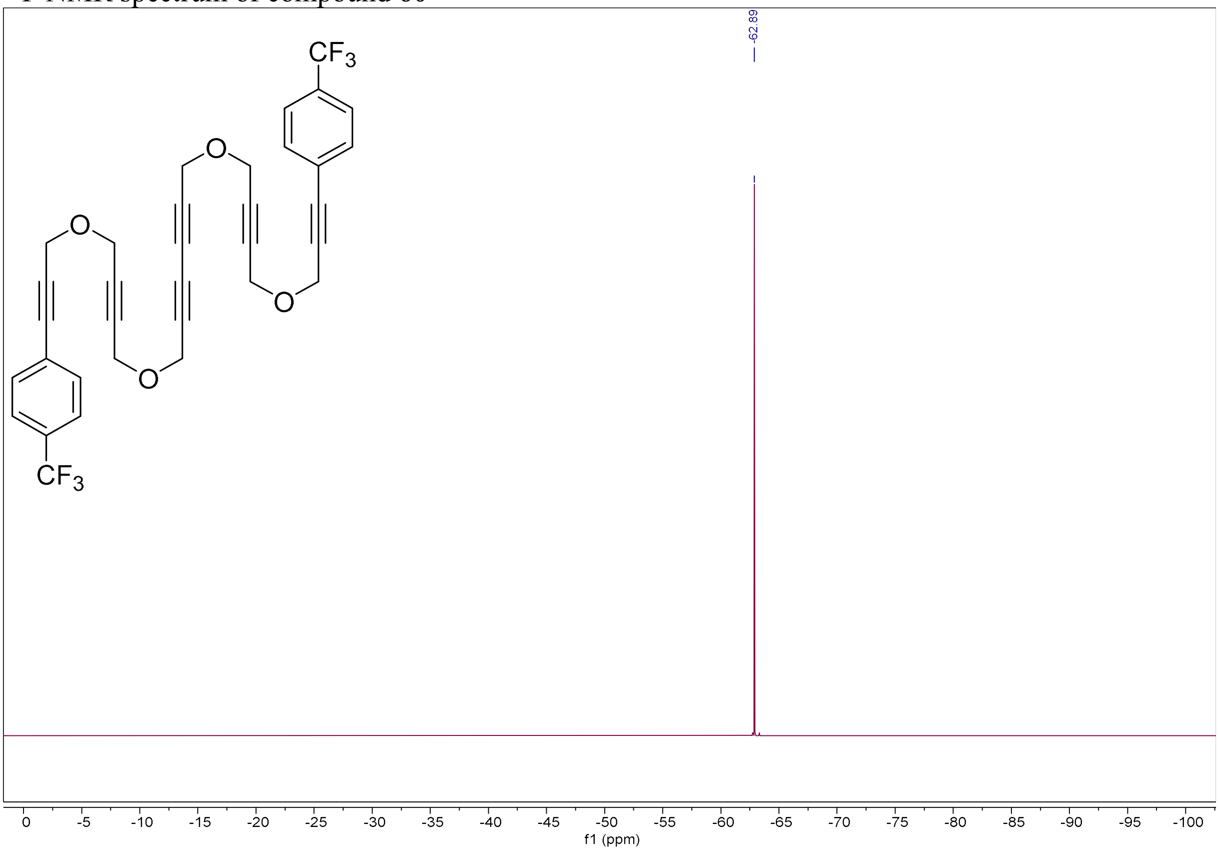

^{1}H NMR spectrum of compound **62**

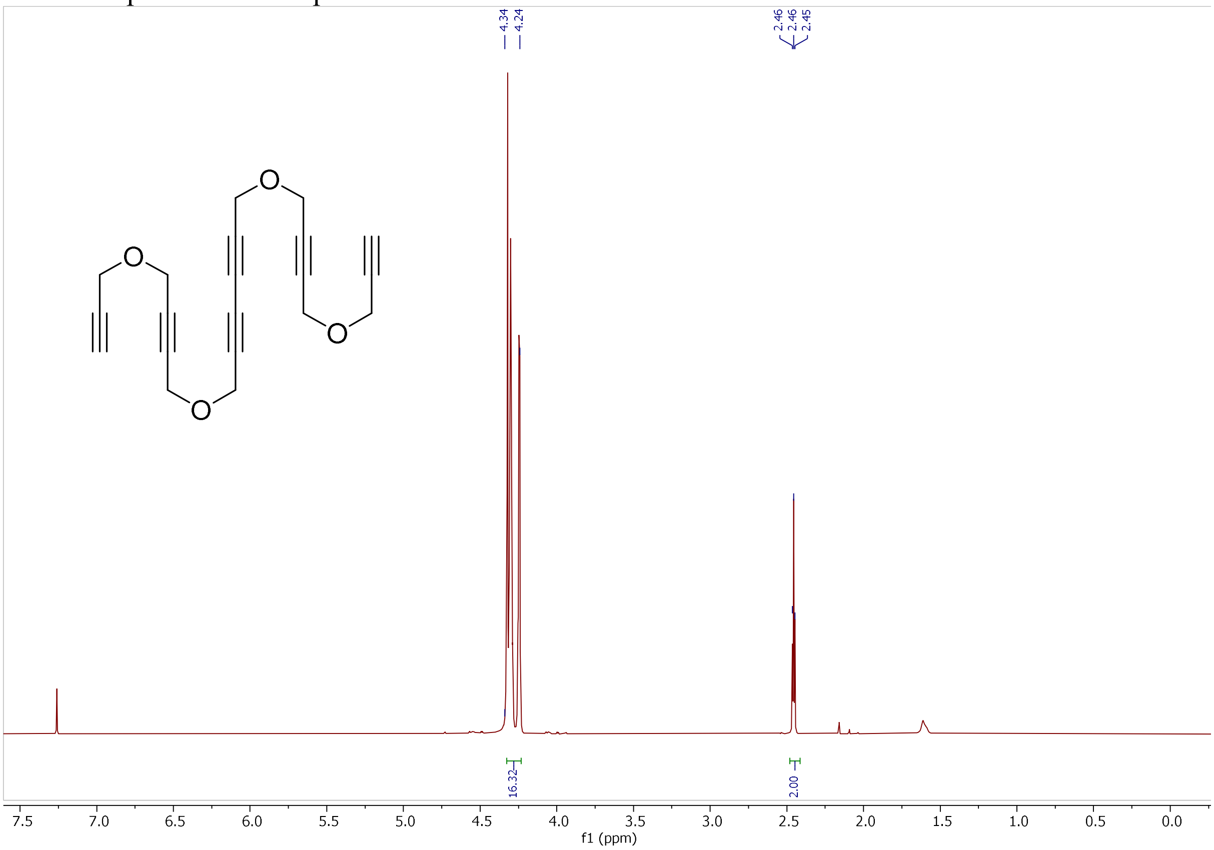

^{13}C{^{1}H} NMR spectrum of **62**

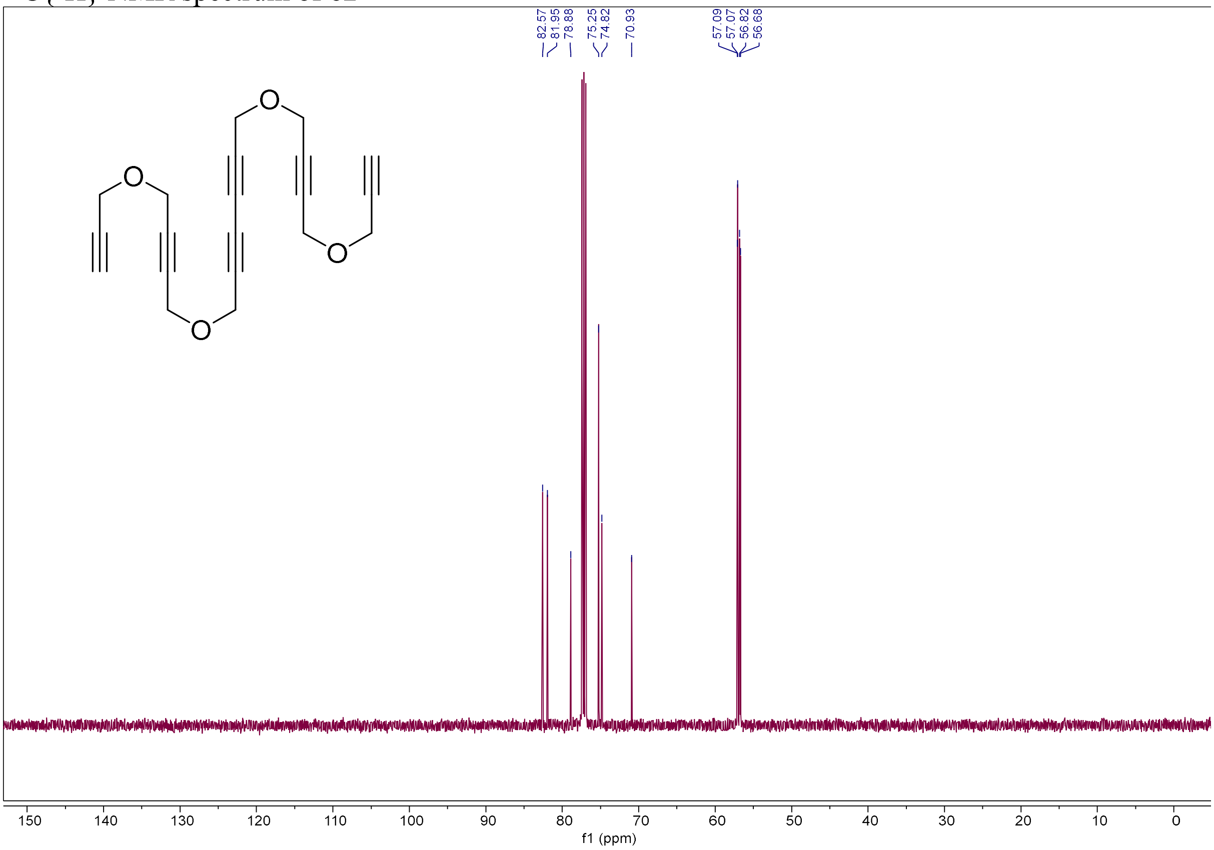

^{1}H NMR spectrum of compound **64**

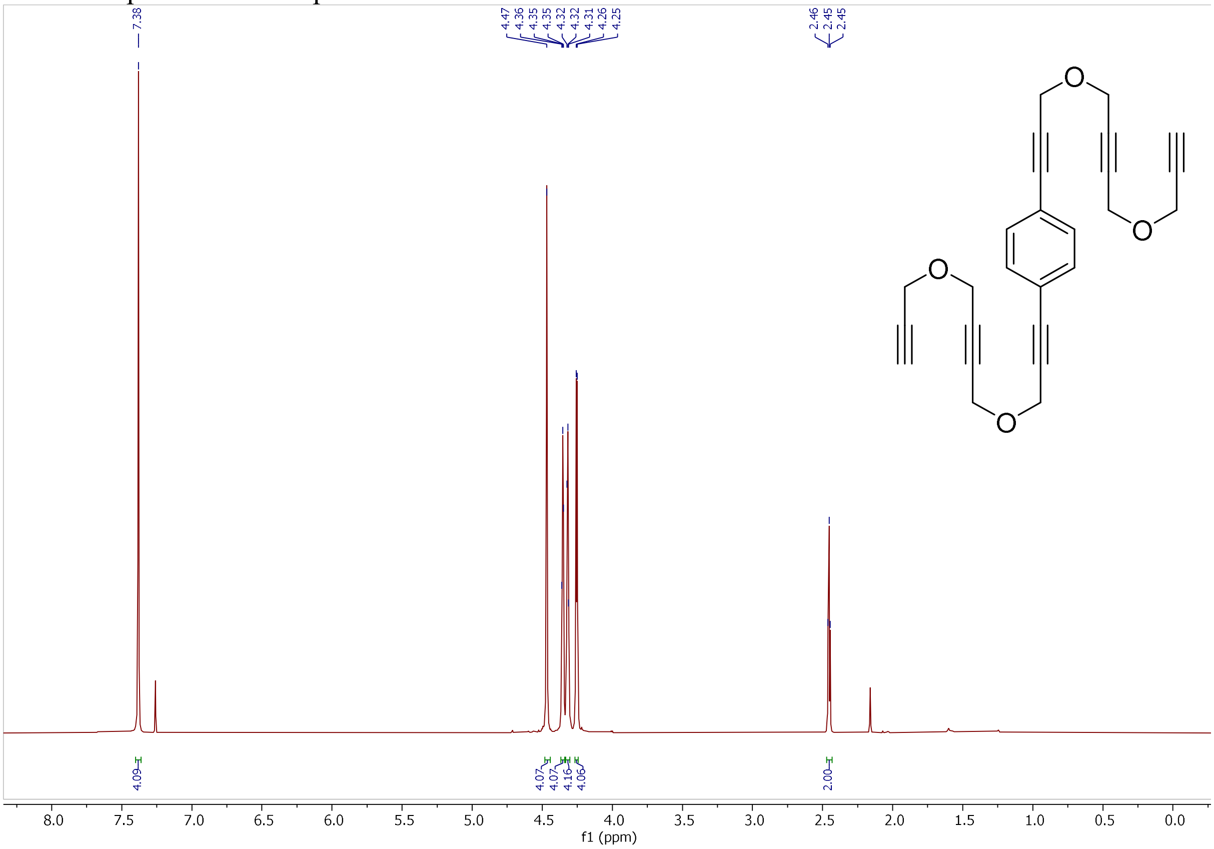

^{13}C{^{1}H} NMR spectrum of **64**

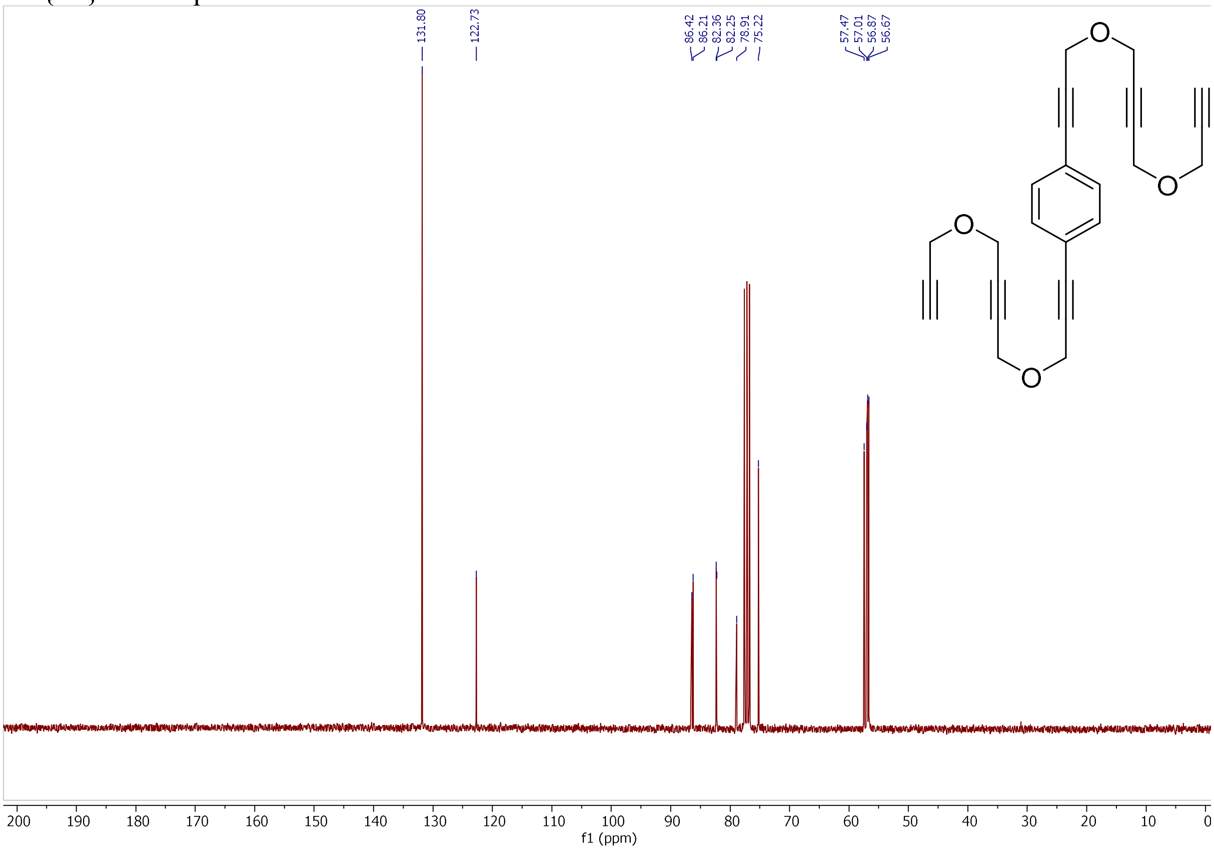

^{1}H NMR spectrum of compound **66**

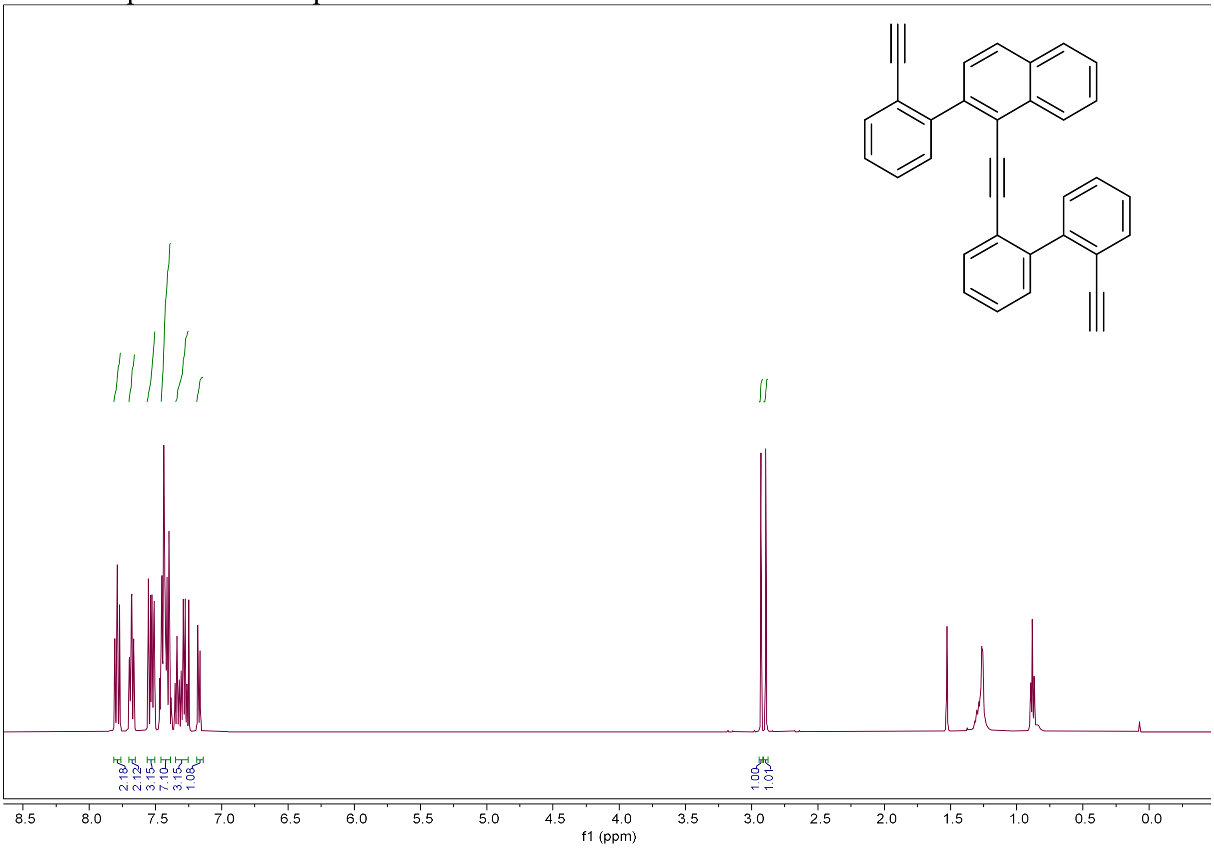

^{1}H NMR spectrum of compound **SI-16**

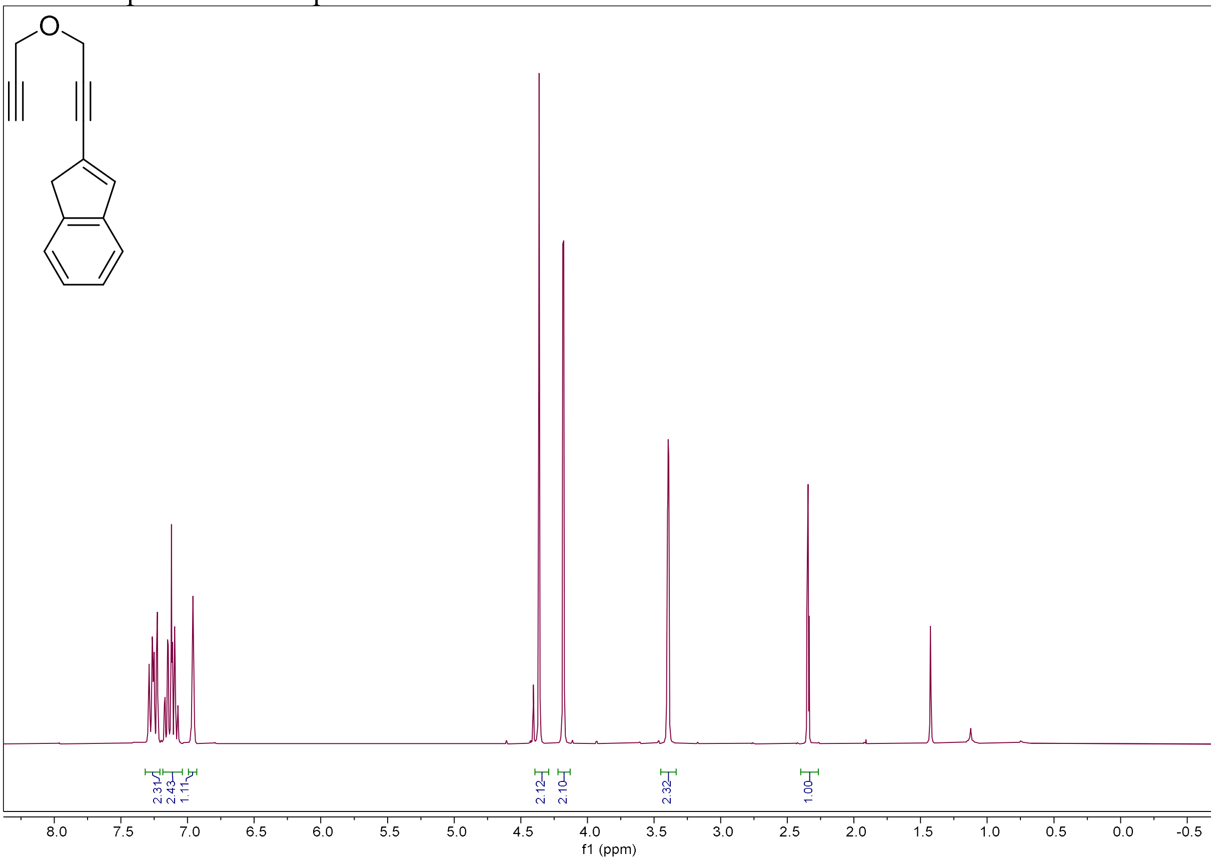

$^{13}C\{^1H\}$ NMR spectrum of **SI-16**

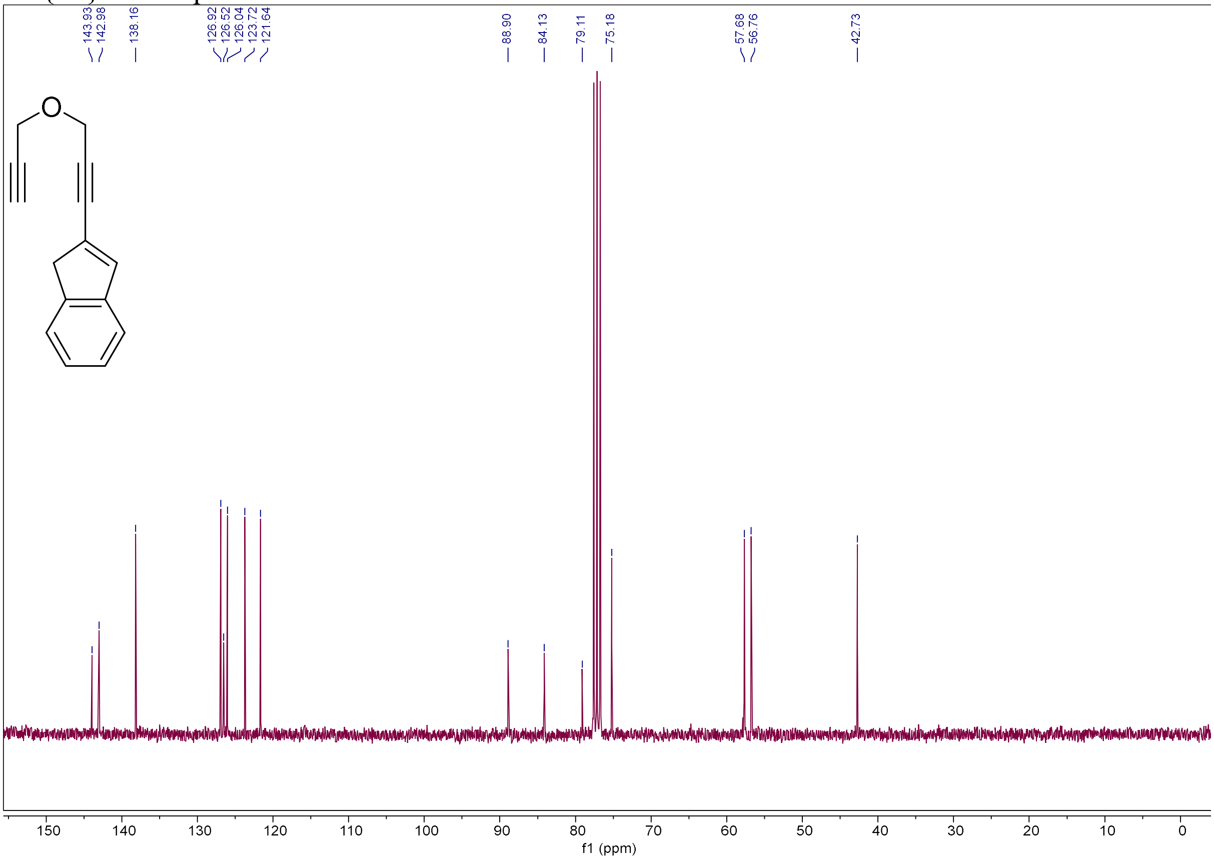

^1H NMR spectrum of compound **SI-10**

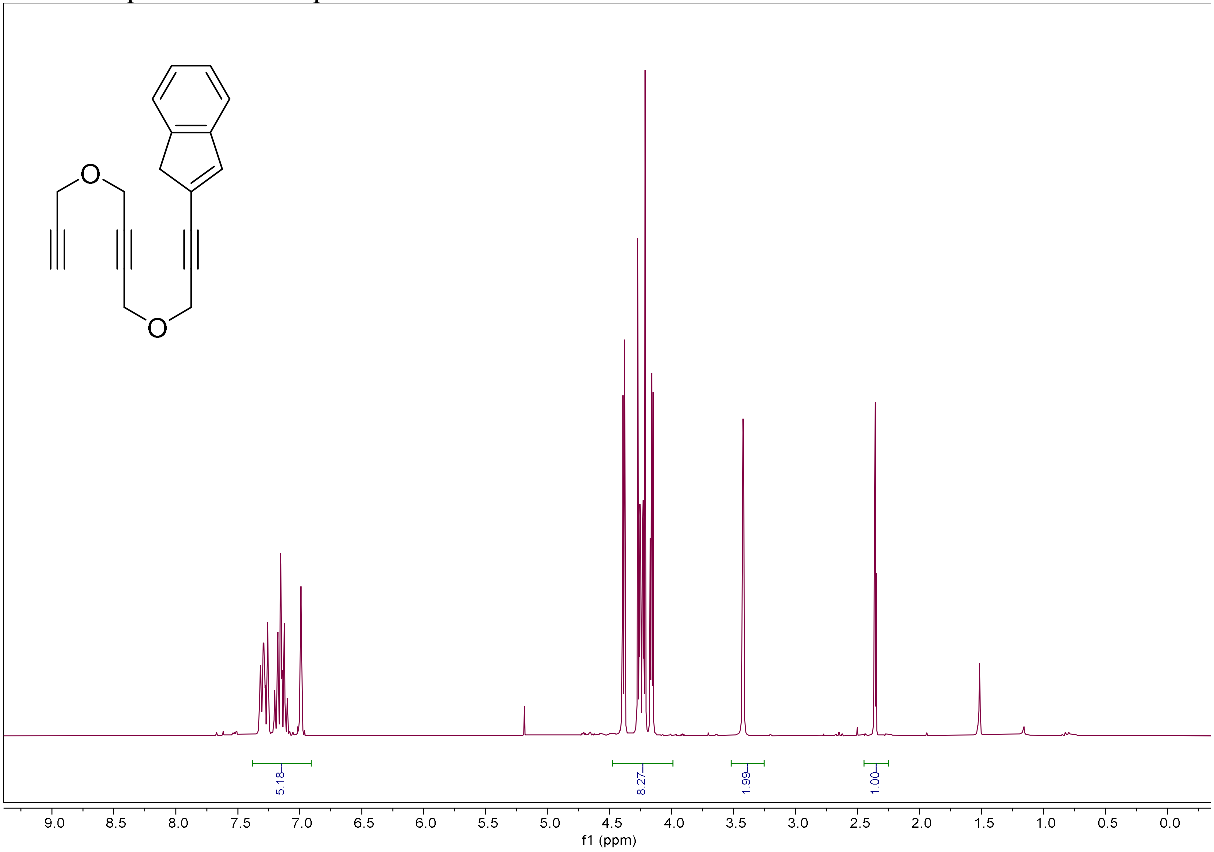

$^{13}C\{^1H\}$ NMR spectrum of **SI-10**

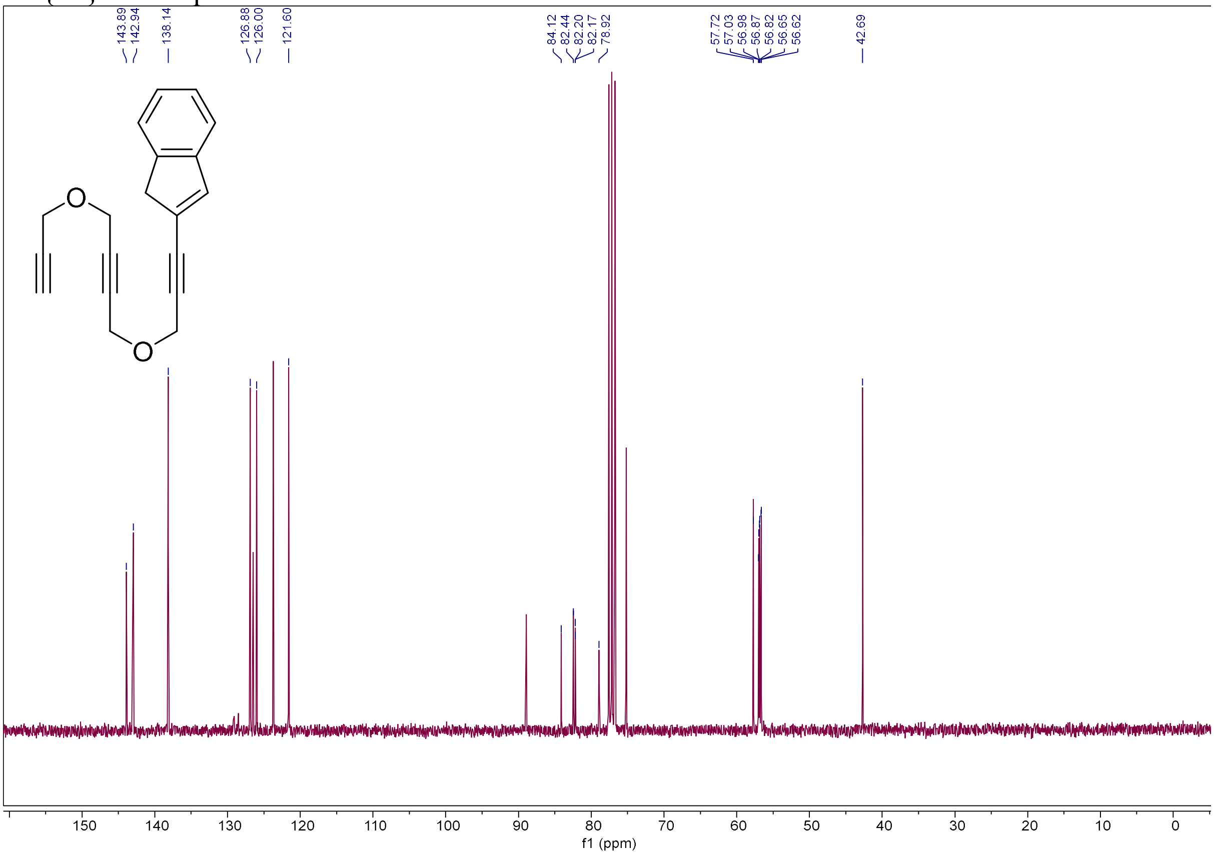

^1H NMR spectrum of diethyl 2,2-di(prop-2-yn-1-yl)malonate

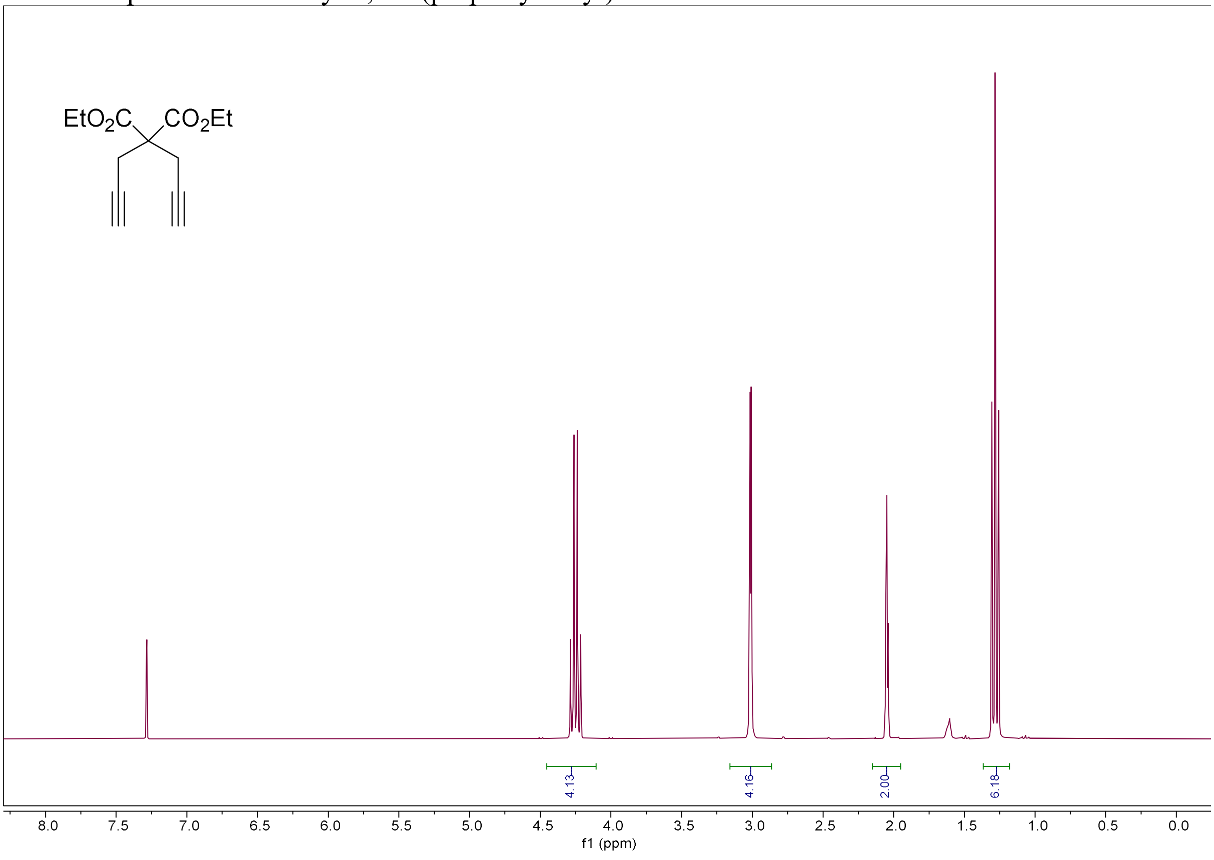

^{1}H NMR spectrum of compound **PA1**

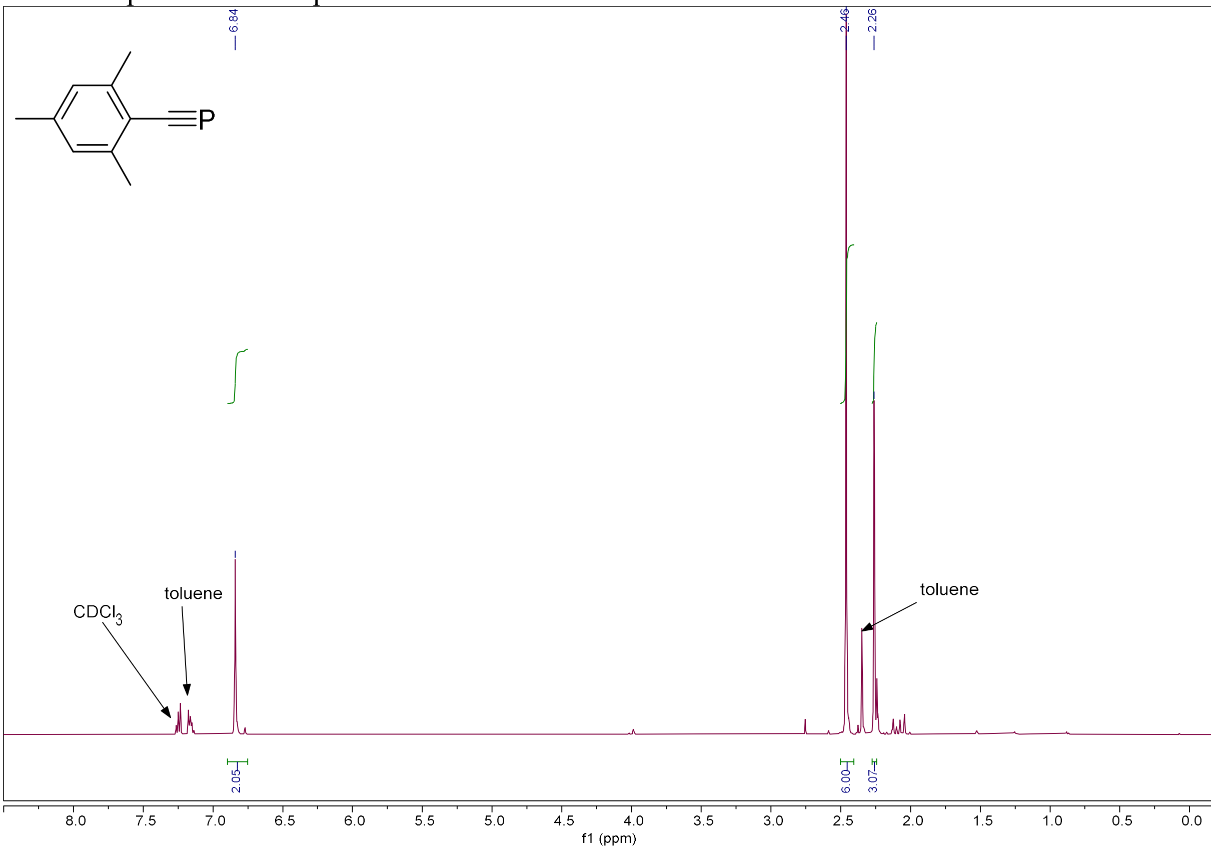

^{31}P{^{1}H} NMR spectrum of compound **PA1**

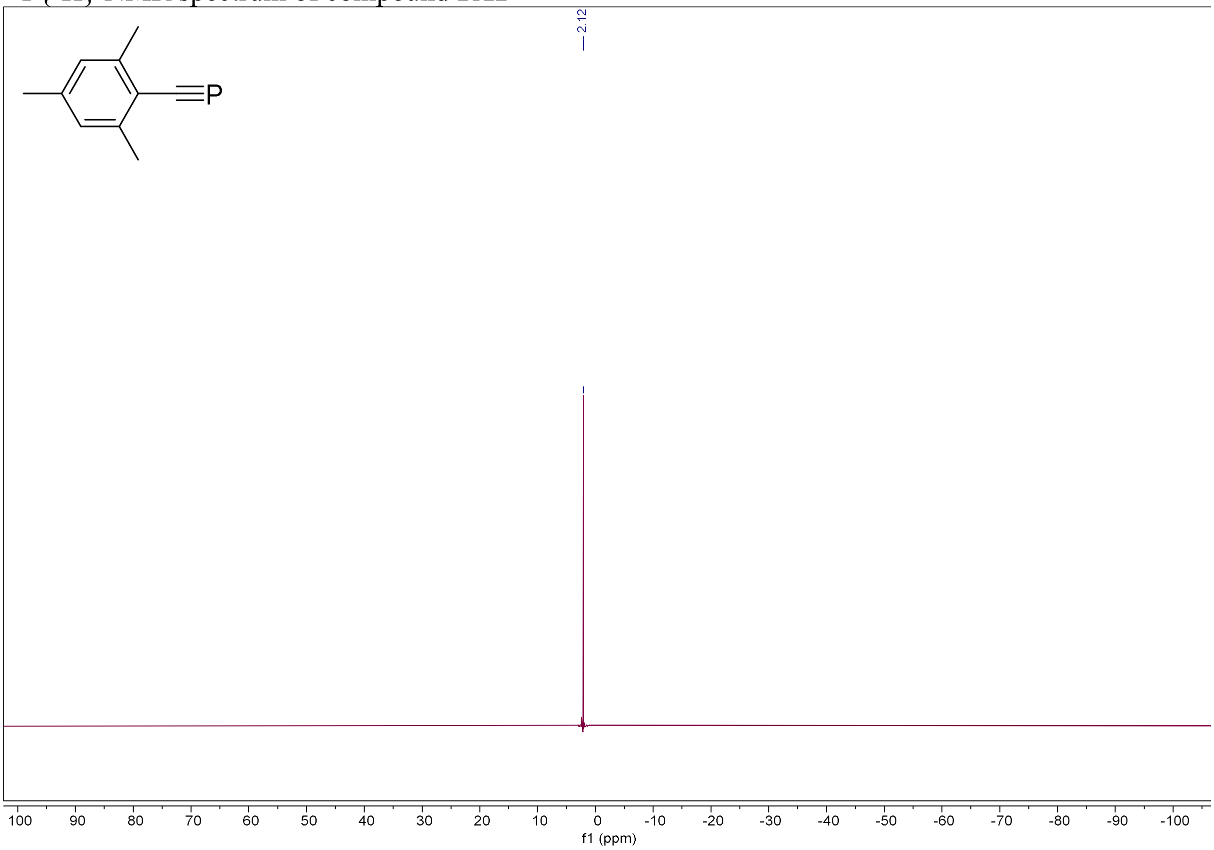

^{1}H NMR spectrum of compound **SI-2**

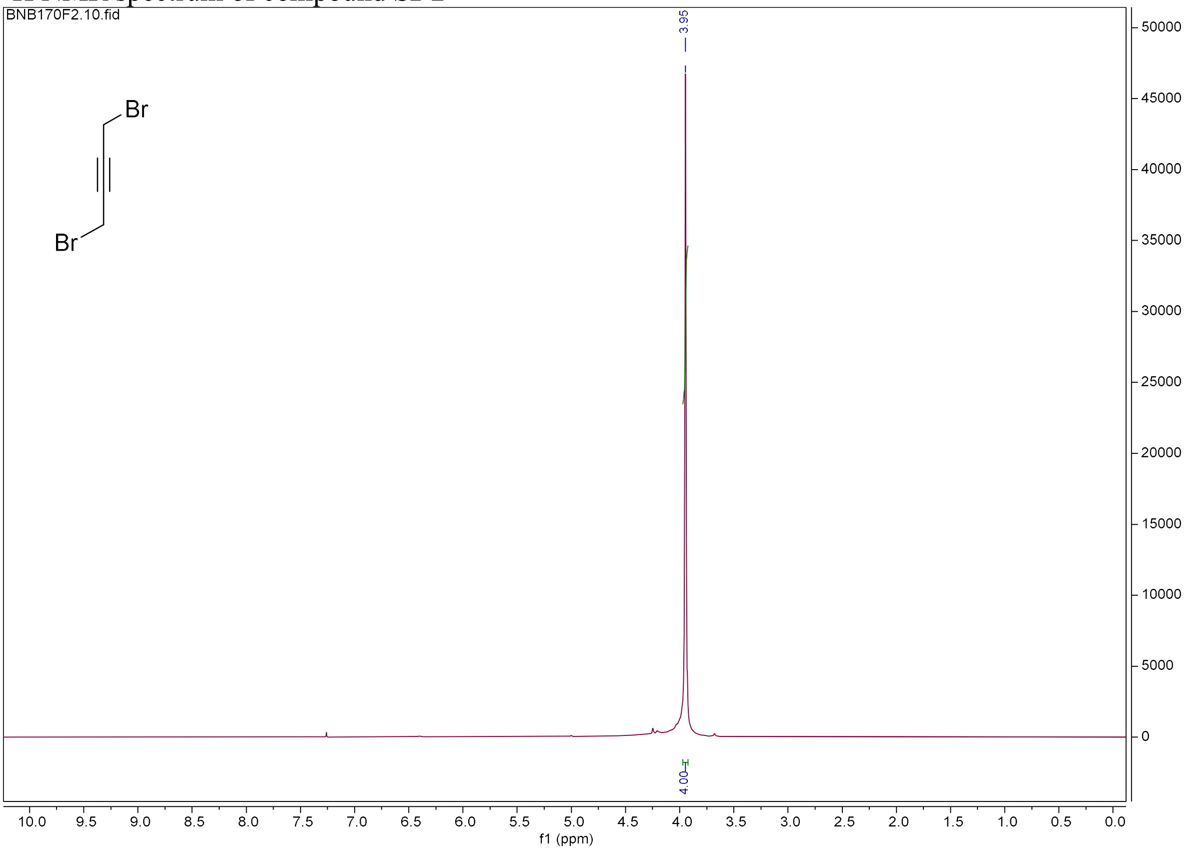

^{1}H NMR spectrum of compound **SI-3**

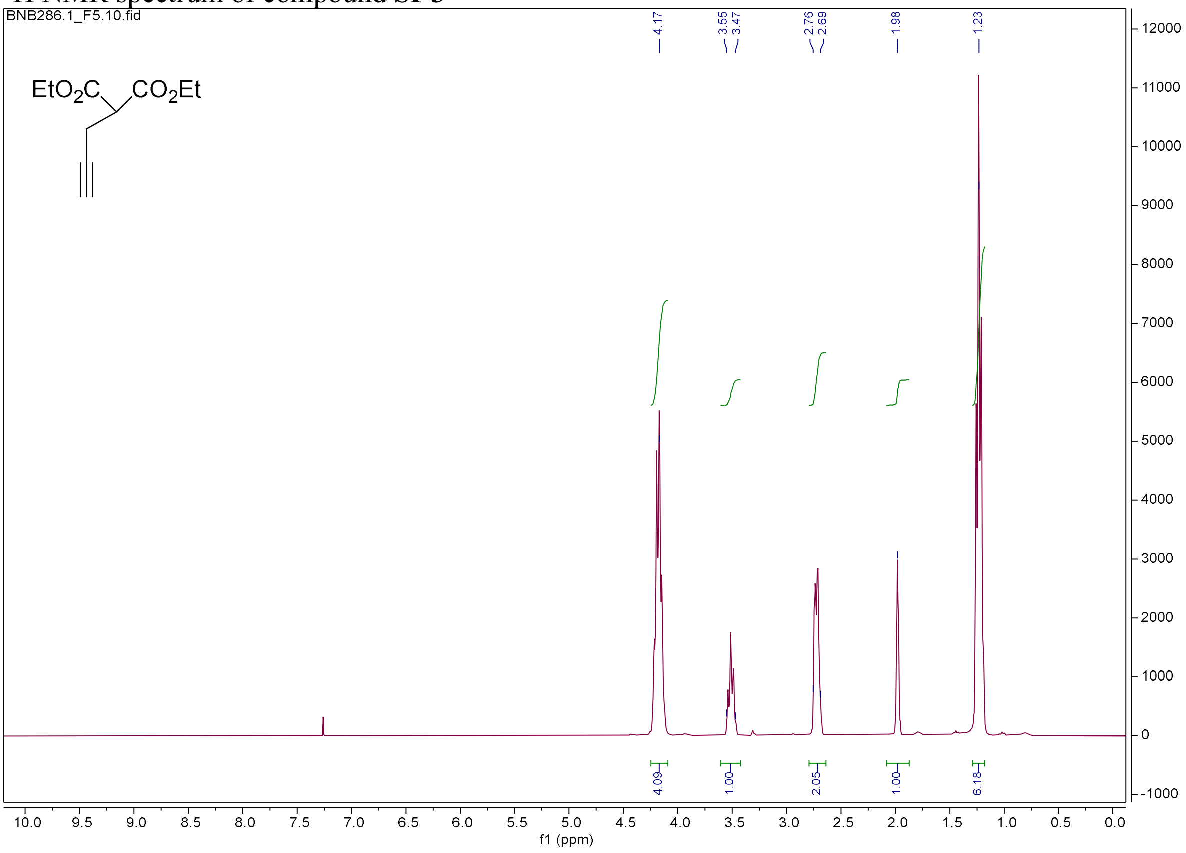

^{1}H NMR spectrum of compound **SI-4**

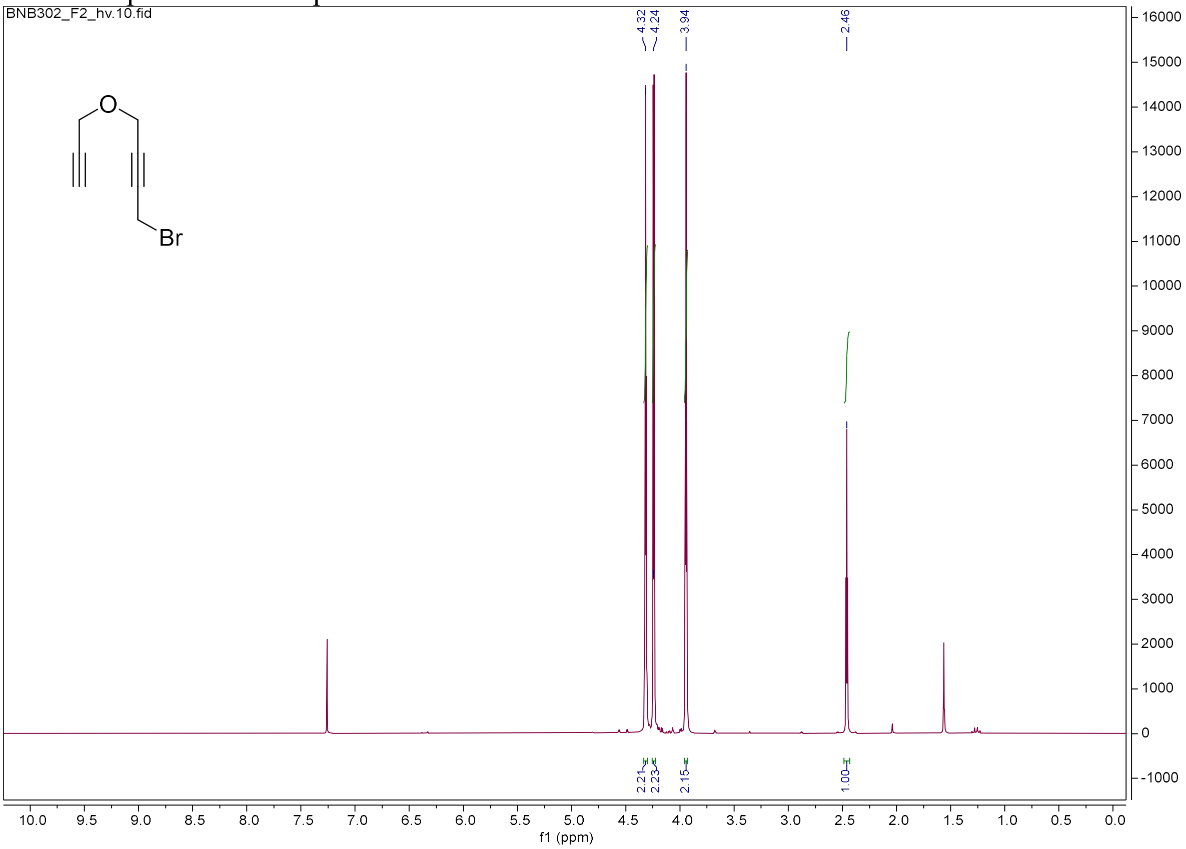

^{1}H NMR spectrum of compound **SI-5**

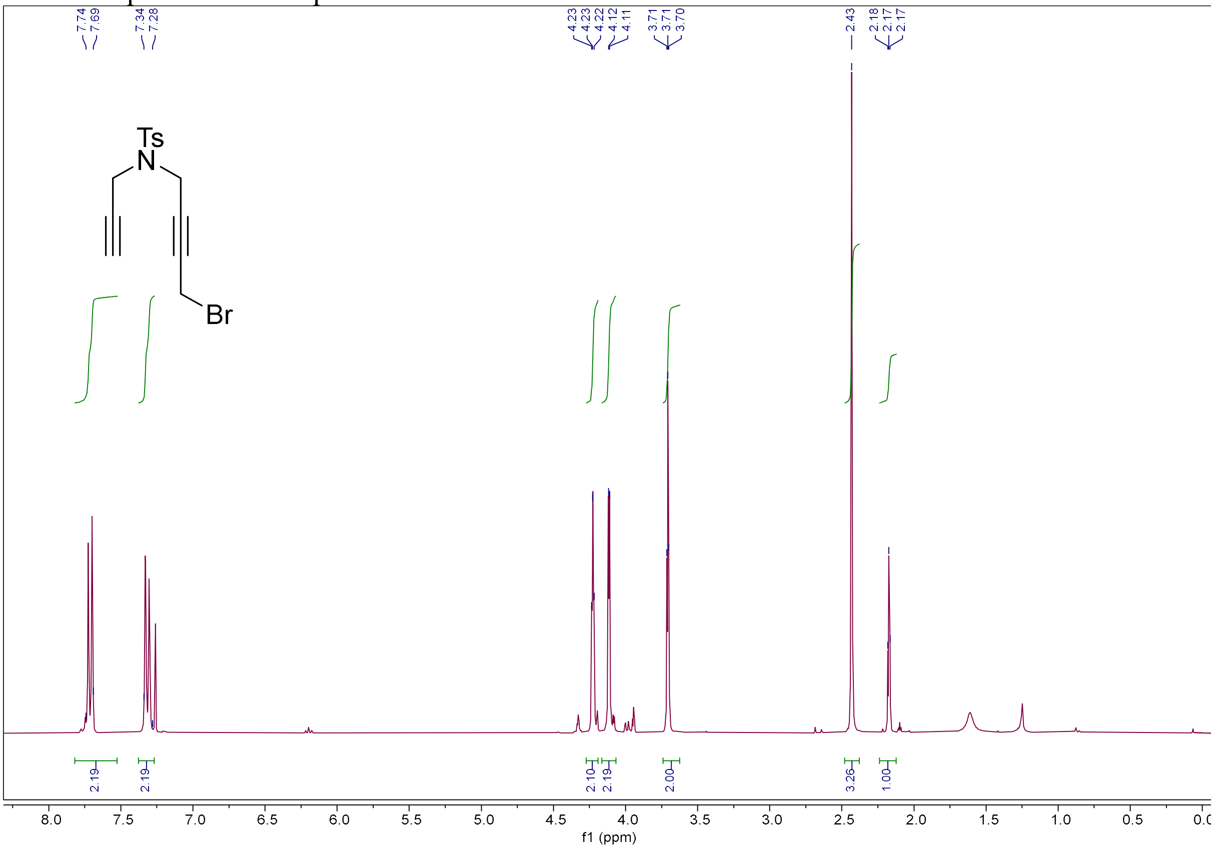

^{1}H NMR spectrum of compound **SI-6**

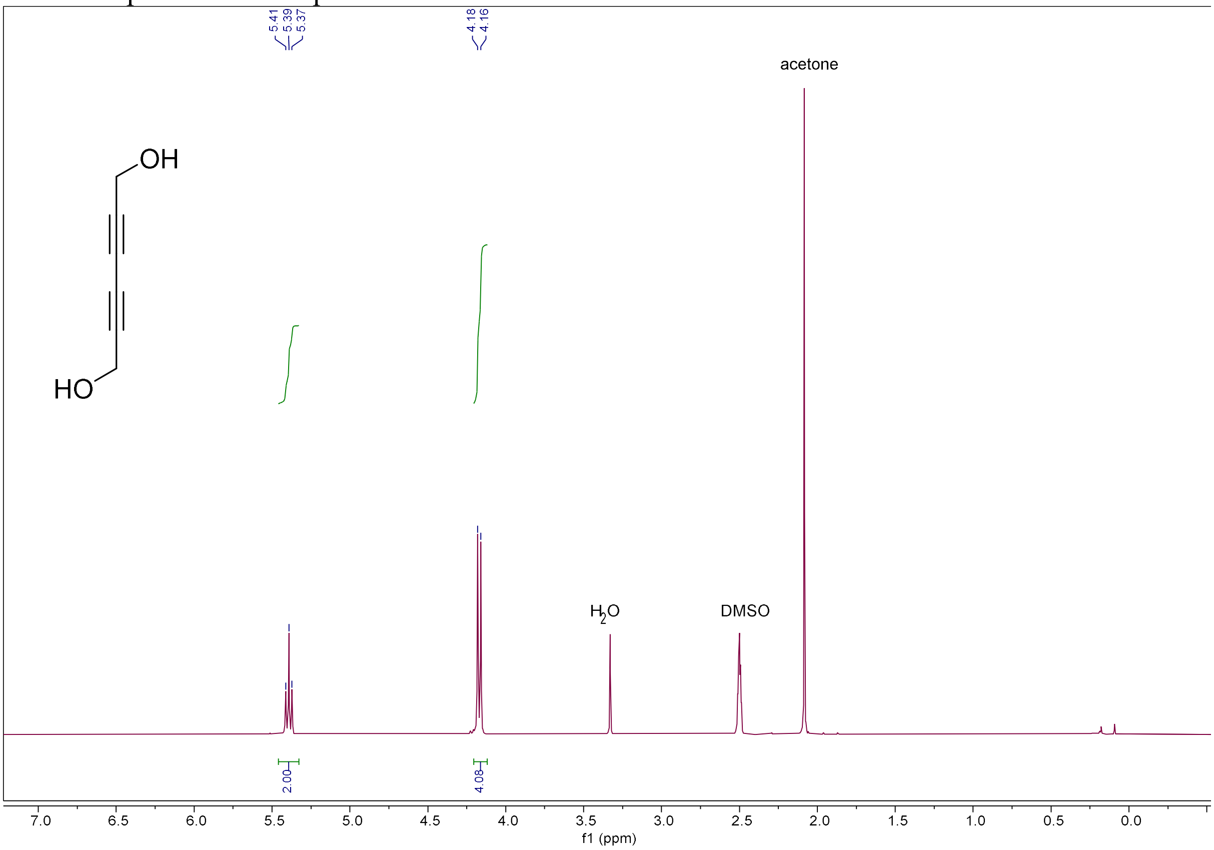

^{1}H NMR spectrum of compound **SI-7**

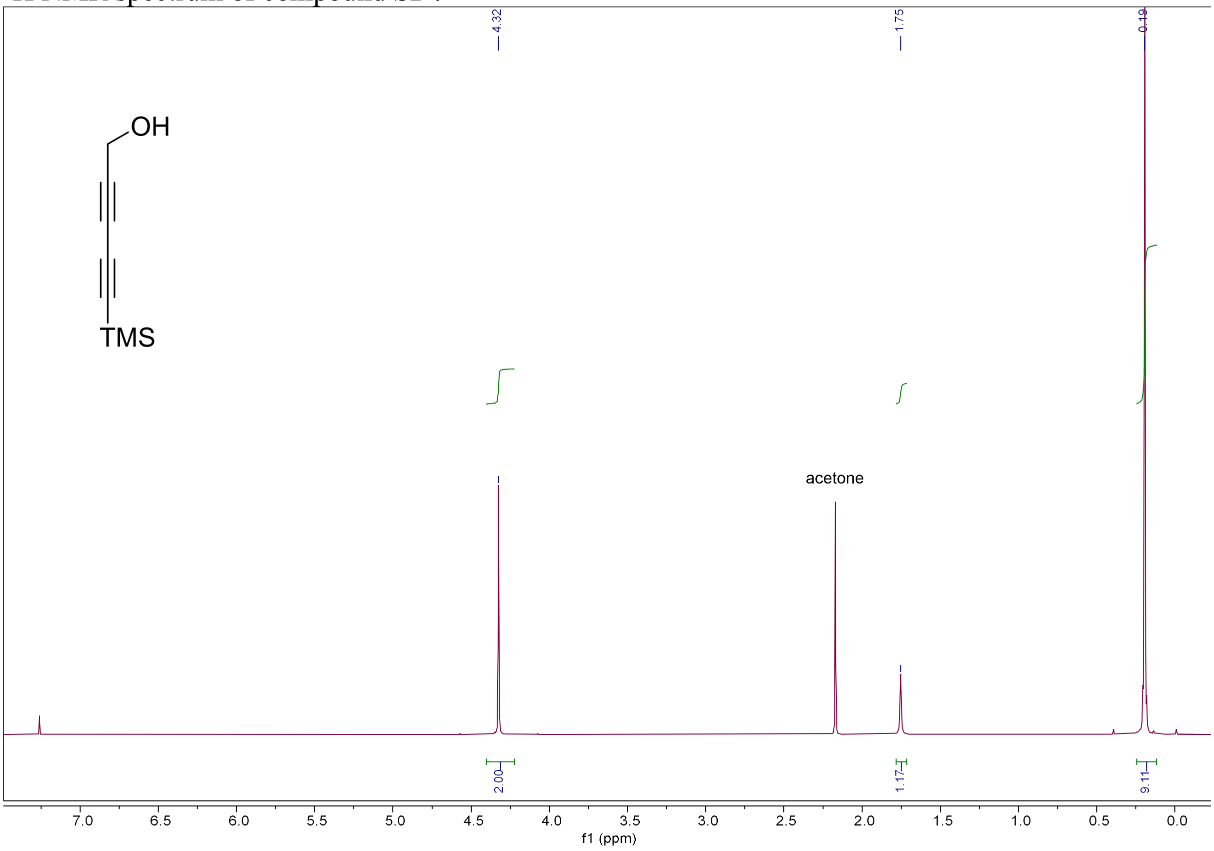

^{1}H NMR spectrum of compound **SI-8**

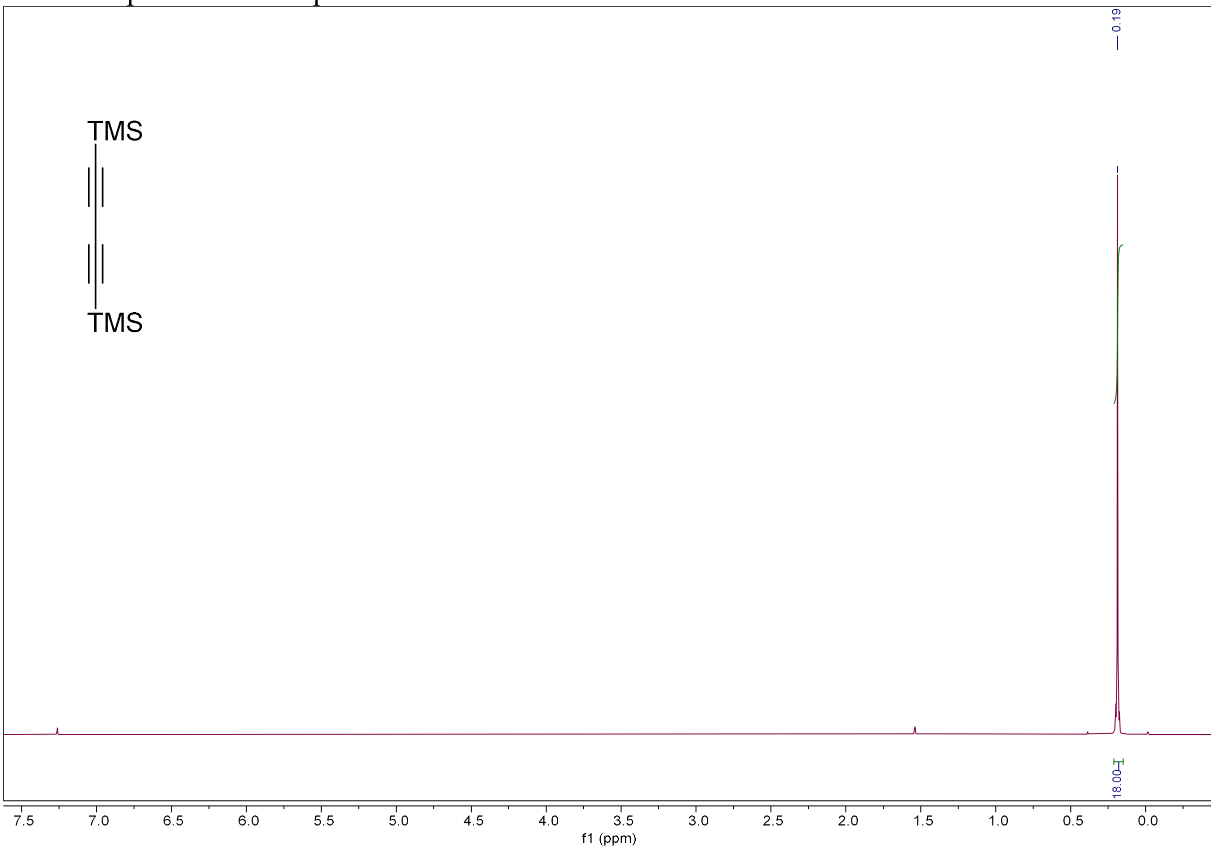

^{1}H NMR spectrum of compound **SI-11**

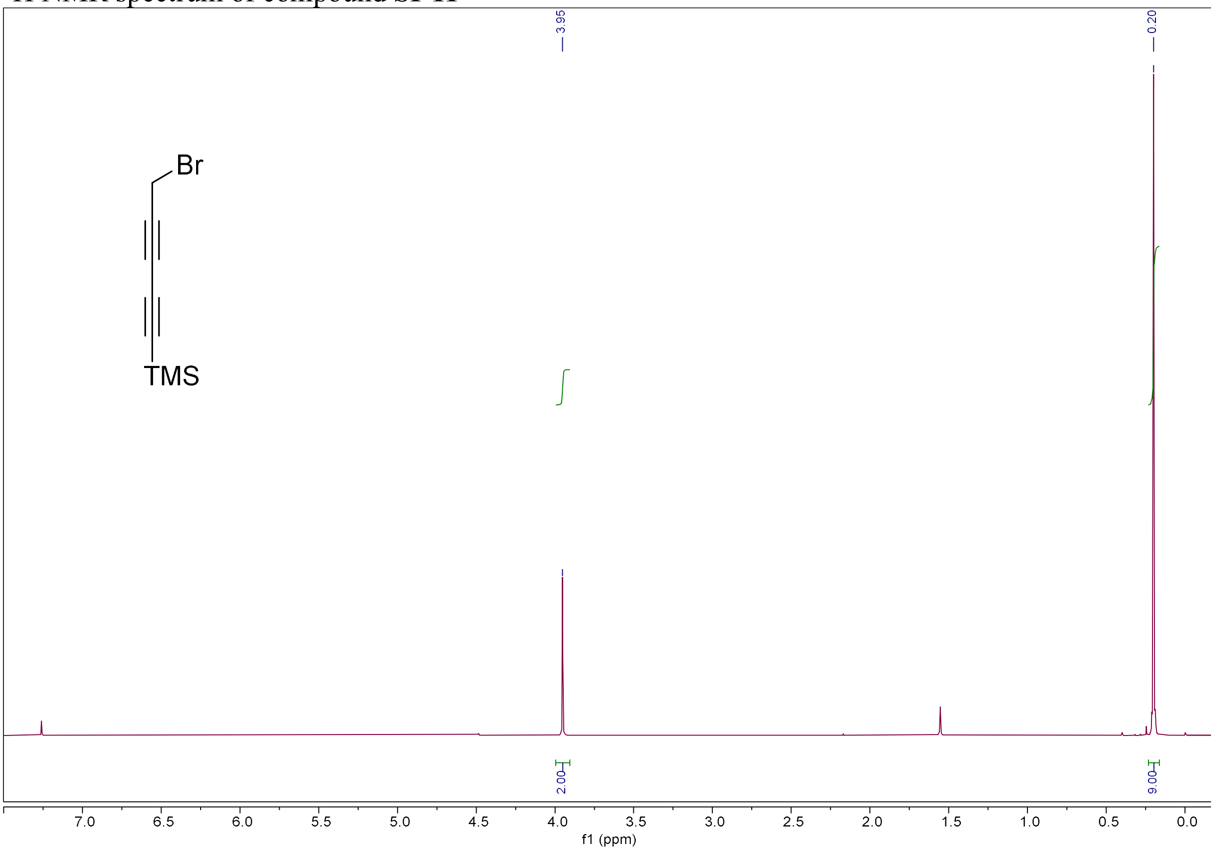

^{1}H NMR spectrum of compound **SI-12**

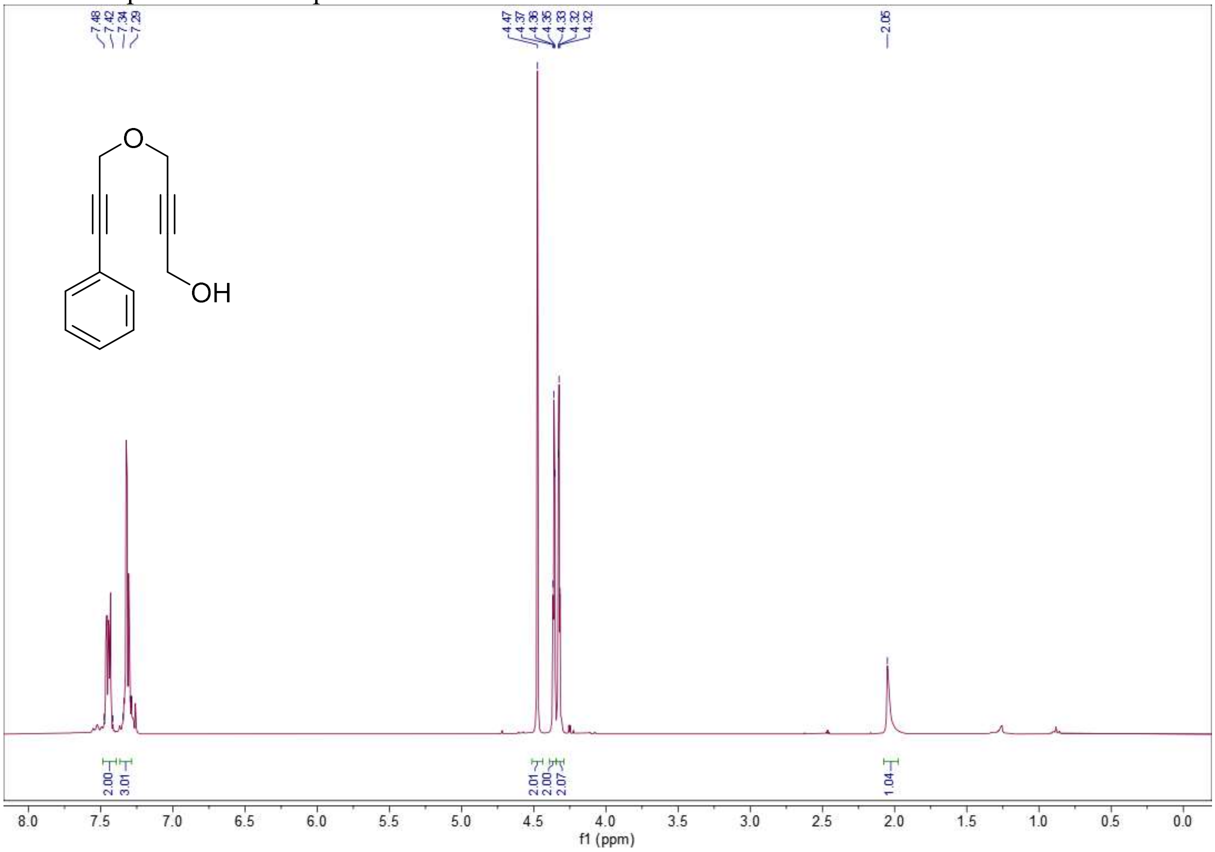

^{1}H NMR spectrum of compound **SI-13**

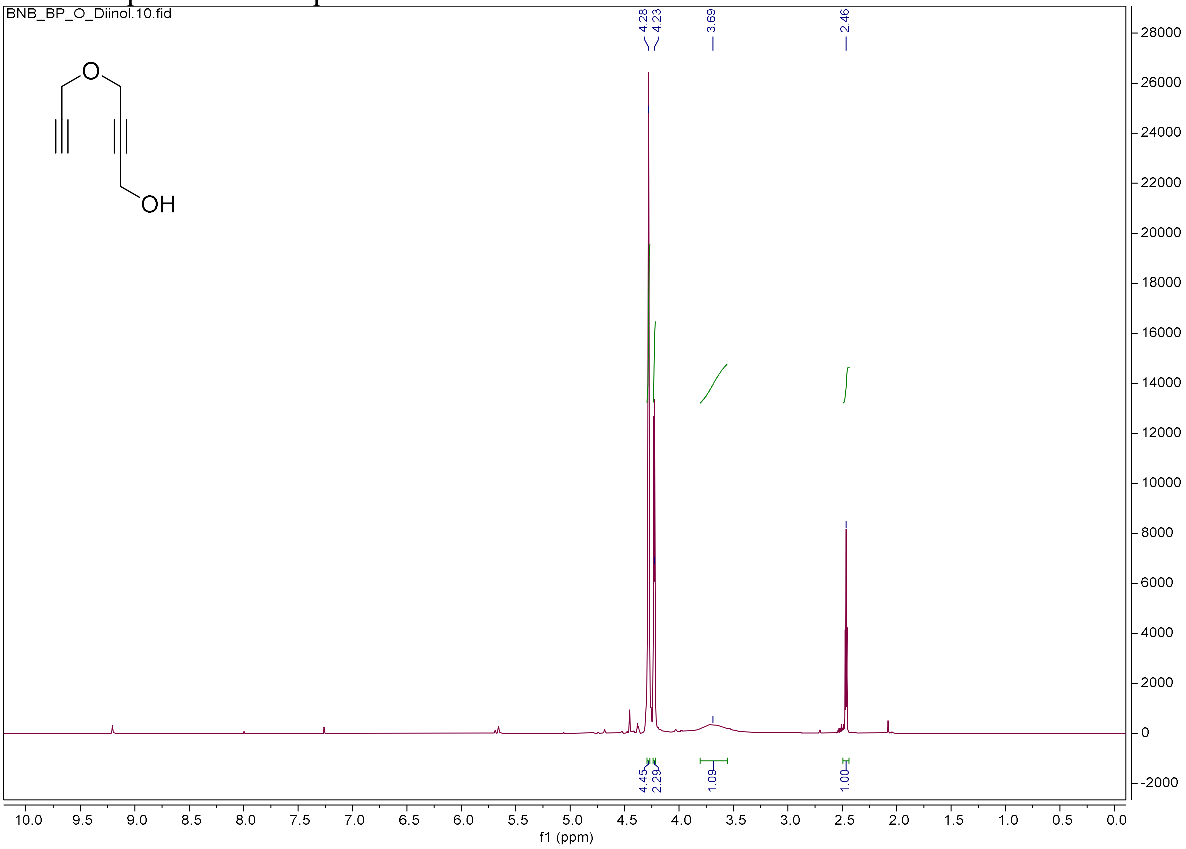

^{1}H NMR spectrum of compound **SI-15**

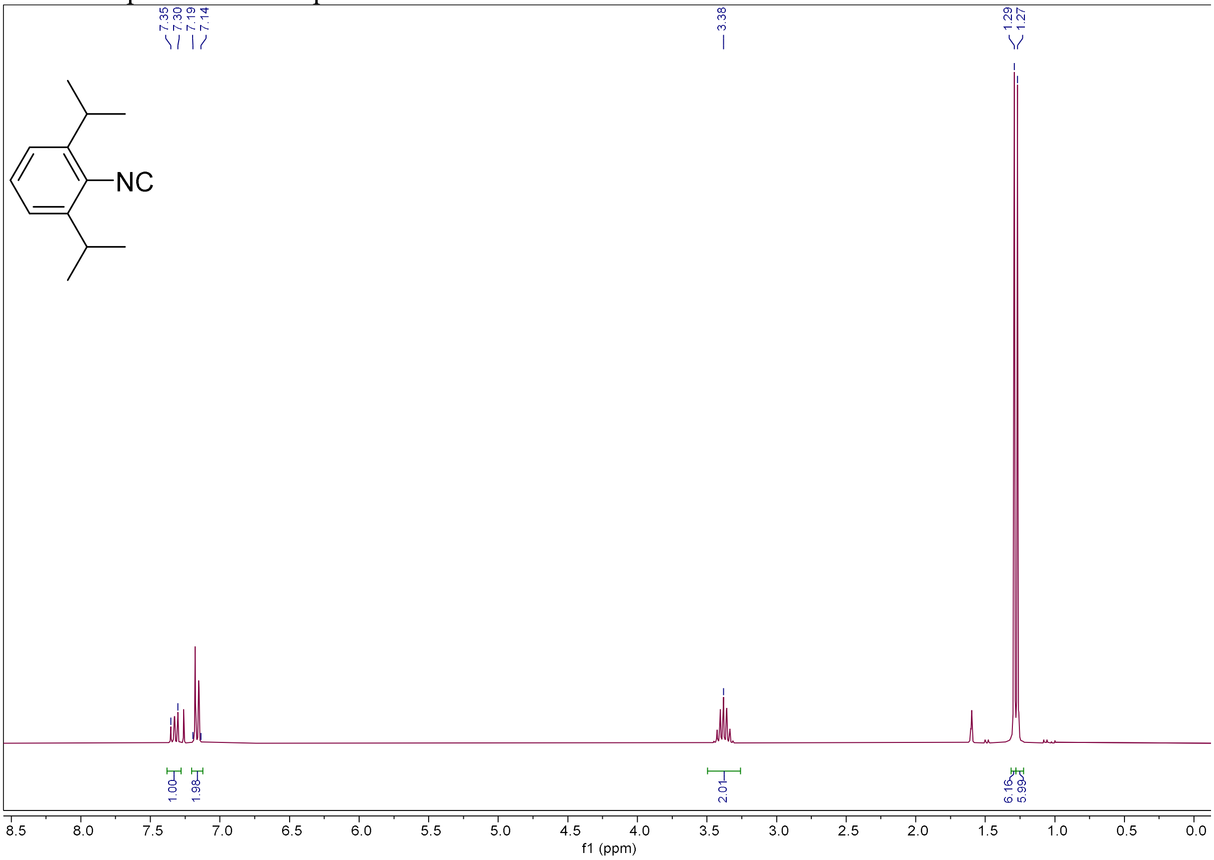

4 Mechanistic Investigations

4.1 NMR spectroscopic investigations

^{31}P NMR for the irradiation of a sample of MnBr(dppm)(CO)$_3$ (**Mn7**) (1 mM in toluene), irradiation by blue LED strip (10 W, 21 units, λ_{max} = 467 nm, manufacturer: Ledxon) at room temperature (Figure SI-6).

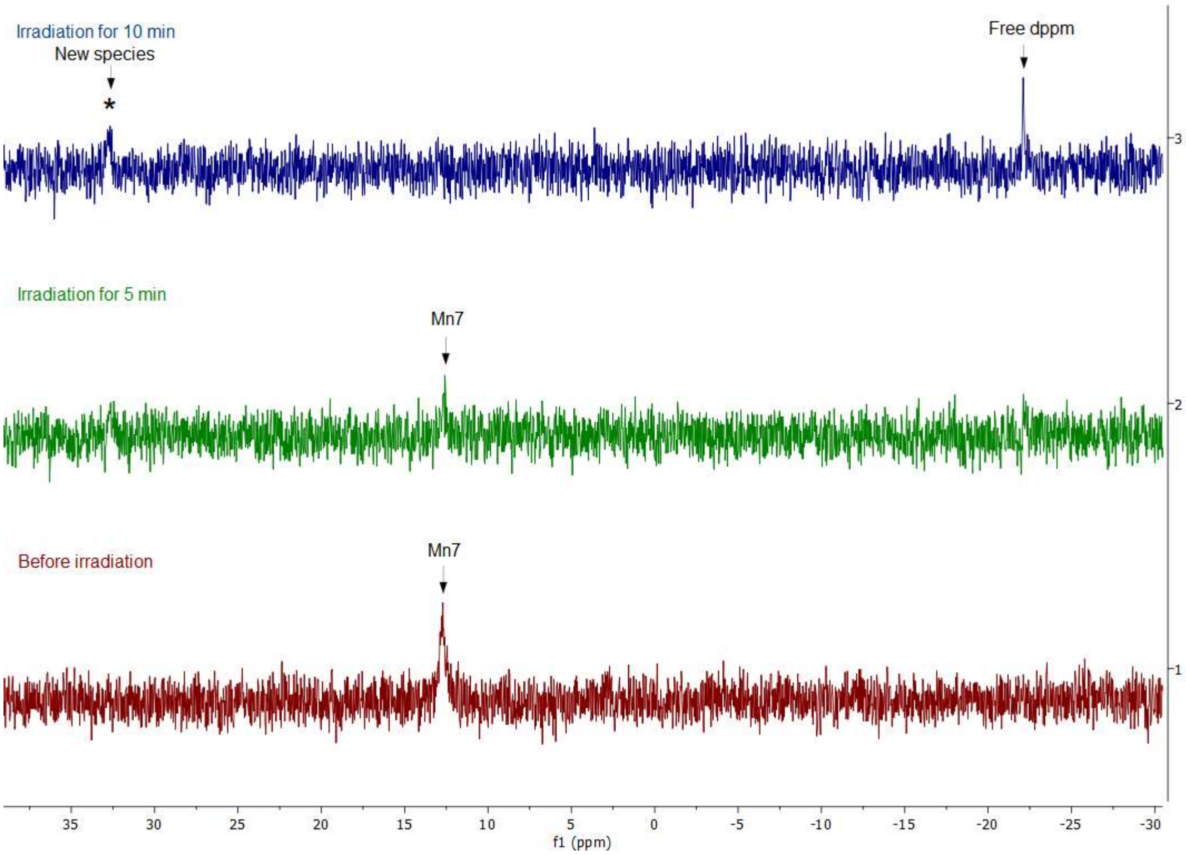

Figure SI-6: ^{31}P NMR study of **Mn7** at different irradiation times.

The activation of precatalyst **Mn7** was investigated by performing ^{31}P NMR studies at different irradiation times. The initial signal for the ligand P atoms in the pre-catalyst is recorded at 12.7 ppm. Upon irradiation, this signal decreased and disappeared completely after 10 minutes. It was replaced by the appearances of two new resonances, at -22 ppm for free dppm ligand and at 32.6 ppm for a new species, which has not been further identified. Besides, broadening of peaks in ^{1}H NMR upon irradiation was observed (Figure S1), revealing the formation of paramagnetic species in the system.

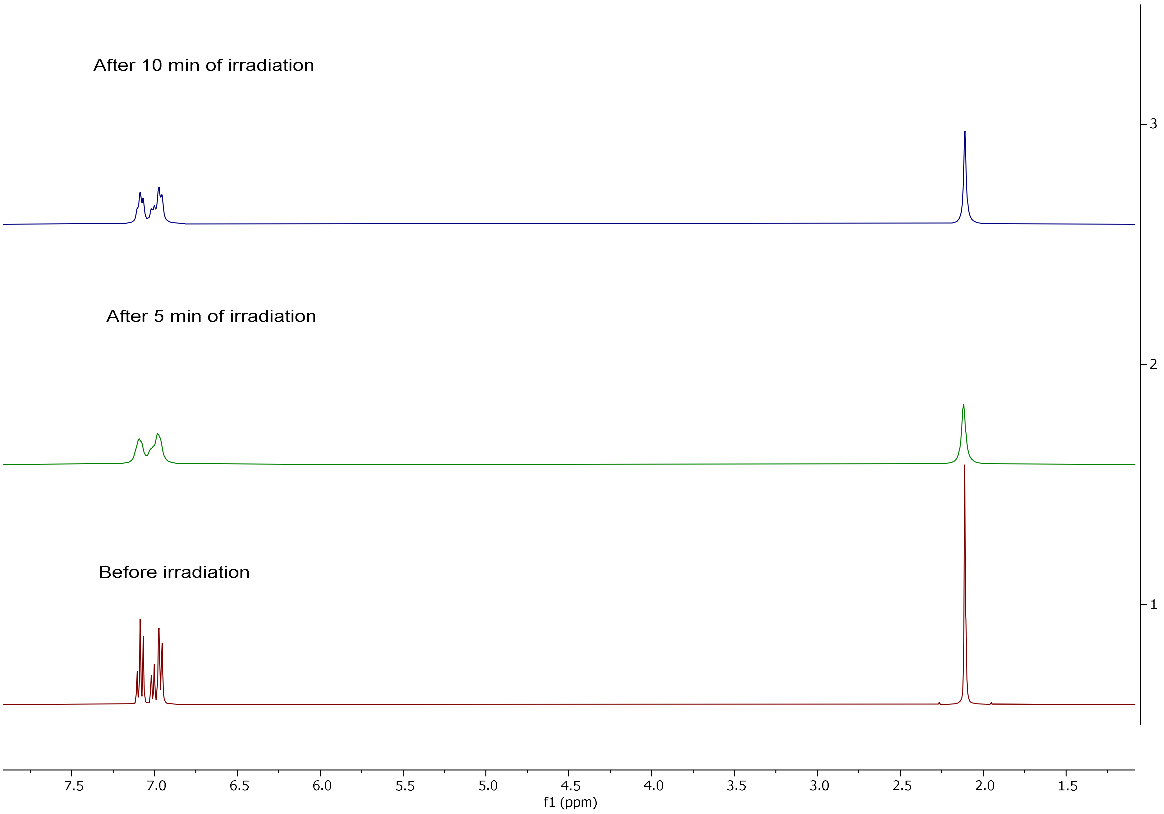

Figure SI-7: ^{1}H NMR spectra of precatalyst **Mn7** after different irradiation time.

^{31}P NMR for the irradiation of an in situ mixture of $MnBr(CO)_5$ (**Mn1**) (1 mM) and dppp (**L8**) (1mM), irradiation by blue LED strip (10 W, 21 units, λ_{max} = 467 nm, manufacturer: Ledxon) at room temperature (Figure SI-8).

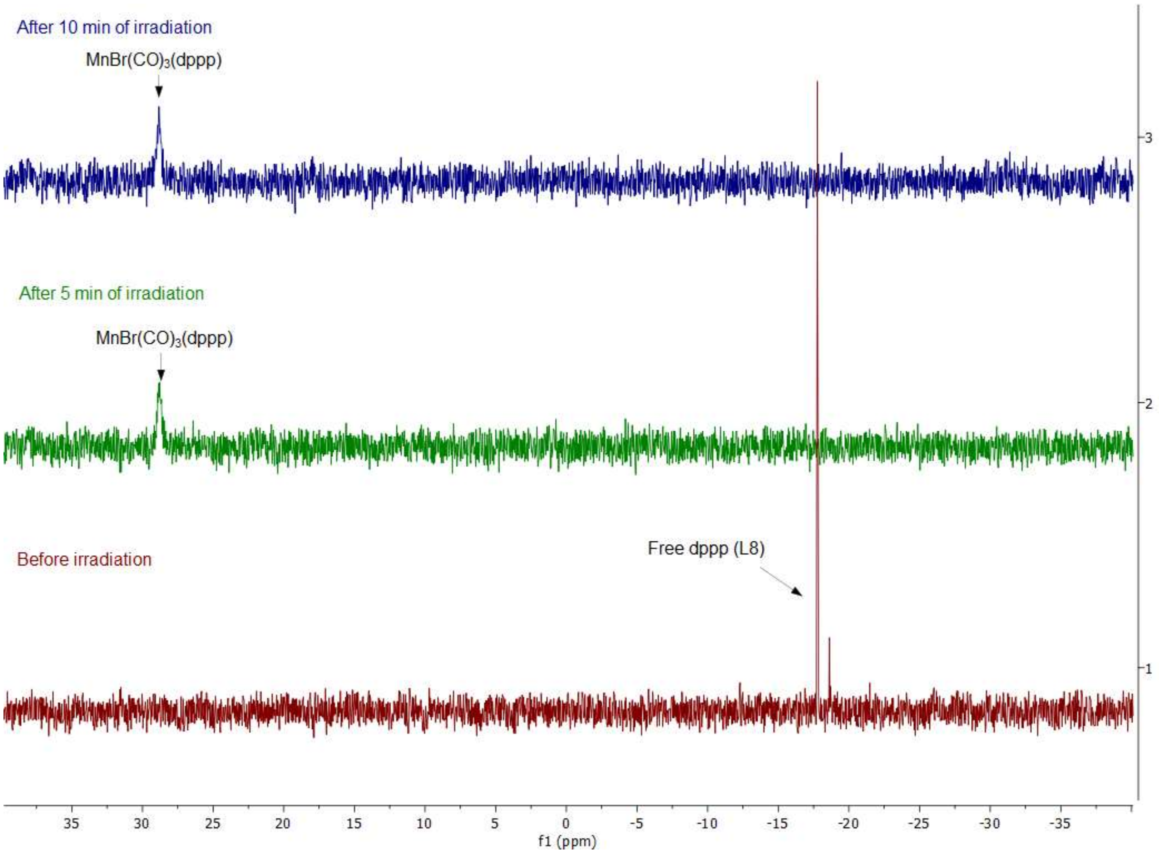

Figure SI-8: ^{31}P NMR study of a 1:1 mixture of **Mn1/dppp (L8)** at different irradiation times.

The irradiation of a mixture of $MnBr(CO)_5$ (**Mn1**) and dppp (**L8**) as bidentate ligand with a larger dihedral angle led to the formation of stable *in situ* generated $MnBr(CO)_3$(dppp) complex, as suggested by the appearance of a new resonance at 28.8 ppm, which can be assigned to the $MnBr(CO)_3$(dppp) complex and the disappearance of the free dppp ligand resonance at -17.8 ppm. This new signal did not disappear upon further irradiation, indicating that the formed complex was stable. Accordingly, the signal in the ^{1}H NMR spectrum of this mixture (Figure SI-9) still maintained sharp after irradiating, indicating that no paramagnetic species was formed in this case.

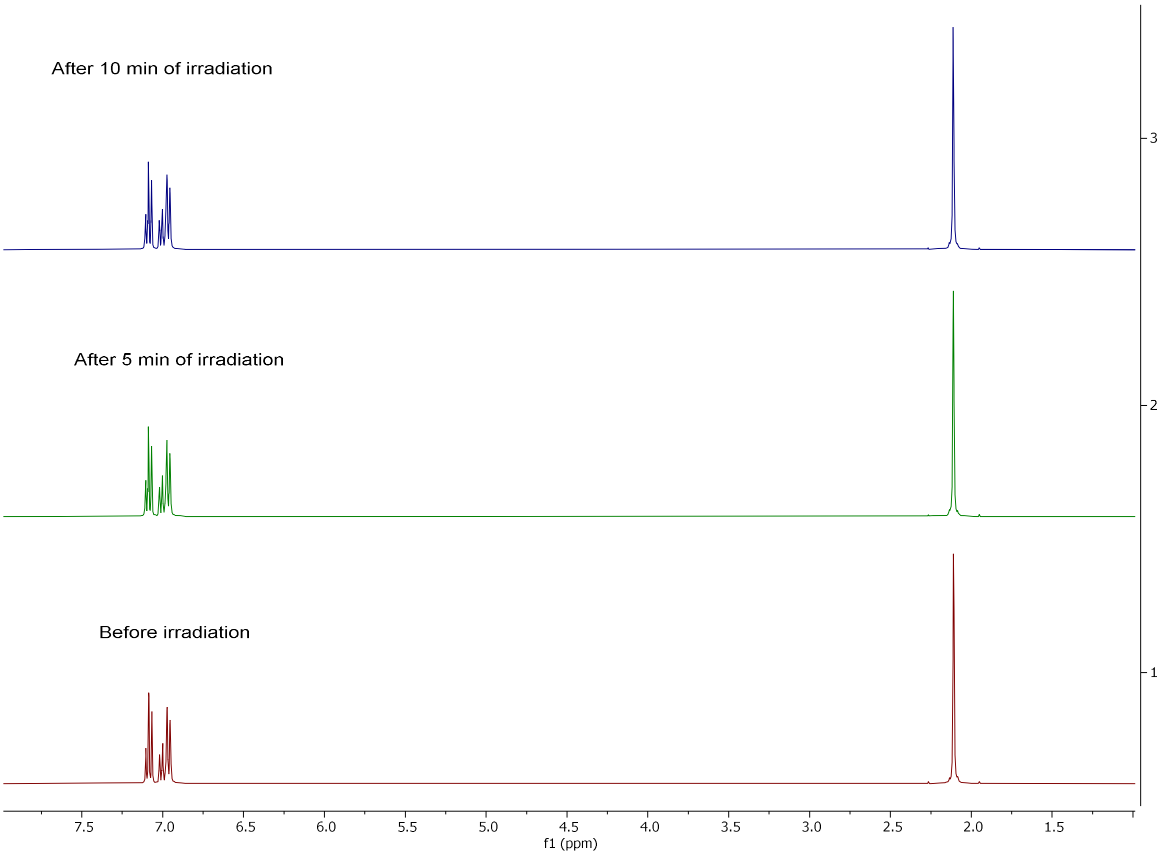

Figure SI-9: ^{1}H NMR spectra of **Mn1** and **dppp (L8)** ligand after different irradiation time.

4.2 Other spectroscopic data

To characterize the formation of paramagnetic species upon irradiation, a series of *in situ* EPR measurements of mixture between **Mn1** (1 mM) and different ligands (1 mM) was performed:

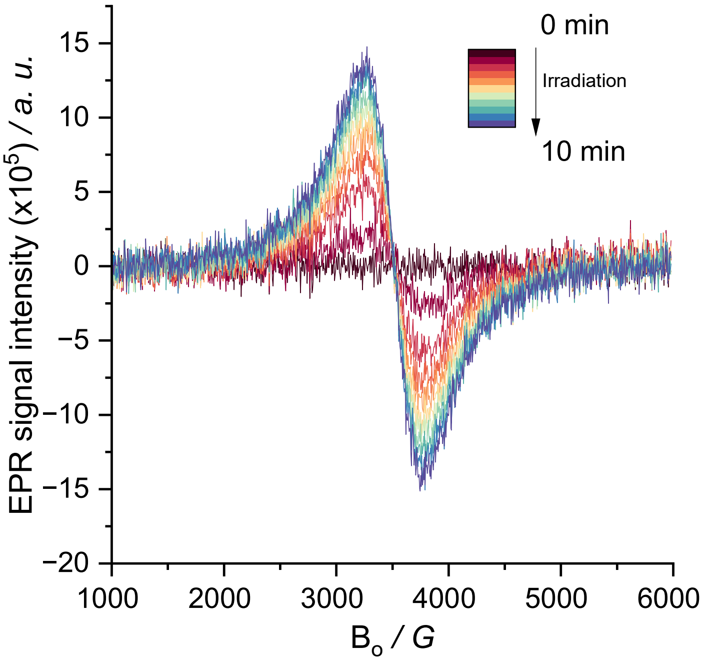

Figure SI-10: *In situ* EPR spectra of **Mn1** during the irradiation.

To monitor the reaction and characterize the CO band of Mn-catalyst, *in situ* IR spectroscopic experiments were conducted. The mixture was irradiated by blue-LEDs strip (10 W, 21 units, λmax = 467 nm, manufacturer: Ledxon) at room temperature. A spectrum of neat toluene recorded before each experiment was used as a background for subtraction.

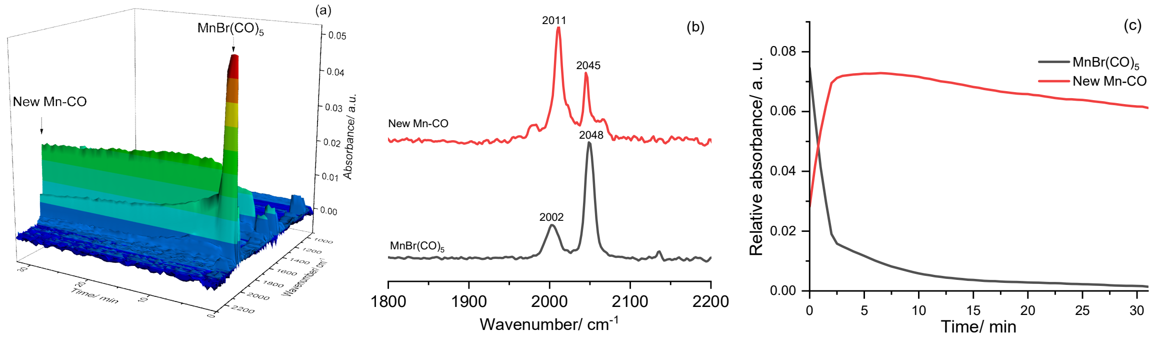

Figure SI-11: IR-spectroscopic data of **Mn1** (5 mM) during the irradiation.

With the help of the chemometric decomposition of the spectral data matrix by PGA (peak group analysis), we were able to determine two different components of Mn (Figure SI-12b). Firstly, **Mn1** is characterized by a stretching mode of carbonyl group observed at 2048, 2002 cm^{-1} (Figure SI-12b, black curve). These two bands were quickly disappearing upon the irradiation and replaced by new bands at 2011 and 2045 cm^{-1}, indicating that the degradation of **Mn1** took place promptly.

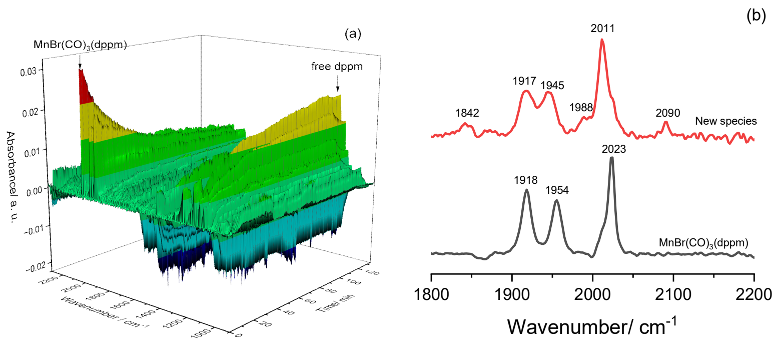

Figure SI-12: IR-spectroscopic data of **Mn7** (5 mM) during the irradiation.

With the addition of dppm ligand, **Mn7** is characterized by three vibration bands in carbonyl stretching region (υ = 1918, 1954 and 2023 cm^{-1}) (Figure SI-13). Upon irradiation, these bands gradually decreased and were replaced by unresolved bands (red spectra), which have not been further identified. Based on the concentration profile of extracted components, the degradation of **Mn7** was slower than **Mn1**, indicating the role of dppm ligand in decelerating the catalyst decomposition. Besides, the free dppm ligand was also observed soon upon the irradiation, which can also confirm the dissociation of the ligand by light.

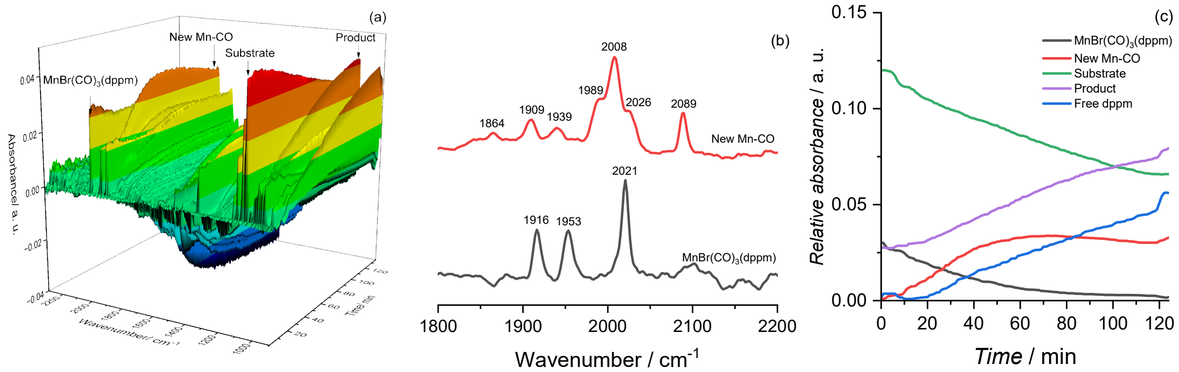

Figure SI-13: IR-spectroscopic data of the reaction mixture between **Mn7** (5 mM) and triyne **17** (50 mM) during the irradiation.

In the presence of triyne **17**, the species resulted from **Mn7** decomposition has addition signal as a broad band in 1970 – 2040 cm^{-1} region. This could be due to the coordination between the manganese atom and the substrate. Interestingly, the rate of ligand dissociation is in correlation with this of cyclization product formation.

5 Computational Calculation Data

All calculations were carried out with Gaussian 16 program.[39] Geometry optimization was performed in gas phase at the M06L level[40] with the TZVP[41] basis set (M06L/TZVP) and all optimized structures were further characterized either as energy minimums without imaginary frequencies or transition states with only one imaginary frequency by frequency analysis. Based on M06L geometries in the gas phase, single-point energies were calculated by including the solvation effect of diethyl ether as solvent based on solute electron density (SMD[42]) at the M06L-SCRF level using the same basis sets (M06L-SCRF/TZVP//M06L/TZV). To rule out the effect of oversimplification, real-size molecules were used in our calculations. For discussion we used the computed Gibbs free energy (ΔG) at the final computed single-point energy level.

Table SI-2: M06L/TZVP computed total electronic energies (HF, au), zero-point vibrational energies (ZPE, au), sum of electronic and thermal enthalpies (Htot, au), sum of electronic and thermal free energies (Gtot, au), number of Imaginary frequencies (NImag) in gas phase, as well as M06L-SCRF/TZVP//M06L/TZVP/SP computed sum of electronic and thermal free energies (Gtot, au) in solution.

	**M06L/TZVP//FOpt**	**M06L-SCRF/TZVP//SP**
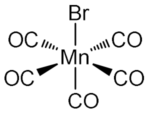	HF=-4291.9678644<br>ZPE=0.043267<br>NImag=0<br>Htot=-4291.909779<br>Gtot= -4291.966398	Gtot= -4291.970109
CO	HF=-113.3383397<br>ZPE=0.005030<br>NImag=0<br>Htot=-113.330005<br>Gtot= -113.352435	Gtot= -113.348045
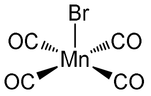	HF=-4178.5491151<br>ZPE= 0.034036<br>NImag=0<br>Htot= -4178.502315<br>Gtot= -4178.554265	Gtot= -4178.561958
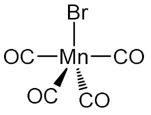	HF=- 4178.5813849<br>ZPE= 0.034705<br>NImag=0<br>Htot= -4178.534172<br>Gtot= -4178.587372	Gtto= -4178.589191
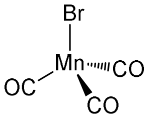<br>(singlet)	HF=-4065.2012958<br>ZPE=0.026496<br>NImag=0<br>Htot=-4065.164726<br>Gtot= -4065.212449	Gtot= -4065.212463
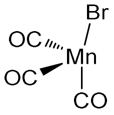<br>(triplet)	HF=-4065.1752031<br>ZPE= 0.024333<br>NImag=0<br>Htot= -4065.139974<br>Gtot= -4065.190555	Gtot= -4065.193519
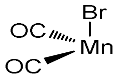<br>(singlet)	HF=-3951.7770467<br>ZPE=0.017337<br>NImag=0<br>Htot=-3951.751749<br>Gtot= -3951.793963	Gtot= -3951.804579

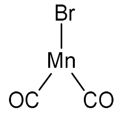<br>(triplet)	HF=-3951.800521<br>ZP=0.016820<br>NImag=0<br>Htot=-3951.775592<br>Gtot= -3951.819326	
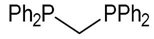	HF=-1648.7703333<br>ZPE=0.393413<br>NImag=0<br>Htot=-1648.351929<br>Gtot= -1648.434986	Gtot= -1648.457008
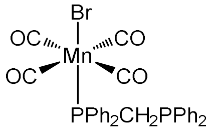	HF=-5827.3977918<br>ZPE=0.430352<br>NImag=0<br>Htot=-5826.929611<br>Gtot= -5827.041869	Gtot=-5827.063067
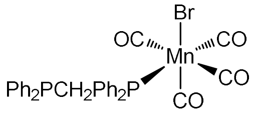	HF=-5827.4071581<br>ZPE=0.430662<br>NImag=0<br>Htot=-5826.938873<br>Gtot=-5827.050109	Gtot= -5827.079150
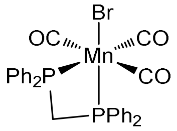	HF=-5714.039768<br>ZPE=0.421804<br>NImag=0<br>Htot=-5713.582607<br>Gtot= -5713.690074	Gtot= -5713.718145<br><br>Less stable by 5.66 kcal/mol
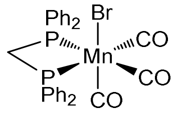	HF=-5714.0533224<br>ZPE=0.422728<br>NImag=0<br>Htot=-5713.595619<br>Gtot= -5713.700069	Gtot= -5713.727171
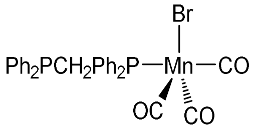	HF=-5714.024710<br>ZPE= 0.421532<br>NImag=0<br>Htot= -5713.567524<br>Gtot= -5713.676092	Gtot= -5713.700601
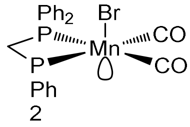<br>(singlet)	HF=-5600.629104<br>ZPE=0.413191<br>NImag=0<br>Htot=-5600.182915<br>Gtot=-5600.283026	Gtot= -5600.305373
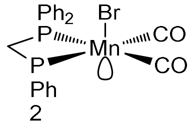<br>(triplet)	HF=-5600.6386697<br>ZPE=0.412107<br>NImag=0<br>Htot=-5600.193006<br>Gtot= -5600.296518	Gtot= -5600.323375
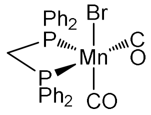	HF=-5600.6522824<br>ZPE=0.413921<br>NImag=0<br>Htot=-5600.205644<br>Gtot= -5600.304645	Gtot= -5600.332521
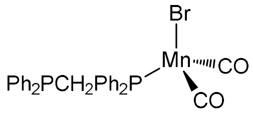	HF=-5600.6348775<br>ZPE= 0.413297<br>NImag=0<br>Htot= -5600.188277<br>Gtot= -5600.291781	Gtot= -5600.319539
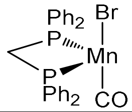	HF=-5487.2495265<br>ZPE=0.404643<br>NImag=0	Gtot= -5486.939555

(singlet)	Htot=-5486.814180<br>Gtot= -5486.910688	
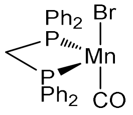<br>(triplet)	HF= -5487.2538391<br>ZPE=0.404038<br>NImag= 0<br>Htot=-5486.818728<br>Gtot= -5486.916344	Gtot= -5486.946815
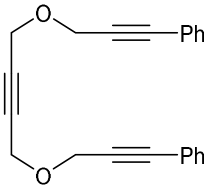<br>Triyne (CI-Symm)	HF=-999.5877702<br>ZPE=0.333893<br>NImag=<br>Htot=-999.230621<br>Gtot= -999.309037	Gtot= -999.327994
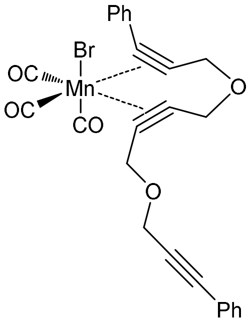	HF=-5064.8153114<br>ZPE=0.362039<br>NImag=0<br>Htot=-5064.419609<br>Gtot= -5064.523771	Gtot= -5064.548552
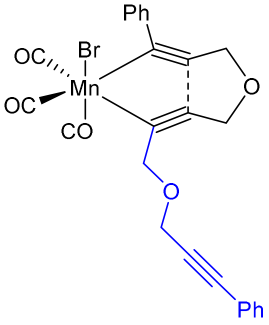	HF=-5064.787939<br>ZPE=0.360650<br>NImag=1 (-206.2498)<br>Htot=-5064.394323<br>Gtot= -5064.498846	Gtot= -5064.523546
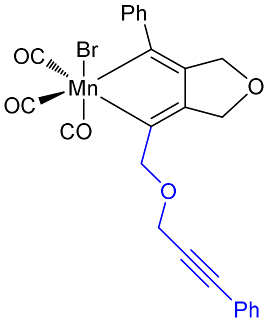	HF=-5064.8318881<br>ZPE=0.362704<br>NImag=0<br>Htot=-5064.437301<br>Gtot= -5064.533959	Gtot= -5064.555749
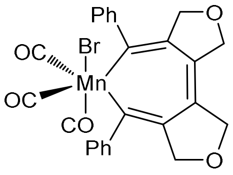	HF=-5064.8670947<br>ZPE=0.363632<br>NImag=0<br>Htot=-5064.473004<br>Gtot= -5064.566072	Gtot= -5064.590850

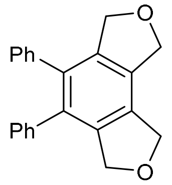	HF=-999.7806601<br>ZPE=0.340264<br>NImag=0<br>Htot=-999.420383<br>Gtot= -999.488371	Gtot= -999.510037
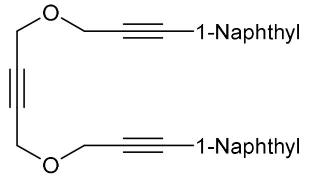	HF=-1306.9309009<br>ZPE=0.427998<br>NImag=0<br>Htot=-1306.474582<br>Gtot= -1306.564239	Gtot= -1306.590174
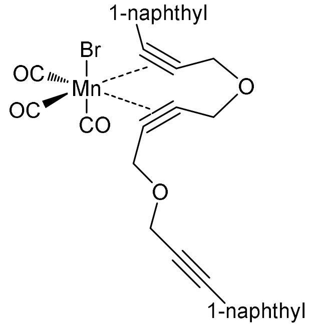	HF=-5372.1541543<br>ZPE=0.455192<br>NImag=0<br>Htot=-5371.659937<br>Gtot= -5371.775960	Gtot= -5371.807021
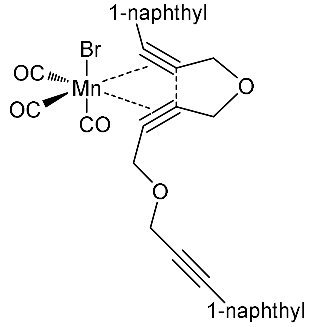	HF=-5372.1257919<br>ZPE=0.454476<br>NImag= (1, -205)<br>Htot=-5371.633176<br>Gtot= -5371.749546	Gtot= -5371.781465
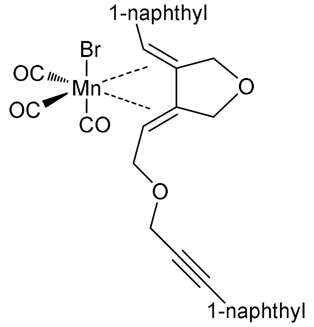	HF=-5372.1704789<br>ZPE=0.456228<br>NImag=0<br>Htot=-5371.677038<br>Gtot= -5371.785578	Gtot= -5371.818565
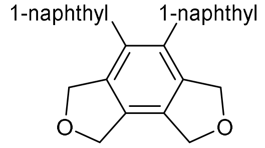	HF=-1307.1177602<br>ZPE=0.434132<br>NImag=0<br>Htot=-1306.658285<br>Gtot= -1306.738206	Gtot= -1306.766521

Table SI-3: M06L/TZVP optimized Cartesian Coordinates in gas phase

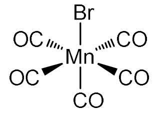	Mn,0,0.,0.,1.402991951<br>C,0,1.879372233,0.0003133863,1.248448979<br>O,0,3.0109830048,0.000502083,1.1254895328<br>C,0,0.0003133863,-1.879372233,1.248448979<br>O,0,0.000502083,-3.0109830048,1.1254895328<br>C,0,-1.879372233,-0.0003133863,1.248448979<br>O,0,-3.0109830048,-0.000502083,1.1254895328<br>C,0,-0.0003133863,1.879372233,1.248448979<br>O,0,-0.000502083,3.0109830048,1.1254895328<br>C,0,0.,0.,3.2238000404<br>O,0,0.,0.,4.3707737239<br>Br,0,0.,0.,-1.1472173928
CO	O,0,0.,0.,1.1649355744<br>C,0,0.,0.,0.0350644256
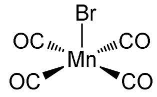	Mn,0,0.,0.,1.3853789504<br>C,0,1.8757549356,0.0003127314,1.261525955<br>O,0,3.0070851811,0.0005013501,1.1153371116<br>C,0,0.0003127314,-1.8757549356,1.261525955<br>O,0,0.0005013501,-3.0070851811,1.1153371116<br>C,0,-1.8757549356,-0.0003127314,1.261525955<br>O,0,-3.0070851811,-0.0005013501,1.1153371116<br>C,0,-0.0003127314,1.8757549356,1.261525955<br>O,0,-0.0005013501,3.0070851811,1.1153371116<br>Br,0,0.,0.,-1.1417004483
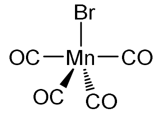	Mn,0,-0.2686835656,-0.0000447788,1.195879843<br>C,0,-0.2056055647,-1.8832390922,1.114546237<br>O,0,-0.1692342262,-3.0208799071,1.0739630264<br>C,0,-2.0540181824,-0.0003423586,1.4680696605<br>O,0,-3.1938725453,-0.0005323498,1.6047459228<br>C,0,-0.2062333523,1.8831704572,1.1145462243<br>O,0,-0.1702412613,3.0208233334,1.0739630061<br>C,0,0.0261666012,0.000004373,2.9783884503<br>O,0,0.2481611995,0.000041379,4.1046672256<br>Br,0,1.1002727273,0.0001833932,-0.7621909491
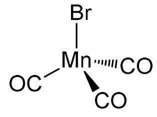<br>(singlet)	Mn,0,0.2724146599,-0.2723238707,1.0348555399<br>C,0,2.0455408809,-0.2412919517,1.3024912693<br>O,0,3.1843400388,-0.2036474509,1.4506848327<br>C,0,0.2419737796,-2.0454603375,1.3024912663<br>O,0,0.2047088775,-3.1842719805,1.4506848277<br>C,0,-0.0170913239,0.0170856251,2.7811995102<br>O,0,-0.222616151,0.222541954,3.8929474336<br>Br,0,-0.7976955095,0.7974296604,-0.6459421192
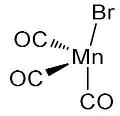<br>(triplet)	Mn,0,0.3968224569,-0.3966902058,1.1258849159<br>C,0,2.1493961807,0.3657696208,1.3061066125<br>O,0,3.2092904508,0.7690745932,1.4266449382<br>C,0,-0.3650531409,-2.1495179837,1.3061066083<br>O,0,-0.7680047958,-3.2095466291,1.4266449316<br>C,0,0.278533777,-0.2784409515,2.9451170455<br>O,0,0.1746239501,-0.1745657571,4.0889634724<br>Br,0,-0.1300787941,0.1300354458,-1.0927442192
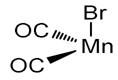<br>(singlet)	Mn,0,0.4773474014,-0.2537068069,1.0446022647<br>C,0,0.2855013242,-2.0022906137,1.2862141489<br>O,0,0.0956376613,-3.13088438,1.4313374887<br>C,0,0.0308485981,0.0262424506,2.7401858248<br>O,0,-0.3232523646,0.2064789246,3.8232192199<br>Br,0,-0.8843876204,0.6891624254,-0.509321947

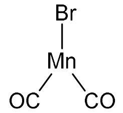<br>(triplet)	Mn,0,-0.1971800018,-0.2635038439,0.940403666<br>C,0,0.1566273526,-1.9903766421,1.4075296805<br>O,0,0.3808446136,-3.0749639481,1.7269377027<br>C,0,-0.0788336632,-0.1127667355,2.7540234586<br>O,0,0.000221823,-0.04063029,3.901700678<br>Br,0,-0.5799851242,1.0172434595,-0.9143581856
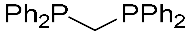<br>dppm	H,0,-0.2647854254,-0.476753102,-0.4622050332<br>C,0,0.1573598839,-0.1710321364,0.4982494926<br>H,0,1.2437053875,-0.1416085341,0.3834317562<br>P,0,-0.2526958494,-1.4545231112,1.7983618573<br>P,0,-0.5033095831,1.5424077007,0.8582390446<br>C,0,0.8059407702,2.5461224781,0.0271593532<br>C,0,2.6384991067,4.1675311272,-1.3369582809<br>C,0,1.2710642034,3.7335243068,0.5948655137<br>C,0,1.2659134258,2.194929058,-1.2439953165<br>C,0,2.1770098066,2.9947957676,-1.9165522254<br>C,0,2.1795934579,4.5339522098,-0.0799956894<br>H,0,0.9287485898,4.0272536085,1.5808511424<br>H,0,0.9123297149,1.2845768191,-1.7157957295<br>H,0,2.5276717754,2.6993900537,-2.8981104679<br>H,0,2.5353103894,5.4467466437,0.3825453912<br>H,0,3.3508721804,4.7917771222,-1.8615435601<br>C,0,-0.0231792006,1.7796435217,2.6253834727<br>C,0,0.5763236553,2.1789615149,5.321870519<br>C,0,1.1932018332,1.3453882665,3.1533312994<br>C,0,-0.9292290944,2.4226761082,3.4663209426<br>C,0,-0.63094643,2.6260025796,4.8068164833<br>C,0,1.4884618802,1.5375690839,4.4932645569<br>H,0,1.9136199906,0.8500493286,2.5118586236<br>H,0,-1.8828147462,2.7491491569,3.0653940564<br>H,0,-1.3447361891,3.1270349557,5.4493171011<br>H,0,2.4317667626,1.1867679946,4.8934714298<br>H,0,0.8087042071,2.3288760484,6.3691001703<br>C,0,-0.4144123625,-2.9483516869,0.7301142257<br>C,0,-0.5249513654,-5.3309015953,-0.7399307016<br>C,0,0.4336584472,-3.1581551663,-0.3585218194<br>C,0,-1.308049941,-3.9642070886,1.0784144907<br>C,0,-1.3661715979,-5.1396948363,0.347649847<br>C,0,0.376340422,-4.3366685088,-1.0882051317<br>H,0,1.1480597932,-2.3944529064,-0.644882575<br>H,0,-1.9744634241,-3.8251873014,1.9225866525<br>H,0,-2.0745438294,-5.9099794997,0.6277087978<br>H,0,1.0390768021,-4.4761525018,-1.9337562698<br>H,0,-0.5718193491,-6.2495276493,-1.3111121697<br>C,0,-2.0293085129,-1.061800803,2.1183927264<br>C,0,-4.661137698,-0.3426689867,2.7000417209<br>C,0,-2.3885217153,-0.6689407115,3.4056389008<br>C,0,-3.0103525497,-1.0993878125,1.1256561839<br>C,0,-4.3164322,-0.742332922,1.4142274845<br>C,0,-3.6975053213,-0.3076115112,3.6954044312<br>H,0,-1.6288951298,-0.6307021157,4.1795040475<br>H,0,-2.7454302375,-1.4104596034,0.1206955828<br>H,0,-5.0684317222,-0.7694294525,0.6351036888<br>H,0,-3.9619684095,0.0024375891,4.6991624985<br>H,0,-5.6829287363,-0.0609999552,2.9232140522
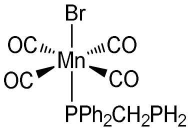	Mn,0,-0.1282374418,-0.0076868862,1.1831617601<br>C,0,-1.9396950644,0.1443006947,1.5673358834<br>O,0,-3.0584952138,0.258484742,1.7889143487<br>C,0,-0.5100807516,0.0224432033,-0.6366347935<br>O,0,-0.7361042142,0.0554749806,-1.7595748007<br>C,0,1.6997352408,0.0684106529,0.7704635514

	O,0,2.8106712873,0.1432882946,0.515243078<br>C,0,0.3207978892,0.1740427851,2.9897949023<br>O,0,0.6178277599,0.3177054918,4.0847897934<br>Br,0,-0.085303477,2.5533595244,1.0778591008<br>P,0,-0.1717285649,-2.3012059738,1.2260157029<br>C,0,-0.515833085,-3.1456376845,2.8213641326<br>C,0,-1.2178875535,-4.4463134751,5.1881508213<br>C,0,-0.080067918,-4.4479522315,3.0720421609<br>C,0,-1.3173860614,-2.5119073752,3.7682541595<br>C,0,-1.6695037005,-3.1591235983,4.94308606<br>C,0,-0.4229104424,-5.0895618657,4.2504970861<br>H,0,0.5324820839,-4.9602581697,2.3404080429<br>H,0,-1.6720708624,-1.504041374,3.59145832<br>H,0,-2.292510369,-2.6518087372,5.6684404377<br>H,0,-0.0734336496,-6.0979517619,4.4332123314<br>H,0,-1.4863800989,-4.9495160656,6.1084706815<br>C,0,1.4096832809,-3.0018502254,0.6236852569<br>C,0,3.928852811,-3.7156383471,-0.3384872679<br>C,0,1.6602587737,-3.0390377697,-0.7482617192<br>C,0,2.448150411,-3.2914473613,1.5079950507<br>C,0,3.6973121187,-3.6545069566,1.0272625701<br>C,0,2.9081570225,-3.4026853221,-1.2251165899<br>H,0,0.8767461001,-2.7904860889,-1.4549213939<br>H,0,2.283370772,-3.2275229599,2.5775219403<br>H,0,4.4939020243,-3.8807155927,1.724840307<br>H,0,3.0809556398,-3.4418294854,-2.2932017592<br>H,0,4.9053580512,-3.9968062423,-0.7118725572<br>C,0,-1.4897958518,-2.9997944223,0.1220373716<br>H,0,-1.3090719684,-2.6468950719,-0.8972138438<br>H,0,-2.4009527477,-2.5013769871,0.4650663891<br>P,0,-1.8503338326,-4.8350851829,0.159202929<br>C,0,-3.2642601888,-4.9003967267,-1.0264374349<br>C,0,-5.4447389524,-5.2264779518,-2.7525269397<br>C,0,-3.9705962832,-3.7957360649,-1.4980503937<br>C,0,-3.6774468825,-6.1743235062,-1.4291752262<br>C,0,-4.7498876551,-6.3359838369,-2.2881430047<br>C,0,-5.0534164092,-3.9595951989,-2.3529743077<br>H,0,-3.683323804,-2.7915478262,-1.2125840325<br>H,0,-3.1441631282,-7.0477818727,-1.0683502223<br>H,0,-5.0485726369,-7.3314560442,-2.5927641592<br>H,0,-5.587590303,-3.087689206,-2.7099529444<br>H,0,-6.2875674315,-5.3513761001,-3.4204436438<br>C,0,-0.5057243893,-5.4732857889,-0.9403840233<br>C,0,1.5991568283,-6.4366150907,-2.500934531<br>C,0,-0.4464355257,-5.1880197624,-2.3053525329<br>C,0,0.4937689259,-6.2636480513,-0.3761677988<br>C,0,1.5446229519,-6.7374971149,-1.1488689945<br>C,0,0.5985865714,-5.665391187,-3.0795883487<br>H,0,-1.2285045412,-4.5913742153,-2.7639586412<br>H,0,0.4438178649,-6.5138349264,0.6781056507<br>H,0,2.318296047,-7.3441782588,-0.6946409629<br>H,0,0.6329537161,-5.4379288105,-4.138228391<br>H,0,2.416027989,-6.808190989,-3.1072916906
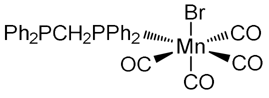	Mn,0,0.1447583622,0.2173584192,1.0853273895<br>C,0,0.2473916073,-0.7599747087,-0.4210205427<br>O,0,0.3137505092,-1.4037765398,-1.3787768443<br>C,0,1.9766679834,0.2714285549,1.2883402978<br>O,0,3.1147081336,0.2972842858,1.4042852818<br>C,0,0.1410339022,1.9139010447,0.2839170578<br>O,0,0.1407428595,2.9693695352,-0.1542569264<br>C,0,0.0062498189,-1.2441949478,2.2340205345

	O,0,-0.0902529203,-2.1183695487,2.9672620577<br>P,0,-2.2421289905,0.1606790414,0.9238129556<br>Br,0,0.0812269181,1.5935815289,3.2494889948<br>C,0,-3.1048530385,0.7304934294,2.424177623<br>C,0,-4.1517679325,1.6482374273,4.8359268791<br>C,0,-3.2472250137,-0.1352762334,3.5066501294<br>C,0,-3.4476080196,2.0730943825,2.5781536596<br>C,0,-3.9801119653,2.524500502,3.7745794859<br>C,0,-3.7758727503,0.3199877717,4.7025962672<br>H,0,-2.945882673,-1.1724458686,3.4249264731<br>H,0,-3.2855692527,2.7708976823,1.7647694725<br>H,0,-4.2460746082,3.5684248111,3.8818082651<br>H,0,-3.88978337,-0.3670340923,5.5314866734<br>H,0,-4.5623817686,2.004604929,5.7722918216<br>C,0,-3.006063179,1.0680823094,-0.4786118672<br>C,0,-4.1842514393,2.2656981695,-2.7074327781<br>C,0,-4.3279974967,1.5152945437,-0.4284163242<br>C,0,-2.2927188892,1.2139915944,-1.666665973<br>C,0,-2.8782852658,1.8069202844,-2.7749676361<br>C,0,-4.9072104825,2.1181425364,-1.5328094087<br>H,0,-4.9061371475,1.3893942584,0.4785073986<br>H,0,-1.2695385286,0.865738449,-1.7321894738<br>H,0,-2.3083097575,1.9145010998,-3.6889075707<br>H,0,-5.9312862687,2.4650721117,-1.4768388969<br>H,0,-4.6398968441,2.7357387194,-3.5698865323<br>C,0,-2.8236599985,-1.5736624437,0.6246077281<br>H,0,-2.5367185131,-2.1955690797,1.4759751312<br>H,0,-2.2253554141,-1.9199591242,-0.2199049562<br>P,0,-4.6247318047,-1.8395008327,0.1651612744<br>C,0,-4.5003111478,-3.5827076526,-0.4290109731<br>C,0,-4.4482949656,-6.1497306812,-1.5511872546<br>C,0,-3.5550874698,-3.9226084264,-1.4003265051<br>C,0,-5.4367946529,-4.5472821579,-0.0527161977<br>C,0,-5.4081355308,-5.8180589497,-0.6071483875<br>C,0,-3.5238186927,-5.1947347257,-1.9484200533<br>H,0,-2.8354241121,-3.1880247384,-1.7466648321<br>H,0,-6.1929902892,-4.3051392202,0.6845142332<br>H,0,-6.140180112,-6.5530188118,-0.2952792495<br>H,0,-2.7750980593,-5.438166966,-2.6920802057<br>H,0,-4.4240492629,-7.1436197507,-1.9796866749<br>C,0,-5.3583199731,-2.0521525557,1.8440060249<br>C,0,-6.4626308908,-2.1918522313,4.4034081656<br>C,0,-4.9306269069,-3.0256356066,2.749417819<br>C,0,-6.3524404031,-1.1594480083,2.238138503<br>C,0,-6.9000194734,-1.2251262334,3.5121710796<br>C,0,-5.4786939667,-3.0955183852,4.0193313645<br>H,0,-4.164807638,-3.7360881994,2.4545417622<br>H,0,-6.6930713425,-0.4020125759,1.5403917564<br>H,0,-7.6654884122,-0.5186938676,3.8081628919<br>H,0,-5.1382695106,-3.8545377365,4.7131755236<br>H,0,-6.8874517607,-2.2451188313,5.3982309325
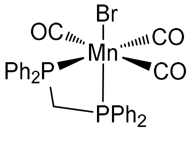	Mn,0,0.0003419177,-0.0406557989,0.1415716936<br>Br,0,2.5245346015,-0.4086267469,-0.1876931521<br>C,0,-0.4610494968,-0.2702513871,-1.6000750296<br>O,0,-0.7810860557,-0.4196290698,-2.6987794751<br>C,0,-0.0054953424,-1.8867411674,0.4056755598<br>O,0,0.0336967904,-3.0216163391,0.5630540841<br>C,0,0.3094896072,1.763078379,-0.1356594443<br>O,0,0.5045866755,2.8805173406,-0.3208156831<br>P,0,0.249809077,0.2541543192,2.4476164933<br>P,0,-2.0408844904,0.3847804083,0.9936598121

	C,0,-1.4637053112,0.9287371485,2.673886205<br>H,0,-1.3907047177,2.0175350509,2.6839869127<br>H,0,-2.0225610107,0.5988683246,3.5506942114<br>C,0,-3.2579554039,-0.9578246323,1.306004958<br>C,0,-5.0739808973,-3.0276278329,1.727315095<br>C,0,-3.2937762817,-2.0500114753,0.443781149<br>C,0,-4.1527579658,-0.9049651335,2.3748321579<br>C,0,-5.051882104,-1.9374485521,2.587196922<br>C,0,-4.200486387,-3.0789271654,0.6519568661<br>H,0,-2.6054132749,-2.095331372,-0.3926552978<br>H,0,-4.149031043,-0.054661758,3.0483894489<br>H,0,-5.7371193064,-1.8914874611,3.4245103394<br>H,0,-4.2168723601,-3.9249958098,-0.0231535565<br>H,0,-5.7749299603,-3.8354329804,1.8958346899<br>C,0,-3.1096142707,1.7482471664,0.3820746477<br>C,0,-4.7427753645,3.7738433349,-0.6083045726<br>C,0,-3.8557667756,2.5404974446,1.2530931341<br>C,0,-3.1867800561,1.9824691408,-0.9887372833<br>C,0,-4.003842465,2.9888525617,-1.4807052924<br>C,0,-4.667010312,3.5498428992,0.7591604969<br>H,0,-3.8015518137,2.3749248763,2.3237751731<br>H,0,-2.6021998353,1.3787349144,-1.6732966582<br>H,0,-4.0553998685,3.1646125242,-2.5477042856<br>H,0,-5.2384946292,4.1647602183,1.4432425251<br>H,0,-5.3744822501,4.5646089167,-0.9928408981<br>C,0,0.3504618544,-1.248990257,3.4786250409<br>C,0,0.6037018356,-3.6779014988,4.8156699944<br>C,0,1.6147616052,-1.7619770947,3.7722871866<br>C,0,-0.7828812387,-1.971214839,3.8453532012<br>C,0,-0.6544982295,-3.1784416485,4.516037139<br>C,0,1.736626327,-2.9683610878,4.4414107599<br>H,0,2.5016771662,-1.2181750944,3.4665659575<br>H,0,-1.7735356613,-1.6072319425,3.5971197297<br>H,0,-1.5423120419,-3.7306901267,4.7985986723<br>H,0,2.7211514183,-3.3595094508,4.664702291<br>H,0,0.7026063118,-4.6227190687,5.3350345656<br>C,0,1.3769227337,1.4296772749,3.2805312501<br>C,0,3.0462478623,3.2541089271,4.544770811<br>C,0,1.1980604306,1.7234143078,4.6340635949<br>C,0,2.3984170303,2.048391684,2.5663920001<br>C,0,3.231538753,2.9583178739,3.203752264<br>C,0,2.0290153453,2.6352933372,5.2615597439<br>H,0,0.4128745707,1.2297282445,5.1973344955<br>H,0,2.5550033862,1.7923408576,1.5241232236<br>H,0,4.0291798973,3.4332779865,2.6467526227<br>H,0,1.8868682423,2.8620580018,6.3109125732<br>H,0,3.6978511589,3.9648536983,5.0379187993
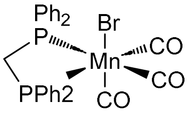	Mn,0,-0.3287643878,-0.3518847784,1.5477578331<br>C,0,-2.1579506201,-0.4111632482,1.6495412185<br>O,0,-3.3048138953,-0.4176025756,1.7191278513<br>C,0,-0.1080111433,-2.1566711994,1.3708143492<br>O,0,0.0422753739,-3.2918766191,1.2627432679<br>C,0,-0.1020327096,-0.455831063,3.3212662555<br>O,0,0.0652730013,-0.5681910853,4.4612644233<br>P,0,-0.2080037915,2.000303968,1.3463575429<br>P,0,1.9018743392,0.2800834399,1.2132599946<br>C,0,1.4906195218,1.9812713007,0.6089287017<br>H,0,2.1669743399,2.7986687606,0.8609260902<br>H,0,1.3937937396,1.8928466749,-0.4725890911<br>C,0,-1.3028063883,3.0070380807,0.2776575821<br>C,0,-3.0916780059,4.4765799272,-1.2678630931

	C,0,-2.4768520309,3.5077327244,0.8412700321<br>C,0,-1.0392729244,3.2381084676,-1.0692678446<br>C,0,-1.9290627846,3.9773036889,-1.8343145338<br>C,0,-3.3665838412,4.2376701375,0.0712278711<br>H,0,-2.6931046429,3.3298575843,1.8894849346<br>H,0,-0.1510032909,2.8279361688,-1.5312168511<br>H,0,-1.7147983176,4.1536415931,-2.880812822<br>H,0,-4.2754183517,4.6204288542,0.5181369987<br>H,0,-3.7863675883,5.0482548308,-1.8703396682<br>C,0,-0.0498862792,3.0922357535,2.8160354677<br>C,0,0.2087126195,4.7508548732,5.0389590586<br>C,0,-0.5837110781,2.7122063673,4.042632207<br>C,0,0.5916826785,4.3275589759,2.7054439516<br>C,0,0.7272476745,5.1480060602,3.8120979637<br>C,0,-0.4538174405,3.5391829402,5.1505506421<br>H,0,-1.0995505776,1.7650259826,4.1354087849<br>H,0,0.9838760773,4.6497480574,1.7462878822<br>H,0,1.2319650588,6.1015768358,3.718084412<br>H,0,-0.8679173264,3.230036841,6.1018051709<br>H,0,0.3168907476,5.391938247,5.9048893295<br>C,0,2.8995256905,0.496645009,2.7338761153<br>C,0,4.2795079314,0.6323389854,5.1535623367<br>C,0,3.4006193567,-0.6623175618,3.3313520384<br>C,0,3.0971577897,1.7210282829,3.3644518938<br>C,0,3.7828295259,1.7864131372,4.5702487059<br>C,0,4.0897737962,-0.5936530718,4.5287162863<br>H,0,3.2431493683,-1.6249256502,2.8546779473<br>H,0,2.71029498,2.6339416035,2.9299063112<br>H,0,3.9257565738,2.7462267444,5.0513185462<br>H,0,4.4728347308,-1.4996933447,4.9809896255<br>H,0,4.8117078428,0.6847972206,6.0949403878<br>C,0,3.1180347846,-0.4360105314,0.0461603581<br>C,0,4.9962778446,-1.5114123393,-1.6958461645<br>C,0,4.3771680276,0.1525437605,-0.0796593324<br>C,0,2.8070165797,-1.567415112,-0.7014381517<br>C,0,3.7491191936,-2.1031049271,-1.5684599882<br>C,0,5.3102214682,-0.3810775495,-0.9522340111<br>H,0,4.6297149838,1.023856967,0.5156511053<br>H,0,1.8232177135,-2.0120182754,-0.6196465965<br>H,0,3.5027286094,-2.9835814163,-2.1481482694<br>H,0,6.2851653645,0.0803957447,-1.0481888362<br>H,0,5.7277733285,-1.9312462051,-2.3751780808<br>Br,0,-0.5190478302,-0.2349813639,-1.027196756
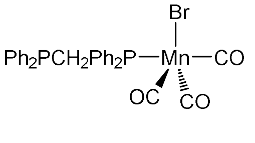	Mn,0,-0.6502243433,-0.1329709798,0.7974387744<br>Br,0,1.7456894836,-0.4066450306,0.8937917217<br>P,0,-0.2656734447,2.1325083687,0.2542886085<br>C,0,-2.0607563933,0.3841948324,1.7721526012<br>O,0,-2.9493777325,0.7420291518,2.4180504098<br>C,0,-1.6949427094,-0.3304088261,-0.6315422752<br>O,0,-2.3372230686,-0.4468343739,-1.5852424372<br>C,0,-0.8305058082,-1.8993853026,1.2951514147<br>O,0,-0.974060245,-2.9958179083,1.5937092443<br>C,0,-1.7878345299,3.0187375597,-0.3072359713<br>P,0,-1.6268217124,4.8822128883,-0.5468881984<br>C,0,-2.0993261852,5.0223866943,-2.3218834358<br>C,0,-1.5172619014,4.1789344066,-3.2707645238<br>C,0,-1.7688957732,4.3527532259,-4.6226944705<br>C,0,-2.5987158935,5.3775816266,-5.0531556501<br>C,0,-3.1707123853,6.2307730563,-4.1206497749<br>C,0,-2.9200893057,6.0598142406,-2.7678047969<br>C,0,-3.1748267433,5.425859959,0.2990971856

	C,0,-3.0665731164,6.3444193223,1.3414420411<br>C,0,-4.1945210251,6.7632808371,2.0351083621<br>C,0,-5.4422307438,6.2675352751,1.6915361998<br>H,0,-2.100927327,2.5395082306,-1.2372824138<br>H,0,-2.5772593556,2.820894237,0.4197469561<br>C,0,0.9123533772,2.4933595362,-1.0975160386<br>C,0,2.4551072522,3.0787869081,-3.3387314787<br>C,0,1.0825852751,1.5424644364,-2.1031681246<br>C,0,1.5353778799,3.7362005092,-1.2158434918<br>C,0,2.303347855,4.023693231,-2.3344148269<br>C,0,1.8468783247,1.836896824,-3.2212436998<br>H,0,0.6260261441,0.5645513439,-2.0003054604<br>H,0,1.4107371173,4.4799878872,-0.4383843875<br>H,0,2.7811185656,4.9913789429,-2.4212767082<br>H,0,1.9755521864,1.0914760792,-3.9957215891<br>H,0,3.0544879974,3.3076303395,-4.211083995<br>C,0,0.3067178136,3.0965566181,1.695015494<br>C,0,1.1659529629,4.4440777694,3.9763101673<br>C,0,1.660822403,3.1211945287,2.0295694026<br>C,0,-0.613632176,3.7376311163,2.5248897031<br>C,0,-0.1832668274,4.4115890479,3.6571084135<br>C,0,2.0849258172,3.7966123338,3.1623588461<br>H,0,2.3790192154,2.6071310834,1.4018787775<br>H,0,-1.672768959,3.7195669877,2.2926098462<br>H,0,-0.9072239609,4.9118789835,4.2884584711<br>H,0,3.1384867856,3.8144372011,3.4112194292<br>H,0,1.5013902505,4.971047676,4.8607351998<br>H,0,-3.3759368669,6.7328626817,-2.0506918341<br>H,0,-0.8534127864,3.3803190286,-2.9569775429<br>H,0,-1.312077583,3.6822468008,-5.3406950539<br>H,0,-3.8196550743,7.0345391328,-4.4465497118<br>H,0,-2.7973912426,5.5116798573,-6.1089690766<br>C,0,-4.438116264,4.9348393932,-0.0386416238<br>C,0,-5.5630574424,5.3527053896,0.6525605708<br>H,0,-2.0882205624,6.7284624172,1.6095860534<br>H,0,-4.5390437077,4.2270524508,-0.8546357638<br>H,0,-6.5374504966,4.9648303067,0.3820389824<br>H,0,-4.0971259478,7.478048273,2.8429324<br>H,0,-6.3228951671,6.5925958933,2.2314246795
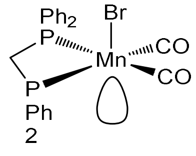<br>(singlet)	Mn,0,-0.3848977882,-0.37016284,1.3556716672<br>C,0,-2.1894009254,-0.549662479,1.4482147925<br>O,0,-3.3375775715,-0.6600292816,1.4750108597<br>C,0,-0.0732320325,-2.1479588315,1.4530454488<br>O,0,0.1372014361,-3.2845471361,1.4958680952<br>P,0,-0.2975524499,1.9817721148,1.1527492043<br>P,0,1.8395446862,0.3355767959,1.1064231342<br>C,0,1.3836141338,1.9744684276,0.3727410854<br>H,0,2.0273697688,2.8395767408,0.5352458971<br>H,0,1.2536109486,1.7910153184,-0.6944089095<br>C,0,-1.3611490407,3.1219001595,0.2011489523<br>C,0,-3.0871079636,4.7539351412,-1.250709111<br>C,0,-2.3114062817,3.9039533428,0.8586105699<br>C,0,-1.295031543,3.1505784565,-1.1915415776<br>C,0,-2.1532882976,3.9683152423,-1.9098301994<br>C,0,-3.1673021399,4.7176403057,0.1339318008<br>H,0,-2.3789983888,3.8808002546,1.9401867732<br>H,0,-0.5922338837,2.513291396,-1.7139379376<br>H,0,-2.0949498762,3.9846396139,-2.9908118276<br>H,0,-3.8991505619,5.3236423289,0.6532733686<br>H,0,-3.7566661345,5.390214044,-1.8158041024<br>C,0,-0.002088207,2.8484078693,2.7419317696

	C,0,0.6294826034,4.0176653296,5.1894850467<br>C,0,-0.1321780845,2.1260740957,3.9257360099<br>C,0,0.4364744519,4.1716645994,2.7980308758<br>C,0,0.7494496244,4.7523724459,4.0160181097<br>C,0,0.1859532235,2.7048917341,5.1454235187<br>H,0,-0.4785302648,1.0961001349,3.8902618063<br>H,0,0.5311341506,4.7464767579,1.8826891275<br>H,0,1.0894292113,5.7800563109,4.0524820676<br>H,0,0.090950699,2.1300586514,6.0579322163<br>H,0,0.8802060965,4.4724355484,6.139756654<br>C,0,2.7145849747,0.7187015818,2.6754908344<br>C,0,3.9393923095,1.1449437298,5.1463126367<br>C,0,2.7963500015,-0.3110250346,3.6155653987<br>C,0,3.2553547027,1.9631208842,2.9907301491<br>C,0,3.8607048457,2.1748345032,4.220577189<br>C,0,3.4101847597,-0.1009653375,4.8395812476<br>H,0,2.374956742,-1.2839054322,3.3822276532<br>H,0,3.1935779103,2.7823872856,2.2845191945<br>H,0,4.266578558,3.1509442687,4.4566648482<br>H,0,3.4688901242,-0.9091169122,5.5578817763<br>H,0,4.4102384744,1.3134616772,6.1067119929<br>C,0,3.1490880281,-0.4080909865,0.0660052619<br>C,0,5.1287334772,-1.554935892,-1.5078945441<br>C,0,4.3970380032,0.2044037566,-0.0465944069<br>C,0,2.8982124696,-1.5988060277,-0.6099083481<br>C,0,3.8911430632,-2.1692323269,-1.3934797412<br>C,0,5.3818762318,-0.3663813073,-0.8352170999<br>H,0,4.6014330752,1.1241143232,0.4915217782<br>H,0,1.9218259917,-2.0616781334,-0.5366016585<br>H,0,3.6922219921,-3.0947003996,-1.9187769478<br>H,0,6.3492842281,0.1126051699,-0.9216790424<br>H,0,5.9005171375,-2.0023117459,-2.1219848179<br>Br,0,-0.4923121851,-0.4285877196,-1.1915419887
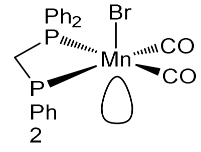<br>(triplet)	Mn,0,-0.4320393219,-0.4692865385,1.18408108<br>C,0,-1.6695697511,-0.8785002053,2.5179206436<br>O,0,-2.3941045048,-1.211002444,3.352594637<br>C,0,-0.0490911977,-2.2408466221,1.2268037652<br>O,0,0.212293984,-3.3680623576,1.2758389323<br>P,0,-0.3313677633,1.9517199035,1.1104755404<br>P,0,1.8482819583,0.2793851565,1.0477753252<br>C,0,1.3475407923,1.9101980637,0.3178782197<br>H,0,1.9942225838,2.7770367249,0.4620604913<br>H,0,1.2018103422,1.7203797089,-0.7467774327<br>C,0,-1.3614194554,3.1216951096,0.1559271248<br>C,0,-3.0447610551,4.8114199027,-1.2818115093<br>C,0,-2.331981613,3.8782420321,0.8143713086<br>C,0,-1.253359571,3.2070641439,-1.2312274085<br>C,0,-2.0896522184,4.0535950136,-1.9424084221<br>C,0,-3.1663356703,4.7201024461,0.0974891667<br>H,0,-2.4304381277,3.8137731937,1.8919239832<br>H,0,-0.5326625059,2.5959710372,-1.7592548656<br>H,0,-1.9966766386,4.1142975786,-3.0193532174<br>H,0,-3.9137076293,5.3052650726,0.6185598402<br>H,0,-3.6974821143,5.4696435094,-1.8413933779<br>C,0,-0.007594678,2.8446607994,2.6820237886<br>C,0,0.6369280031,4.1175548841,5.0738810587<br>C,0,-0.1288233659,2.1706561244,3.893854651<br>C,0,0.4321332166,4.1697335256,2.6798284873<br>C,0,0.7520236331,4.8017448645,3.8698776196<br>C,0,0.1953411888,2.8037607615,5.0853592407<br>H,0,-0.4624545654,1.1385856478,3.8999357153

	H,0,0.5199012167,4.7074730692,1.7416381156<br>H,0,1.0917088453,5.8301114231,3.8599589211<br>H,0,0.1055318673,2.2666262471,6.0212338137<br>H,0,0.8909255834,4.6122313168,6.0032195699<br>C,0,2.6212561641,0.6794394161,2.6578681967<br>C,0,3.6945811306,1.1826158731,5.1800279935<br>C,0,2.5409097791,-0.282503444,3.6654641301<br>C,0,3.2485754539,1.894592872,2.9277982671<br>C,0,3.776866601,2.1460074332,4.1847618271<br>C,0,3.0815429813,-0.0341474597,4.9170839542<br>H,0,2.0395334216,-1.2238788595,3.4660360237<br>H,0,3.3150035039,2.6579264198,2.1613665918<br>H,0,4.2483359105,3.0996943275,4.387588907<br>H,0,3.0130958511,-0.787771335,5.691476596<br>H,0,4.1043501644,1.3825572138,6.162242855<br>C,0,3.2010369458,-0.4105933008,0.0265726215<br>C,0,5.1906307954,-1.4941192417,-1.5836085966<br>C,0,4.5423796298,-0.1360121853,0.2905642371<br>C,0,2.8614224044,-1.2414696186,-1.0414985013<br>C,0,3.8572763197,-1.7748716699,-1.8446328591<br>C,0,5.5319644732,-0.6764615912,-0.5155752834<br>H,0,4.814329851,0.4923564879,1.1307729471<br>H,0,1.8174665511,-1.4585620699,-1.2421999312<br>H,0,3.5883317467,-2.4189284835,-2.6723002533<br>H,0,6.5725070942,-0.4618519642,-0.305628261<br>H,0,5.9663346328,-1.9177990203,-2.2093925956<br>Br,0,-0.8112983594,-0.3765063757,-1.2921371216
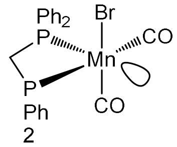<br>(singlet)	Mn,0,-0.2109288337,-0.5545877072,1.2604625501<br>C,0,0.2745040492,-2.2771597361,1.0243787246<br>O,0,0.6421618465,-3.3634660628,0.8945859453<br>C,0,-0.2858701674,-0.8297025628,3.0171261961<br>O,0,-0.3652257653,-1.0831914156,4.1495005234<br>P,0,-0.3361022675,1.8564116854,1.2723507868<br>P,0,1.8685433241,0.2616128543,1.1984576139<br>C,0,1.3259094768,1.8621215118,0.4436456252<br>H,0,1.9431098266,2.752816496,0.572834037<br>H,0,1.1803177424,1.6508417734,-0.6165594993<br>C,0,-1.4209312265,2.972487078,0.3041776821<br>C,0,-3.1886623504,4.5940399492,-1.1147449822<br>C,0,-2.4671737274,3.6227351573,0.96034577<br>C,0,-1.2768004217,3.1349332784,-1.0720521841<br>C,0,-2.1552154992,3.9467377914,-1.7741637019<br>C,0,-3.3439630774,4.4296222691,0.2546031231<br>H,0,-2.5888908783,3.5051392318,2.0315180348<br>H,0,-0.4869076146,2.6193819549,-1.6021283683<br>H,0,-2.0318128966,4.0680902371,-2.8429894497<br>H,0,-4.149535752,4.9317183514,0.7756809411<br>H,0,-3.8737344437,5.2252936817,-1.6667013084<br>C,0,0.0093344366,2.8783136598,2.7650373465<br>C,0,0.6965969721,4.4090552569,4.9910912418<br>C,0,-0.1252056458,2.3558889044,4.0469880489<br>C,0,0.4756801816,4.1860588925,2.6094779902<br>C,0,0.8199736674,4.9449978045,3.7147824988<br>C,0,0.2186162664,3.1192192906,5.1551903579<br>H,0,-0.4894355548,1.3478420761,4.1863106696<br>H,0,0.5655049616,4.6111083821,1.6150341021<br>H,0,1.1828985655,5.9568903886,3.5823691399<br>H,0,0.116020592,2.6976318145,6.1472461735<br>H,0,0.9691385501,5.0016616494,5.8556544768<br>C,0,2.7304360694,0.6903648432,2.7551053296<br>C,0,3.9780942878,1.2263093236,5.1891492461

	C,0,2.8557344032,-0.3084179654,3.7211150301<br>C,0,3.2479385983,1.9568712478,3.0186047256<br>C,0,3.8627595464,2.2239500549,4.2325850925<br>C,0,3.4788522389,-0.0423991084,4.9292190339<br>H,0,2.4537387858,-1.2969824187,3.5280787305<br>H,0,3.1617136939,2.7483182808,2.2843153646<br>H,0,4.2476110426,3.2166992442,4.4308137945<br>H,0,3.5659384067,-0.8249777565,5.6721652127<br>H,0,4.4546377252,1.4376339987,6.138250069<br>C,0,3.1871314421,-0.4384358887,0.1326983034<br>C,0,5.1208563811,-1.506902107,-1.557815197<br>C,0,4.5384513817,-0.337031822,0.4602241662<br>C,0,2.810215986,-1.0893148665,-1.0425924437<br>C,0,3.7764780636,-1.6139497008,-1.8854103661<br>C,0,5.4996565667,-0.8702197938,-0.3852587432<br>H,0,4.8405927601,0.1556581316,1.3766703047<br>H,0,1.7574878751,-1.1815974226,-1.2919942148<br>H,0,3.4762021935,-2.1173231526,-2.7957570231<br>H,0,6.5475882864,-0.7898348755,-0.1240471629<br>H,0,5.8738487316,-1.9254465749,-2.2140317168<br>Br,0,-0.9505380779,-0.3931344692,-1.0818696582
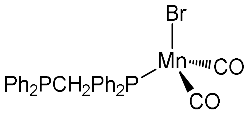	P,0,0.0895482563,0.3244321909,0.1201696327<br>Mn,0,2.2830697743,0.5746820226,0.3519071044<br>C,0,2.1096531901,1.9993934926,1.3737630086<br>O,0,1.9894736626,2.9632161559,2.0116528482<br>C,0,2.3326628756,-0.4609473928,1.794598057<br>O,0,2.368451513,-1.1567350103,2.72127768<br>Br,0,3.2272324833,0.6333017898,-1.7516299925<br>C,0,-0.9998626079,0.0349847527,1.5689034354<br>C,0,-2.6781221017,-0.3172449745,3.7642955138<br>C,0,-0.7353548546,0.6892455048,2.7712408673<br>C,0,-2.1179345673,-0.7945356355,1.4759656447<br>C,0,-2.9476277987,-0.9729985237,2.5727646146<br>C,0,-1.5746665412,0.5184313276,3.8599583753<br>H,0,0.1295073917,1.3346712548,2.8610715851<br>H,0,-2.3403150912,-1.3014961289,0.5448670882<br>H,0,-3.8087022922,-1.6249229027,2.4922070058<br>H,0,-1.3619050851,1.0364888905,4.786514144<br>H,0,-3.3280247304,-0.4566191934,4.6194162846<br>C,0,-0.2425706793,-1.115904467,-0.9575092757<br>C,0,-0.5431182963,-3.3871548567,-2.5442933276<br>C,0,-0.7530281125,-1.0098006785,-2.248107576<br>C,0,0.1247026911,-2.3738866998,-0.4724632859<br>C,0,-0.0301896903,-3.5018049534,-1.259228198<br>C,0,-0.9045855115,-2.1422560912,-3.0350853208<br>H,0,-1.047731222,-0.0467140011,-2.6456686483<br>H,0,0.5250894417,-2.4693096536,0.5315732837<br>H,0,0.2561225055,-4.4711648997,-0.8713848951<br>H,0,-1.3074109116,-2.0483728153,-4.0356669362<br>H,0,-0.6595868604,-4.2682046902,-3.1626560987<br>C,0,-0.6196592811,1.7923174048,-0.740672426<br>H,0,-0.211281469,1.783159951,-1.7530260609<br>H,0,-0.1937321901,2.6724888131,-0.2562318208<br>P,0,-2.490684146,1.9138800742,-0.8150193531<br>C,0,-2.6081096444,3.3398763257,-1.9845903908<br>C,0,-2.8983304446,5.3712978673,-3.8897313075<br>C,0,-2.0440025024,3.2167241601,-3.2571125874<br>C,0,-3.3398732734,4.4908623597,-1.6932558213<br>C,0,-3.4820093502,5.4965161655,-2.6388185183<br>C,0,-2.1787812792,4.2248669943,-4.1968068863<br>H,0,-1.4910057671,2.3211877957,-3.5227397762

	H,0,-3.7971436548,4.6073641266,-0.7181428456<br>H,0,-4.0490623366,6.3855890895,-2.391086347<br>H,0,-1.7225590371,4.1135857016,-5.1727078859<br>H,0,-3.0053162829,6.1600360244,-4.623570983<br>C,0,-2.8235012139,2.7343920024,0.8054741035<br>C,0,-3.4142050833,3.8751218459,3.2851230526<br>C,0,-2.0623127575,3.7955934504,1.2997504744<br>C,0,-3.8890182331,2.2625660163,1.5684313068<br>C,0,-4.1845959683,2.8303619179,2.8005565223<br>C,0,-2.351930989,4.3588105048,2.5312726766<br>H,0,-1.240859357,4.1964266446,0.7156951647<br>H,0,-4.4828389298,1.4351947716,1.196255441<br>H,0,-5.0122806636,2.4473944239,3.3850522787<br>H,0,-1.748871753,5.1777523664,2.9034968368<br>H,0,-3.6388681233,4.3147202886,4.2490840336
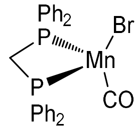<br>(singlet)	Mn,0,-0.1982077039,-0.1329115688,1.7385939436<br>C,0,0.0012458519,-0.1307867257,3.4970672933<br>O,0,0.113026061,-0.1767322395,4.6573469364<br>P,0,-0.1249141274,2.0767581841,1.5942183923<br>P,0,1.9436971217,0.3497284528,1.4581989843<br>C,0,1.6626289186,2.1521059129,1.0779861029<br>H,0,2.2951601241,2.8549059692,1.6230801935<br>H,0,1.7532031936,2.3380809764,0.0074848114<br>C,0,-1.0326958682,2.9629797237,0.2579355434<br>C,0,-2.4258907393,4.1618207121,-1.8369400737<br>C,0,-1.8680927507,4.050351868,0.5001472181<br>C,0,-0.9164137563,2.4702994389,-1.0429786952<br>C,0,-1.6025036468,3.0709030419,-2.0840966277<br>C,0,-2.5605230385,4.645809864,-0.5453077798<br>H,0,-1.9784877824,4.4371009084,1.5062070591<br>H,0,-0.3022735396,1.5953422625,-1.2356382704<br>H,0,-1.5029102512,2.6807383133,-3.0892403168<br>H,0,-3.2075783664,5.4912941506,-0.3464670687<br>H,0,-2.9673957687,4.6278793588,-2.6506507275<br>C,0,-0.2668560299,3.2180609641,3.0238045226<br>C,0,-0.5089893945,4.9204109048,5.2157545337<br>C,0,-1.0918910211,2.8774680703,4.0925888765<br>C,0,0.4312419928,4.4260611054,3.0605832718<br>C,0,0.313023556,5.2707251305,4.1525086639<br>C,0,-1.2138877936,3.7268677595,5.1826171413<br>H,0,-1.6313781525,1.9382046835,4.0730737969<br>H,0,1.0619293124,4.7127895861,2.2259015848<br>H,0,0.8612369222,6.2044629642,4.1737638076<br>H,0,-1.854802534,3.4500333825,6.010074272<br>H,0,-0.5982166863,5.5794993573,6.0703344321<br>C,0,3.1386742407,0.361375411,2.8528350098<br>C,0,4.8125767162,0.3086544024,5.0816909212<br>C,0,4.1540983644,-0.5876613771,2.9498588187<br>C,0,2.9589376355,1.2716401025,3.8945796711<br>C,0,3.795379305,1.2493385756,4.9982477466<br>C,0,4.9874670804,-0.6106466534,4.0583982747<br>H,0,4.2991966021,-1.307523245,2.1526481035<br>H,0,2.1499702936,1.9954125814,3.8526971448<br>H,0,3.6465578822,1.9635559753,5.7984561703<br>H,0,5.7753291688,-1.3510395105,4.1216613749<br>H,0,5.4639780513,0.2891802717,5.9464070937<br>C,0,2.9476062656,-0.2819881734,0.0525719899<br>C,0,4.4109606763,-1.2155974911,-2.1228193794<br>C,0,4.1882525681,0.2805431785,-0.2558132302<br>C,0,2.4474002289,-1.3112425658,-0.7388991712<br>C,0,3.1802051547,-1.7773108553,-1.8226114759

	C,0,4.9147182767,-0.184219443,-1.3390113249<br>H,0,4.5880510802,1.0785827409,0.3612417324<br>H,0,1.4766073815,-1.7369337982,-0.5077678509<br>H,0,2.7847816266,-2.5797617038,-2.4327223635<br>H,0,5.8759637965,0.2564771778,-1.5733811641<br>H,0,4.9814800271,-1.5787287389,-2.9687591836<br>Br,0,-1.3390099301,-1.2008129746,0.0046776501
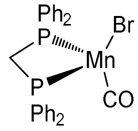<br>(triplet)	Mn,0,-0.3743441535,-0.3752599302,1.7227792087<br>C,0,-0.2335289532,-0.3683606556,3.4984787612<br>O,0,-0.1647128886,-0.4203851326,4.6656113899<br>P,0,-0.4062741517,1.9906631788,1.5627836291<br>P,0,1.9094746843,0.1038945425,1.4670447447<br>C,0,1.3759377343,1.8374193302,1.0180684004<br>H,0,2.0207804157,2.6000795554,1.4606609287<br>H,0,1.414916245,1.927517044,-0.0679213494<br>C,0,-1.1677859118,3.0085618927,0.2377050356<br>C,0,-2.3992937693,4.4067196898,-1.8354267997<br>C,0,-1.847811916,4.1971369118,0.4966328045<br>C,0,-1.1331304514,2.513723571,-1.0678888618<br>C,0,-1.7371563896,3.2147293623,-2.0974183087<br>C,0,-2.4579272865,4.8918032192,-0.5380270732<br>H,0,-1.8987823903,4.5850446485,1.5067715958<br>H,0,-0.6547937704,1.5607649564,-1.2719547169<br>H,0,-1.7014148607,2.8217912985,-3.1057342765<br>H,0,-2.9806155325,5.8164845959,-0.3264842571<br>H,0,-2.8766622392,4.9510002323,-2.6406256522<br>C,0,-0.3306282042,3.125893441,3.0010961258<br>C,0,-0.1464213315,4.7556543209,5.2530455558<br>C,0,-1.0102539307,2.7918309337,4.1696650207<br>C,0,0.4330070058,4.2950754496,2.9673085576<br>C,0,0.5274322086,5.1026183571,4.0885259374<br>C,0,-0.9190002533,3.6051539606,5.2904930686<br>H,0,-1.5957406683,1.881447721,4.2059193555<br>H,0,0.9527669426,4.5762362097,2.0574142522<br>H,0,1.1267815499,6.0039775973,4.0551278769<br>H,0,-1.4453173036,3.3316418407,6.1960776098<br>H,0,-0.0678685454,5.3850522954,6.1307436797<br>C,0,3.063665059,0.3334520655,2.8697004987<br>C,0,4.6923603464,0.5836510101,5.1197305665<br>C,0,4.2042850297,-0.4567388371,3.0068308531<br>C,0,2.7385038434,1.2362220793,3.8826211452<br>C,0,3.5521516844,1.3652737126,4.9957770471<br>C,0,5.01331789,-0.328839986,4.1257873307<br>H,0,4.4647573434,-1.1699359464,2.2335277536<br>H,0,1.8365724971,1.8352958989,3.8092885748<br>H,0,3.288377962,2.0734793106,5.7716257701<br>H,0,5.8982531556,-0.9459643258,4.2198968684<br>H,0,5.3260841554,0.6816870184,5.9921272777<br>C,0,3.0008790255,-0.388319463,0.0782578278<br>C,0,4.5802878789,-1.1159240391,-2.0926860115<br>C,0,4.1865784374,0.2990179602,-0.1915153448<br>C,0,2.6131952449,-1.4393508773,-0.7485013866<br>C,0,3.4047133397,-1.8012818616,-1.8303226733<br>C,0,4.9704500024,-0.063204288,-1.2736422543<br>H,0,4.4975034393,1.1146427967,0.4527411665<br>H,0,1.6812006638,-1.9577170637,-0.5517966006<br>H,0,3.0973879683,-2.6195119858,-2.4693047105<br>H,0,5.8878222182,0.4743974155,-1.4794655439<br>H,0,5.195864543,-1.3992471565,-2.9374984601<br>Br,0,-1.1841770169,-1.2474474766,-0.351513558

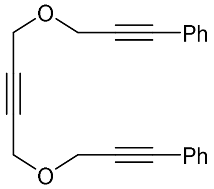<br><br>Triyne (CI-Symm)	O,0,0.7739294459,-0.2562213178,1.3924928456<br>C,0,0.3899609527,0.4266075204,0.2034588569<br>H,0,0.9085193359,-0.0947655153,-0.6029355689<br>C,0,0.383147721,0.3970276464,2.5939772422<br>H,0,0.6016955295,-0.3164150376,3.3902440225<br>H,0,-0.6896593527,0.2947113052,0.03226762<br>H,0,1.013173279,1.281971174,2.762540284<br>C,0,-1.0103970481,0.8037071671,2.6222431011<br>C,0,-2.1486984523,1.1930358426,2.5827364268<br>C,0,-3.491435483,1.7350348114,2.4651935101<br>H,0,-3.5260723567,2.7158565708,2.9616898533<br>H,0,-4.2242342566,1.0936956649,2.9573787732<br>O,0,-3.9401784765,1.8469169453,1.1217013085<br>C,0,-3.076745895,2.6090402133,0.2860369074<br>H,0,-2.1145175953,2.0917218507,0.1565153337<br>H,0,-3.5698747859,2.6276169597,-0.687371127<br>C,0,0.6987022978,1.843442263,0.2012581044<br>C,0,0.9005678273,3.0302583317,0.2910167246<br>C,0,1.1566138264,4.4173087293,0.4350782968<br>C,0,1.6830460884,7.1394689658,0.7642072178<br>C,0,0.9664145127,5.0357796118,1.6780478695<br>C,0,1.604959736,5.1902667969,-0.6426920284<br>C,0,1.8622294736,6.5400548941,-0.4748334453<br>C,0,1.2340591627,6.3831155282,1.8380304753<br>H,0,0.5948983511,4.4457493204,2.5077444089<br>H,0,1.7509241171,4.719022494,-1.6060602368<br>H,0,2.2083354975,7.1269884752,-1.3165484013<br>H,0,1.071128363,6.8511293194,2.800947863<br>H,0,1.8816989785,8.1963022054,0.8918731307<br>C,0,-2.8149127995,3.9544666858,0.7569685284<br>C,0,-2.5564349014,5.0576820866,1.1717079224<br>C,0,-2.2413682418,6.3531615169,1.6511902821<br>C,0,-1.5672803225,8.8925312522,2.6007668559<br>C,0,-2.1880424678,6.6088114717,3.0276473118<br>C,0,-1.9505641643,7.3927280464,0.7597145828<br>C,0,-1.6158691843,8.6482563358,1.2350695402<br>C,0,-1.8556310905,7.8684877863,3.4935862129<br>H,0,-2.4079647882,5.8066282223,3.7212221626<br>H,0,-1.9767534797,7.1968882869,-0.3045203262<br>H,0,-1.3876347533,9.4415189539,0.5339349448<br>H,0,-1.8207344245,8.0529632969,4.5601764157<br>H,0,-1.3072493948,9.8771224935,2.9685742618
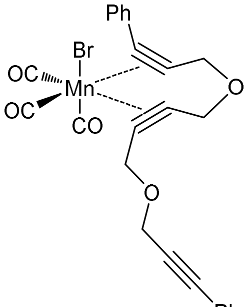 	C,0,-0.8457507341,-0.4788651566,-1.6683963214<br>O,0,-0.9952963734,-0.8046618626,-2.758697714<br>Br,0,1.849511396,0.2245080147,-0.4781339167<br>Mn,0,-0.6358622615,0.1051894576,0.0342084221<br>C,0,0.0952594536,-2.4924592492,0.2094692291<br>C,0,-0.6594746498,-2.1076562372,1.0937587109<br>C,0,-0.5649991341,0.4794439644,2.4236719124<br>C,0,0.135176227,1.3730986257,1.9719412228<br>C,0,1.007066269,2.5495964202,1.8518681008<br>C,0,0.9455765087,-3.0524452008,-0.7705289296<br>C,0,2.6024196045,-4.2503428521,-2.654554067<br>C,0,2.3303097166,-3.1098209935,-0.5584993585<br>C,0,0.4033570204,-3.6082018183,-1.9373974769<br>C,0,1.2314982452,-4.2025107375,-2.8705233138<br>C,0,3.1474196619,-3.7077118999,-1.4985728912<br>H,0,2.7425543567,-2.6717455238,0.3395782333<br>H,0,-0.6657978654,-3.5669106139,-2.0991543247<br>H,0,0.8073772892,-4.6267184562,-3.7712867897<br>H,0,4.2162276538,-3.7445931432,-1.3329214104

	H,0,3.2482724433,-4.7129985773,-3.390273911<br>C,0,-1.5343592043,-2.346892682,2.250552011<br>H,0,-1.4781297787,-3.4059695632,2.5078892988<br>H,0,-2.5772078052,-2.1229293497,1.9832906867<br>C,0,-1.4257229068,-0.272653393,3.3347627555<br>H,0,-1.2668493131,0.1231021528,4.3399145742<br>H,0,-2.479031162,-0.0949304535,3.0705481146<br>O,0,-1.1655749473,-1.6537292703,3.4202962722<br>C,0,-0.7889712296,1.8020805999,-0.5537772409<br>O,0,-0.8924750071,2.8788818168,-0.9417219041<br>C,0,-2.4088246771,0.0072402918,0.3310118919<br>O,0,-3.5532569532,-0.037993169,0.4818342796<br>H,0,1.5257311176,2.545629441,0.8864716062<br>H,0,0.3824729221,3.4451922473,1.901362242<br>O,0,1.9099533411,2.6347480771,2.9297657525<br>C,0,2.955145093,1.6672286111,2.8144803344<br>C,0,3.8384624516,1.7649567289,3.9487025983<br>H,0,2.526484229,0.6558831301,2.7430075913<br>H,0,3.5054510534,1.8249222122,1.8753492948<br>C,0,4.5961444397,1.827361447,4.8842707564<br>C,0,5.4842261998,1.9055609019,5.9861296344<br>C,0,5.3439255951,2.9176250646,6.9444623259<br>C,0,6.2141551985,2.9905103911,8.0175084573<br>C,0,7.2371343703,2.0624809468,8.1564784642<br>C,0,6.5192316161,0.9736602729,6.1362304801<br>C,0,7.3849401077,1.055877367,7.2122503529<br>H,0,4.546535986,3.6408607934,6.8333653394<br>H,0,6.0936830389,3.7783873675,8.7505977443<br>H,0,7.9164969561,2.1236316025,8.9972218227<br>H,0,6.6333431613,0.1893967229,5.3991018861<br>H,0,8.1810032795,0.3289055336,7.314647171
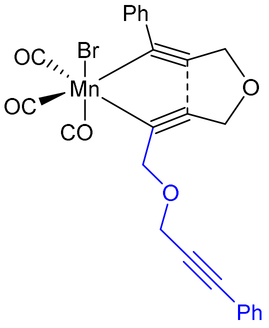	Br,0,2.2556035582,0.2241797396,-2.1104504801<br>Mn,0,0.5226591015,0.1710974681,-0.1560516723<br>O,0,-0.9906454128,1.7438327134,-2.1960245038<br>O,0,1.4103656533,2.8444070868,0.7809969972<br>O,0,-1.6008429343,0.108080948,1.9119677666<br>C,0,-0.7684080964,0.1311627468,1.1194747311<br>C,0,-0.4130008228,1.1247516336,-1.4291408977<br>C,0,1.0726661423,1.8079255021,0.425703704<br>C,0,-0.5647283249,-1.4137422987,-1.1191726595<br>C,0,-1.6872276635,-1.4077838619,-2.0150936776<br>C,0,-1.4687570978,-1.2857018027,-3.391756602<br>C,0,-2.5438667522,-1.2889127152,-4.2628535235<br>C,0,-3.8397841348,-1.4009223025,-3.7780914976<br>C,0,-4.0607012803,-1.509021575,-2.4121147433<br>C,0,-2.9939760131,-1.5070451125,-1.5298560849<br>C,0,0.4046671138,-2.0350550732,-0.6085036299<br>C,0,1.1306664084,-3.3220209738,-0.6759291438<br>O,0,1.295070378,-3.843772212,0.6199375781<br>C,0,1.6577823983,-2.8089097381,1.5149671338<br>C,0,1.6369783046,-1.4752281247,0.8767779754<br>C,0,2.1941905862,-0.3425262277,0.9497553828<br>C,0,3.3523236237,0.3564089373,1.4986394694<br>O,0,4.159823277,-0.5813514902,2.1791458105<br>C,0,5.3659374911,0.021231385,2.6377334189<br>C,0,6.1747230833,-0.9530725025,3.3265650516<br>C,0,7.6781197936,-2.7080795505,4.5685114128<br>C,0,7.7096685376,-2.7651497084,5.9676594617<br>C,0,8.5063574342,-3.6945373458,6.6122323435<br>C,0,9.2814918535,-4.5815952642,5.8778102878<br>C,0,9.2571359609,-4.5348222065,4.4905971044

	C,0,8.4649707876,-3.6075386716,3.8377994816<br>H,0,0.545407138,-4.0405799857,-1.250624162<br>H,0,2.092771853,-3.1576950617,-1.1835163243<br>H,0,0.9794201585,-2.8487661461,2.3748789094<br>H,0,2.6798530947,-2.9526132985,1.8821310475<br>H,0,-3.1620779901,-1.586297668,-0.4628582006<br>H,0,-0.4539646263,-1.180238065,-3.7569050826<br>H,0,-2.3678978234,-1.1988297398,-5.3272636428<br>H,0,-5.0702243713,-1.5932675978,-2.0301607469<br>H,0,-4.6773188849,-1.3997910539,-4.4639681905<br>H,0,3.0384004751,1.1680890416,2.1742638428<br>H,0,3.9012274468,0.8326931912,0.6697013249<br>H,0,5.9200534511,0.4420187607,1.7846572119<br>H,0,5.1341425156,0.8644851396,3.30696009<br>C,0,6.8642369311,-1.7579226623,3.9006510183<br>H,0,8.4431560253,-3.5674920693,2.7565027921<br>H,0,9.8599670079,-5.2251256295,3.9140205847<br>H,0,9.9030800377,-5.3081672083,6.3857121747<br>H,0,7.1027125746,-2.0730245378,6.5368481152<br>H,0,8.5222945322,-3.728070113,7.694382043
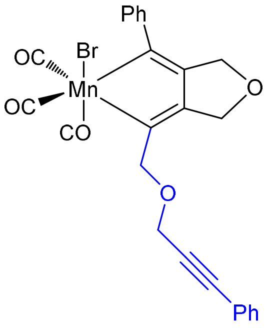	Br,0,2.4759933091,-0.5518976244,-0.2129735177<br>Mn,0,0.0533470227,0.0235160496,-0.0046221343<br>C,0,-0.289664864,-0.6523417837,-1.749484666<br>O,0,-0.5442670389,-0.9578403878,-2.8219572805<br>C,0,0.410725057,1.6648758754,-0.844216581<br>O,0,0.6141147717,2.6139941679,-1.4517123907<br>C,0,-0.2398988266,-1.7712303348,0.9404603126<br>C,0,-0.333814302,-3.1002768374,0.3807287573<br>C,0,0.3967768328,-3.4503245342,-0.7704212761<br>C,0,0.3124067943,-4.7206820592,-1.3068314918<br>C,0,-0.5346846235,-5.6643845922,-0.7406091945<br>C,0,-1.2814137474,-5.3383915616,0.3860329816<br>C,0,-1.165351521,-4.085264473,0.9528190084<br>C,0,-0.3626849249,-1.5865538678,2.3307025469<br>C,0,-0.5040636578,-2.4719830117,3.5320189481<br>O,0,-0.1313395071,-1.6550264273,4.6416587531<br>C,0,-0.2047271007,-0.28731635,4.2652745352<br>C,0,-0.1996664201,-0.2893075077,2.7765030265<br>C,0,-0.0133284282,0.7328198941,1.8247739952<br>C,0,-0.0766928018,2.1442776393,2.299336175<br>O,0,0.7776811453,3.0565255648,1.6491827262<br>C,0,2.1713375304,2.6992305386,1.640035594<br>C,0,2.5654332038,1.6868502733,2.590694747<br>H,0,1.0738686245,-2.7210857543,-1.1953196899<br>H,0,-1.7621279496,-3.8387945658,1.8215947482<br>H,0,-1.9506471142,-6.0688343906,0.823035727<br>H,0,0.9031625458,-4.9758464799,-2.1773560164<br>H,0,-0.6143337439,-6.6531140567,-1.1751776163<br>C,0,-1.7290961687,0.3058368143,0.2565428923<br>O,0,-2.8481899367,0.4708001655,0.4701588755<br>H,0,-1.1288997707,0.181738382,4.6449744509<br>H,0,0.6373105992,0.2515266326,4.7092530174<br>H,0,0.1468785805,-3.3512239703,3.5045236289<br>H,0,-1.5365703628,-2.8305767298,3.6637838721<br>H,0,0.0986373687,2.1783243465,3.3862706172<br>H,0,-1.0866606436,2.5410384814,2.1400797559<br>H,0,2.43984373,2.3345447176,0.6403581157<br>H,0,2.7181275332,3.6314562266,1.8048882589<br>C,0,2.883023649,0.7629183049,3.3031029225<br>C,0,3.1884536292,-0.3846373287,4.0760500787<br>C,0,3.1706303161,-1.6460969604,3.4636662221

	C,0,3.4252234849,-2.7822034159,4.210851907<br>C,0,3.6935015536,-2.6832224341,5.5691625836<br>C,0,3.4619743593,-0.2941325374,5.447011502<br>C,0,3.7114053969,-1.4379315984,6.1835471787<br>H,0,2.9550502125,-1.7072284818,2.4023626866<br>H,0,3.412385947,-3.7524734859,3.7295136169<br>H,0,3.8882417051,-3.575703508,6.1505209008<br>H,0,3.4745641792,0.6794466223,5.9207972977<br>H,0,3.9208263723,-1.3590356455,7.2428538915
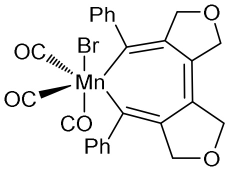	Mn,0,0.3538189732,1.0588264615,0.0000784531<br>C,0,1.4200642103,2.0645144931,1.2846196121<br>C,0,1.4203295781,2.0642699934,-1.284474733<br>O,0,2.0775396829,2.6812949987,1.9811140351<br>O,0,2.0780246063,2.6810260507,-1.9807804999<br>C,0,-1.053816231,2.1709748653,-0.0000498599<br>O,0,-1.9693635267,2.8721346496,-0.0003312635<br>Br,0,2.5510419616,-0.3400927119,0.0004026156<br>C,0,-0.4703320512,0.0317132712,-1.4279570225<br>C,0,-0.4703582027,0.0317677077,1.4279587783<br>C,0,-2.8884706336,-0.1854299378,0.7060044679<br>C,0,-2.888424689,-0.1856902107,-0.7059541722<br>C,0,-1.8576628729,-0.1610863447,-1.6247520984<br>C,0,-1.8577316911,-0.1607036061,1.6248089607<br>C,0,-2.4568512588,-0.5058965327,-2.9683259074<br>C,0,-2.4570048396,-0.5049753147,2.9684820812<br>H,0,-2.1842955865,-1.5332768668,-3.2596421009<br>H,0,-2.1847054365,-1.5323473787,3.2600748221<br>H,0,-2.1171400293,0.1528628392,-3.7707733926<br>H,0,-2.1171307948,0.1539208456,3.7707480763<br>C,0,-4.1597895155,-0.4825162417,-1.4473370582<br>C,0,-4.1598900603,-0.4818633296,1.4474690358<br>H,0,-4.9712829536,0.2082900545,-1.1936879218<br>H,0,-4.9713335243,0.2088875715,1.1935268004<br>O,0,-3.8555576671,-0.3520780702,-2.8162223774<br>O,0,-3.8556618029,-0.3508426596,2.8163060266<br>C,0,0.3420623639,-0.5381573086,-2.5262660282<br>C,0,0.3419561729,-0.5383123841,2.5262097416<br>C,0,1.8222407368,-1.6848828767,-4.5995545851<br>C,0,1.8220513704,-1.6854427635,4.5993262483<br>C,0,0.7421856504,-1.8747351354,-2.4586193854<br>C,0,0.7413548309,-1.8751161763,2.4587030698<br>C,0,0.6853454884,0.2105712682,-3.6518848563<br>C,0,0.6859668564,0.210460393,3.6515694946<br>C,0,1.4215593625,-0.360434496,-4.6790977479<br>C,0,1.4221185338,-0.3607599654,4.6787108005<br>C,0,1.4778481284,-2.4386082401,-3.4865422029<br>C,0,1.4769812482,-2.4391915208,3.4865366119<br>H,0,0.4935468071,-2.4573132964,-1.5794721261<br>H,0,0.4921930089,-2.4577187889,1.5797164835<br>H,0,0.3691286357,1.2451414053,-3.7250527573<br>H,0,0.3703630444,1.24522575,3.7246082509<br>H,0,1.6828772985,0.237254823,-5.5434600511<br>H,0,1.6839922479,0.2369562154,5.5428863526<br>H,0,1.793523387,-3.4716685888,-3.4110326381<br>H,0,1.7920908508,-3.4724326025,3.411136325<br>H,0,2.403267253,-2.1270547349,-5.3985764467<br>H,0,2.4030465523,-2.1277736147,5.3982826681<br>H,0,-4.5191914289,-1.4980361009,1.2062944786<br>H,0,-4.5190377448,-1.4986043577,-1.205727058

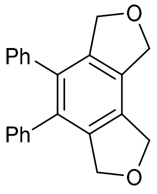	C,0,-0.112742,1.1704971,-0.2460788<br>C,0,0.0608967,1.2509054,2.556625<br>C,0,0.095475,0.0191454,0.5129254<br>C,0,-0.235703827,2.4063986795,0.430611006<br>C,0,-0.138487762,2.410814549,1.8218629489<br>C,0,0.1969656927,0.0492341889,1.8963316937<br>C,0,0.2913243,-1.398126,0.0718921<br>H,0,1.0354038,-1.5099314,-0.7226453<br>H,0,-0.6431651,-1.8480253,-0.2952793<br>C,0,0.4467899,-1.3337798,2.3929724<br>H,0,1.2924503,-1.4060001,3.0847466<br>H,0,-0.4336357,-1.7438191,2.9141027<br>C,0,0.0875702293,1.5956390011,4.0065350269<br>H,0,1.1018622024,1.5029948742,4.4278272217<br>H,0,-0.572915347,0.9725068177,4.6183960514<br>C,0,-0.2597043957,3.5478318152,2.7883120191<br>H,0,-1.1407912576,4.1741154114,2.6183142877<br>H,0,0.6181848729,4.2102710332,2.7570171298<br>O,0,0.7473602,-2.0950467,1.2292592<br>O,0,-0.3686801136,2.9415021342,4.074256971<br>C,0,-0.5235866,3.6689876,-0.2877136<br>C,0,-1.0860366,6.0615164,-1.6280339<br>C,0,0.2718515,4.7996989,-0.0977409<br>C,0,-1.6113801,3.7630077,-1.1588282<br>C,0,-1.8907658,4.9469143,-1.8199393<br>C,0,-0.003363,5.98374,-0.7648858<br>H,0,1.132022,4.7392846,0.5591861<br>H,0,-2.240524,2.8946487,-1.3145701<br>H,0,-2.7412,5.0003697,-2.4883237<br>H,0,0.6331873,6.8466464,-0.612531<br>H,0,-1.3023202,6.9860473,-2.148442<br>C,0,-0.1368181085,1.0793491918,-1.7239522427<br>C,0,-0.1614923734,0.8915386548,-4.5170009266<br>C,0,0.7167289487,1.8649510753,-2.501938249<br>C,0,-0.9976136702,0.1940625798,-2.3740430495<br>C,0,-1.0136991738,0.1038860607,-3.7576571999<br>C,0,0.7060150865,1.7704012626,-3.8831350632<br>H,0,1.3933565822,2.5550032659,-2.0119097202<br>H,0,-1.6811195819,-0.4089187034,-1.7872470878<br>H,0,-1.6976083669,-0.5811823198,-4.2434197492<br>H,0,1.3783992772,2.3855438591,-4.4682270223<br>H,0,-0.1725940927,0.8208654407,-5.597390958
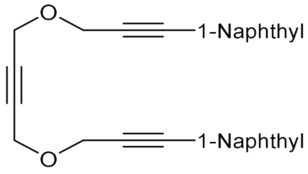	C,0,-0.3761264316,-1.464578134,3.3914235081<br>C,0,-0.5434972481,-2.2314068263,4.305340572<br>C,0,-0.8511944723,-3.1462478427,5.3883873112<br>H,0,0.039426929,-3.6910689485,5.7061363401<br>H,0,-1.205177192,-2.5659717418,6.2526438786<br>C,0,-0.2778076243,-0.5571812011,2.2637860998<br>H,0,-0.2091130551,-1.1476002233,1.3387726038<br>H,0,0.6257698667,0.051429509,2.3281420192<br>O,0,-1.799737626,-4.1476479297,5.0441211336<br>O,0,-1.3499874868,0.3723756124,2.1777051922<br>C,0,-3.0033198072,-3.6374015564,4.4702632507<br>H,0,-2.817250514,-3.2990009224,3.4393632098<br>H,0,-3.6738177225,-4.4958788334,4.410592784<br>C,0,-2.6452211679,-0.2267117895,2.219398046<br>H,0,-3.3385957919,0.5832753842,1.9886088271<br>H,0,-2.8722739239,-0.5665695188,3.2415699665<br>C,0,-3.5980757759,-2.5442916012,5.2123268996<br>C,0,-4.0098503478,-1.58014866,5.81224913<br>C,0,-2.8107368927,-1.3468518754,1.3154217031

	C,0,-2.8761801134,-2.3279614607,0.6138678862<br>H,0,-6.2586945715,-1.3242653729,7.1835661748<br>C,0,-5.6684360431,-0.4175118785,7.1615743356<br>C,0,-4.4634664498,-0.4182605865,6.4827772933<br>C,0,-5.3919992534,1.8886387041,7.7837111975<br>C,0,-3.6710172739,0.7730010117,6.4459242929<br>C,0,-6.1299237014,0.7349051538,7.8095688726<br>C,0,-4.154640544,1.9404132445,7.1068520223<br>C,0,-2.4318255877,0.8363877636,5.7757877894<br>H,0,-7.0778552009,0.7062810557,8.3316976205<br>H,0,-3.7505488214,4.0032171092,7.5653476535<br>H,0,-5.7492440964,2.7820073552,8.2828421374<br>C,0,-1.7070351572,1.9984176455,5.7449413743<br>H,0,-2.0562018324,-0.0517376946,5.2796937375<br>H,0,-0.7636400995,2.0269872867,5.2134658646<br>C,0,-2.1832258973,3.1510250484,6.39309034<br>H,0,-1.6017768172,4.0640097531,6.3631855209<br>C,0,-3.3777790851,3.1190562138,7.0609337081<br>H,0,-4.3492284924,-2.7161701313,-1.5500890306<br>C,0,-3.7588987214,-3.580949873,-1.2766709793<br>C,0,-2.9375688565,-3.508159631,-0.1663392544<br>C,0,-3.0972242703,-5.8537475509,-1.699021213<br>C,0,-2.1497792108,-4.643238601,0.2070522846<br>C,0,-3.8372715618,-4.7525607014,-2.0396379249<br>C,0,-2.24203364,-5.8311251585,-0.5765183243<br>C,0,-1.2893940997,-4.6319785712,1.3243291173<br>H,0,-4.4902150788,-4.7808529153,-2.9027000375<br>H,0,-1.5472048711,-7.8548588266,-0.8009802821<br>H,0,-3.1588220753,-6.76200742,-2.2873517667<br>C,0,-0.5627773635,-5.7424062092,1.6642771948<br>H,0,-1.2094625489,-3.7276566406,1.9174413635<br>H,0,0.0822263665,-5.7145031089,2.5340290457<br>C,0,-0.6544676841,-6.9152036302,0.8951883689<br>H,0,-0.0749595091,-7.7869125297,1.1723386377<br>C,0,-1.4729896178,-6.9548061615,-0.201373336
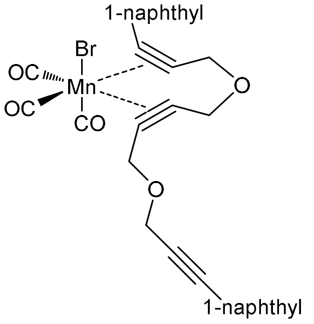	C,0,-0.6274525772,-0.4404640473,-1.635004023<br>O,0,-0.7596875444,-0.5816885908,-2.7657209945<br>Br,0,2.014566718,0.3212874142,-0.4488044376<br>Mn,0,-0.4252135556,-0.0623919804,0.1309475577<br>C,0,0.4603905744,-2.5979354585,-0.0441957644<br>C,0,-0.0894018443,-2.3111182293,1.0150383788<br>C,0,-0.2336269948,0.1773909496,2.5393403271<br>C,0,0.308124808,1.1813894347,2.1036754974<br>C,0,1.0090508953,2.4686348495,2.0012089182<br>C,0,1.0299481194,-3.0294531685,-1.2579982337<br>C,0,2.1808043584,-3.9118895445,-3.6469354373<br>C,0,2.3968944016,-2.9322217876,-1.4654973084<br>C,0,0.1858183862,-3.6004626645,-2.2652686029<br>C,0,0.7857166029,-4.0431057648,-3.4790124247<br>C,0,2.9677576375,-3.3725605129,-2.6628869579<br>H,0,3.0117156551,-2.4941659847,-0.6922000246<br>C,0,-1.2075424891,-3.7377073546,-2.1086648648<br>C,0,-0.0350951516,-4.6004523545,-4.4834455816<br>H,0,4.0359230177,-3.2791842004,-2.8074975944<br>H,0,2.6225706063,-4.2495754957,-4.5774952833<br>C,0,-0.7115956253,-2.7675725051,2.2689982679<br>H,0,-0.4093345805,-3.801202189,2.4443906959<br>H,0,-1.8056159847,-2.7555516279,2.1423769079<br>C,0,-0.8741535663,-0.760023048,3.4567459364<br>H,0,-0.7145461508,-0.3908870484,4.4719436597<br>H,0,-1.9596247684,-0.7752314369,3.2759348267

	O,0,-0.3499030373,-2.0661527124,3.4330078735<br>C,0,-0.8464670876,1.6414450195,-0.274705649<br>O,0,-1.1299521919,2.7205768513,-0.5529545587<br>C,0,-2.1436505775,-0.4615282384,0.4976783498<br>O,0,-3.2479881815,-0.7320985184,0.7015933647<br>H,0,1.4660893354,2.5772113585,1.010994172<br>H,0,0.2789158108,3.2713756376,2.1310458842<br>O,0,1.9545347298,2.6212907277,3.0338730565<br>C,0,3.1114478707,1.8098626156,2.8111664823<br>C,0,4.0378303121,1.9721150854,3.9028413934<br>H,0,2.8177975116,0.7551394687,2.7048848322<br>H,0,3.5791317416,2.0881395084,1.8554441436<br>C,0,4.8318042355,2.0988070902,4.8020225599<br>C,0,5.7599934123,2.2557475758,5.8601468427<br>C,0,6.0886040052,3.5216233007,6.3116733496<br>C,0,6.9985354538,3.7006016946,7.3607129002<br>C,0,7.5895712118,2.6199206978,7.959589943<br>C,0,6.3613383151,1.1114158009,6.4716772558<br>C,0,7.2912746717,1.3077934836,7.5348224718<br>H,0,5.6280243883,4.3807397356,5.8418415215<br>H,0,7.2334082397,4.7038211422,7.6932160564<br>H,0,8.2964978664,2.7563953705,8.7697935337<br>C,0,6.0692612998,-0.204755121,6.0597030034<br>C,0,7.8875074531,0.1768187647,8.1349749995<br>C,0,6.6652429463,-1.2796855161,6.6644586641<br>H,0,5.3609353004,-0.348253404,5.2527750536<br>H,0,6.4288360187,-2.2840201993,6.3357025577<br>C,0,7.5836804331,-1.0885471098,7.7112707844<br>H,0,8.5943059026,0.3326200953,8.9420126854<br>H,0,8.0492607945,-1.9458752897,8.1811783963<br>C,0,-1.9758440354,-4.2808960784,-3.1046545991<br>H,0,-1.6684632425,-3.3958125946,-1.1885674408<br>H,0,-3.0461419102,-4.3727107024,-2.9695773527<br>C,0,-1.3865706493,-4.7169521047,-4.3030812942<br>H,0,0.429191092,-4.9335606177,-5.4043153182<br>H,0,-2.0052944863,-5.1438705714,-5.081963659
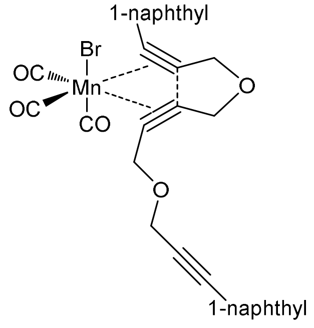	Br,0,0.2781558276,-4.2382581557,-0.6214221445<br>Mn,0,0.7639544152,-1.7205740806,-1.1064385403<br>O,0,-1.1997943735,-2.2916859623,-3.2790050492<br>O,0,2.736617355,-2.4416796243,-3.2012560546<br>O,0,1.1928979277,1.1397766025,-1.7613755926<br>C,0,1.0190937569,0.0351742236,-1.4949911269<br>C,0,-0.4706727114,-2.0754901917,-2.4258845516<br>C,0,1.9828697782,-2.1653980164,-2.3823724025<br>C,0,-1.0727856524,-1.1742255046,-0.0925904657<br>C,0,-2.4255946832,-0.9872541712,-0.5290638685<br>C,0,-3.2203567424,-2.0918868692,-0.7741535052<br>C,0,-4.5486728303,-1.9381352522,-1.1893092709<br>C,0,-5.0764067719,-0.688498476,-1.3779163598<br>C,0,-4.3017549407,0.467561347,-1.1442793951<br>C,0,-2.9513757917,0.328545747,-0.7057033941<br>C,0,-0.2603916537,-1.3076922478,0.8602598253<br>C,0,-0.234703131,-1.3990092255,2.3388920792<br>O,0,0.7061037827,-0.4693122068,2.8133828762<br>C,0,1.9383752936,-0.7333797422,2.1808668602<br>C,0,1.7672746956,-1.4420983816,0.8856880144<br>C,0,2.4425275574,-2.1157771516,0.0581028789<br>C,0,3.693285165,-2.8393594756,-0.1427462412<br>O,0,4.4525894227,-2.7768326686,1.0483352749<br>C,0,5.5861150478,-3.6301777688,0.9561634342<br>C,0,6.4057063725,-3.5271097389,2.1370230581

	C,0,8.0469599108,-3.4405804134,4.1797748053<br>C,0,7.5876586342,-3.0987685607,5.4894320879<br>C,0,8.531376211,-3.0578813906,6.557758302<br>C,0,9.8857298081,-3.3562724345,6.2987069278<br>C,0,10.3011136451,-3.6833080576,5.035394692<br>C,0,9.3860739737,-3.724679543,3.9771426339<br>H,0,-1.2092450213,-1.1204854971,2.7398270235<br>H,0,-0.0015644533,-2.4303018456,2.6422078793<br>H,0,2.4622197809,0.218227166,2.0642171759<br>H,0,2.5738981805,-1.3952695275,2.7855242387<br>C,0,-2.1937401527,1.4925701229,-0.4623762054<br>H,0,-2.8012967106,-3.0815145984,-0.6400752667<br>H,0,-5.1522322677,-2.8189207335,-1.3663108821<br>C,0,-4.8270864449,1.7652564652,-1.3271083326<br>H,0,-6.1010310215,-0.5680376134,-1.709856733<br>H,0,4.2621838059,-2.4174386839,-0.9863583963<br>H,0,3.4555287328,-3.8811737775,-0.4131610673<br>H,0,5.2565457721,-4.6714249268,0.8126584803<br>H,0,6.184808549,-3.3729604447,0.0676412387<br>C,0,7.1491361531,-3.4909652872,3.0859205644<br>H,0,9.7228906647,-3.9812453475,2.9812189886<br>H,0,11.3422780375,-3.9108481038,4.8452073385<br>H,0,10.5921968571,-3.3216423957,7.1198751992<br>C,0,6.23760645,-2.7987906514,5.7626339517<br>C,0,8.0794450624,-2.7192269446,7.8522811788<br>C,0,-2.7352536964,2.7368713536,-0.6516980534<br>H,0,-1.1745982948,1.3913558686,-0.109728509<br>H,0,-2.1378231152,3.6191842801,-0.4599941523<br>C,0,-4.0628604896,2.8758930513,-1.0903106767<br>H,0,-5.8537533824,1.8640738062,-1.6601964374<br>H,0,-4.479723399,3.8643296452,-1.2363162062<br>C,0,6.7611868947,-2.4357590083,8.0863297524<br>H,0,8.8013937535,-2.6896716336,8.6602673919<br>H,0,6.428946155,-2.1791901435,9.0844516188<br>C,0,5.8335742332,-2.4766142479,7.0313841237<br>H,0,5.5241639981,-2.8288102731,4.9474170785<br>H,0,4.7929878704,-2.2502418832,7.2258928076
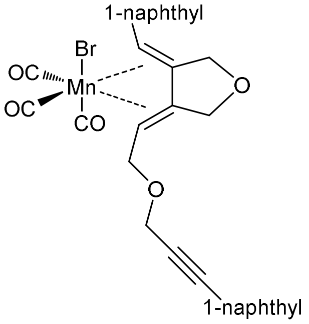	Br,0,2.4421270163,-0.3229758635,-0.284346089<br>Mn,0,-0.0233254209,-0.0061026736,0.0888991778<br>C,0,-0.2937778467,-0.8035706521,-1.6069908372<br>O,0,-0.4835566279,-1.2434835303,-2.6500831998<br>C,0,0.1523527448,1.6398064123,-0.7938259284<br>O,0,0.2620550826,2.596496983,-1.4202936595<br>C,0,-0.1459065759,-1.7755290413,1.0818182309<br>C,0,-0.2798285611,-3.1078186281,0.5283077855<br>C,0,0.6491831329,-3.522265766,-0.4306211064<br>C,0,0.6976478181,-4.8549133819,-0.8715021794<br>C,0,-0.2210679682,-5.7747009612,-0.4123291221<br>C,0,-1.2443881712,-5.3901021759,0.491381762<br>C,0,-1.2899680508,-4.0388485393,0.965905772<br>C,0,-0.1124763385,-1.5825096574,2.4815060662<br>C,0,-0.0073672974,-2.4662953978,3.6877426982<br>O,0,0.3112764851,-1.5878008067,4.7578885002<br>C,0,0.0332503686,-0.2511827565,4.394072682<br>C,0,-0.045321263,-0.2632309136,2.9052703438<br>C,0,-0.0100507973,0.7442793193,1.9095038693<br>C,0,-0.1225960469,2.1660217904,2.3479083601<br>O,0,0.6467929098,3.0904243853,1.6285920727<br>C,0,2.0542682491,2.8470315907,1.5857216711<br>C,0,2.538619053,1.8122620238,2.4767387491<br>H,0,1.3916719743,-2.8009604843,-0.7833979146

	C,0,-2.3762411452,-3.6525003608,1.7864981733<br>C,0,-2.2401120452,-6.3050422009,0.9148303643<br>H,0,1.4693665461,-5.1559175593,-1.582858301<br>H,0,-0.1901315606,-6.8112585704,-0.7583294593<br>C,0,-1.7969283947,0.1106289343,0.4593230015<br>O,0,-2.9113276828,0.1691830614,0.7619355414<br>H,0,-0.9278089891,0.1007397341,4.8313807351<br>H,0,0.8157565611,0.4133998042,4.8002985687<br>H,0,0.7879263278,-3.2267138328,3.5929598191<br>H,0,-0.9464849717,-3.0161382097,3.8986831399<br>H,0,0.0871061214,2.2528180067,3.4351449235<br>H,0,-1.170386783,2.4968028241,2.2210695818<br>H,0,2.3405860872,2.566397895,0.5542861069<br>H,0,2.5501395676,3.808786054,1.8000257023<br>C,0,2.9102214508,0.8667155557,3.1551762046<br>C,0,3.2457055753,-0.3011141086,3.8902852925<br>C,0,3.320750485,-1.522277618,3.2238452202<br>C,0,3.5766027639,-2.7110585889,3.9302139534<br>C,0,3.7402888027,-2.6901420712,5.2982247353<br>C,0,3.4270604164,-0.2518399201,5.3136676676<br>C,0,3.6707860692,-1.4723049314,6.0203584892<br>H,0,3.1665140135,-1.5378423112,2.1409920608<br>H,0,3.6363752063,-3.655337944,3.3836467505<br>H,0,3.9290640754,-3.6152120607,5.8494630536<br>C,0,3.3563338311,0.9579583847,6.0442518003<br>C,0,3.8345489956,-1.426765386,7.4278285103<br>C,0,-3.3355163382,-4.5655006483,2.1777500158<br>H,0,-2.4731311401,-2.60520815,2.0842722119<br>H,0,-4.1662742695,-4.2423846977,2.8088480262<br>C,0,-3.2611470143,-5.9059910013,1.7501579805<br>H,0,-2.1922690652,-7.3346453187,0.5507193771<br>H,0,-4.0251674085,-6.6204362811,2.0638327284<br>C,0,3.5174605545,0.9679070294,7.4134986626<br>H,0,3.174159045,1.8882961404,5.4991695791<br>H,0,3.4611628394,1.9111275278,7.9616660412<br>C,0,3.7593096101,-0.234159547,8.1122417921<br>H,0,4.0208092942,-2.3619349885,7.9624080779<br>H,0,3.8865213997,-0.2154095206,9.1968812666
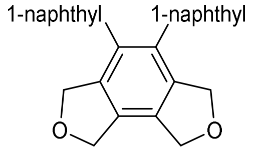<br><br>Dinaphthylbenzene derivative	C,0,1.3572588214,-1.011250533,2.2995552144<br>C,0,-1.013734709,0.4510018014,2.6391320198<br>C,0,0.1212286046,-1.5922501651,1.9550326462<br>C,0,1.3628280992,0.2903837334,2.8003403274<br>C,0,0.1981964682,1.0234117453,2.9686861055<br>C,0,-1.0394882408,-0.8404668135,2.1356612903<br>H,0,1.0664313114,-2.3495999955,-0.4102238216<br>C,0,0.5883207629,-3.171697023,0.1103494671<br>C,0,0.0528468726,-2.9494425947,1.3593616786<br>C,0,-0.0785780255,-5.4802921996,0.1437437484<br>C,0,-0.5580581589,-4.0289411606,2.0586924889<br>C,0,0.5236627794,-4.4338077734,-0.4995600563<br>C,0,-0.6274847511,-5.3108712324,1.4322838775<br>C,0,-1.0703140695,-3.8938885531,3.3697714788<br>H,0,0.9504668947,-4.5710326738,-1.4851454391<br>H,0,-1.2872179971,-7.3476411358,1.6422556372<br>H,0,-0.1373614172,-6.4568440061,-0.3229082956<br>C,0,-1.6441626785,-4.9545683558,4.0188874164<br>H,0,-0.9949417121,-2.9312324451,3.8623879022<br>H,0,-2.0288448311,-4.8289227974,5.0232970765<br>C,0,-1.7341730119,-6.208950883,3.3903925213<br>H,0,-2.1936990504,-7.0395857292,3.911293042<br>C,0,-1.2330089406,-6.3802946345,2.1287108904

	H,0,2.0477260602,-3.1615062574,3.6999410159<br>C,0,2.8258356819,-2.872694673,3.0024002978<br>C,0,2.621570379,-1.7799712274,2.189755246<br>C,0,5.0088286433,-3.2591443315,2.0746722918<br>C,0,3.6297791062,-1.4067291016,1.2560576658<br>C,0,4.0168142392,-3.6127579952,2.9479009132<br>C,0,4.8433595025,-2.158673874,1.207537667<br>C,0,3.4651262326,-0.3384926553,0.3442826011<br>H,0,4.1455339817,-4.4630454344,3.6057865199<br>H,0,6.7617735815,-2.3593647686,0.2550785753<br>H,0,5.9338355451,-3.8224119099,2.030344931<br>C,0,4.4510193314,-0.0102728765,-0.5478956012<br>H,0,2.5350541173,0.2181857273,0.3558477989<br>H,0,4.3030712722,0.8108976184,-1.2381102927<br>C,0,5.6548713846,-0.7358817162,-0.5764484223<br>H,0,6.4292896211,-0.4650066837,-1.2831017263<br>C,0,5.8413886881,-1.78697663,0.2795746048<br>C,0,-2.4548888937,-1.1832468729,1.802473248<br>H,0,-2.5991955368,-1.2834781012,0.7160900113<br>H,0,-2.8003151481,-2.1160897264,2.2597290329<br>C,0,-2.4028339706,0.9889982766,2.6653898315<br>H,0,-2.7059062566,1.36645538,3.6490794415<br>H,0,-2.5304349258,1.8172575564,1.9508026296<br>C,0,2.5078063129,1.1138731122,3.2930487423<br>H,0,3.3160114918,1.2240692119,2.5628027919<br>H,0,2.951460293,0.6819774391,4.2028490043<br>C,0,0.5398308672,2.347413567,3.5603207498<br>H,0,0.1446168214,2.4485225896,4.5833129937<br>H,0,0.1533075638,3.1957978818,2.9834189257<br>O,0,1.9616220778,2.4041374185,3.5639793719<br>O,0,-3.2395218954,-0.1078190048,2.3165827284

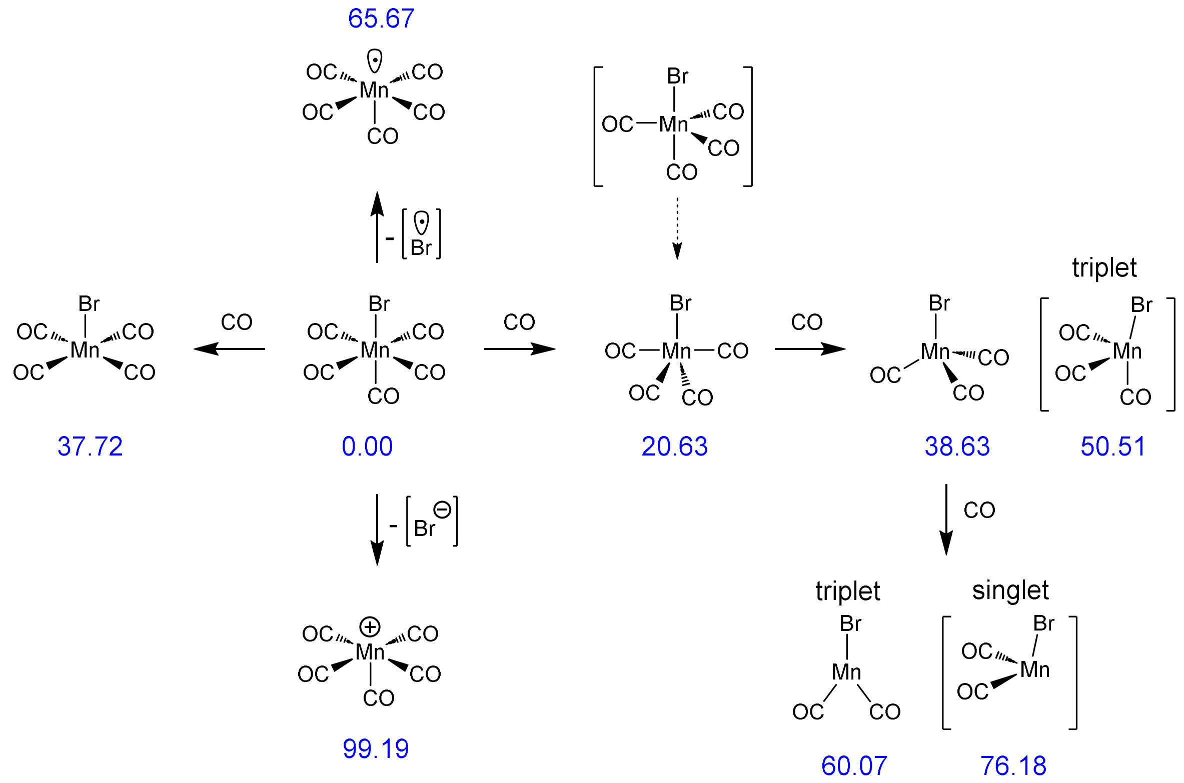

Figure SI-14: CO de-coordination vs. Br-Mn homolytic and heterolytic dissociation (ΔG, kcal/mol).

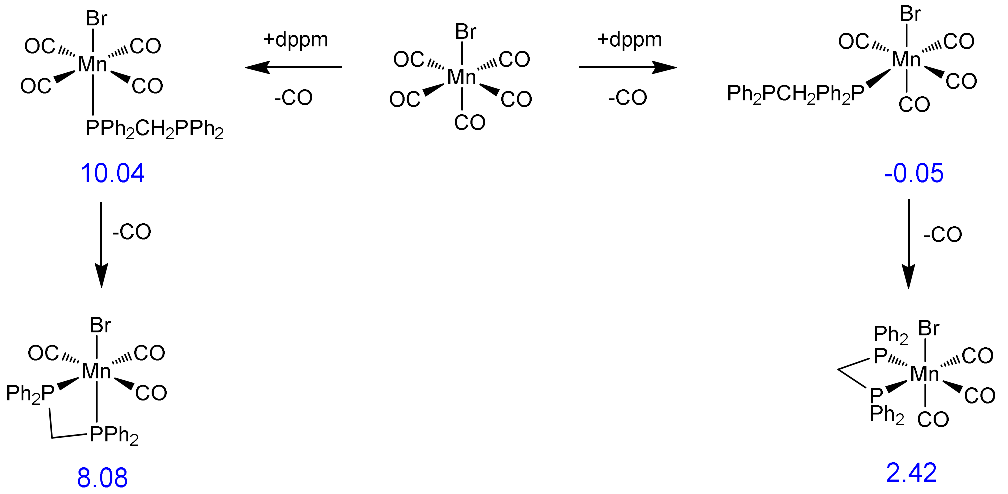

Figure SI-15: Sequential substitution energy of CO by dppm ligand (ΔG, kcal/mol).

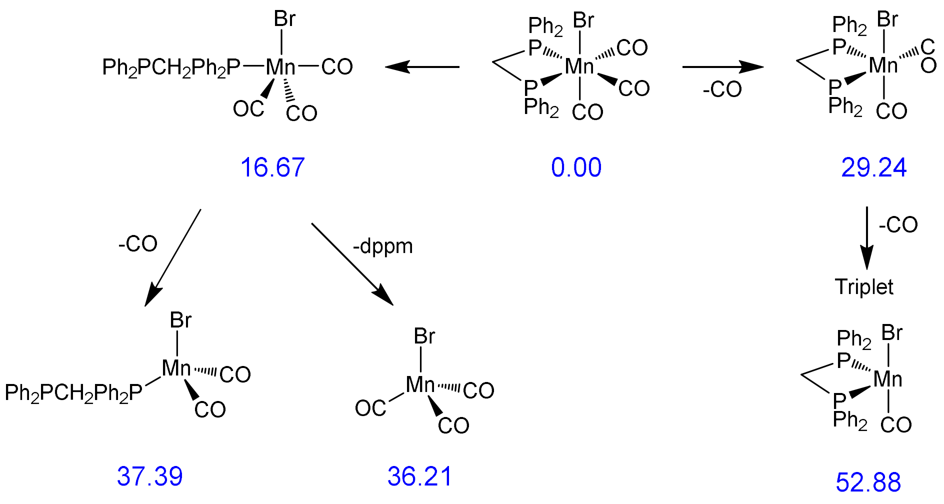

Figure SI-16: Sequential de-coordination energy of CO vs. dppm (Δ*G*, kcal/mol).

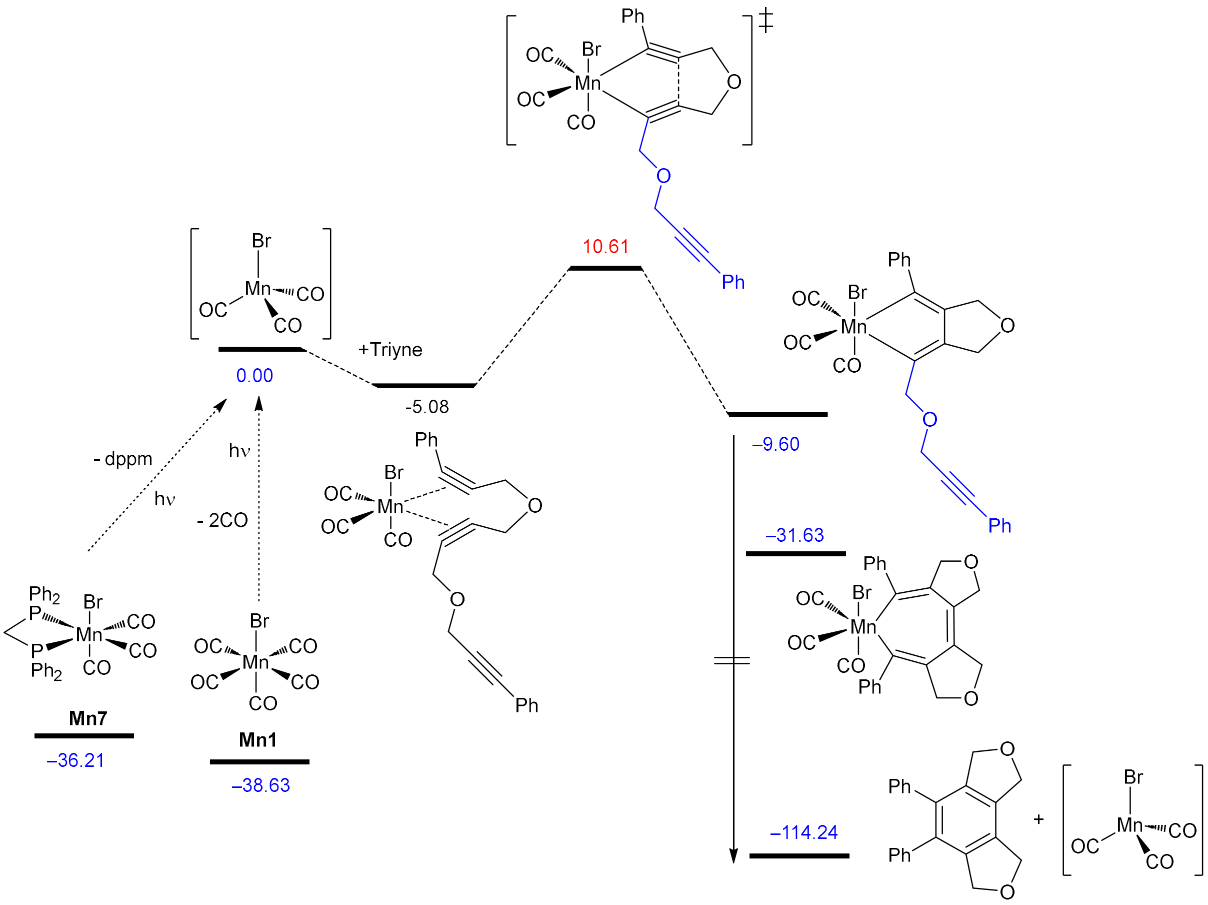

Figure SI-17: Mechanistic proposal for Mn-catalyzed cyclization reaction using **Mn1** and **Mn7** (Δ*G*, kcal/mol).

6 References

(1) Sawall, M.; Kubis, C.; Barsch, E.; Selent, D.; Börner, A.; Neymeyr, K. Peak Group Analysis for the Extraction of Pure Component Spectra. *J. Iran. Chem. Soc.* **2016**, *13*, 191-205.
(2) Webpage: https://www.math.uni-rostock.de/facpack/ (last accessed: 02.09.2024).
(3) Kireev, N. V.; Filippov, O. A.; Gulyaeva, E. S.; Shubina, E. S.; Vendier, L.; Canac, Y.; Sortais, J.-B.; Lugan, N.; Valyaev, D. A. Bis[diphenylphosphino]methane and its bridge-substituted analogues as chemically non-innocent ligands for H_2 activation. *Chem. Commun.* **2020**, *56*, 2139-2142.
(4) Saino, N.; Amemiya, F.; Tanabe, E.; Kase, K.; Okamoto, S. A highly practical instant catalyst for cyclotrimerization of alkynes to substituted benzenes. Okamoto, *Org. Lett.* **2006**, *8*, 1439-1442.
(5) Ojima, I.; Vu, A. T.; McCullagh, J. V.; Kinoshita, A. Rhodium-Catalyzed intramolecular silylcarbotricyclization (SICAT) of triynes. *J. Am. Chem. Soc.* **1999**, *121*, 3230-3231.
(6) Yamamoto, Y.; Nagata, A.; Nagata, H.; Ando, Y.; Arikawa, Y.; Tatsumi, K.; Itoh, K. Palladium(0)-Catalyzed Intramolecular [2+2+2] Alkyne Cyclotrimerizations with Electron-Deficient Diynes and Triynes. *Chem. Eur. J.* **2003**, *8*, 2469-2483.
(7) Fernández, M.; Ferré, M.; Pla-Quintana, A.; Parella, T.; Pleixats, R.; Roglans, A. Rhodium-NHC hybrid silica materials as recyclable catalysts for [2+2+2] cycloaddition reactions of alkynes. *Eur. J. Org. Chem.* **2014**, 6242-6251.
(8) Iannazzo, L.; Vollhardt, K. P. C.; Malacria, M.; Aubert, C.; Gandon, V. Alkynylboronates and -boramides in CoI- and RhI-Catalyzed [2+2+2] Cycloadditions: Construction of Oligoaryls through Selective Suzuki Couplings. *Eur. J. Org. Chem.* **2011**, *2011*, 3283-3292.
(9) Baumann, B. N.; Lange, H.; Seeberger, F.; Büschelberger, P.; Wolf, R.; Hapke, M. Cobalt and iron metallates as catalysts for cyclization reactions of diynes and triynes: [2+2+2] Cycloaddition vs. Garratt-Braverman reaction. *Mol. Catal.* **2023**, *550*, 113482.
(10) Geny, A.; Agenet, N.; Iannazzo, L.; Malacria, M.; Aubert, C.; Gandon, V. Air-Stable {$(C_5H_5)Co$} catalysts for [2+2+2] cycloadditions. *Angew. Chem. Int. Ed.* **2009**, *48*, 1810-1813.
(11) Shibata, T.; Tsuchikama, K.; Otsuka, M. Enantioselective intramolecular [2+2+2] cycloaddition of triynes for the synthesis of atropisomeric chiral ortho-diarylbenzene derivatives. *Tetrahedron Asymmetry* **2006**, *17*, 614-619.
(12) Jančařík, A.; Rybáček, J.; Cocq, K.; Chocholoušová, J. V.; Vacek, J.; Pohl, R.; Bednárová, L.; Fiedler, P.; Císařová, I.; Stará, I. G.; Starý, I. Rapid Access to Dibenzohelicenes and their Functionalized Derivatives. *Angew. Chem. Int. Ed.* **2013**, *52*, 9970-9975.
(13) Nakajima, K.; Takata, S.; Sakata, K.; Nishibayashi, Y. Synthesis of Phosphabenzenes by an Iron-Catalyzed [2+2+2] Cycloaddition Reaction of Diynes with Phosphaalkynes. *Angew. Chem. Int. Ed.* **2015**, *54*, 7597-7601.
(14) Chen, L.; Ren, P.; Carrow, B. P. Tri(1-adamantyl)phosphine: Expanding the Boundary of Electron-Releasing Character Available to Organophosphorus Compounds. *J. Am. Chem. Soc.* **2016**, *138*, 6392-6395.
(15) Mallagaray, A.; Medina, S.; Dominiguez, G.; Perez-Castells, J.; Ruthenium Carbene Mediated [2+2+2] Cyclotrimerizations. *Synlett*, **2010**, *14*, 2114-2118.
(16) Yang, K.; Wang, P.; Sun, Z.-Y.; Guo, M.; Zhao, W.; Tang, X.; Wang, G.; Hydrogen-Bonding Controlled Nickel-Catalyzed Regioselective Cyclotrimerization of Terminal Alkynes. Org. Lett., **2021**, *23*, 3933–3938.
(17) Brenna, D.; Villa, M.; Gieshoff, T. N.; Fischer, F.; Hapke, M.; Jacobi von Wangelin, A.; Iron-catalyzed Cyclotrimerization of Terminal alkynes by Dual Catalyst Activation in the Absence of Reductants. Angew. Chem. Int. Ed. **2017**, *56*, 8451-8454.
(18) Weding, N.; Jackstell, R.; Jiao, H.; Spannenberg, A.; Hapke, M. Synthesis of Group 9 Metal-Olefin Complexes with Identical Ligand Frameworks and Comparison of their Catalytic Activity in [2+2+2] Cycloaddition and other Addition Reactions. *Adv. Synth. Catal.* **2011**, *353*, 3423-3433.
(19) Teng, Y.-H. G.; Chien, C.-W.; Chiou, W.-H.; Honda, T.; Ojima, I. Construction of fused tropone systems through intramolecular Rh(I)-Catalyzed carbonylative [2+2+2+1] cycloadditon of triynes. *Front. Chem.* **2018**, *6*, 401.
(20) Jungk, P.; Fischer, F.; Thiel, I.; Hapke, M. $CoCl(PPh_3)_3$ as Cyclotrimerization Catalyst for Functionalized Triynes under Mild Conditions. *J. Org. Chem.* **2015**, *80*, 9781-9793.
(21) Görlich, T.; Frost, D. S.; Boback, N.; Coles, N. T.; Dittrich, B.; Müller, P.; Jones, W. D.; Müller, C. Photochemical C(sp)–C(sp2) Bond Activation in Phosphaalkynes: A New Route to Reactive Terminal Cyaphido Complexes LnM–C≡P. *J. Am. Chem. Soc.*, **2021**, *143*, 19365-19373.
(22) Ruhl, K. E.; Rovis, T. Visible Light-Gated cobalt catalysis for a spatially and temporally resolved [2+2+2] cycloaddition. *J. Am. Chem. Soc.* **2016**, *138*, 15527-15530.
(23) Blond, G.; Bour, C.; Salem, B.; Suffert, J. A new Pd-Catalyzed cascade reaction for the synthesis of strained aromatic polycycles. *Org. Lett.* **2008**, *10*, 1075-1078.
(24) Mandal, J.; Prasad, S. K.; Rao, D. S. S.; Ramakrishnan, S. Periodically clickable polyesters: Study of intrachain Self-Segregation induced folding, crystallization, and mesophase formation. *J. Am. Chem. Soc.* **2014**, *136*, 2538-2545.

(25) Banti, D.; Groaz, E.; North, M. Ene–yne metathesis of polyunsaturated norbornene derivatives. *Tetrahedron* **2004**, *60*, 8043-8052.
(26) Göbel, M.; Lautens, M.; Stark, T.; Suhartono, M. A Palladium-Catalyzed domino reaction as key step for the synthesis of functionalized aromatic amino acids. *Synlett* **2013**, *24*, 2730-2734.
(27) Mukai, C.; Hara, Y.; Miyashita, Y.; Inagaki, F. Thermal [2+2] Cycloaddition of Allenynes: Easy Construction of Bicyclo[6.2.0]deca-1,8-dienes, Bicyclo[5.2.0]nona-1,7-dienes, and Bicyclo[4.2.0]octa-1,6-dienes. *J. Org. Chem.* **2007**, *72*, 4454-4461.
(28) Caldarelli, S. A.; Fangour, S. E.; Wein, S.; Van Ba, C. T.; Périgaud, C.; Pellet, A.; Vial, H. J.; Peyrottes, S. New bis-thiazolium analogues as potential antimalarial agents: design, synthesis, and biological evaluation. *J. Med. Chem.* **2013**, *56*, 496-509.
(29) He, Y.-T.; Karimata, A.; Gladkovskaya, O.; Khaskin, E.; Fayzullin, R. R.; Sarbajna, A.; Khusnutdinova, J. R. C–C Bond Elimination from High-Valent Mn Aryl Complexes. *Organometallics* **2021**, *40*, 2320-2331.
(30) Nitschke, J.; Schmidt, S. P.; Trogler, W. C. Properties of (trifluoromethanesulfonato)pentacarbonylmanganese(I) and -rhenium(I). Reactions in superacid solvents. *Inorg. Chem.* **1985**, *24*, 1972-1978.
(31) Sattler, W.; Ener, M. E.; Blakemore, J. D.; Rachford, A. A.; LaBeaume, P. J.; Thackeray, J. W.; Cameron, J. F.; Winkler, J. R.; Gray, H. B. Generation of powerful tungsten reductants by visible light excitation. *J. Am. Chem. Soc.* **2013**, *135*, 10614-10617.
(32) Son, S. U.; Paik, S.-J.; Lee, S. I.; Chung, Y. K. The strategy for $Co_2(CO)_8$-catalyzed double carbonylative [2+2+1] cycloaddition or [2+2+2] cycloaddition reaction of triynes: a new synthetic method for tetracyclic compounds. *J. Chem. Soc., Perkin Trans.* **2000**, *1*, 141-144.
(33) Bennacer, B.; Fujiwara, M.; Lee, S.-Y.; Ojima, I. Silicon-Initiated carbonylative carbotricyclization and [2+2+2+1] cycloaddition of enediynes catalyzed by rhodium complexes. *J. Am. Chem. Soc.* **2005**, *127*, 17756-17767.
(34) Luo, M.; Qin, Y.; Chen, X.; Xiao, Q.; Zhao, B.; Yao, W.; Ma, M. $ZnBr_2$-Catalyzed dehydrogenative borylation of terminal alkynes. *J. Org. Chem.* **2021**, *86*, 16666-16674.
(35) Zhu, B. C.; Jiang, X. Z. A new CuAl–hydrotalcite catalyzed homocoupling reaction of terminal alkynes at room temperature. *Appl. Organomet. Chem.* **2007**, *21*, 345-349.
(36) Hoheisel, T. N.; Frauenrath, H. A Convenient Negishi Protocol for the Synthesis of Glycosylated Oligo(ethynylene)s. *Org. Lett.* **2008**, *10*, 4525-528.
(37) Coles, B. F.; Walton, D. R. M. The preparation of Silyl-Protected Functional Penta-1,3-diynes. *Synthesis* **1975**, *6*, 390-391.
(38) Kuciński, K.; Hreczycho, G. Transition Metal-Free Catalytic C−H Silylation of Terminal Alkynes with bis(Trimethylsilyl)acetylene Initiated by KHMDS. *ChemCatChem* **2022**, *14*, e202200794.
(39) M. J. Frisch, *et al.*, Gaussian 09, Revision C.01, Gaussian, Inc., Wallingford CT, 2010.
(40) Zhao, Y.; Truhlar, D. G. A new local density functional for main-group thermochemistry, transition metal bonding, thermochemical kinetics, and noncovalent interactions. *J. Chem. Phys.*, 2006, **125**, 194101.
(41) Schäfer, A.; Huber, C.; Ahlrichs, R. Fully optimized contracted Gaussian basis sets of triple zeta valence quality for atoms Li to Kr. *J. Chem. Phys.*, 1994, **100**, 5829-5835.
(42) Marenich, A. V.; Cramer, C. J.; Truhlar, D. G. Universal solvation model based on solute electron density and on a continuum model of the solvent defined by the bulk dielectric constant and atomic surface tensions. *J. Phys. Chem. B*, 2009, **113**, 6378-6396.
